# Prognostic factors of microsurgical treatment of intracranial meningiomas – A multivariate analysis

**DOI:** 10.1371/journal.pone.0202520

**Published:** 2018-10-16

**Authors:** Maika Kreßner, Felix Arlt, Wolf Riepl, Jürgen Meixensberger

**Affiliations:** 1 Department of Neurosurgery, University Hospital Leipzig, Leipzig, Germany; 2 Social Science Research, Dresden, Germany; Universidad de Navarra, SPAIN

## Abstract

**Objective:**

Peri- and postoperative time course of meningioma patients who had undergone surgical treatment was evaluated to determine prognostic factors of neurological outcome by focusing on preoperative parameters.

**Material and methods:**

A retrospective monocenter analysis was performed including patients who were operated in the Department of Neurosurgery, University Hospital Leipzig, from 2009 to 2015. Data from all patients with histologically confirmed diagnosis of intracranial meningioma treated microsurgically were included in the study. The individual characteristics of the patients, meningiomas and Karnofsky Performance scores (KPS) were analyzed by multivariate tests.

**Results:**

Two hundred ninety-four patients with a median age of 61 years (range: 17–89) were included. Preoperative KPS (p < 0.001), skull base as tumor origin and tumor size (p < 0.05) proved to be significantly strong prognostic factors of KPS deterioration one year postoperative by multivariate analysis. According to uni- and bivariate analysis, the following prognostic factors could also be found: preoperative mass displacement, preexisting recurrence and presence of preoperative symptoms. In this study, age had no significant influence on deterioration in patient health state, measured by KPS, one year postoperative.

**Conclusion:**

Patients generally obtained an improvement in KPS score after microsurgical treatment. The knowledge of prognostic factors can be very helpful in the decision-making process for meningioma treatment of the elderly, particularly to estimate the postoperative outcome and quality of life.

## Introduction

Meningioma is the most common primary brain tumor and constitutes approximately 20–30% of all intracranial neoplasm [1–3,4]. The incidence of meningioma amounts to 2.3–7:100,000 people, and between 80 and 85% of these intracranial tumors are regarded as histologically benign (WHO I)[5–7]. Due to the frequency of occurrence of intracranial meningioma in the population, it is necessary to be aware of the influencing factors and utilize the knowledge acquired efficiently to exert a positive influence on the course of the disease after surgery.

A series of comparable studies referring to meningioma patients and their prognostic factors in cases of surgery experienced already exists, but only in patient collectives with a special focus, for instance, on age, tumor grade, extent of surgical removal, tumor location or irradiation therapy[8–10]. However, there has been no examination performed which investigated risk factors or predictors in meningioma patients without such limitations in the last few years. Therefore, the subject of the study was a general observation of the patient group related to preoperative prognostic factors.

Medical development does not stand still, and this process is moving forward continuously, based on the innovations and improvements in microsurgical technology and diagnostic imaging. Therefore, there is a change in the prerequisite for surgery referring to the conditions of the patient. Patients are living longer, which also means that more patients who are elderly will undergo surgery compared to previously. Consequently, performing an update is necessary.

The aim of the present study was to identify preoperative prognostic factors for patients with a diagnosis of intracranial meningioma undergoing surgical treatment and to examine whether there is a general benefit of surgery in patients suffering from intracranial meningioma. The postoperative outcome for evaluating prognostic factors was analyzed using the Karnofsky Performance Status (KPS) score to measure the degree of disability. Regarding especially the change in KPS, provides the possibility of assessing negative predictors which lead to a deterioration in the patient’s functional status after surgery and, consequently, at best, considering the influence of the postoperative course of disease.

## Patients and methods

### Patient’s characteristics

All patients with intracranial meningioma who underwent surgery for primary and recurrent tumors at the Department of Neurosurgery, University Hospital Leipzig, between 2009 and 2015 were selected. The total number of patients included was 294. The WHO grade and histological subtype are given in Table 1. All patients who suffered from a spinal meningioma were excluded from the study. Each patient was considered only once. Only the first surgery could be considered during the evaluation period due to formal statistical reasons. The subsequent surgeries were excluded from the analysis. Patient data were obtained retrospectively from the SAP Patient Administration program. The study design and analysis were approved by our local ethics committee (Ethics Committee at the Medical Faculty University Leipzig AZ: 152/17-ek). Data was recorded and evaluated completely anonymized. A declaration of consent is not required because of the study’s retrospective character.

**Table 1:** 

Histology	WHO grade	*Subtype*
Grade I	243 (82,7%)	*Meningothelial 127 (43*,*2%)*
Grade II	46 (15,6%)	*Transitional 58 (19*,*7%)*
Grade IV	5 (1,7%)	*Fibrous 27 (9*,*2%)*
		*Others* * *37 (12*,*6%)*

WHO Grading and histological subtype of meningiomas included

* others = psammomatous, angiomatous, microcystic, clear-cell, metaplastic

### Methods

The analysis of relevant data was based on a list of 89 variables which contains information on the following categories: preoperative status (e.g. patient-specific data, tumor-related data), postoperative facts (e.g. complications, recurrence) and assessment of functional status (Table 1, Table 2).

**Table 2:** 

Preoperative data			Postoperative data
*Patient-related*	Tumor-related	*Diagnostic intervention*	Histology
*Gender*, *age (years)*	Number of tumors	*CCT/MRi*	WHO grade
*Duration of Symptoms (month)*	Size (>,< 4 cm)	*CSF circulation*	Subtypes
*First symptom*	Location	*Tumor edema*	
*Neurological symptoms* *		*Mass effect*	
Risk factors**		*Preoperative embolization*	
*ASA score*			
*Radiation*			
*Preexisting recurrence*			

List of variables of patients operated for meningioma

* Headache attacks, nausea, emesis, ophthalmoplegia, visual impairment, scotoma, exophthalmos, an-par-kakosmia, diplopia, papilledema, optic atrophy, hemiparesis or cerebellar symptoms, dysfunctional concentration, orientation, memory or personality changes, motor disorders, reduction of vigilance or dysphasia

** Arterial hypertonia, heart disease, obesity, lung disease, liver disease, kidney disease, diabetes mellitus or varicosis

*** Falx, parasagittal, convexity, sphenoid bone, sella, olfactory, tentorium, clivus, orbit, intraventricular, skull base, petrosal bone or cerebellopontine angle

The degree of disability was used as a criterion for the prognosis of disease in this study. The KPS Index[11] was utilized as an assessment tool for evaluation.

The KPS Index contains a percentage value scale from 0 to 100 in ten-percent steps. The lower the KPS score, the worse the functional impairment and survival for most serious illnesses. The KPS was determined from the patient’s data available at three different times:

Immediately preoperative                                       (K1)

Between three and six months postoperative        (K2)

About one year postoperative                                (K3)

In addition, the difference between K1 and K3 was gathered (Kd) as an indicator of change in the patient’s condition by comparing pre- and postoperative functional status. Statistical tests were performed to determine preoperative prognostic factors based on the KPS scores. Data regarding the tumor grade and neuropathological histological subtype were collected according to the 2007 WHO classification of tumors of the central nervous system[12]. The American Society of Anesthesiologists (ASA) classification[13] was used to describe the preoperative health state of all patients included. The ASA scores were obtained from anesthesia protocols.

A nearly complete data set could be recorded: Only eight patients missed a checkup between three and six months postoperative (K2), 40 cases one year after and 40 missing values regarding Kd.

## Statistical analysis

The statistical evaluation was performed by IBM SPSS Statistics (version 24.0, Inc., Chicago, USA). The significance level was determined as p < 0.05 (*), p < 0.01 (**) or < 0.001 (***) to quantify and compare the various results. All p-values were two-sided. The Spearman coefficient (rho) was used to detect correlation between different metric variables which are not normal distributed, for example, between age and KPS score. The Mann-Whitney U test was used to compare measured values which were not normally distributed, for instance, the KPS indices, with dichotomously classified variables, such as gender. The Chi-squared test was used to analyze the relationship between nominally scaled and categorical variables (e.g. localization of tumor and gender). The Kruskal-Wallis test, an extension of the U test, was utilized to compare KPS values with nominally classified variables with more than two value labels.

Binary logistic regression was used to find several predictor variables, which estimate the probability that the characteristic of Kd_di (1 = Kd < 0/postoperative aggravation of patients’ state of health) is present.

The factor analysis finally used is a statistical method for the general description of the relationship between a series of parameters by summarizing them to factors which are independent of each other. Consequently, it was necessary to dichotomize all nominal variables with more than two value labels.

## Results

### Preoperative data

**Fig 1:**
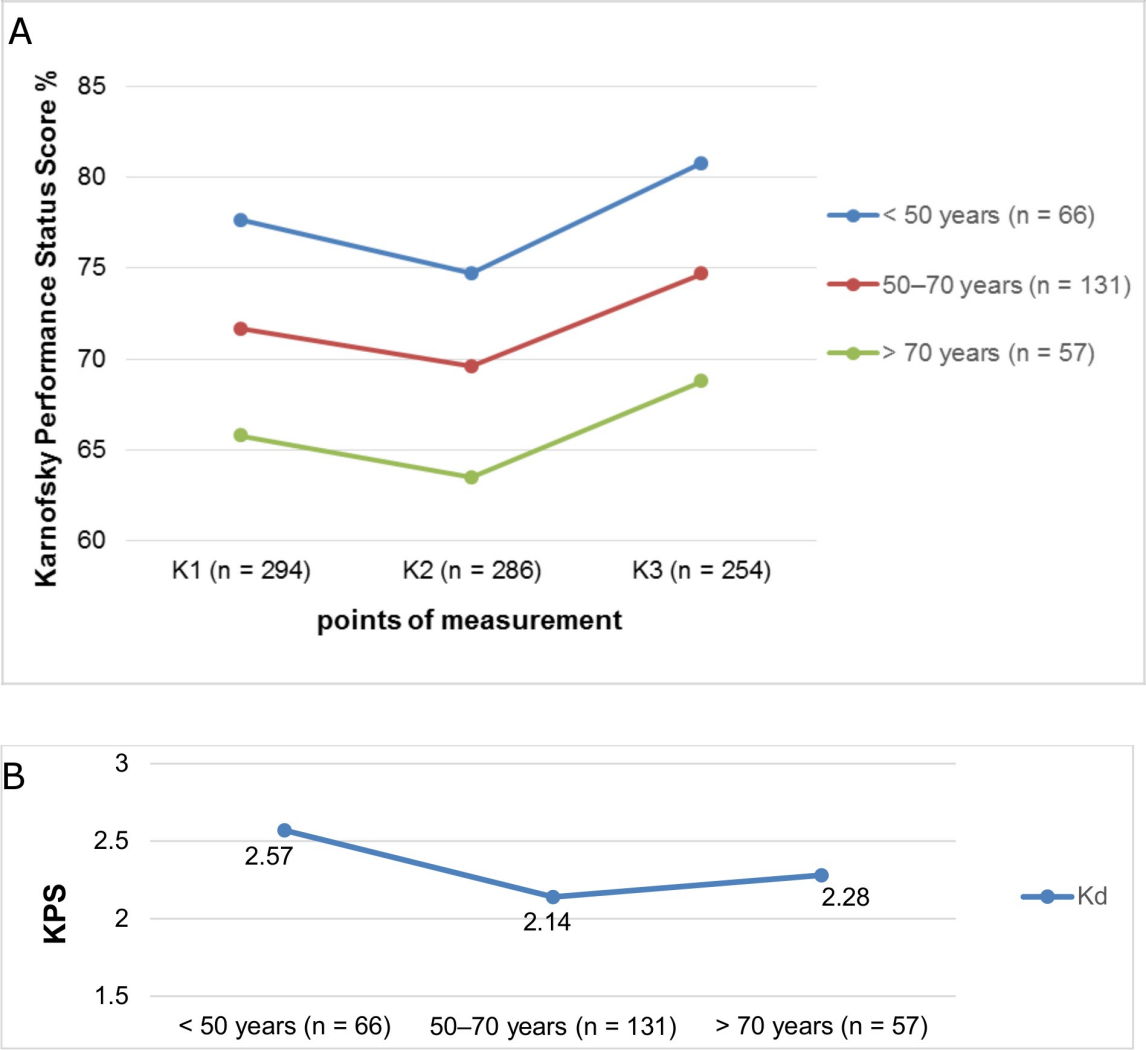
a, b Karnofsky Performance Score (KPS) at three points of measurement and Kd in relation to the patient’s age (50 years vs. 50–70 years vs. > 70 years), K1 = preoperative; K2 = 3–6 months postoperative; K3 = 1 year postoperative; Kd = difference between K3 and K1.

**Fig 2:**
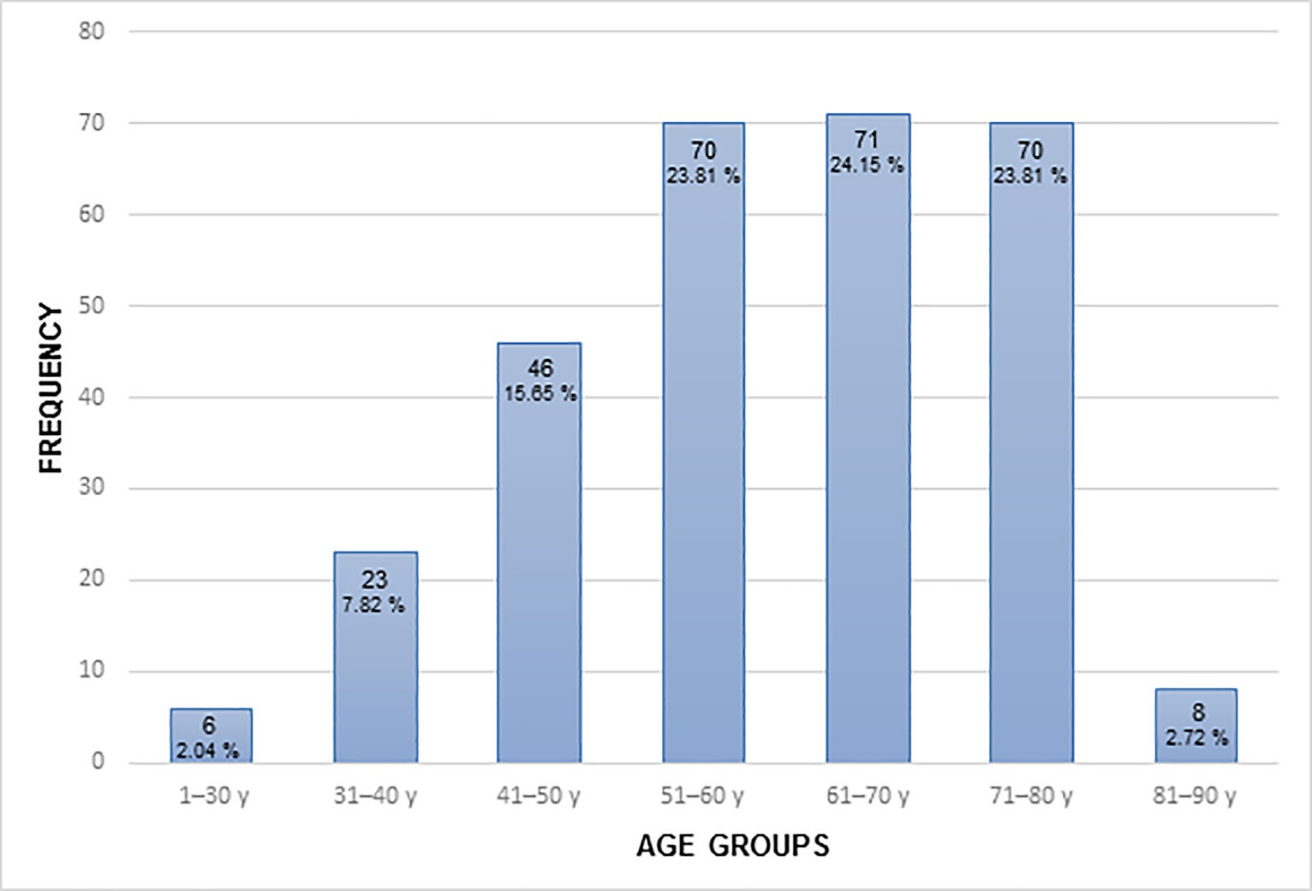
Distribution of patient’s age at time of surgery (groups: 1–30 years, 31–40 years, 41–50 years, 51–60 years, 61–70 years, 71–80 years and 81–90 years).

The mean age of the 294 patients included was 59.8 years (SD 13.60 years), 200 patients (68%) were female and 94 were male (32%). The female-to-male ratio was 2:1 (Table 3). Age exhibited an inverse correlation with K1-3 (rho = -0.36/-0.41, -0.39, p < 0.01). It could be proven that significant differences in K1–3 between both groups (p < 0.001, Mann-Whitney U test) also exist by creating two groups with a cut-off age of 65 years. Similar results could be achieved by subdividing age into three groups (< 50 years vs. 50–70 years vs. > 70 years). Significant differences in K1, K2 and K3 values (p < 0.001) were determined between the various groups by using the Kruskal-Wallis test, but without any influence on Kd (p > 0.05) (Fig 1A, Fig 1B, Fig 2).

**Table 3:** 

Preoperative patient related data		
Sex	200 (68.0%) Female	94 (32%) Male
Mean/Median age at surgery (range) (years)	59.8 (61.0) (17–89)	(SD * 13.60)
Age subgroups (years)	*< 50* (n = 71, 24.1%)	*50–70* (n = 145, 49.3%)
	*> 70* (n = 78, 26.5%)	*> 80* (n = 8, 2.7%)
Presence of neurological symptoms	*yes* 250 (85.0%)	*no* 44 (15.0%)
Presence of risk factors **	*yes* 202 (68.7%)	*no* 92 (31.3%)
Mean/Median duration of symptoms`presence (month)	9.14 (3.00)	(SD 17.19)
ASA score	I	19 (6.5%)
	II	188 (64.0%)
	III	83 (28.2%)
	IV	3 (1.0%)
	V	1 (0.3%)
Recurrence already	*yes* 31 (10.5%)	*no* 263 (89.5%)
Radiation preoperative	*yes 10 (3*.*4%)*	*no* 284 (96.6%)

Preoperative characteristics of 294 patients with meningioma

* SD: standard deviation

** Arterial hypertonia, heart disease, obesity, lung disease, liver disease, kidney disease, diabetes mellitus or varicosis

The majority (85%) showed clinical symptoms with an average duration of 9.14 months. Patients with preoperative disorders had a substantially lower K1 score (mean of 70.08 vs. 79.77), but much better Kd issue (mean of 3.58 vs. -4.29, p < 0.001, Mann-Whitney U test). The most common initial disorders were optical symptoms (20.3%), headaches (17.0%), cerebellar symptoms (14.5%) and epileptic seizures (14.5%).

A total of 202 patients (68.7%) presented risk factors, such as arterial hypertension (39.9%), obesity (24.0%), heart disease (12.8%) and diabetes mellitus (11.3%). Regarding preoperative health status, 6.5% of patients were assigned to ASA 1, 63.9% to ASA 2, 28.2% to ASA 3, three cases to ASA 4 and only one case to ASA 5. A significance difference of ASA groups (p < 0.001) in K1–K3 could be shown by the Kruskal-Wallis test. The worse the ASA score, the better the KPS scores K1–K3, which was confirmed by Spearman’s correlation (rho: -0.36/-0.35/-0.31, p < 0.01). There is no proven connection between the Kd value and ASA score in this study (Table 3).

Considering patients with already existing recurrence of meningioma, they had a significantly different K3 and Kd value compared to those patients who underwent surgery for the first time (mean of 70.80/-3.20 vs. 75.37/2.88, respectively, p < 0.01, U test). Ten patients received irradiation and they showed worse results of K3 (70.00 vs. 75.30, p < 0.05, U test) compared to those receiving no radiation therapy, but no significant influence on Kd (p < 0.05, U test).

When the tumor’s location is grouped into convexity and skull base, there is a significant difference in Kd (mean of 4.60 vs. 1.35, p < 0.05, U test). When dividing the patients according to the tumor origin of the meningioma into different groups (group 1: falx/parasagital, group 2: convexity, group 3: skull base, group 4: petrous bone/ tentorial), a significant difference between the four groups (mean of 65.58 (group1) vs. 70.35 (group2) vs. 69.47 (group3) vs. 70.68 (group4)) could be found only in K2 (p < 0.05, Kruskal-Wallis test). No significant difference in K1, K3 and Kd (p > 0.05) of the four categories was shown using the Kruskal-Wallis and Chi-squared test.

**Fig 3:**
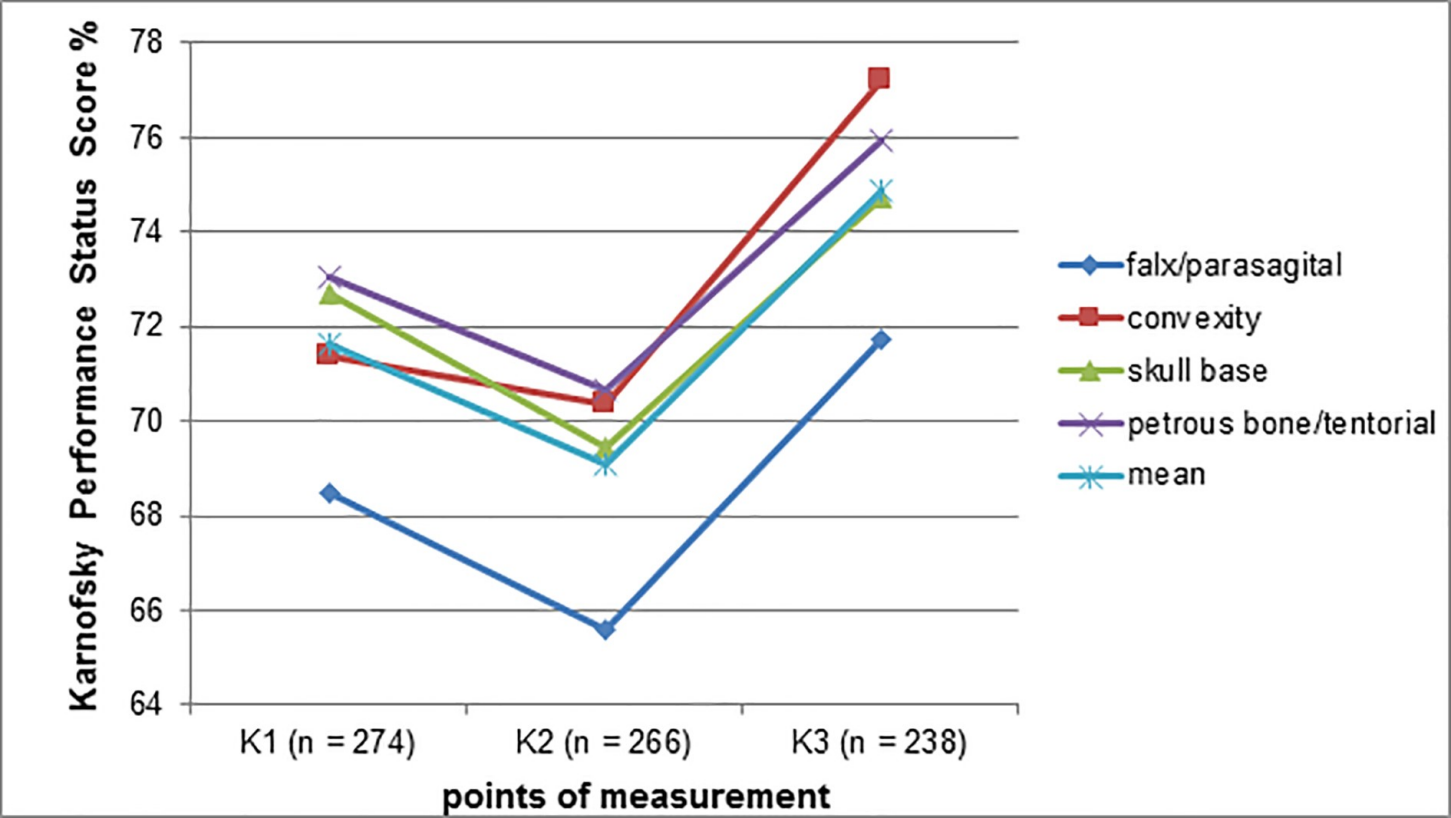
Karnofsky performance score at three points of measurement in relation to tumor origins (falx/parasagital vs. convexity vs. skull base vs. petrous/tentorial vs. average), K1 = preoperative; K2 = 3–6 months postoperative; K3 = 1 year postoperative; Kd = difference between K3 and K1.

Considering the tumor size, 53.7% had less, 40.1% more than 4 cm diameter and 6.2% was unknown. The tumor diameter showed significant differences between K1, 2 and 3 (> 4 cm: mean of 67.03/66.02/72.42 vs. < 4 cm: 74.43/71.86/76.53, p < 0.001/0.001/0.01, U test, respectively), but not in Kd (p > 0.05). There were mass effects in 183 cases (62.20%), divided into two groups (with (a) and without (b) midline shift) which are distributed approximately equally and there were no (c) mass effects in 111 cases. A statistically highly significant difference in Kd (mean of 7.04/0.94/0.00, p < 0.001) between the three categories a–c, respectively, could be found using the Kruskal-Wallis test. Tumor edema was detected in 43.2% of patients by MRI or CT imaging. These patients obtained significantly worse values of K1*** (mean of 67.64 vs. 74.49) and K2** (mean of 66.99 vs. 70.92), but better Kd* scores (mean of 4.38 vs. 0.81) than those without tumor edema (U test) (Fig 3, Table 4)).

**Table 4:** 

Preoperative data		
*Tumor-related*		
Tumor size	*< 4 cm* 158 (53.8%)	*> 4 cm* 118 (40.1%) *unknown* 18 (6.1%)
Location		
1) Hemisphere	*right* 118 (40.1%) *both sides* 59 (20.1%)	*left* 117 (39.8%)
2) Origin	*Falx/Parasagittal* 53 (18.0%)	*Convexity* 59 (20.1%)
	*Sphenoid bone* 63 (21.4%)	*Olfactory* 30 (10.2%)
	*Petrosal bone* 25 (8.5%)	*Others* 64 (21.8%)
Incidental finding	*yes* 90 (30.6%)	*no* 204 (69.4%)
Tumor edema preoperative	*yes* 127 (43.2%)	*no* 167 (56.8%)
Circulatory disturbance of CSF**	*yes* 7 (2.4%)	*no* 287 (97.6%)
Preoperative embolization	*yes* 26 (8.8%)	*no* 268 (91.2%)
Mass effects	*yes* 183 (62.2%)	*no* 111 (37.8%)
1) with midline shift	87 (29.6%)	
2) without midline shift	98 (32.7%)	

Tumor-related, preoperative data of 294 patients with meningioma

** CSF: cerebrospinal fluid

### Karnofsky performance status score

Regarding KPS indices, the mean K1 value was 71.53 (SD 11.95), mean K2 was 69.23 (SD 10.27) and K3 was 74.92 (SD 10.01). The absolute differential amount between mean K3 and K1 was + 3.39, whereas the relative difference between K3 and K1 (Kd), considering only the data of those patients who appeared regularly at follow-up examinations, amounts to 2.28% (SD 11.20). However, a general improvement of the patients’ state of health and quality of life (QOL) could be shown. The increasing K1 value is significantly associated with an increasing K2 (Rho = 0.48, p < 0.01) and K3 (Rho = 0.45, p < 0.01) value. Furthermore, there is an inverse correlation between K1 and Kd (Rho = -0.55, p < 0.01). Therefore, it can be concluded that the higher the preoperative KPS score, the better the postoperative K2 and K3 values (Table 5).

**Table 5:** 

KPS*		
**K1 mean** **K2** **K3** Kd**	71.53 (n = 294) 69.23 (n = 286) 74.92 (n = 254) 2.28 (n = 254)	(SD 11.95) (SD 10.27) (SD 10.01) (SD 11.20)

* Karnofsky Performance Status score of 294 patients with meningioma at three points (K1 = preoperative, K2 = between three and six months postoperative, K3 = about one year postoperative)

** Kd = difference between K1 and K3 (means a change in the patient’s condition)

**Fig 4:**
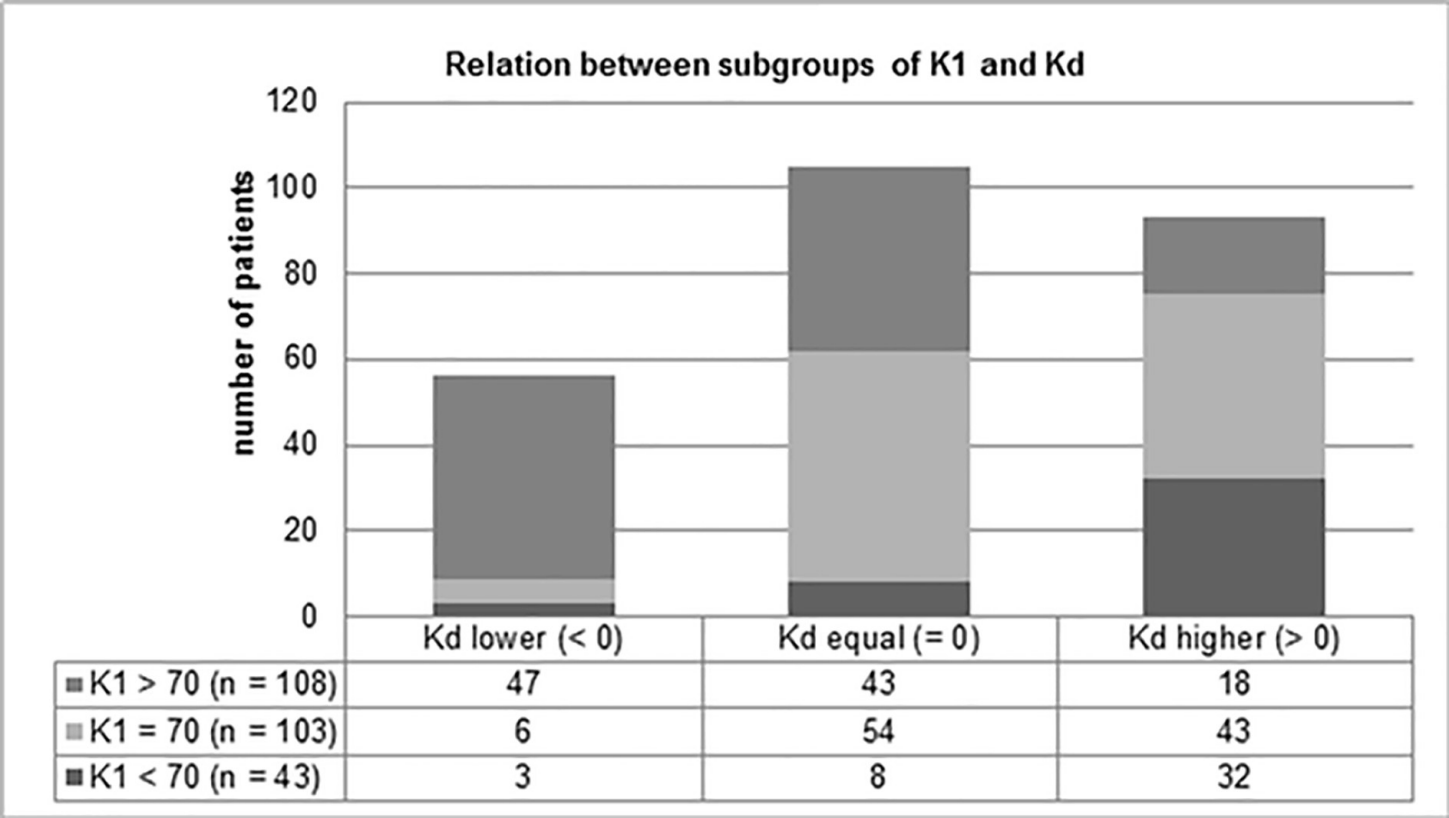
Relation between subgroups of K1 and Kd, K1 = KPS preoperative, KD = difference between K1 and K3, Kd lower means a deterioration in the patient’s condition one year after surgery, Kd equal means no change in the patient’s state. Kd higher means an improvement in the patient’s condition one year after surgery.

Additionally, the influence of preoperative variables was proven when dividing K1 and Kd into the different subgroups: K1 < 70/ = 70/ > 70 and Kd classified into categories lower/equal/higher (Fig 4). There was 20.1% with K1 value of lower than 70, 41.2% with exactly 70 and 38.8% of patients with a KPS score of higher than 70 before surgery. Referring to the Kd subgroups, 22% of patients deteriorated in their health state, 41.3% showed no change in their KPS scores comparing pre- and postoperative condition, and 36.6% obtained a better Kd score, which is associated with an improvement in QOL. Considering the relations between the subgroups of K1 and Kd, significant influence and negative correlation could be confirmed by the Chi-squared test (p < 0.001): Exemplarily, 7% of patients with preoperative KPS score of lower than 70 showed a deterioration in health state 12 months postoperatively (Kd < 0), whereas 43.5% of patients with more than 70 points in K1 obtained a lower Kd, meaning a deterioration in comparison to the preoperative state. Furthermore, 74.4% of patients underwent surgery with preoperative KPS scores less than 70 and only 16.7% of those with a K1 value of more than 70 demonstrated a higher Kd and, consequently, an improvement in QOL.

It was evaluated Significant differences in K1 (between the three categories < 70, = 70 and > 70) were evaluated referring to the following parameters: tumor diameter, mass effects, tumor edema, presence of risk factors, such as arterial hypertension and diabetes and presence of neurological symptoms (p < 0.05, Chi-squared test). A significant influence of the mass effects on Kd of patients with a tumor could also be analyzed (Chi-squared test, p < 0.05) in comparison to the U and Kruskal-Wallis test used. A total of 42.9% showed a better KPS score postoperative than preoperative, whereas 26.5% of the patients without preoperative mass effects had a higher Kd value. Furthermore, significant differences in Kd between meningioma patients presenting neurological symptoms and those without was confirmed (p < 0.001, Chi-squared test). The existence especially of dysfunctional concentration, orientation and memory showed statistically significant differences in Kd divided in three groups, as described above (higher Kd: 56.6% with vs. 34.6% without the disorder named, p < 0.05 Chi-squared). However, there were significant differences in the Kd between the tumor location on one or both hemispheres based on the Chi-square test (p < 0.05) in contrast to the U test. Patients with meningioma located on both hemispheres obtained lower Kd scores more often than those with only one affected hemisphere (Kd lower: 34.7% vs. 19.0%, p < 0.05).

### Binary logistic regression

Three significant predictor variables could be found which are most appropriate for estimating the probability that the difference between K3 and K1 is negative, meaning a postoperative deterioration of the patient’s condition. It can generally be stated that tumor location relating to the skull base, a tumor size greater than 4cm or a change in K1 of one unit (10% steps) is associated with a higher probability of the occurrence of the event Kd < 0. The chart below shows, for example, that the odds of obtaining a negative Kd value is 2.81 times greater for patients with a skull base/falx tumor than those with a convexity tumor. Patients with a tumor diameter larger than 4 cm are 2.64 times more likely to obtain the result Kd < 0 than patients with a tumor size smaller than 4 cm. An increasing K1 value (10% steps) is associated with an increased likelihood, by a factor of 1.20, of exhibiting a negative Kd value (Table 6).

**Table 6:** 

Variables	B**	Standard deviation	Wald***	Sig.****	Exp(B)*****
Skull base/Falx tumor	1.036	0.523	3.919	0.048	2.817
Size > 4cm	0.969	0.425	5.200	0.023	2.635
Preoperative KPS*	0.143	0.026	29.530	0.000	1.154
Constant******	-13.367	2.234	35.818	0.000	0.000

* Karnofsky performance status scale

Binary logistic regression. The presence of the following factors is associated with an increased likelihood of exhibiting a deterioration in the patient’s postoperative state (negative Kd score): skull base/falx tumor (in comparison to convexity tumor), tumor size greater than 4cm or an increasing preoperative KPS value.

** regression coefficient B (means b1 in regression equation)

*** Wald test to verify the significance of each predictor/variable

**** p value

***** Exp(B) value reflects the respective de-logarithmic coefficient (b1) as an odds ratio

****** the constant (b0) in the regression equation

### Factor analysis

A total of nine factors were formed. All variables for factor analysis could not be included for statistical reasons, therefore, the final number of variables used was 36. Those variables which correlated the strongest with the respective factor were assigned to it. Consequently, a set of variables was created which correlates stronger with the factor characterizing it than with the other ones. The highest (I) and second (II) strongest factor loading of each single variable representing the correlation are shown in Table 7. The following factors presented are based on preoperative variables of a patient’s specific situation.

**Table 7:** 

**Factor 1: Patient-related parameters**		
Variables correlating with the factor	Factor loading * I	II (number of factor)
Number of risk factors	+0.688	-
Age at surgery	+0.659	+0.212 (1)
ASA score	+0.654	+0.164 (3)
**Factor 2: Tumor-related parameters**		
Preoperative edema	+0.674	-
Tumor size	+0.630	-
Disorientation/difficulties in concentrating/ memory disorder	+0.518	+0.180 (1)
Preoperative embolization	+0.388	-
**Factor 3: Preoperative symptoms**		
Number of symptoms	+0.771	.0.261 (2)
Sympt. 13: anosmia, hyposmia	+0.585	-0.188 (2)
Sympt.14: other cranial nerve deficits	+0.551	- 0.224 (2)
Sympt. 3: headaches	+0.507	- 0.188 (2)
Incidental finding	- 0.487	- 0.389 (1)
Sympt. 8: visual impairment	+0.389	- 0.275 (4)
**Factor 4: Particularities**		
Already existing recurrence	+0.761	+0.100 (3)
Radiation	+0.721	+0.119 (2)

Factor analysis: Every individual factor consists of different variables, with a strong correlation to the respective one. The figures of factor loading I/II represents the highest correlation and the second highest correlation to the factors, respectively.

* factor loading is a measure of the correlation between the variables and the respective factors: The higher the value (0 to 1/ 0 to -1) of the variables, the stronger the influence on the individual factor.

### IV. Discussion

Patients generally obtained an improvement in their KFS score (Kd) after microsurgical treatment (Kd +2.28%). Similar results were achieved by Meister et al.[14] with a mean Kd value of 4.4%, and by Umansky et al. with a much better Kd value of 21.0%–but with the addition that the collective examined only included 37 cases [15]. This underlines the medical meaningfulness of microsurgical treatment in intracranial meningiomas. Moreover, this study corresponds to Miao et al., who examined the change in the QOL before and after surgery by evaluating the health-related QOL in 2010, which represents a mixture of the KFS and World Health Organization QOL 100 Scale[16]. Their results showed an increase in the health-related QOL score from 73.9 to 84.9, which means that there is a significant improvement in their QOL and, thus, a favorable outcome. Further studies by Kalkanis et al. and other authors support this attitude, while also observing QOL improvement after meningioma surgery [1,17–19]. When regarding the relationship of the Karnofsky indices to each other, binary logistic regression indicated a negative association between K1 and Kd, which was confirmed by Spearman’s correlation. This means that the better the KPS score as a functional surrogate for QOL before operation, the lower the increase in KPS (Kd) after surgery. This might seem contradictory at first glance, but those who are in very poor health have a greater potential of getting better compared to those with only slight restrictions on health and everyday life. The considerable influence of K1 for the patient has been verified by different authors [20,21]. Based on a study determining and assessing prognostic indicators among elderly patients, the KPS on hospital admission affected outcome parameters some years after surgery [6].

Considering the results of similar studies, a change in a patient’s mean age at the time of surgery could be observed over the last decades. While the average age a few decades ago was just over 50 years [20,22], meningioma patients who undergo surgery today are approximately 60 years old, similar to this study [9,21,23,24]. This is not surprising, since the ongoing advances in medicine mean that people are living longer and, consequently, also in the case of patients undergoing surgery. This has allowed the assumption that older patients are associated with a worse outcome because of a higher risk profile. A series of comparative studies confirms the presumed relationship between age and outcome[25–27]. Based on McCarthy et al., survival rates in meningioma were inversely related to age^[^. The association between old age and a worse outcome postoperatively was also significant in a report by Wang et al.[10]. By contrast, the present study could not achieve such clear results. Although there was a significantly worse outcome, meaning KFS 12 months postoperative, in patients older than 65 years of age, the difference between the pre- and postoperative KFS score (Kd) was not significant either by using bivariate tests or by multivariate analysis. Therefore, age cannot be considered as a strong prognostic factor in this study. The factor analysis performed in this study shows a connection between age, the number of risk factors and the ASA score. Considering the inverse correlation between age and Karnofsky indices (K1–K3) demonstrated, it can be concluded that the ASA score and the presence of a high number of risk factors play a role in determining the outcome.

Studies demonstrate that despite a higher prevalence of meningioma among women, with a female to male ratio of 3:2 to 2:1, gender is not regarded as a risk factor for survival[28–31]. In line with this, the study revealed no statistically proven impact on prognosis. On the other hand, Duntze et al. identify the female gender as a negative prognostic factor, but there were only 36 cases examined in their retrospective study in 2012 [32]. In turn, female gender gained a significantly better KFS score five years after surgical treatment in a 2010 report of 250 patients aged over 65 years. Even women’s morbidity and mortality were also substantially lower[6]. Further studies are needed to ascertain the impact of gender on prognosis because of the inconsistent findings.

Study results are contradictory regarding the tumor location with a focus on attachment as a possible prognostic factor. Based on the Pechlivanis et al. report in 2011, Konglund et al. in 2013 and some others, outcome was not associated with tumor origin[8,18,27], whereas Saraf et al. and Kotecha et al. report a less favorable outcome in meningiomas located at the skull base in comparison to brain convexity neoplasm[25,33]. These findings are supported by Zaher et al., although they only include atypical meningiomas[34]. One possible reason for the worse outcome might be the less accessible location and nearby neural or vascular structures which complicate complete tumor resection[7]. This study could substantiate by means of bivariate analysis and binary regression that skull base tumors are more associated with a poorer outcome (Kd) than menigiomas located in the cerebral convexities. In conclusion, location can be seen as a further relevant prognostic factor. However, our study showed no significant relevance of outcome improvement when subdividing (four groups in our study) meningiomas according to tumor origin.

Another important factor determining prognosis is tumor size. This attitude is supported by studies from Miao et al. and Wu et al.[16,35]. Dobran et al. also propose that the tumor size is an important prognostic factor in meningioma outcome[36]. However, it should be noted that the patients included were over 80 years old. According to multivariate analysis, tumor diameter was found to be a significantly poor prognostic factor. More exactly, patients with a meningioma diameter of more than 4 cm tend more to a worsening of their general condition (Kd < 0) compared to those with a tumor size lower than 4cm, meaning a more unfavorable outcome. These results could be confirmed by uni- and bivariate tests, but only regarding the postoperative KFS scores (K2, K3), thus, Kd showed no significant difference between tumor size greater and smaller than 4 cm.

However, only a poor significant connection between preexisting recurrence and Simpson resection grades of 3–5 could be found regarding those patients with a preexisting recurrence of meningioma in this study. Therefore, patients with an already existing recurrence of meningioma are more highly connected with tumors of a resection grade of 3–5 in percentage terms than clients without previous recurrence before the evaluation period (32.3 vs. 15.6%). Additionally, in percentage terms, patients with preexisting recurrence show a greater tendency to WHO II–III tumors than the comparison group (41.9%, 14.4%, respectively). Although patients with preexisting recurrence presented significantly worse K3 and Kd scores in bivariate analysis, that aspect was not found to influence the KFS deterioration in the multivariate analysis. Nevertheless, the findings above support the assertion that the fact of preexisting recurrence has an impact on the outcome and, thus, it could be seen as a weak prognostic factor. There is too little data in the literature considering this special aspect to examine its influence on a patient’s outcome. Only one author described in his paper that about 385 meningioma patients experienced a significant decrease in their KFS scores six months after surgery, but he did not find a difference in Kd among recurrent patients or those who suffer from meningioma for the first time[14]. As another interesting point, our factor analysis could demonstrate a correlation between radiation therapy and preexisting recurrence, therefore, they might be linked.

Markovic et al. investigated the influence of peritumoral edema on the prognosis of morbidity and postoperative complications referring to 78 patients with supratentorial meningiomas treated surgically[37]. They determined that the outcome was significantly better in cases without than in patients with peritumoral edema, but there is no clear consensus in the literature [1,27]. This study, however, identified a connection between the occurrence of peritumoral edema or mass effects and an improved Kd score.

A similar report in 2010 demonstrated the impact of the presence of preoperative dysfunctions on improved outcomes, which is in accordance with our findings. Patients presenting with aphasia, motor deficits, confusion and changes in behavior have shown a clear improvement of symptoms, on the one hand, and a more favorable KPS after surgical intervention, on the other hand[6]. Even this matches the present study. Further studies, such as Awad et al. and Gijtenbeek et al., have confirmed the influence of neurological symptoms on postoperative outcomes[18,38]. They evinced that patients with neurological symptoms are associated with significantly worse outcomes, at first. However, when considering the increase of Kd, a much better result in patients with preoperative disorders is clearly demonstrated[14]. Even the total number of all recorded symptoms showed a negative correlation with K1–2, on the one hand, though a positive correlation with the other. Although we could not confirm the significance of presenting symptoms generally by means of binary logistic regression, uni- and bivariate analysis indicated that the presence of preoperative neurological symptoms might be a possible candidate for a prognostic factor in meningioma patients treated microsurgically.

Nevertheless, there are some limitations in this study which underlie the retrospective analysis of patient data. Based on available patient data via SAP, the study could only generate incomplete records for statistical analysis. After successful surgical intervention, not all patients attended the follow-up visits, therefore, the data of K2 and K3 values could not be collected completely (eight patients missed the checkup between three and six months postoperative and 40 patients one year after intervention). Consequently, this resulted in a discrepancy between the absolute and relative Kd score. The absolute differential amount between mean K3 and K1 is + 3.39, whereas the Kd value recorded an increase of 2.28% considering only data of those patients who appeared regularly at follow-up examinations (3–6 months, one year after surgical removal of meningioma). Another limitation is the follow-up regarded for one year only.

### V. Conclusion

In summary, our retrospective cohort analysis determines different prognostic factors based on preoperative variables in patients suffering from microsurgical meningioma treatment. Specific predictors for postoperative worsening of outcome in descending order of importance could be found by using multivariate analysis: skull base meningioma, tumor size larger than 4 cm and high preoperative KPS score. In addition, further negative prognostic factors were designated based on uni- and bivariate analysis: the presence and the number of symptoms, the occurrence of hemiplegic symptoms, motor deficits or impaired memory, concentration and orientation, mass effects by displacement or peritumoral edema, meningioma located on both hemispheres, incidental findings and preexisting recurrence. However, patients age did not influence the neurological outcome negatively. This specific information may help in the decision-making process for microsurgical treatment of cranial meningiomas and balance operative treatment versus additional treatment options, such as irradiation, which is already the theme of many studies[5,7,8].

## Supporting information

S1 Dataset M.K.1.sav. (PDF)
