# Supplementary material for: Prognostic factors of microsurgical treatment of intracranial meningiomas – A multivariate analysis
Source: PLoS One. 2018 Oct 16;13(10):e0202520. doi: 10.1371/journal.pone.0202520 (PMC6191082; doi:10.1371/journal.pone.0202520)
Supplement: S1 Dataset — (PDF) [file pone.0202520.s001.pdf]

	Number	Sex	OP.year	Age	Histology.WHO	Type.histology
1	1,00	1,00	2014,00	49,00	1,00	3,00
2	2,00	,00	2014,00	82,00	2,00	12,00
3	3,00	1,00	2014,00	75,00	1,00	1,00
4	4,00	1,00	2014,00	43,00	1,00	1,00
5	5,00	1,00	2014,00	74,00	1,00	3,00
6	6,00	,00	2014,00	49,00	1,00	3,00
7	7,00	1,00	2014,00	71,00	1,00	3,00
8	8,00	1,00	2010,00	73,00	2,00	12,00
9	9,00	1,00	2014,00	46,00	1,00	1,00
10	10,00	,00	2014,00	41,00	1,00	2,00
11	11,00	1,00	2014,00	76,00	1,00	3,00
12	12,00	1,00	2013,00	75,00	1,00	3,00
13	13,00	1,00	2014,00	58,00	1,00	1,00
14	14,00	,00	2014,00	32,00	1,00	1,00
15	15,00	,00	2014,00	59,00	1,00	3,00
16	16,00	,00	2013,00	36,00	1,00	1,00
17	17,00	1,00	2014,00	49,00	1,00	1,00
18	18,00	1,00	2014,00	47,00	1,00	1,00
19	19,00	1,00	2014,00	85,00	1,00	1,00
20	20,00	1,00	2013,00	60,00	1,00	3,00
21	21,00	,00	2014,00	72,00	1,00	1,00
22	22,00	1,00	2014,00	67,00	1,00	3,00
23	23,00	,00	2014,00	32,00	2,00	5,00
24	24,00	1,00	2014,00	67,00	1,00	1,00
25	25,00	1,00	2014,00	78,00	1,00	3,00
26	26,00	1,00	2014,00	51,00	1,00	1,00
27	27,00	1,00	2014,00	63,00	1,00	3,00
28	28,00	1,00	2014,00	59,00	1,00	,00
29	29,00	,00	2014,00	44,00	1,00	1,00
30	30,00	1,00	2014,00	69,00	1,00	3,00
31	31,00	1,00	2014,00	64,00	1,00	7,00
32	32,00	1,00	2014,00	78,00	1,00	3,00
33	33,00	1,00	2014,00	54,00	1,00	1,00
34	34,00	1,00	2014,00	69,00	1,00	3,00
35	35,00	1,00	2014,00	82,00	1,00	3,00
36	36,00	,00	2014,00	79,00	1,00	3,00
37	37,00	1,00	2014,00	37,00	1,00	1,00

	Side	Localisation	Duration.of.symptoms	No.symptoms	First.symptom	Haedache
1	2,00	9,00	62,00	,00	1,00	1,00
2	3,00	2,00	8,00	,00	17,00	,00
3	3,00	1,00	,00	,00	21,00	,00
4	2,00	11,00	,00	1,00	,00	,00
5	2,00	1,00	4,00	,00	17,00	,00
6	1,00	1,00	,00	,00	,00	,00
7	1,00	11,00	,00	,00	22,00	,00
8	3,00	13,00	,00	1,00	,00	,00
9	1,00	1,00	6,00	,00	17,00	1,00
10	1,00	11,00	4,00	1,00	,00	,00
11	2,00	4,00	12,00	,00	6,00	,00
12	2,00	2,00	,00	1,00	17,00	,00
13	2,00	2,00	,00	,00	4,00	,00
14	1,00	12,00	6,00	,00	21,00	,00
15	1,00	5,00	33,00	,00	17,00	,00
16	1,00	2,00	12,00	,00	14,00	,00
17	2,00	1,00	,00	1,00	,00	,00
18	3,00	5,00	,00	,00	6,00	,00
19	3,00	10,00	,00	,00	14,00	,00
20	3,00	5,00	1,00	,00	14,00	,00
21	1,00	3,00	24,00	,00	7,00	,00
22	2,00	3,00	4,00	,00	1,00	1,00
23	1,00	13,00	1,00	,00	7,00	1,00
24	1,00	11,00	48,00	,00	12,00	,00
25	3,00	1,00	,00	1,00	,00	,00
26	2,00	3,00	,00	1,00	1,00	,00
27	1,00	3,00	60,00	,00	1,00	1,00
28	2,00	2,00	,00	,00	16,00	,00
29	1,00	2,00	,00	,00	1,00	1,00
30	3,00	5,00	,00	1,00	,00	,00
31	2,00	7,00	,00	1,00	12,00	,00
32	1,00	1,00	,00	,00	15,00	1,00
33	1,00	8,00	4,00	,00	10,00	,00
34	2,00	3,00	,00	,00	15,00	,00
35	1,00	11,00	,00	,00	15,00	1,00
36	2,00	3,00	,00	,00	16,00	,00
37	1,00	11,00	,00	,00	15,00	,00

	Nausea	Emesis	Nausea_Emesis	Seizures	Oculo.paresis	Viszual.paresis
1	,00	,00	,00	,00	,00	,00
2	,00	,00	,00	,00	,00	,00
3	,00	,00	,00	,00	,00	,00
4	,00	,00	,00	,00	,00	,00
5	,00	,00	,00	,00	,00	,00
6	,00	,00	,00	,00	,00	,00
7	1,00	1,00	1,00	,00	,00	,00
8	,00	,00	,00	,00	,00	,00
9	,00	,00	,00	,00	,00	1,00
10	,00	,00	,00	,00	,00	,00
11	,00	,00	,00	,00	,00	1,00
12	,00	,00	,00	,00	,00	,00
13	,00	,00	,00	1,00	,00	,00
14	,00	,00	,00	,00	,00	,00
15	,00	,00	,00	,00	,00	,00
16	,00	,00	,00	1,00	,00	,00
17	,00	,00	,00	,00	,00	,00
18	,00	,00	,00	,00	,00	1,00
19	,00	,00	,00	,00	,00	,00
20	,00	,00	,00	,00	,00	,00
21	,00	,00	,00	,00	,00	,00
22	,00	,00	,00	,00	,00	,00
23	,00	,00	,00	,00	,00	,00
24	,00	,00	,00	,00	,00	,00
25	,00	,00	,00	,00	,00	,00
26	,00	,00	,00	,00	,00	,00
27	,00	,00	,00	,00	,00	,00
28	,00	,00	,00	,00	,00	,00
29	,00	,00	,00	,00	,00	,00
30	,00	,00	,00	,00	,00	,00
31	,00	,00	,00	,00	,00	,00
32	,00	,00	,00	,00	,00	,00
33	,00	,00	,00	,00	,00	,00
34	,00	,00	,00	1,00	,00	,00
35	,00	,00	,00	,00	,00	,00
36	,00	,00	,00	,00	,00	,00
37	,00	,00	,00	,00	,00	,00

	Viszual.deficite	Papilloedema	Optic.atrophie	Exophthalmus	Kakosmia	Other.nerve.paresis
1	1,00	,00	?	?	?	?
2	,00	,00	?	?	?	?
3	,00	,00	?	?	?	?
4	,00	,00	?	?	?	?
5	,00	,00	?	?	?	?
6	,00	,00	?	?	?	?
7	,00	,00	?	?	?	?
8	,00	,00	?	?	?	?
9	,00	,00	?	?	?	?
10	,00	,00	?	?	?	?
11	,00	,00	?	?	?	?
12	,00	,00	?	?	?	?
13	,00	,00	?	?	?	?
14	,00	,00	?	?	?	?
15	,00	,00	?	?	?	?
16	,00	,00	?	?	?	?
17	,00	,00	?	?	?	?
18	1,00	,00	?	?	?	?
19	,00	,00	?	?	?	?
20	1,00	,00	?	?	?	?
21	1,00	,00	?	?	?	?
22	,00	,00	?	?	?	?
23	1,00	,00	?	?	?	?
24	,00	,00	?	?	?	?
25	,00	,00	?	?	?	?
26	1,00	,00	?	?	?	?
27	,00	,00	?	?	?	?
28	,00	,00	?	?	?	?
29	,00	,00	?	?	?	?
30	,00	,00	?	?	?	?
31	,00	,00	?	?	?	?
32	,00	,00	?	?	?	?
33	,00	,00	?	?	?	?
34	,00	,00	?	?	?	?
35	,00	,00	?	?	?	?
36	,00	,00	?	?	?	?
37	,00	,00	?	?	?	?

	Sensibility.disorder	Motoric.disorder	Cerebellar.symptoms	Aphasia	Concentration.disoreders	Personallity.change
1	,00	,0	,00	,00	1,00	,00
2	,00	,0	1,00	,00	1,00	1,00
3	,00	,0	,00	,00	,00	,00
4	,00	,0	1,00	,00	,00	,00
5	,00	1,0	,00	1,00	1,00	,00
6	,00	,0	,00	,00	,00	,00
7	,00	,0	,00	,00	,00	,00
8	,00	,0	,00	,00	,00	,00
9	,00	,0	,00	,00	,00	,00
10	,00	,0	1,00	,00	,00	,00
11	,00	,0	,00	,00	,00	,00
12	,00	1,0	,00	,00	1,00	1,00
13	,00	,0	,00	,00	1,00	,00
14	,00	,0	,00	,00	,00	,00
15	,00	,0	,00	,00	1,00	,00
16	,00	1,0	,00	,00	,00	,00
17	,00	,0	,00	,00	,00	,00
18	,00	,0	,00	,00	,00	,00
19	,00	1,0	,00	,00	,00	,00
20	,00	1,0	,00	,00	,00	1,00
21	,00	,0	,00	,00	,00	,00
22	,00	,0	,00	,00	,00	,00
23	,00	,0	1,00	,00	,00	,00
24	,00	,0	,00	,00	,00	,00
25	1,00	1,0	,00	,00	,00	,00
26	,00	,0	,00	,00	,00	,00
27	,00	,0	,00	,00	,00	,00
28	,00	1,0	,00	1,00	,00	,00
29	,00	,0	,00	,00	,00	,00
30	,00	,0	,00	,00	1,00	,00
31	,00	,0	,00	,00	,00	,00
32	,00	,0	1,00	,00	,00	,00
33	,00	,0	,00	,00	,00	,00
34	,00	,0	1,00	,00	,00	,00
35	,00	,0	1,00	,00	,00	,00
36	,00	,0	,00	1,00	,00	,00
37	,00	,0	1,00	,00	,00	,00

	Other.motoric .disorder	loss.counsciu oness	Double.vision	Histological.c lear	Histological.u nclear	Size
1	,00	,00	1,00	1,00	,00	2,00
2	1,00	,00	,00	1,00	,00	,00
3	1,00	,00	,00	1,00	,00	1,00
4	,00	,00	,00	1,00	,00	,00
5	,00	,00	,00	1,00	,00	2,00
6	,00	,00	,00	2,00	,00	1,00
7	,00	,00	,00	1,00	,00	1,00
8	,00	,00	,00	1,00	,00	2,00
9	,00	,00	1,00	1,00	,00	1,00
10	,00	,00	,00	1,00	,00	1,00
11	,00	,00	,00	1,00	,00	1,00
12	,00	,00	,00	1,00	,00	,00
13	,00	,00	,00	1,00	,00	1,00
14	,00	,00	1,00	1,00	,00	2,00
15	,00	,00	,00	1,00	,00	1,00
16	,00	,00	,00	1,00	,00	1,00
17	,00	,00	,00	1,00	,00	1,00
18	,00	,00	,00	1,00	,00	,00
19	,00	1,00	,00	1,00	,00	2,00
20	,00	,00	,00	1,00	,00	2,00
21	1,00	,00	,00	1,00	,00	2,00
22	,00	,00	,00	2,00	,00	1,00
23	,00	,00	,00	1,00	,00	2,00
24	,00	,00	,00	1,00	,00	1,00
25	,00	,00	,00	1,00	,00	2,00
26	,00	,00	,00	1,00	,00	1,00
27	,00	,00	,00	1,00	,00	2,00
28	,00	,00	,00	1,00	1,00	2,00
29	,00	,00	,00	1,00	,00	,00
30	,00	1,00	,00	1,00	,00	2,00
31	,00	,00	,00	1,00	,00	1,00
32	,00	,00	,00	1,00	1,00	2,00
33	,00	,00	,00	1,00	1,00	1,00
34	,00	,00	,00	1,00	,00	,00
35	,00	,00	,00	1,00	,00	1,00
36	,00	,00	,00	1,00	,00	,00
37	,00	,00	,00	1,00	,00	1,00

	MRi.CCT	Form	CSF	Edema	Masseffect	Embolisation
1	2,00	1,00	,00	1,00	?	?
2	1,00	1,00	,00	1,00	?	?
3	2,00	.	,00	1,00	?	?
4	2,00	2,00	,00	,00	?	?
5	2,00	.	,00	,00	?	?
6	2,00	2,00	,00	,00	?	?
7	2,00	.	,00	1,00	?	?
8	2,00	2,00	,00	,00	?	?
9	2,00	2,00	,00	1,00	?	?
10	2,00	.	,00	,00	?	?
11	2,00	1,00	,00	,00	?	?
12	2,00	1,00	,00	1,00	?	?
13	2,00	1,00	,00	1,00	?	?
14	2,00	.	,00	1,00	?	?
15	2,00	.	,00	1,00	?	?
16	2,00	.	,00	1,00	?	?
17	2,00	2,00	,00	,00	?	?
18	2,00	.	,00	,00	?	?
19	2,00	1,00	,00	1,00	?	?
20	2,00	.	,00	,00	?	?
21	2,00	.	,00	,00	?	?
22	2,00	.	,00	1,00	?	?
23	2,00	2,00	,00	1,00	?	?
24	2,00	.	,00	,00	?	?
25	1,00	.	,00	1,00	?	?
26	2,00	1,00	,00	,00	?	?
27	2,00	.	,00	,00	?	?
28	2,00	.	,00	1,00	?	?
29	2,00	.	,00	,00	?	?
30	2,00	1,00	,00	,00	?	?
31	2,00	2,00	,00	,00	?	?
32	2,00	1,00	,00	1,00	?	?
33	2,00	2,00	,00	,00	?	?
34	2,00	.	,00	,00	?	?
35	2,00	.	,00	,00	?	?
36	2,00	.	,00	,00	?	?
37	2,00	.	,00	1,00	?	?

	Hypertonia	Adipositas	Heart.disorder	Lung.disorder	Liver.disorder	Kindey.disorder
1	?	?	?	?	?	?
2	?	?	?	?	?	?
3	?	?	?	?	?	?
4	?	?	?	?	?	?
5	?	?	?	?	?	?
6	?	?	?	?	?	?
7	?	?	?	?	?	?
8	?	?	?	?	?	?
9	?	?	?	?	?	?
10	?	?	?	?	?	?
11	?	?	?	?	?	?
12	?	?	?	?	?	?
13	?	?	?	?	?	?
14	?	?	?	?	?	?
15	?	?	?	?	?	?
16	?	?	?	?	?	?
17	?	?	?	?	?	?
18	?	?	?	?	?	?
19	?	?	?	?	?	?
20	?	?	?	?	1,00	,00
21	,00	,00	,00	,00	,00	,00
22	,00	,00	,00	,00	,00	,00
23	1,00	1,00	,00	1,00	,00	,00
24	1,00	,00	,00	,00	,00	,00
25	1,00	,00	1,00	,00	,00	,00
26	1,00	,00	,00	,00	,00	,00
27	1,00	,00	,00	,00	,00	,00
28	,00	,00	,00	,00	,00	,00
29	,00	,00	,00	,00	,00	,00
30	,00	,00	,00	,00	,00	1,00
31	1,00	,00	,00	,00	,00	,00
32	1,00	1,00	1,00	1,00	,00	,00
33	,00	,00	,00	,00	,00	,00
34	1,00	,00	,00	,00	,00	,00
35	,00	,00	,00	,00	,00	,00
36	1,00	,00	1,00	1,00	,00	,00
37	,00	,00	,00	,00	,00	,00

	Diabetes	Varicosis	ASA	Simpson.grade	OP.duration	intraOP.brain swelling
1	,00	,00	3,00	1,00	506,00	1,00
2	,00	,00	3,00	4,00	588,00	,00
3	1,00	,00	3,00	2,00	333,00	,00
4	,00	,00	1,00	1,00	264,00	,00
5	1,00	,00	4,00	1,00	552,00	,00
6	,00	,00	1,00	1,00	289,00	,00
7	1,00	,00	2,00	1,00	321,00	,00
8	1,00	,00	3,00	3,00	593,00	,00
9	,00	1,00	3,00	1,00	231,00	,00
10	,00	,00	3,00	1,00	246,00	,00
11	,00	,00	3,00	1,00	190,00	,00
12	1,00	,00	2,00	1,00	202,00	,00
13	,00	,00	3,00	2,00	239,00	,00
14	,00	,00	2,00	4,00	417,00	,00
15	,00	,00	2,00	1,00	188,00	,00
16	,00	,00	2,00	2,00	300,00	,00
17	,00	,00	1,00	1,00	180,00	,00
18	,00	,00	3,00	1,00	355,00	,00
19	,00	,00	3,00	1,00	269,00	,00
20	,00	,00	3,00	4,00	626,00	1,00
21	,00	,00	2,00	4,00	591,00	,00
22	,00	,00	2,00	1,00	331,00	,00
23	,00	,00	2,00	2,00	310,00	,00
24	,00	,00	2,00	2,00	127,00	,00
25	,00	,00	3,00	1,00	552,00	,00
26	,00	,00	2,00	4,00	211,00	,00
27	,00	,00	2,00	4,00	289,00	,00
28	,00	,00	3,00	1,00	321,00	,00
29	,00	,00	2,00	1,00	201,00	,00
30	,00	,00	3,00	1,00	379,00	1,00
31	,00	,00	2,00	4,00	424,00	,00
32	,00	,00	2,00	1,00	186,00	,00
33	,00	,00	2,00	1,00	435,00	,00
34	,00	,00	2,00	2,00	279,00	,00
35	,00	,00	3,00	1,00	124,00	,00
36	,00	,00	3,00	1,00	121,00	,00
37	,00	,00	2,00	1,00	301,00	,00

	Transfusion	Use.CUSA	Craniotomy	Sinus.lesion	Bleeding	Dura.closure
1	,00	1,00	1,00	,00	,00	3,00
2	,00	1,00	1,00	,00	,00	1,00
3	,00	,00	1,00	,00	,00	3,00
4	,00	1,00	1,00	,00	,00	3,00
5	,00	1,00	1,00	,00	,00	1,00
6	,00	,00	2,00	1,00	,00	3,00
7	,00	1,00	1,00	,00	,00	1,00
8	3,00	,00	2,00	1,00	1,00	2,00
9	,00	,00	1,00	,00	,00	3,00
10	,00	1,00	1,00	,00	,00	1,00
11	,00	,00	1,00	,00	,00	2,00
12	,00	1,00	2,00	,00	,00	3,00
13	,00	,00	1,00	1,00	,00	1,00
14	,00	1,00	1,00	,00	,00	3,00
15	,00	,00	1,00	,00	,00	1,00
16	,00	1,00	1,00	,00	,00	1,00
17	,00	1,00	1,00	,00	,00	2,00
18	,00	1,00	1,00	,00	,00	2,00
19	,00	1,00	1,00	,00	,00	2,00
20	,00	1,00	1,00	,00	,00	2,00
21	,00	1,00	1,00	,00	1,00	3,00
22	,00	1,00	1,00	,00	,00	2,00
23	,00	1,00	1,00	1,00	1,00	1,00
24	,00	,00	1,00	,00	,00	3,00
25	,00	1,00	1,00	,00	,00	3,00
26	,00	,00	1,00	,00	,00	3,00
27	,00	1,00	1,00	,00	,00	3,00
28	,00	1,00	1,00	,00	,00	2,00
29	,00	,00	1,00	,00	,00	2,00
30	,00	1,00	1,00	,00	,00	2,00
31	,00	1,00	1,00	,00	,00	3,00
32	,00	,00	1,00	,00	,00	3,00
33	,00	1,00	2,00	,00	,00	2,00
34	,00	,00	1,00	,00	,00	3,00
35	,00	1,00	1,00	,00	,00	3,00
36	,00	,00	1,00	,00	,00	3,00
37	,00	1,00	1,00	,00	,00	3,00

	Tachosil.Fibrin	Transfusion.postOP	Seizure.therapy	Antibiotics.postOP	CSF.circulatory.disorder	Edema.postOP
1	1,00	,00	1,00	,00	,00	1,00
2	1,00	4,00	,00	,00	,00	,00
3	1,00	,00	,00	,00	,00	1,00
4	1,00	,00	,00	,00	,00	,00
5	1,00	,00	,00	,00	,00	1,00
6	1,00	,00	,00	,00	,00	,00
7	1,00	,00	,00	,00	,00	,00
8	1,00	,00	,00	1,00	,00	,00
9	1,00	,00	,00	,00	,00	,00
10	1,00	,00	,00	,00	,00	,00
11	1,00	,00	,00	,00	,00	,00
12	1,00	,00	,00	1,00	,00	,00
13	1,00	,00	2,00	,00	,00	,00
14	1,00	,00	,00	,00	,00	,00
15	1,00	,00	,00	,00	,00	,00
16	1,00	,00	2,00	,00	,00	,00
17	1,00	,00	,00	,00	,00	,00
18	1,00	,00	,00	1,00	,00	1,00
19	1,00	,00	,00	,00	,00	,00
20	1,00	,00	,00	,00	,00	1,00
21	1,00	,00	,00	,00	,00	,00
22	1,00	,00	,00	,00	,00	,00
23	1,00	,00	,00	,00	,00	,00
24	1,00	,00	,00	,00	,00	1,00
25	1,00	,00	2,00	,00	,00	,00
26	1,00	,00	,00	,00	,00	,00
27	1,00	,00	,00	,00	,00	,00
28	1,00	,00	,00	,00	,00	1,00
29	1,00	,00	,00	,00	,00	,00
30	1,00	,00	,00	,00	,00	,00
31	1,00	,00	,00	,00	,00	,00
32	1,00	,00	,00	,00	,00	1,00
33	1,00	,00	,00	,00	,00	,00
34	1,00	,00	,00	,00	,00	,00
35	1,00	,00	,00	1,00	,00	,00
36	1,00	,00	,00	,00	,00	,00
37	1,00	,00	,00	,00	,00	,00

	Kind.of.bleeding	Infection	Revision1	Revision2	Revision3	Seizures.postOP
1	,00	,00	,00	,00	?	?
2	,00	,00	,00	,00	?	?
3	,00	,00	,00	,00	?	?
4	,00	,00	,00	,00	?	?
5	,00	,00	,00	,00	?	?
6	,00	,00	,00	,00	?	?
7	,00	1,00	2,00	,00	?	?
8	,00	1,00	2,00	4,00	?	?
9	,00	,00	,00	,00	?	?
10	,00	,00	,00	,00	?	?
11	,00	,00	,00	,00	?	?
12	,00	1,00	2,00	,00	?	?
13	,00	1,00	,00	,00	?	?
14	,00	1,00	2,00	,00	?	?
15	,00	,00	,00	,00	?	?
16	,00	,00	,00	,00	?	?
17	,00	,00	,00	,00	?	?
18	,00	1,00	2,00	2,00	?	?
19	1,00	,00	,00	,00	?	?
20	,00	,00	1,00	2,00	?	?
21	,00	,00	,00	,00	,00	,00
22	,00	,00	,00	,00	,00	,00
23	,00	,00	,00	,00	,00	,00
24	,00	,00	,00	,00	,00	,00
25	,00	,00	,00	,00	,00	1,00
26	,00	,00	,00	,00	,00	,00
27	3,00	,00	,00	,00	,00	,00
28	,00	,00	,00	,00	,00	,00
29	,00	,00	,00	,00	,00	1,00
30	,00	,00	,00	,00	,00	,00
31	,00	,00	,00	,00	,00	,00
32	,00	,00	,00	,00	,00	,00
33	,00	,00	,00	,00	,00	,00
34	,00	,00	,00	,00	,00	,00
35	,00	1,00	2,00	,00	,00	,00
36	,00	,00	,00	,00	,00	,00
37	,00	,00	,00	,00	,00	,00

	Thro.Emb	D.insidipus	Dys.Aphasia	Sens.Hemi	Motor.Hemi	Other.symptoms
1	?	?	?	?	?	?
2	?	?	?	?	?	?
3	?	?	?	?	?	?
4	?	?	?	?	?	?
5	?	?	?	?	?	?
6	?	?	?	?	?	?
7	?	?	?	?	?	?
8	?	?	?	?	?	?
9	?	?	?	?	?	?
10	?	?	?	?	?	?
11	?	?	?	?	?	?
12	?	?	?	?	?	?
13	?	?	?	?	?	?
14	?	?	?	?	?	?
15	?	?	?	?	?	?
16	?	?	?	?	?	?
17	?	?	?	?	?	?
18	?	?	?	?	?	?
19	?	?	?	?	?	?
20	?	?	?	?	?	?
21	1,00	,00	,00	,00	,00	,00
22	,00	,00	,00	,00	,00	,00
23	,00	,00	,00	,00	,00	,00
24	,00	,00	,00	,00	,00	1,00
25	,00	,00	,00	,00	1,00	,00
26	,00	,00	,00	,00	,00	,00
27	,00	,00	,00	,00	,00	,00
28	,00	,00	,00	,00	,00	,00
29	,00	,00	1,00	,00	,00	,00
30	,00	,00	,00	,00	,00	1,00
31	,00	,00	,00	,00	,00	1,00
32	,00	,00	,00	,00	,00	,00
33	,00	,00	,00	,00	,00	,00
34	,00	,00	,00	,00	,00	1,00
35	,00	,00	,00	,00	,00	1,00
36	,00	,00	,00	,00	,00	,00
37	,00	,00	,00	,00	,00	,00

	ICU.stay	NCH.stay	Total.duratior	Rehabilitation	Radiation	Recurrence
1	?	?	?	?	?	?
2	?	?	?	?	?	?
3	?	?	?	?	?	?
4	?	?	?	?	?	?
5	?	?	?	?	?	?
6	?	?	?	?	?	?
7	?	?	?	?	?	?
8	?	?	?	?	?	?
9	?	?	?	?	?	?
10	?	?	?	?	?	?
11	?	?	?	?	?	?
12	?	?	?	?	?	?
13	?	?	?	?	?	?
14	?	?	?	?	?	?
15	?	?	?	?	?	?
16	?	?	?	?	?	?
17	?	?	?	?	?	?
18	?	?	?	?	?	?
19	?	?	?	?	?	?
20	?	?	?	?	?	?
21	5,00	10,00	?	?	?	?
22	1,00	7,00	?	?	?	?
23	1,00	6,00	?	?	?	?
24	1,00	9,00	?	?	?	?
25	1,00	11,00	?	?	?	?
26	1,00	6,00	?	?	?	?
27	2,00	5,00	?	?	?	?
28	1,00	8,00	?	?	?	?
29	1,00	5,00	?	?	?	?
30	6,00	13,00	?	?	?	?
31	1,00	12,00	?	?	?	?
32	1,00	12,00	?	?	?	?
33	1,00	11,00	?	?	?	?
34	1,00	5,00	?	?	?	?
35	1,00	11,00	?	?	?	?
36	3,00	7,00	?	?	?	?
37	1,00	6,00	?	?	?	?

	Recurrence1	Daeth	Karnofsky.score.pre	Karnofsky.score.post1.3.month	Karnofsky.score.post6.12.month	Difference.K3.K1
1	?	?	?	?	?	?
2	?	?	?	?	?	?
3	?	?	?	?	?	?
4	?	?	?	?	?	?
5	?	?	?	?	?	?
6	?	?	?	?	?	?
7	?	?	?	?	?	?
8	?	?	?	?	?	?
9	?	?	?	?	?	?
10	?	?	?	?	?	?
11	?	?	?	?	?	?
12	?	?	?	?	?	?
13	?	?	?	?	?	?
14	?	?	?	?	?	?
15	?	?	?	?	?	?
16	?	?	?	?	?	?
17	?	?	?	?	?	?
18	?	?	?	?	?	?
19	?	?	?	?	?	?
20	?	?	?	?	?	?
21	?	?	?	?	?	?
22	?	?	?	?	?	?
23	?	?	?	?	?	?
24	?	?	?	?	?	?
25	?	?	?	?	?	?
26	?	?	?	?	?	?
27	?	?	?	?	?	?
28	?	?	?	?	?	?
29	?	?	?	?	?	?
30	?	?	?	?	?	?
31	?	?	?	?	?	?
32	?	?	?	?	?	?
33	?	?	?	?	?	?
34	?	?	?	?	?	?
35	?	?	?	?	?	?
36	?	?	?	?	?	?
37	?	?	?	?	?	?

	Diffenrence.K3.K2	K2K1	Agegroup1	Agegroup2
1	.	,00	4,00	3,00
2	.	10,00	8,00	5,00
3	10,00	20,00	7,00	4,00
4	,00	-10,00	4,00	3,00
5	10,00	10,00	7,00	4,00
6	,00	-30,00	4,00	3,00
7	,00	40,00	7,00	4,00
8	10,00	-30,00	7,00	4,00
9	,00	10,00	4,00	3,00
10	,00	10,00	4,00	3,00
11	,00	,00	7,00	4,00
12	10,00	-10,00	7,00	4,00
13	,00	20,00	5,00	3,00
14	20,00	-10,00	3,00	2,00
15	10,00	-10,00	5,00	3,00
16	20,00	,00	3,00	2,00
17	-10,00	,00	4,00	3,00
18	.	.	4,00	3,00
19	.	10,00	8,00	5,00
20	,00	10,00	5,00	3,00
21	?	?	?	?
22	?	?	?	?
23	?	?	?	?
24	?	?	?	?
25	?	?	?	?
26	?	?	?	?
27	?	?	?	?
28	?	?	?	?
29	?	?	?	?
30	?	?	?	?
31	?	?	?	?
32	?	?	?	?
33	?	?	?	?
34	10,00	,00	6,00	4,00
35	,00	10,00	8,00	5,00
36	,00	,00	7,00	4,00
37	10,00	10,00	3,00	2,00

	Symptoms.duration	Number.tumors	Number.symptoms
1	7,00	,00	5,00
2	4,00	1,00	4,00
3	1,00	3,00	1,00
4	1,00	,00	1,00
5	3,00	3,00	3,00
6	1,00	,00	,00
7	1,00	2,00	2,00
8	1,00	3,00	,00
9	3,00	2,00	3,00
10	3,00	,00	1,00
11	4,00	2,00	1,00
12	1,00	4,00	3,00
13	1,00	2,00	2,00
14	3,00	,00	2,00
15	6,00	1,00	1,00
16	4,00	1,00	2,00
17	1,00	,00	,00
18	1,00	2,00	2,00
19	1,00	1,00	2,00
20	1,00	3,00	4,00
21	5,00	,00	2,00
22	3,00	,00	1,00
23	1,00	3,00	3,00
24	6,00	1,00	1,00
25	1,00	2,00	2,00
26	1,00	1,00	1,00
27	6,00	1,00	2,00
28	1,00	,00	2,00
29	1,00	,00	1,00
30	1,00	1,00	2,00
31	1,00	1,00	1,00
32	1,00	4,00	2,00
33	3,00	,00	1,00
34	1,00	1,00	2,00
35	1,00	,00	2,00
36	1,00	3,00	1,00
37	1,00	,00	2,00

	Operation.time	Volume.transfosion
1	5,00	,00
2	5,00	,00
3	3,00	,00
4	3,00	,00
5	5,00	,00
6	3,00	,00
7	3,00	,00
8	5,00	849,00
9	2,00	,00
10	3,00	,00
11	2,00	,00
12	2,00	,00
13	2,00	,00
14	4,00	,00
15	2,00	,00
16	3,00	,00
17	2,00	,00
18	3,00	,00
19	3,00	,00
20	5,00	,00
21	5,00	,00
22	3,00	,00
23	3,00	,00
24	2,00	,00
25	5,00	,00
26	2,00	,00
27	3,00	,00
28	3,00	,00
29	2,00	,00
30	4,00	,00
31	4,00	,00
32	2,00	,00
33	4,00	,00
34	3,00	,00
35	2,00	,00
36	2,00	,00
37	3,00	,00

	Volume.transfusion.postOP	Rebleeding	ICU.stay.groups
1	,00	?	?
2	1132,00	?	?
3	,00	?	?
4	,00	?	?
5	,00	?	?
6	,00	?	?
7	,00	?	?
8	,00	?	?
9	,00	?	?
10	,00	?	?
11	,00	?	?
12	,00	?	?
13	,00	?	?
14	,00	?	?
15	,00	?	?
16	,00	?	?
17	,00	?	?
18	,00	?	?
19	,00	?	?
20	,00	?	?
21	,00	?	?
22	,00	?	?
23	,00	?	?
24	,00	?	?
25	,00	?	?
26	,00	?	?
27	,00	?	?
28	,00	?	?
29	,00	?	?
30	,00	?	?
31	,00	?	?
32	,00	?	?
33	,00	?	?
34	,00	?	?
35	,00	?	?
36	,00	?	?
37	,00	?	?

	NCH.stay.groups	Number.symptoms.postOP	Symptoms.postOP	First.symptome.groups
1	3,00	,00	,00	1,00
2	3,00	2,00	1,00	3,00
3	2,00	2,00	1,00	4,00
4	2,00	1,00	1,00	,00
5	4,00	1,00	1,00	3,00
6	1,00	,00	,00	,00
7	2,00	,00	,00	9,00
8	4,00	1,00	1,00	,00
9	1,00	,00	,00	3,00
10	2,00	,00	,00	,00
11	2,00	,00	,00	4,00
12	2,00	,00	,00	3,00
13	1,00	,00	,00	2,00
14	1,00	1,00	1,00	4,00
15	1,00	,00	,00	3,00
16	1,00	,00	,00	5,00
17	1,00	,00	,00	,00
18	1,00	2,00	1,00	4,00
19	3,00	1,00	1,00	5,00
20	1,00	2,00	1,00	5,00
21	2,00	1,00	1,00	4,00
22	1,00	,00	,00	1,00
23	1,00	,00	,00	4,00
24	2,00	1,00	1,00	6,00
25	2,00	2,00	1,00	,00
26	1,00	,00	,00	1,00
27	1,00	,00	,00	1,00
28	2,00	,00	,00	8,00
29	1,00	2,00	1,00	1,00
30	2,00	1,00	1,00	,00
31	2,00	1,00	1,00	6,00
32	2,00	,00	,00	7,00
33	2,00	,00	,00	4,00
34	1,00	1,00	1,00	7,00
35	2,00	1,00	1,00	7,00
36	1,00	,00	,00	8,00
37	1,00	,00	,00	7,00

	Neurological.disorder	Histology.groups	Revision.groups	Localisation.revision
1	1,00	3,00	,00	1,00
2	1,00	4,00	,00	2,00
3	1,00	1,00	,00	2,00
4	1,00	1,00	,00	1,00
5	1,00	3,00	,00	1,00
6	,00	3,00	,00	1,00
7	1,00	3,00	1,00	1,00
8	,00	4,00	1,00	2,00
9	1,00	1,00	,00	1,00
10	1,00	2,00	,00	1,00
11	1,00	3,00	,00	1,00
12	1,00	3,00	1,00	1,00
13	1,00	1,00	,00	1,00
14	1,00	1,00	1,00	1,00
15	1,00	3,00	,00	1,00
16	1,00	1,00	,00	1,00
17	,00	1,00	,00	1,00
18	1,00	1,00	1,00	2,00
19	1,00	1,00	,00	2,00
20	1,00	3,00	1,00	2,00
21	1,00	1,00	,00	1,00
22	1,00	3,00	,00	1,00
23	1,00	5,00	,00	1,00
24	1,00	1,00	,00	1,00
25	1,00	3,00	,00	2,00
26	1,00	1,00	,00	1,00
27	1,00	3,00	,00	1,00
28	1,00	5,00	,00	1,00
29	1,00	1,00	,00	1,00
30	1,00	3,00	,00	2,00
31	1,00	5,00	,00	1,00
32	1,00	3,00	,00	1,00
33	1,00	1,00	,00	1,00
34	1,00	3,00	,00	1,00
35	1,00	3,00	1,00	1,00
36	1,00	3,00	,00	1,00
37	1,00	1,00	,00	1,00

	Masseffect.revision	Simpson.revision	antiepileptc.therapy.revision
1	1,00	1,00	1,00
2	1,00	4,00	,00
3	1,00	1,00	,00
4	,00	1,00	,00
5	1,00	1,00	,00
6	,00	1,00	,00
7	1,00	1,00	,00
8	1,00	1,00	,00
9	1,00	1,00	,00
10	,00	1,00	,00
11	1,00	1,00	,00
12	1,00	1,00	,00
13	,00	1,00	1,00
14	1,00	4,00	,00
15	,00	1,00	,00
16	1,00	1,00	1,00
17	,00	1,00	,00
18	1,00	1,00	,00
19	1,00	1,00	,00
20	1,00	4,00	,00
21	?	?	?
22	?	?	?
23	?	?	?
24	?	?	?
25	?	?	?
26	?	?	?
27	?	?	?
28	?	?	?
29	?	?	?
30	?	?	?
31	?	?	?
32	?	?	?
33	?	?	?
34	?	?	?
35	?	?	?
36	?	?	?
37	?	?	?

	Post.revision.symptoms	Recurrence.revision	ASA.class.4	Kd_disorder
1	,00	,00	3,00	.
2	,00	1,00	3,00	.
3	,00	,00	3,00	,00
4	,00	,00	1,00	1,00
5	1,00	,00	4,00	,00
6	,00	,00	1,00	1,00
7	,00	,00	2,00	,00
8	,00	,00	3,00	1,00
9	,00	,00	3,00	,00
10	,00	,00	3,00	,00
11	,00	,00	3,00	,00
12	,00	,00	2,00	,00
13	,00	,00	3,00	,00
14	,00	1,00	2,00	,00
15	,00	,00	2,00	,00
16	,00	,00	2,00	,00
17	,00	,00	1,00	1,00
18	,00	,00	3,00	.
19	1,00	,00	3,00	.
20	,00	1,00	3,00	,00
21	,00	,00	2,00	,00
22	,00	,00	2,00	,00
23	,00	1,00	2,00	,00
24	,00	,00	2,00	,00
25	1,00	,00	3,00	,00
26	,00	,00	2,00	1,00
27	,00	,00	2,00	,00
28	,00	,00	3,00	,00
29	1,00	,00	2,00	,00
30	,00	,00	3,00	,00
31	,00	,00	2,00	,00
32	,00	,00	2,00	,00
33	,00	,00	2,00	,00
34	,00	,00	2,00	,00
35	,00	,00	3,00	,00
36	,00	,00	3,00	,00
37	,00	,00	2,00	,00

	age_disorder	K1_cut	K1_3gr	Kd_3gr	ASA_di
1	,00	,00	3,00	?	?
2	1,00	1,00	1,00	?	?
3	1,00	1,00	1,00	?	?
4	,00	,00	3,00	?	?
5	1,00	1,00	1,00	?	?
6	,00	,00	3,00	?	?
7	1,00	1,00	1,00	?	?
8	1,00	,00	3,00	?	?
9	,00	,00	2,00	?	?
10	,00	,00	3,00	?	?
11	1,00	,00	3,00	?	?
12	1,00	,00	2,00	?	?
13	,00	1,00	1,00	?	?
14	,00	,00	3,00	?	?
15	,00	,00	3,00	?	?
16	,00	,00	2,00	?	?
17	,00	,00	3,00	?	?
18	,00	1,00	1,00	?	?
19	1,00	1,00	1,00	?	?
20	,00	1,00	1,00	?	?
21	1,00	1,00	1,00	3,00	,00
22	1,00	,00	3,00	2,00	,00
23	,00	,00	3,00	2,00	,00
24	1,00	,00	3,00	2,00	,00
25	1,00	1,00	1,00	2,00	1,00
26	,00	,00	3,00	1,00	,00
27	1,00	,00	2,00	3,00	,00
28	,00	1,00	1,00	3,00	1,00
29	,00	,00	3,00	2,00	,00
30	1,00	1,00	1,00	3,00	1,00
31	1,00	,00	2,00	2,00	,00
32	1,00	1,00	1,00	3,00	,00
33	,00	,00	2,00	3,00	,00
34	1,00	,00	2,00	3,00	,00
35	1,00	1,00	1,00	3,00	1,00
36	1,00	,00	2,00	2,00	1,00
37	,00	,00	2,00	3,00	,00

	Simpson_2gr	WHO_di	Age_cut	Localisation_di
1	,00	,00	,00	.
2	1,00	1,00	1,00	,00
3	,00	,00	1,00	1,00
4	,00	,00	,00	1,00
5	,00	,00	1,00	1,00
6	,00	,00	,00	1,00
7	,00	,00	1,00	1,00
8	1,00	1,00	1,00	.
9	,00	,00	,00	1,00
10	,00	,00	,00	1,00
11	,00	,00	1,00	1,00
12	,00	,00	1,00	,00
13	,00	,00	,00	,00
14	1,00	,00	,00	.
15	,00	,00	,00	1,00
16	,00	,00	,00	,00
17	,00	,00	,00	1,00
18	,00	,00	,00	1,00
19	,00	,00	1,00	1,00
20	1,00	,00	,00	1,00
21	1,00	,00	1,00	1,00
22	,00	,00	1,00	1,00
23	,00	1,00	,00	.
24	,00	,00	1,00	1,00
25	,00	,00	1,00	1,00
26	1,00	,00	,00	1,00
27	1,00	,00	,00	1,00
28	,00	,00	,00	,00
29	,00	,00	,00	,00
30	,00	,00	1,00	1,00
31	1,00	,00	,00	1,00
32	,00	,00	1,00	1,00
33	,00	,00	,00	.
34	,00	,00	1,00	1,00
35	,00	,00	1,00	1,00
36	,00	,00	1,00	1,00
37	,00	,00	,00	1,00

	K3_3gr	Age3gr	RF_r	Reha_r	K3_cut
1	.	1,00	,00	1,00	.
2	.	3,00	1,00	2,00	.
3	3,00	3,00	1,00	1,00	,00
4	3,00	1,00	,00	1,00	,00
5	2,00	3,00	1,00	2,00	1,00
6	2,00	1,00	,00	1,00	1,00
7	2,00	3,00	1,00	1,00	1,00
8	1,00	3,00	1,00	,00	1,00
9	3,00	1,00	1,00	1,00	,00
10	3,00	1,00	,00	1,00	,00
11	3,00	3,00	1,00	1,00	,00
12	2,00	3,00	1,00	1,00	1,00
13	2,00	2,00	1,00	1,00	1,00
14	3,00	1,00	,00	1,00	,00
15	3,00	2,00	1,00	1,00	,00
16	3,00	1,00	1,00	1,00	,00
17	3,00	1,00	,00	1,00	,00
18	.	1,00	1,00	,00	.
19	.	3,00	1,00	2,00	.
20	1,00	2,00	1,00	2,00	1,00
21	2,00	3,00	,00	1,00	1,00
22	3,00	2,00	,00	1,00	,00
23	3,00	1,00	1,00	,00	,00
24	3,00	2,00	1,00	1,00	,00
25	1,00	3,00	1,00	1,00	1,00
26	2,00	2,00	1,00	1,00	1,00
27	3,00	2,00	1,00	,00	,00
28	3,00	2,00	,00	1,00	,00
29	3,00	1,00	,00	1,00	,00
30	1,00	2,00	1,00	2,00	1,00
31	2,00	2,00	1,00	1,00	1,00
32	2,00	3,00	1,00	1,00	1,00
33	3,00	2,00	,00	1,00	,00
34	3,00	2,00	1,00	1,00	,00
35	2,00	3,00	,00	1,00	1,00
36	2,00	3,00	1,00	1,00	1,00
37	3,00	1,00	,00	1,00	,00

	Localisation3gr	localisation2gr	Uni_di	age7groups
1	.	.	1,00	3,00
2	3,00	,00	1,00	7,00
3	2,00	.	1,00	6,00
4	1,00	1,00	1,00	3,00
5	2,00	.	1,00	6,00
6	2,00	.	,00	3,00
7	1,00	1,00	1,00	6,00
8	.	.	1,00	6,00
9	2,00	.	,00	3,00
10	1,00	1,00	,00	3,00
11	1,00	1,00	1,00	6,00
12	3,00	,00	1,00	6,00
13	3,00	,00	,00	4,00
14	.	.	,00	2,00
15	1,00	1,00	,00	4,00
16	3,00	,00	,00	2,00
17	2,00	.	,00	3,00
18	1,00	1,00	1,00	3,00
19	1,00	1,00	1,00	7,00
20	1,00	1,00	1,00	4,00
21	1,00	1,00	1,00	6,00
22	1,00	1,00	,00	5,00
23	.	.	,00	2,00
24	1,00	1,00	,00	5,00
25	2,00	.	1,00	6,00
26	1,00	1,00	,00	4,00
27	1,00	1,00	,00	5,00
28	3,00	,00	,00	4,00
29	3,00	,00	,00	3,00
30	1,00	1,00	1,00	5,00
31	1,00	1,00	1,00	5,00
32	2,00	.	1,00	6,00
33	.	.	1,00	4,00
34	1,00	1,00	,00	5,00
35	1,00	1,00	1,00	7,00
36	1,00	1,00	,00	6,00
37	1,00	1,00	,00	2,00

	Number	Sex	OP.year	Age	Histology.WHO	Type.histology
38	38,00	1,00	2014,00	48,00	2,00	,00
39	39,00	1,00	2014,00	63,00	1,00	3,00
40	40,00	1,00	2014,00	57,00	2,00	12,00
41	?	?	?	?	?	?
42	?	?	?	?	?	?
43	?	?	?	?	?	?
44	?	?	?	?	?	?
45	?	?	?	?	?	?
46	?	?	?	?	?	?
47	?	?	?	?	?	?
48	?	?	?	?	?	?
49	?	?	?	?	?	?
50	?	?	?	?	?	?
51	?	?	?	?	?	?
52	?	?	?	?	?	?
53	?	?	?	?	?	?
54	?	?	?	?	?	?
55	?	?	?	?	?	?
56	?	?	?	?	?	?
57	?	?	?	?	?	?
58	?	?	?	?	?	?
59	?	?	?	?	?	?
60	?	?	?	?	?	?
61	?	?	?	?	?	?
62	?	?	?	?	?	?
63	?	?	?	?	?	?
64	?	?	?	?	?	?
65	?	?	?	?	?	?
66	?	?	?	?	?	?
67	?	?	?	?	?	?
68	?	?	?	?	?	?
69	?	?	?	?	?	?
70	?	?	?	?	?	?
71	?	?	?	?	?	?
72	?	?	?	?	?	?
73	?	?	?	?	?	?
74	?	?	?	?	?	?

	Side	Localisation	Duration.of.symptoms	No.symptoms	First.symptom	Haedache
38	3,00	1,00	,00	,00	,00	,00
39	2,00	9,00	,00	,00	1,00	1,00
40	2,00	2,00	,00	,00	16,00	,00
41	?	?	?	?	?	?
42	?	?	?	?	?	?
43	?	?	?	?	?	?
44	?	?	?	?	?	?
45	?	?	?	?	?	?
46	?	?	?	?	?	?
47	?	?	?	?	?	?
48	?	?	?	?	?	?
49	?	?	?	?	?	?
50	?	?	?	?	?	?
51	?	?	?	?	15,00	,00
52	?	?	?	?	,00	1,00
53	?	?	?	?	4,00	,00
54	?	?	?	?	,00	,00
55	?	?	?	?	,00	,00
56	?	?	?	?	15,00	,00
57	?	?	?	?	12,00	,00
58	?	?	?	?	11,00	1,00
59	?	?	?	?	22,00	,00
60	?	?	?	?	4,00	,00
61	2,00	3,00	36,00	,00	,00	1,00
62	2,00	1,00	3,00	,00	15,00	1,00
63	3,00	4,00	8,00	,00	1,00	1,00
64	2,00	2,00	,00	,00	,00	,00
65	1,00	3,00	,00	1,00	,00	,00
66	1,00	2,00	24,00	,00	4,00	,00
67	1,00	3,00	3,00	,00	21,00	1,00
68	3,00	7,00	1,00	,00	14,00	,00
69	3,00	5,00	2,00	,00	1,00	1,00
70	1,00	1,00	,00	,00	4,00	,00
71	2,00	3,00	,00	,00	4,00	,00
72	3,00	5,00	12,00	,00	15,00	,00
73	2,00	2,00	,00	,00	,00	,00
74	1,00	2,00	2,00	,00	1,00	1,00

	Nausea	Emesis	Nausea_Emesis	Seizures	Oculo.paresis	Viszual.paresis
38	,00	,00	,00	,00	,00	,00
39	1,00	1,00	1,00	,00	,00	,00
40	,00	,00	,00	,00	,00	,00
41	,00	,00	,00	1,00	,00	,00
42	,00	,00	,00	,00	,00	,00
43	,00	,00	,00	,00	,00	,00
44	,00	,00	,00	,00	,00	,00
45	,00	,00	,00	,00	,00	,00
46	,00	,00	,00	,00	,00	,00
47	,00	,00	,00	,00	,00	,00
48	,00	,00	,00	1,00	,00	,00
49	,00	,00	,00	,00	,00	,00
50	,00	,00	,00	,00	,00	,00
51	,00	,00	,00	,00	,00	,00
52	,00	,00	,00	,00	,00	1,00
53	,00	,00	,00	1,00	,00	,00
54	,00	,00	,00	1,00	,00	,00
55	,00	,00	,00	,00	,00	,00
56	,00	,00	,00	,00	,00	,00
57	,00	,00	,00	,00	,00	,00
58	,00	,00	,00	,00	,00	,00
59	,00	,00	,00	,00	,00	,00
60	,00	,00	,00	1,00	,00	,00
61	,00	,00	,00	1,00	,00	,00
62	,00	,00	,00	,00	,00	,00
63	,00	,00	,00	,00	1,00	1,00
64	,00	,00	,00	1,00	,00	,00
65	,00	,00	,00	,00	,00	,00
66	,00	,00	,00	1,00	,00	,00
67	,00	,00	,00	,00	,00	,00
68	,00	,00	,00	,00	,00	,00
69	,00	,00	,00	,00	,00	1,00
70	,00	,00	,00	1,00	,00	,00
71	,00	,00	,00	1,00	,00	,00
72	,00	,00	,00	,00	,00	,00
73	,00	,00	,00	,00	,00	,00
74	1,00	1,00	1,00	,00	,00	,00

	Viszual.deficite	Papilloedema	Optic.atrophies	Exophthalmus	Kakosmia	Other.nerve.paresis
38	,00	,00	,00	,00	,00	,00
39	,00	,00	,00	,00	,00	,00
40	,00	,00	,00	,00	,00	,00
41	,00	,00	,00	,00	,00	,00
42	,00	,00	,00	,00	,00	,00
43	,00	,00	,00	,00	,00	,00
44	,00	,00	,00	,00	,00	,00
45	1,00	,00	,00	,00	,00	,00
46	,00	,00	,00	,00	,00	,00
47	,00	,00	,00	,00	,00	,00
48	,00	,00	,00	,00	,00	,00
49	,00	,00	,00	,00	,00	,00
50	,00	,00	,00	,00	,00	,00
51	,00	,00	,00	,00	,00	,00
52	,00	,00	,00	,00	1,00	,00
53	,00	,00	,00	,00	,00	,00
54	,00	,00	,00	,00	,00	,00
55	,00	,00	,00	,00	,00	,00
56	,00	,00	,00	,00	,00	,00
57	,00	,00	,00	,00	,00	1,00
58	,00	,00	,00	,00	1,00	,00
59	,00	,00	,00	,00	,00	,00
60	,00	,00	,00	,00	,00	,00
61	,00	,00	?	?	?	?
62	,00	,00	?	?	?	?
63	,00	,00	,00	,00	,00	1,00
64	,00	,00	,00	,00	,00	,00
65	,00	,00	,00	1,00	,00	,00
66	,00	,00	,00	,00	,00	,00
67	,00	,00	,00	,00	,00	,00
68	,00	,00	,00	,00	,00	,00
69	1,00	,00	,00	,00	1,00	,00
70	,00	,00	,00	,00	,00	,00
71	,00	,00	,00	,00	,00	,00
72	,00	,00	,00	,00	1,00	1,00
73	,00	,00	,00	,00	,00	1,00
74	,00	,00	,00	,00	,00	1,00

	Sensibility.disorder	Motoric.disorder	Cerebellar.symptoms	Aphasia	Concentration.disoreders	Personallity.change
38	1,00	,0	,00	,00	,00	,00
39	,00	,0	,00	,00	,00	,00
40	,00	,0	,00	1,00	,00	,00
41	,00	,0	,00	,00	,00	,00
42	,00	,0	,00	,00	,00	,00
43	,00	,0	,00	,00	,00	,00
44	,00	1,0	,00	,00	,00	,00
45	,00	,0	,00	,00	,00	,00
46	,00	,0	,00	1,00	,00	,00
47	,00	,0	,00	,00	,00	,00
48	,00	,0	,00	,00	,00	,00
49	,00	,0	1,00	,00	1,00	,00
50	1,00	1,0	,00	,00	,00	,00
51	,00	,0	1,00	,00	,00	,00
52	1,00	,0	,00	,00	,00	,00
53	,00	,0	,00	,00	,00	,00
54	,00	,0	,00	,00	,00	,00
55	,00	,0	,00	,00	,00	1,00
56	1,00	,0	,00	,00	,00	,00
57	,00	,0	,00	,00	,00	,00
58	,00	,0	1,00	,00	,00	,00
59	,00	,0	,00	,00	,00	,00
60	,00	,0	,00	,00	,00	,00
61	,00	1,0	,00	,00	1,00	,00
62	,00	,0	1,00	,00	,00	,00
63	,00	,0	1,00	,00	,00	,00
64	,00	,0	,00	,00	,00	,00
65	,00	,0	,00	,00	,00	,00
66	,00	,0	,00	,00	,00	,00
67	,00	,0	,00	,00	,00	,00
68	1,00	1,0	,00	,00	,00	,00
69	,00	,0	,00	,00	,00	1,00
70	,00	1,0	,00	,00	,00	,00
71	,00	,0	,00	,00	,00	,00
72	,00	,0	1,00	,00	,00	,00
73	,00	,0	,00	1,00	,00	,00
74	,00	1,0	,00	,00	,00	,00

	Other.motoric.disorder	loss.counsciuoness	Double.vision	Histological.clear	Histological.unclear	Size
38	,00	,00	,00	1,00	1,00	2,00
39	,00	,00	,00	1,00	,00	2,00
40	,00	,00	,00	1,00	,00	2,00
41	?	?	?	?	?	?
42	?	?	?	?	?	?
43	?	?	?	?	?	?
44	?	?	?	?	?	?
45	?	?	?	?	?	?
46	?	?	?	?	?	?
47	?	?	?	?	?	?
48	?	?	?	?	?	?
49	?	?	?	?	?	?
50	?	?	?	?	?	?
51	?	?	?	?	?	?
52	?	?	?	?	?	?
53	?	?	?	?	?	?
54	?	?	?	?	?	?
55	?	?	?	?	?	?
56	?	?	?	?	?	?
57	?	?	?	?	?	?
58	?	?	?	?	?	?
59	?	?	?	?	?	?
60	?	?	?	?	?	?
61	?	?	?	?	?	?
62	?	?	?	?	?	?
63	?	?	?	?	?	?
64	?	?	?	?	?	?
65	?	?	?	?	?	?
66	?	?	?	?	?	?
67	?	?	?	?	?	?
68	?	?	?	?	?	?
69	?	?	?	?	?	?
70	?	?	?	?	?	?
71	?	?	?	?	?	?
72	?	?	?	?	?	?
73	?	?	?	?	?	?
74	?	?	?	1,00	2,00	2,00

	MRi.CCT	Form	CSF	Edema	Masseffect	Embolisation
38	2,00	1,00	,00	1,00	1,00	1,00
39	2,00	1,00	,00	1,00	2,00	,00
40	2,00	.	,00	1,00	2,00	,00
41	2,00	2,00	,00	1,00	?	?
42	1,00	1,00	,00	1,00	?	?
43	2,00	1,00	,00	1,00	?	?
44	1,00	.	,00	1,00	?	?
45	2,00	.	,00	1,00	?	?
46	2,00	.	,00	1,00	?	?
47	2,00	2,00	,00	,00	?	?
48	2,00	2,00	,00	1,00	2,00	1,00
49	2,00	1,00	1,00	,00	1,00	,00
50	2,00	.	,00	1,00	1,00	,00
51	2,00	.	,00	,00	1,00	,00
52	2,00	1,00	,00	1,00	,00	,00
53	2,00	2,00	,00	,00	,00	,00
54	2,00	1,00	,00	,00	2,00	1,00
55	2,00	1,00	,00	,00	1,00	1,00
56	2,00	2,00	,00	1,00	2,00	,00
57	2,00	.	,00	,00	1,00	,00
58	2,00	1,00	,00	1,00	,00	,00
59	2,00	2,00	,00	,00	,00	,00
60	2,00	2,00	,00	1,00	2,00	,00
61	2,00	1,00	,00	1,00	2,00	,00
62	2,00	2,00	,00	1,00	,00	,00
63	2,00	.	,00	,00	1,00	,00
64	2,00	1,00	,00	,00	,00	,00
65	2,00	2,00	,00	,00	2,00	,00
66	2,00	.	,00	,00	2,00	,00
67	2,00	2,00	,00	1,00	2,00	,00
68	2,00	1,00	,00	,00	1,00	,00
69	2,00	1,00	,00	1,00	1,00	,00
70	2,00	1,00	,00	1,00	2,00	,00
71	2,00	.	,00	1,00	2,00	1,00
72	2,00	1,00	,00	1,00	1,00	,00
73	2,00	2,00	,00	,00	1,00	,00
74	2,00	1,00	,00	1,00	2,00	,00

	Hypertonia	Adipositas	Heart.disorder	Lung.disorder	Liver.disorder	Kindey.disorder
38	,00	1,00	,00	,00	,00	,00
39	1,00	,00	,00	,00	,00	,00
40	,00	,00	,00	,00	,00	,00
41	,00	,00	,00	,00	,00	,00
42	1,00	,00	,00	1,00	,00	,00
43	,00	,00	,00	,00	,00	,00
44	1,00	1,00	,00	,00	,00	,00
45	1,00	,00	,00	,00	,00	,00
46	,00	,00	,00	,00	,00	,00
47	,00	,00	,00	,00	,00	,00
48	1,00	,00	,00	,00	,00	,00
49	,00	,00	,00	,00	,00	,00
50	,00	,00	,00	,00	,00	,00
51	1,00	,00	,00	,00	,00	,00
52	,00	,00	,00	,00	,00	,00
53	,00	1,00	,00	,00	,00	,00
54	1,00	,00	,00	1,00	,00	,00
55	1,00	,00	,00	1,00	1,00	1,00
56	,00	1,00	,00	,00	,00	1,00
57	,00	,00	,00	,00	,00	,00
58	1,00	,00	,00	,00	,00	,00
59	,00	1,00	,00	,00	,00	,00
60	,00	1,00	,00	,00	,00	,00
61	1,00	,00	,00	,00	,00	,00
62	1,00	,00	1,00	,00	,00	,00
63	1,00	,00	,00	,00	,00	,00
64	,00	1,00	,00	,00	,00	,00
65	,00	,00	,00	,00	,00	,00
66	1,00	1,00	,00	,00	,00	,00
67	,00	,00	,00	,00	,00	,00
68	1,00	,00	,00	,00	,00	,00
69	1,00	,00	,00	,00	,00	,00
70	1,00	,00	1,00	,00	1,00	,00
71	,00	,00	,00	,00	,00	,00
72	1,00	,00	,00	,00	,00	,00
73	1,00	,00	,00	,00	,00	,00
74	,00	,00	,00	,00	,00	,00

	Diabetes	Varicosis	ASA	Simpson.grade	OP.duration	intraOP.brain swelling
38	,00	,00	2,00	4,00	233,00	,00
39	,00	,00	4,00	1,00	315,00	,00
40	,00	,00	3,00	1,00	304,00	,00
41	,00	,00	?	?	?	?
42	,00	,00	?	?	?	?
43	,00	,00	?	?	?	?
44	,00	,00	?	?	?	?
45	,00	,00	?	?	?	?
46	1,00	,00	?	?	?	?
47	,00	,00	?	?	?	?
48	,00	,00	?	?	?	?
49	,00	,00	?	?	?	?
50	,00	,00	?	?	?	?
51	,00	,00	?	?	?	?
52	,00	,00	?	?	?	?
53	,00	,00	?	?	?	?
54	,00	,00	?	?	?	?
55	1,00	1,00	?	?	?	?
56	1,00	,00	?	?	?	?
57	,00	,00	?	?	?	?
58	,00	,00	?	?	?	?
59	,00	,00	?	?	?	?
60	,00	,00	?	?	?	?
61	,00	,00	?	?	?	?
62	,00	,00	?	?	?	?
63	,00	,00	?	?	?	?
64	,00	,00	?	?	?	?
65	,00	,00	?	?	?	?
66	,00	,00	?	?	?	?
67	,00	,00	?	?	?	?
68	,00	,00	?	?	?	?
69	,00	,00	?	?	?	?
70	,00	,00	?	?	?	?
71	,00	,00	?	?	?	?
72	,00	,00	?	?	?	?
73	1,00	,00	?	?	?	?
74	,00	,00	?	?	?	?

	Transfusion	Use.CUSA	Craniotomy	Sinus.lesion	Bleeding	Dura.closure
38	,00	1,00	1,00	1,00	,00	3,00
39	,00	,00	1,00	,00	,00	1,00
40	,00	1,00	1,00	,00	,00	3,00
41	,00	1,00	1,00	,00	1,00	1,00
42	,00	1,00	1,00	,00	,00	1,00
43	,00	1,00	1,00	,00	,00	2,00
44	,00	1,00	2,00	,00	,00	3,00
45	,00	,00	1,00	,00	,00	2,00
46	,00	,00	1,00	1,00	,00	2,00
47	,00	,00	1,00	,00	,00	2,00
48	,00	1,00	1,00	,00	1,00	2,00
49	,00	1,00	1,00	,00	,00	3,00
50	,00	1,00	1,00	,00	,00	3,00
51	,00	1,00	1,00	,00	,00	3,00
52	,00	,00	1,00	,00	,00	2,00
53	,00	,00	1,00	,00	,00	3,00
54	,00	,00	1,00	1,00	,00	2,00
55	,00	,00	1,00	,00	1,00	2,00
56	,00	,00	1,00	,00	1,00	2,00
57	,00	1,00	1,00	,00	,00	2,00
58	,00	,00	1,00	,00	,00	2,00
59	,00	1,00	1,00	,00	,00	2,00
60	,00	1,00	1,00	,00	1,00	2,00
61	,00	1,00	1,00	,00	1,00	3,00
62	,00	,00	1,00	,00	,00	3,00
63	,00	,00	1,00	,00	,00	2,00
64	,00	,00	1,00	1,00	,00	3,00
65	,00	1,00	1,00	,00	,00	3,00
66	,00	,00	1,00	,00	,00	2,00
67	,00	1,00	1,00	,00	,00	3,00
68	,00	1,00	2,00	,00	,00	3,00
69	,00	1,00	1,00	,00	,00	3,00
70	,00	,00	1,00	,00	,00	3,00
71	,00	1,00	1,00	,00	,00	2,00
72	,00	1,00	1,00	,00	,00	2,00
73	,00	,00	2,00	,00	,00	3,00
74	,00	1,00	1,00	,00	,00	3,00

	Tachosil.Fibrin	Transfusion.postOP	Seizure.therapy	Antibiotics.postOP	CSF.circulatory.disorder	Edema.postOP
38	1,00	,00	1,00	,00	,00	,00
39	1,00	,00	,00	,00	,00	,00
40	1,00	,00	,00	,00	,00	,00
41	1,00	,00	1,00	,00	,00	,00
42	1,00	,00	,00	,00	,00	1,00
43	1,00	,00	,00	,00	,00	,00
44	1,00	,00	,00	,00	,00	,00
45	1,00	,00	,00	,00	,00	,00
46	1,00	2,00	,00	,00	,00	,00
47	1,00	,00	,00	,00	,00	,00
48	1,00	,00	,00	,00	,00	,00
49	1,00	,00	,00	,00	,00	1,00
50	1,00	,00	,00	,00	,00	,00
51	1,00	,00	,00	,00	,00	,00
52	1,00	,00	,00	,00	,00	,00
53	1,00	,00	1,00	,00	,00	,00
54	1,00	,00	,00	,00	,00	,00
55	1,00	,00	,00	,00	,00	1,00
56	1,00	,00	,00	,00	,00	,00
57	1,00	,00	,00	,00	,00	,00
58	1,00	,00	,00	,00	,00	,00
59	1,00	,00	,00	,00	,00	,00
60	1,00	,00	2,00	,00	,00	,00
61	1,00	,00	1,00	,00	,00	,00
62	1,00	,00	,00	,00	,00	,00
63	1,00	,00	2,00	,00	,00	,00
64	1,00	,00	2,00	,00	,00	,00
65	1,00	,00	,00	,00	,00	,00
66	1,00	,00	,00	,00	,00	,00
67	1,00	,00	2,00	,00	,00	,00
68	1,00	,00	,00	,00	,00	,00
69	1,00	,00	,00	,00	,00	,00
70	1,00	,00	2,00	,00	,00	,00
71	1,00	,00	2,00	,00	,00	,00
72	1,00	,00	,00	1,00	,00	,00
73	1,00	,00	1,00	,00	,00	,00
74	1,00	,00	,00	1,00	,00	,00

	Kind.of.bleeding	Infection	Revision1	Revision2	Revision3	Seizures.postOP
38	,00	,00	,00	,00	,00	,00
39	1,00	,00	,00	,00	,00	,00
40	,00	,00	,00	,00	,00	,00
41	,00	,00	,00	,00	,00	,00
42	,00	,00	1,00	,00	,00	,00
43	,00	,00	,00	,00	,00	,00
44	,00	,00	,00	,00	,00	,00
45	,00	,00	,00	,00	,00	,00
46	,00	,00	2,00	,00	,00	,00
47	,00	,00	,00	,00	,00	,00
48	,00	,00	,00	,00	,00	,00
49	2,00	,00	1,00	,00	,00	,00
50	,00	,00	,00	,00	,00	,00
51	,00	,00	,00	,00	,00	,00
52	,00	,00	,00	,00	,00	,00
53	,00	,00	,00	,00	,00	,00
54	,00	,00	,00	,00	,00	,00
55	2,00	,00	,00	,00	,00	,00
56	,00	,00	,00	,00	,00	,00
57	,00	,00	,00	,00	,00	,00
58	,00	,00	,00	,00	,00	,00
59	,00	,00	,00	,00	,00	,00
60	,00	,00	,00	,00	,00	1,00
61	,00	,00	,00	,00	?	?
62	,00	,00	,00	,00	?	?
63	,00	,00	,00	,00	?	?
64	,00	,00	,00	,00	?	?
65	,00	,00	,00	,00	?	?
66	,00	,00	,00	,00	?	?
67	,00	,00	,00	,00	?	?
68	,00	,00	,00	,00	?	?
69	1,00	1,00	3,00	2,00	?	?
70	,00	,00	,00	,00	?	?
71	,00	,00	,00	,00	?	?
72	,00	1,00	2,00	,00	?	?
73	,00	,00	,00	,00	?	?
74	,00	1,00	2,00	,00	?	?

	Thro.Emb	D.insidipus	Dys.Aphasia	Sens.Hemi	Motor.Hemi	Other.symptoms
38	,00	,00	,00	,00	1,00	,00
39	,00	,00	,00	,00	1,00	,00
40	,00	,00	,00	,00	,00	,00
41	,00	,00	,00	,00	,00	,00
42	,00	,00	,00	,00	1,00	,00
43	,00	,00	,00	,00	,00	,00
44	,00	,00	,00	,00	,00	1,00
45	,00	,00	,00	,00	,00	1,00
46	,00	,00	,00	,00	,00	1,00
47	,00	,00	,00	,00	,00	1,00
48	,00	,00	,00	,00	,00	1,00
49	,00	,00	,00	,00	,00	1,00
50	,00	,00	,00	,00	,00	,00
51	,00	,00	,00	,00	,00	1,00
52	,00	,00	,00	,00	,00	,00
53	,00	,00	,00	,00	,00	,00
54	,00	,00	,00	,00	,00	,00
55	1,00	,00	,00	,00	1,00	1,00
56	,00	,00	,00	,00	,00	,00
57	,00	,00	,00	,00	,00	,00
58	,00	,00	,00	,00	,00	,00
59	,00	,00	,00	,00	,00	,00
60	,00	,00	,00	,00	,00	,00
61	,00	,00	,00	,00	,00	,00
62	,00	,00	,00	,00	,00	,00
63	,00	,00	,00	,00	,00	1,00
64	,00	,00	,00	,00	,00	,00
65	,00	,00	,00	,00	,00	,00
66	,00	,00	,00	,00	,00	,00
67	,00	,00	,00	,00	,00	1,00
68	,00	,00	,00	,00	,00	1,00
69	,00	,00	,00	,00	,00	,00
70	,00	,00	,00	,00	,00	,00
71	,00	,00	,00	,00	,00	1,00
72	,00	,00	,00	,00	,00	,00
73	,00	,00	,00	,00	,00	,00
74	,00	,00	,00	,00	,00	,00

	ICU.stay	NCH.stay	Total.duration	Rehabilitation	Radiation	Recurrence
38	1,00	13,00	14,00	1,00	1,00	2,00
39	3,00	7,00	10,00	1,00	,00	,00
40	3,00	6,00	9,00	,00	1,00	,00
41	1,00	6,00	7,00	1,00	,00	,00
42	7,00	7,00	14,00	3,00	,00	,00
43	1,00	7,00	8,00	1,00	,00	,00
44	5,00	7,00	12,00	1,00	,00	,00
45	2,00	6,00	8,00	1,00	1,00	,00
46	1,00	8,00	9,00	1,00	,00	2,00
47	1,00	7,00	8,00	1,00	,00	,00
48	2,00	6,00	8,00	1,00	,00	,00
49	15,00	14,00	29,00	3,00	,00	,00
50	2,00	6,00	8,00	1,00	,00	,00
51	3,00	11,00	14,00	1,00	,00	,00
52	1,00	6,00	7,00	1,00	,00	,00
53	1,00	5,00	6,00	,00	1,00	,00
54	2,00	7,00	9,00	1,00	,00	,00
55	22,00	13,00	35,00	3,00	,00	,00
56	2,00	14,00	31,00	3,00	,00	,00
57	1,00	8,00	9,00	1,00	,00	,00
58	1,00	7,00	8,00	1,00	,00	,00
59	1,00	6,00	7,00	1,00	,00	,00
60	2,00	9,00	11,00	1,00	,00	,00
61	4,00	14,00	18,00	,00	,00	,00
62	1,00	5,00	6,00	1,00	,00	,00
63	5,00	14,00	19,00	1,00	,00	2,00
64	1,00	6,00	7,00	1,00	,00	,00
65	2,00	11,00	13,00	1,00	,00	,00
66	1,00	6,00	7,00	1,00	,00	,00
67	1,00	6,00	7,00	1,00	,00	1,00
68	3,00	20,00	23,00	3,00	,00	,00
69	5,00	15,00	20,00	1,00	,00	,00
70	1,00	7,00	8,00	1,00	,00	,00
71	4,00	7,00	11,00	1,00	,00	,00
72	1,00	5,00	6,00	1,00	,00	,00
73	2,00	9,00	11,00	1,00	,00	,00
74	2,00	5,00	7,00	,00	,00	,00

	Recurrence1	Daeth	Karnofsky.score.pre	Karnofsky.score.post1.3.month	Karnofsky.score.post6.12.month	Difference.K3.K1
38	1,00	,00	80,00	70,00	70,00	-10,00
39	,00	,00	30,00	70,00	.	.
40	,00	,00	50,00	70,00	70,00	20,00
41	,00	,00	70,00	70,00	70,00	,00
42	,00	,00	30,00	70,00	70,00	40,00
43	,00	,00	90,00	80,00	80,00	-10,00
44	,00	,00	30,00	60,00	80,00	50,00
45	,00	,00	80,00	70,00	80,00	,00
46	,00	,00	70,00	70,00	70,00	,00
47	,00	,00	90,00	70,00	80,00	-10,00
48	,00	,00	80,00	70,00	70,00	-10,00
49	,00	,00	70,00	70,00	70,00	,00
50	,00	,00	80,00	70,00	80,00	,00
51	,00	,00	70,00	70,00	70,00	,00
52	,00	,00	80,00	70,00	80,00	,00
53	1,00	,00	80,00	70,00	80,00	,00
54	,00	,00	80,00	70,00	80,00	,00
55	,00	,00	70,00	60,00	.	.
56	,00	,00	50,00	60,00	60,00	10,00
57	,00	,00	90,00	70,00	70,00	-20,00
58	,00	,00	70,00	70,00	80,00	10,00
59	,00	,00	80,00	80,00	90,00	10,00
60	,00	,00	80,00	70,00	70,00	-10,00
61	,00	,00	60,00	.	.	.
62	,00	,00	80,00	70,00	70,00	-10,00
63	1,00	,00	80,00	70,00	70,00	-10,00
64	1,00	,00	70,00	60,00	70,00	,00
65	,00	,00	70,00	70,00	80,00	10,00
66	,00	,00	60,00	70,00	70,00	10,00
67	,00	,00	80,00	70,00	70,00	-10,00
68	,00	,00	60,00	50,00	60,00	,00
69	,00	,00	80,00	40,00	70,00	-10,00
70	,00	,00	80,00	70,00	70,00	-10,00
71	1,00	,00	80,00	70,00	70,00	-10,00
72	,00	,00	80,00	40,00	70,00	-10,00
73	1,00	,00	70,00	.	.	.
74	1,00	,00	60,00	50,00	60,00	,00

	Diffenrence.K3.K2	K2K1	Agegroup1	Agegroup2
38	,00	-10,00	4,00	3,00
39	.	40,00	6,00	4,00
40	,00	20,00	5,00	3,00
41	?	?	?	?
42	?	?	?	?
43	?	?	?	?
44	?	?	?	?
45	?	?	?	?
46	?	?	?	?
47	?	?	?	?
48	?	?	?	?
49	?	?	?	?
50	?	?	?	?
51	?	?	?	?
52	?	?	?	?
53	?	?	?	?
54	?	?	?	?
55	?	?	?	?
56	?	?	?	?
57	?	?	?	?
58	?	?	?	?
59	?	?	?	?
60	?	?	?	?
61	?	?	5,00	3,00
62	,00	-10,00	7,00	4,00
63	,00	-10,00	5,00	3,00
64	10,00	-10,00	3,00	2,00
65	10,00	,00	4,00	3,00
66	,00	10,00	6,00	4,00
67	,00	-10,00	3,00	2,00
68	10,00	-10,00	7,00	4,00
69	30,00	-40,00	5,00	3,00
70	,00	-10,00	6,00	4,00
71	,00	-10,00	5,00	3,00
72	30,00	-40,00	6,00	4,00
73	.	.	7,00	4,00
74	10,00	-10,00	2,00	2,00

	Symptoms.duration	Number.tumors	Number.symptoms
38	1,00	1,00	1,00
39	1,00	1,00	3,00
40	1,00	,00	1,00
41	1,00	,00	1,00
42	1,00	2,00	1,00
43	1,00	,00	,00
44	1,00	2,00	2,00
45	3,00	1,00	2,00
46	2,00	1,00	2,00
47	5,00	,00	1,00
48	1,00	1,00	1,00
49	4,00	,00	2,00
50	2,00	,00	2,00
51	1,00	1,00	2,00
52	1,00	,00	4,00
53	2,00	1,00	1,00
54	1,00	2,00	10,00
55	1,00	6,00	1,00
56	2,00	3,00	1,00
57	5,00	,00	1,00
58	3,00	1,00	3,00
59	7,00	1,00	,00
60	6,00	1,00	1,00
61	6,00	1,00	4,00
62	2,00	2,00	2,00
63	4,00	1,00	14,00
64	1,00	1,00	1,00
65	1,00	,00	1,00
66	5,00	2,00	1,00
67	2,00	,00	2,00
68	1,00	1,00	2,00
69	2,00	1,00	5,00
70	1,00	3,00	2,00
71	1,00	,00	1,00
72	4,00	1,00	3,00
73	1,00	2,00	2,00
74	2,00	,00	5,00

	Operation.time	Volume.transfosion
38	2,00	,00
39	3,00	,00
40	3,00	,00
41	4,00	,00
42	3,00	,00
43	2,00	,00
44	3,00	,00
45	2,00	,00
46	3,00	,00
47	4,00	,00
48	4,00	,00
49	5,00	,00
50	5,00	,00
51	2,00	,00
52	3,00	,00
53	3,00	,00
54	2,00	,00
55	4,00	,00
56	5,00	,00
57	3,00	,00
58	2,00	,00
59	2,00	,00
60	3,00	,00
61	4,00	,00
62	2,00	,00
63	5,00	,00
64	2,00	,00
65	5,00	,00
66	2,00	,00
67	5,00	,00
68	4,00	,00
69	5,00	,00
70	3,00	,00
71	4,00	,00
72	4,00	,00
73	3,00	,00
74	2,00	,00

	Volume.transfusion.postOP	Rebleeding	ICU.stay.groups
38	,00	,00	1,00
39	,00	1,00	2,00
40	,00	,00	2,00
41	,00	?	?
42	,00	?	?
43	,00	?	?
44	,00	?	?
45	,00	?	?
46	566,00	?	?
47	,00	?	?
48	,00	?	?
49	,00	?	?
50	,00	?	?
51	,00	?	?
52	,00	?	?
53	,00	?	?
54	,00	?	?
55	,00	?	?
56	,00	?	?
57	,00	?	?
58	,00	?	?
59	,00	?	?
60	,00	?	?
61	,00	?	?
62	,00	?	?
63	,00	?	?
64	,00	?	?
65	,00	?	?
66	,00	?	?
67	,00	?	?
68	,00	?	?
69	,00	?	?
70	,00	?	?
71	,00	?	?
72	,00	?	?
73	,00	?	?
74	,00	?	?

	NCH.stay.groups	Number.symptoms.postOP	Symptoms.postOP	First.symptome.groups
38	2,00	1,00	1,00	,00
39	1,00	1,00	1,00	1,00
40	1,00	,00	,00	8,00
41	1,00	,00	,00	,00
42	1,00	1,00	1,00	,00
43	1,00	,00	,00	,00
44	1,00	1,00	1,00	1,00
45	1,00	1,00	1,00	4,00
46	2,00	1,00	1,00	1,00
47	1,00	1,00	1,00	4,00
48	1,00	1,00	1,00	,00
49	2,00	1,00	1,00	3,00
50	1,00	,00	,00	5,00
51	2,00	1,00	1,00	7,00
52	1,00	,00	,00	,00
53	1,00	,00	,00	2,00
54	1,00	,00	,00	,00
55	2,00	3,00	1,00	,00
56	2,00	,00	,00	7,00
57	2,00	,00	,00	6,00
58	1,00	,00	,00	6,00
59	1,00	,00	,00	9,00
60	2,00	1,00	1,00	2,00
61	2,00	,00	,00	,00
62	1,00	,00	,00	7,00
63	2,00	2,00	1,00	1,00
64	1,00	1,00	1,00	,00
65	2,00	,00	,00	,00
66	1,00	,00	,00	2,00
67	1,00	2,00	1,00	4,00
68	3,00	1,00	1,00	5,00
69	3,00	,00	,00	1,00
70	1,00	,00	,00	2,00
71	1,00	1,00	1,00	2,00
72	1,00	,00	,00	7,00
73	2,00	,00	,00	,00
74	1,00	,00	,00	1,00

	Neurological.disorder	Histology.groups	Revision.groups	Localisation.revision
38	1,00	5,00	,00	2,00
39	1,00	3,00	,00	1,00
40	1,00	4,00	,00	1,00
41	1,00	5,00	,00	1,00
42	1,00	3,00	1,00	2,00
43	,00	2,00	,00	1,00
44	1,00	1,00	,00	1,00
45	1,00	1,00	,00	1,00
46	1,00	1,00	1,00	1,00
47	1,00	1,00	,00	1,00
48	1,00	5,00	,00	1,00
49	1,00	3,00	1,00	2,00
50	1,00	3,00	,00	2,00
51	1,00	1,00	,00	1,00
52	1,00	3,00	,00	1,00
53	1,00	4,00	,00	1,00
54	1,00	3,00	,00	1,00
55	1,00	4,00	,00	1,00
56	1,00	1,00	,00	1,00
57	1,00	1,00	,00	1,00
58	1,00	3,00	,00	1,00
59	,00	2,00	,00	1,00
60	1,00	1,00	,00	2,00
61	1,00	3,00	,00	1,00
62	1,00	4,00	,00	1,00
63	1,00	1,00	,00	2,00
64	1,00	4,00	,00	1,00
65	1,00	1,00	,00	1,00
66	1,00	3,00	,00	1,00
67	1,00	3,00	,00	1,00
68	1,00	1,00	,00	2,00
69	1,00	4,00	1,00	2,00
70	1,00	3,00	,00	1,00
71	1,00	1,00	,00	1,00
72	1,00	1,00	1,00	2,00
73	1,00	4,00	,00	1,00
74	1,00	4,00	1,00	1,00

	Masseffect.revision	Simpson.revision	antiepileptc.therapy.revision
38	1,00	4,00	1,00
39	1,00	1,00	,00
40	1,00	1,00	,00
41	1,00	1,00	1,00
42	1,00	1,00	,00
43	,00	1,00	,00
44	1,00	4,00	,00
45	,00	4,00	,00
46	1,00	1,00	,00
47	,00	1,00	,00
48	1,00	1,00	,00
49	1,00	4,00	,00
50	1,00	4,00	,00
51	1,00	1,00	,00
52	,00	1,00	,00
53	,00	1,00	1,00
54	1,00	1,00	,00
55	1,00	1,00	,00
56	1,00	1,00	,00
57	1,00	1,00	,00
58	,00	1,00	,00
59	,00	1,00	,00
60	1,00	1,00	1,00
61	1,00	1,00	1,00
62	,00	1,00	,00
63	1,00	1,00	1,00
64	,00	1,00	1,00
65	1,00	4,00	,00
66	1,00	1,00	,00
67	1,00	1,00	1,00
68	1,00	1,00	,00
69	1,00	1,00	,00
70	1,00	1,00	1,00
71	1,00	1,00	1,00
72	1,00	4,00	,00
73	1,00	1,00	1,00
74	1,00	1,00	,00

	Post.revision.symptoms	Recurrence.revision	ASA.class.4	Kd_disorder
38	1,00	1,00	2,00	1,00
39	1,00	,00	4,00	.
40	,00	,00	3,00	,00
41	,00	,00	2,00	,00
42	1,00	,00	4,00	,00
43	,00	,00	2,00	1,00
44	,00	,00	3,00	,00
45	,00	1,00	2,00	,00
46	,00	1,00	2,00	,00
47	,00	,00	2,00	1,00
48	,00	,00	2,00	1,00
49	,00	,00	3,00	,00
50	,00	,00	2,00	,00
51	,00	,00	2,00	,00
52	,00	,00	2,00	,00
53	,00	1,00	2,00	,00
54	,00	,00	3,00	,00
55	1,00	,00	2,00	.
56	,00	,00	3,00	,00
57	,00	,00	2,00	1,00
58	,00	,00	2,00	,00
59	,00	,00	2,00	,00
60	,00	,00	2,00	1,00
61	,00	,00	2,00	.
62	,00	1,00	2,00	1,00
63	,00	1,00	1,00	1,00
64	,00	,00	2,00	,00
65	,00	,00	2,00	,00
66	,00	,00	2,00	,00
67	,00	1,00	1,00	1,00
68	,00	,00	2,00	,00
69	,00	,00	2,00	1,00
70	,00	,00	2,00	1,00
71	,00	1,00	2,00	1,00
72	,00	,00	3,00	1,00
73	,00	,00	2,00	.
74	,00	,00	3,00	,00

	age_disorder	K1_cut	K1_3gr	Kd_3gr	ASA_di
38	,00	,00	3,00	1,00	,00
39	1,00	1,00	1,00	.	1,00
40	,00	1,00	1,00	3,00	1,00
41	,00	,00	2,00	?	?
42	1,00	1,00	1,00	?	?
43	1,00	,00	3,00	?	?
44	,00	1,00	1,00	?	?
45	,00	,00	3,00	?	?
46	,00	,00	2,00	?	?
47	,00	,00	3,00	?	?
48	,00	,00	3,00	?	?
49	1,00	,00	2,00	?	?
50	,00	,00	3,00	?	?
51	1,00	,00	2,00	2,00	,00
52	1,00	,00	3,00	2,00	,00
53	,00	,00	3,00	2,00	,00
54	1,00	,00	3,00	2,00	1,00
55	1,00	,00	2,00	.	,00
56	1,00	1,00	1,00	3,00	1,00
57	,00	,00	3,00	1,00	,00
58	1,00	,00	2,00	3,00	,00
59	,00	,00	3,00	3,00	,00
60	,00	,00	3,00	1,00	,00
61	,00	1,00	1,00	?	?
62	1,00	,00	3,00	?	?
63	,00	,00	3,00	?	?
64	,00	,00	2,00	?	?
65	,00	,00	2,00	?	?
66	1,00	1,00	1,00	?	?
67	,00	,00	3,00	?	?
68	1,00	1,00	1,00	?	?
69	,00	,00	3,00	?	?
70	1,00	,00	3,00	?	?
71	,00	,00	3,00	?	?
72	1,00	,00	3,00	?	?
73	1,00	,00	2,00	?	?
74	,00	1,00	1,00	?	?

	Simpson_2gr	WHO_di	Age_cut	Localisation_di
38	1,00	1,00	,00	1,00
39	,00	,00	,00	.
40	,00	1,00	,00	,00
41	,00	,00	,00	1,00
42	,00	,00	,00	1,00
43	,00	,00	1,00	1,00
44	1,00	,00	,00	1,00
45	1,00	,00	,00	.
46	,00	,00	,00	,00
47	,00	,00	,00	1,00
48	,00	,00	,00	1,00
49	1,00	,00	1,00	.
50	1,00	,00	,00	1,00
51	,00	,00	1,00	1,00
52	,00	,00	,00	1,00
53	,00	1,00	,00	1,00
54	,00	,00	1,00	,00
55	,00	1,00	1,00	,00
56	,00	,00	1,00	1,00
57	,00	,00	,00	1,00
58	,00	,00	1,00	,00
59	,00	,00	,00	,00
60	,00	,00	,00	,00
61	?	?	?	?
62	?	?	?	?
63	?	?	?	?
64	?	?	?	?
65	?	?	?	?
66	?	?	?	?
67	?	?	?	?
68	?	?	?	?
69	?	?	?	?
70	?	?	?	?
71	?	?	?	?
72	?	?	?	?
73	?	?	?	?
74	?	?	?	?

	K3_3gr	Age3gr	RF_r	Reha_r	K3_cut
38	2,00	1,00	1,00	1,00	1,00
39	.	2,00	1,00	1,00	.
40	2,00	2,00	,00	,00	1,00
41	2,00	2,00	,00	1,00	1,00
42	2,00	2,00	1,00	2,00	1,00
43	3,00	2,00	,00	1,00	,00
44	3,00	2,00	1,00	1,00	,00
45	3,00	2,00	1,00	1,00	,00
46	2,00	2,00	1,00	1,00	1,00
47	3,00	2,00	,00	1,00	,00
48	2,00	2,00	1,00	1,00	1,00
49	2,00	2,00	,00	2,00	1,00
50	3,00	1,00	,00	1,00	,00
51	2,00	3,00	1,00	1,00	1,00
52	3,00	2,00	,00	1,00	,00
53	3,00	1,00	1,00	,00	,00
54	3,00	3,00	1,00	1,00	,00
55	.	2,00	1,00	2,00	.
56	1,00	3,00	1,00	2,00	1,00
57	2,00	1,00	,00	1,00	1,00
58	3,00	2,00	1,00	1,00	,00
59	3,00	2,00	1,00	1,00	,00
60	2,00	1,00	1,00	1,00	1,00
61	.	2,00	1,00	,00	.
62	2,00	3,00	1,00	1,00	1,00
63	2,00	2,00	1,00	1,00	1,00
64	2,00	1,00	1,00	1,00	1,00
65	3,00	1,00	,00	1,00	,00
66	2,00	2,00	1,00	1,00	1,00
67	2,00	1,00	,00	1,00	1,00
68	1,00	3,00	1,00	2,00	1,00
69	2,00	2,00	1,00	1,00	1,00
70	2,00	2,00	1,00	1,00	1,00
71	2,00	2,00	,00	1,00	1,00
72	2,00	2,00	1,00	1,00	1,00
73	.	3,00	1,00	1,00	.
74	1,00	1,00	,00	,00	1,00

	Localisation3gr	localisation2gr	Uni_di	age7groups
38	2,00	.	1,00	3,00
39	.	.	,00	5,00
40	3,00	,00	,00	4,00
41	1,00	1,00	,00	4,00
42	2,00	.	1,00	5,00
43	2,00	.	,00	5,00
44	1,00	1,00	1,00	4,00
45	.	.	,00	4,00
46	3,00	,00	,00	4,00
47	1,00	1,00	,00	4,00
48	1,00	1,00	,00	4,00
49	.	.	1,00	5,00
50	1,00	1,00	,00	3,00
51	1,00	1,00	1,00	6,00
52	1,00	1,00	,00	5,00
53	2,00	.	,00	2,00
54	3,00	,00	,00	6,00
55	3,00	,00	1,00	5,00
56	1,00	1,00	1,00	6,00
57	1,00	1,00	,00	3,00
58	3,00	,00	,00	5,00
59	3,00	,00	,00	3,00
60	3,00	,00	,00	2,00
61	1,00	1,00	1,00	4,00
62	2,00	.	,00	6,00
63	1,00	1,00	1,00	4,00
64	3,00	,00	,00	2,00
65	1,00	1,00	1,00	3,00
66	3,00	,00	,00	5,00
67	1,00	1,00	,00	2,00
68	1,00	1,00	1,00	6,00
69	1,00	1,00	1,00	4,00
70	2,00	.	,00	5,00
71	1,00	1,00	,00	4,00
72	1,00	1,00	,00	5,00
73	3,00	,00	,00	6,00
74	3,00	,00	,00	1,00

	Number	Sex	OP.year	Age	Histology.WHO	Type.histology
75	75,00	1,00	2012,00	61,00	1,00	1,00
76	76,00	,00	2012,00	69,00	2,00	12,00
77	77,00	1,00	2012,00	52,00	1,00	1,00
78	78,00	1,00	2012,00	42,00	1,00	1,00
79	79,00	1,00	2012,00	63,00	1,00	4,00
80	80,00	1,00	2012,00	54,00	1,00	4,00
81	81,00	1,00	2012,00	75,00	1,00	1,00
82	82,00	,00	2012,00	58,00	1,00	4,00
83	83,00	1,00	2012,00	81,00	1,00	4,00
84	84,00	,00	2012,00	43,00	1,00	5,00
85	85,00	1,00	2012,00	67,00	1,00	5,00
86	86,00	,00	2012,00	73,00	1,00	1,00
87	87,00	1,00	2012,00	46,00	1,00	1,00
88	88,00	1,00	2012,00	70,00	1,00	1,00
89	89,00	,00	2012,00	66,00	1,00	1,00
90	90,00	1,00	2012,00	35,00	1,00	2,00
91	91,00	1,00	2012,00	76,00	1,00	1,00
92	92,00	1,00	2012,00	60,00	1,00	2,00
93	93,00	1,00	2012,00	74,00	2,00	12,00
94	94,00	,00	2012,00	62,00	1,00	1,00
95	95,00	1,00	2011,00	46,00	1,00	1,00
96	96,00	,00	2012,00	79,00	3,00	15,00
97	97,00	1,00	2012,00	61,00	1,00	1,00
98	98,00	,00	2012,00	25,00	1,00	1,00
99	99,00	1,00	2012,00	40,00	1,00	4,00
100	100,00	,00	2012,00	55,00	2,00	12,00
101	101,00	1,00	2012,00	71,00	1,00	1,00
102	102,00	,00	2012,00	72,00	1,00	1,00
103	103,00	1,00	2012,00	48,00	1,00	2,00
104	104,00	1,00	2012,00	70,00	1,00	1,00
105	105,00	1,00	2012,00	47,00	1,00	1,00
106	106,00	,00	2012,00	72,00	1,00	1,00
107	107,00	,00	2012,00	68,00	1,00	1,00
108	108,00	1,00	2012,00	49,00	1,00	2,00
109	109,00	,00	2012,00	64,00	1,00	1,00
110	110,00	,00	2012,00	59,00	1,00	1,00
111	111,00	1,00	2012,00	64,00	1,00	3,00

	Side	Localisation	Duration.of.symptoms	No.symptoms	First.symptom	Haedache
75	2,00	11,00	8,00	,00	15,00	1,00
76	1,00	3,00	,00	,00	,00	,00
77	2,00	5,00	,00	1,00	,00	,00
78	1,00	3,00	3,00	,00	1,00	1,00
79	2,00	3,00	36,00	,00	22,00	,00
80	2,00	2,00	4,00	,00	15,00	1,00
81	3,00	1,00	,00	1,00	,00	,00
82	2,00	3,00	,00	1,00	,00	,00
83	2,00	3,00	,00	1,00	4,00	,00
84	2,00	1,00	5,00	,00	1,00	1,00
85	2,00	11,00	48,00	,00	12,00	,00
86	2,00	2,00	28,00	,00	4,00	,00
87	2,00	3,00	6,00	,00	6,00	,00
88	3,00	5,00	,00	,00	7,00	,00
89	1,00	2,00	,00	,00	4,00	,00
90	1,00	6,00	1,00	,00	15,00	1,00
91	3,00	5,00	,00	,00	,00	,00
92	1,00	1,00	,00	,00	1,00	1,00
93	3,00	1,00	3,00	,00	15,00	1,00
94	2,00	3,00	,00	1,00	,00	,00
95	1,00	13,00	,00	,00	,00	,00
96	1,00	2,00	,00	,00	18,00	,00
97	2,00	4,00	3,00	,00	6,00	,00
98	2,00	2,00	2,00	,00	15,00	,00
99	3,00	5,00	1,00	,00	4,00	,00
100	3,00	1,00	1,00	,00	14,00	,00
101	3,00	1,00	,00	,00	,00	,00
102	3,00	3,00	6,00	1,00	21,00	,00
103	1,00	1,00	36,00	1,00	13,00	1,00
104	2,00	2,00	,00	1,00	,00	,00
105	1,00	3,00	1,00	,00	6,00	,00
106	2,00	1,00	12,00	,00	15,00	,00
107	2,00	2,00	,00	1,00	,00	,00
108	2,00	2,00	36,00	,00	1,00	1,00
109	3,00	5,00	13,00	,00	17,00	,00
110	3,00	4,00	60,00	,00	6,00	,00
111	2,00	5,00	2,00	,00	6,00	,00

	Nausea	Emesis	Nausea_Emesis	Seizures	Oculo.paresis	Viszual.paresis
75	1,00	1,00	1,00	,00	,00	,00
76	,00	,00	,00	,00	,00	,00
77	,00	,00	,00	,00	,00	,00
78	,00	,00	,00	,00	,00	,00
79	,00	,00	,00	,00	,00	,00
80	,00	,00	,00	,00	,00	,00
81	,00	,00	,00	,00	,00	,00
82	,00	,00	,00	,00	,00	,00
83	,00	,00	,00	1,00	,00	,00
84	,00	,00	,00	,00	,00	,00
85	,00	,00	,00	,00	,00	,00
86	,00	,00	,00	1,00	,00	,00
87	,00	,00	,00	,00	,00	1,00
88	,00	,00	,00	,00	,00	,00
89	,00	,00	,00	1,00	,00	,00
90	,00	,00	,00	,00	,00	,00
91	,00	,00	,00	,00	,00	,00
92	,00	,00	,00	,00	,00	,00
93	,00	,00	,00	1,00	,00	1,00
94	,00	,00	,00	,00	,00	,00
95	,00	,00	,00	,00	,00	,00
96	,00	,00	,00	,00	,00	,00
97	,00	,00	,00	,00	,00	1,00
98	,00	,00	,00	,00	,00	,00
99	,00	,00	,00	1,00	,00	,00
100	,00	,00	,00	1,00	,00	,00
101	,00	,00	,00	,00	,00	,00
102	,00	,00	,00	,00	,00	,00
103	1,00	1,00	1,00	,00	,00	1,00
104	,00	,00	,00	,00	,00	,00
105	,00	,00	,00	,00	,00	1,00
106	,00	,00	,00	,00	,00	,00
107	,00	,00	,00	,00	,00	,00
108	,00	1,00	1,00	1,00	,00	,00
109	,00	,00	,00	,00	,00	,00
110	,00	,00	,00	,00	,00	1,00
111	,00	,00	,00	,00	,00	1,00

	Viszual.deficite	Papilloedema	Optic.atrophie	Exophthalmus	Kakosmia	Other.nerve.paresis
75	,00	,00	,00	,00	1,00	,00
76	,00	,00	,00	,00	,00	,00
77	,00	,00	,00	,00	1,00	,00
78	,00	,00	,00	,00	,00	,00
79	,00	,00	,00	,00	,00	,00
80	,00	,00	,00	,00	,00	,00
81	,00	,00	,00	,00	,00	,00
82	,00	,00	,00	,00	,00	,00
83	,00	,00	,00	,00	,00	,00
84	,00	,00	,00	,00	,00	,00
85	,00	,00	,00	,00	,00	1,00
86	,00	,00	,00	,00	,00	,00
87	,00	,00	,00	1,00	,00	,00
88	1,00	,00	,00	,00	,00	,00
89	,00	,00	,00	,00	,00	,00
90	1,00	,00	,00	,00	,00	,00
91	,00	,00	,00	,00	,00	,00
92	,00	,00	,00	,00	,00	,00
93	,00	,00	,00	,00	,00	1,00
94	,00	,00	,00	,00	,00	,00
95	,00	,00	,00	,00	,00	,00
96	,00	,00	,00	,00	,00	1,00
97	,00	,00	,00	,00	,00	,00
98	,00	,00	,00	,00	,00	,00
99	,00	,00	,00	,00	,00	,00
100	,00	,00	,00	,00	,00	,00
101	,00	,00	,00	,00	,00	,00
102	,00	,00	,00	,00	,00	,00
103	,00	,00	,00	,00	,00	,00
104	,00	,00	,00	,00	,00	,00
105	,00	,00	,00	,00	,00	,00
106	,00	,00	,00	,00	,00	,00
107	,00	,00	,00	,00	,00	,00
108	,00	,00	,00	,00	,00	,00
109	,00	,00	,00	,00	,00	1,00
110	1,00	,00	,00	,00	,00	,00
111	1,00	,00	,00	,00	,00	,00

	Sensibility.disorder	Motoric.disorder	Cerebellar.symptoms	Aphasia	Concentration.disoreders	Personallity.change
75	,00	,0	1,00	,00	,00	,00
76	,00	,0	,00	,00	,00	,00
77	,00	,0	,00	,00	,00	,00
78	,00	,0	1,00	,00	,00	,00
79	,00	,0	,00	,00	,00	,00
80	,00	,0	1,00	,00	,00	,00
81	,00	,0	,00	,00	,00	,00
82	,00	,0	,00	,00	,00	,00
83	,00	,0	,00	,00	,00	,00
84	,00	,0	,00	,00	,00	,00
85	,00	,0	1,00	,00	,00	,00
86	,00	,0	,00	,00	,00	,00
87	,00	,0	,00	,00	,00	,00
88	,00	,0	,00	,00	,00	,00
89	,00	1,0	,00	,00	,00	,00
90	,00	,0	1,00	,00	,00	,00
91	,00	,0	,00	,00	,00	,00
92	,00	1,0	1,00	,00	1,00	,00
93	,00	,0	1,00	,00	,00	,00
94	,00	,0	,00	,00	,00	,00
95	,00	,0	,00	,00	,00	,00
96	,00	,0	1,00	,00	,00	1,00
97	,00	,0	,00	,00	,00	,00
98	,00	,0	1,00	,00	,00	,00
99	,00	,0	,00	,00	,00	,00
100	,00	1,0	,00	,00	,00	,00
101	,00	,0	,00	,00	,00	,00
102	,00	,0	,00	,00	,00	,00
103	1,00	,0	,00	1,00	,00	,00
104	,00	,0	,00	,00	,00	,00
105	,00	,0	,00	,00	,00	,00
106	1,00	,0	1,00	,00	,00	,00
107	,00	,0	,00	,00	,00	,00
108	,00	,0	,00	,00	,00	,00
109	,00	,0	,00	,00	1,00	,00
110	,00	,0	,00	,00	,00	,00
111	,00	,0	,00	,00	,00	,00

	Other.motoric.disorder	loss.counsciuoness	Double.vision	Histological.clear	Histological.unclear	Size
75	,00	,00	,00	1,00	,00	1,00
76	,00	,00	,00	1,00	1,00	,00
77	,00	,00	,00	1,00	,00	1,00
78	,00	,00	1,00	1,00	,00	1,00
79	,00	,00	,00	1,00	1,00	1,00
80	,00	,00	,00	1,00	,00	1,00
81	,00	,00	,00	1,00	,00	2,00
82	,00	,00	,00	1,00	,00	1,00
83	,00	,00	,00	1,00	,00	2,00
84	,00	,00	,00	1,00	,00	2,00
85	,00	,00	,00	1,00	,00	1,00
86	,00	,00	,00	1,00	,00	2,00
87	,00	,00	,00	1,00	,00	1,00
88	,00	,00	1,00	3,00	,00	1,00
89	,00	,00	,00	1,00	1,00	2,00
90	,00	1,00	,00	1,00	,00	1,00
91	,00	,00	,00	1,00	,00	2,00
92	,00	,00	,00	1,00	,00	2,00
93	,00	,00	,00	2,00	,00	2,00
94	,00	,00	,00	1,00	,00	1,00
95	,00	,00	,00	1,00	,00	,00
96	,00	,00	,00	1,00	,00	2,00
97	,00	,00	,00	1,00	,00	,00
98	,00	,00	,00	1,00	,00	1,00
99	,00	,00	,00	1,00	,00	1,00
100	,00	,00	,00	1,00	,00	,00
101	,00	,00	,00	1,00	,00	,00
102	,00	,00	1,00	1,00	,00	1,00
103	,00	,00	,00	1,00	,00	1,00
104	,00	,00	,00	1,00	,00	1,00
105	,00	,00	,00	1,00	,00	1,00
106	,00	,00	,00	1,00	,00	1,00
107	,00	,00	,00	1,00	,00	1,00
108	,00	,00	,00	1,00	,00	1,00
109	,00	,00	,00	1,00	,00	2,00
110	,00	,00	,00	1,00	,00	1,00
111	,00	1,00	,00	1,00	,00	2,00

	MRi.CCT	Form	CSF	Edema	Masseffect	Embolisation
75	2,00	1,00	,00	,00	,00	,00
76	2,00	.	,00	1,00	,00	,00
77	2,00	1,00	,00	,00	,00	,00
78	2,00	2,00	,00	,00	1,00	,00
79	2,00	1,00	,00	,00	,00	,00
80	2,00	.	,00	,00	1,00	,00
81	2,00	1,00	,00	,00	1,00	,00
82	2,00	.	,00	,00	,00	,00
83	2,00	1,00	,00	1,00	2,00	,00
84	2,00	1,00	,00	1,00	2,00	1,00
85	2,00	.	,00	,00	1,00	,00
86	2,00	2,00	,00	,00	2,00	1,00
87	2,00	2,00	,00	,00	1,00	,00
88	2,00	2,00	,00	,00	1,00	,00
89	2,00	2,00	,00	1,00	2,00	1,00
90	2,00	1,00	,00	,00	,00	,00
91	2,00	.	,00	,00	1,00	,00
92	2,00	2,00	,00	1,00	2,00	,00
93	2,00	2,00	,00	,00	,00	,00
94	2,00	2,00	,00	,00	,00	,00
95	2,00	.	,00	,00	,00	,00
96	2,00	2,00	,00	1,00	2,00	,00
97	2,00	2,00	,00	,00	1,00	,00
98	2,00	1,00	,00	,00	,00	,00
99	2,00	2,00	,00	1,00	,00	,00
100	2,00	2,00	,00	1,00	,00	,00
101	2,00	2,00	,00	1,00	2,00	,00
102	2,00	2,00	,00	1,00	1,00	,00
103	2,00	.	,00	,00	,00	,00
104	2,00	1,00	,00	1,00	1,00	,00
105	2,00	1,00	,00	,00	1,00	,00
106	2,00	2,00	,00	1,00	2,00	,00
107	2,00	1,00	,00	,00	,00	,00
108	2,00	1,00	,00	,00	1,00	,00
109	1,00	2,00	,00	1,00	1,00	,00
110	2,00	1,00	,00	,00	1,00	,00
111	2,00	1,00	,00	,00	2,00	,00

	Hypertonia	Adipositas	Heart.disorder	Lung.disorder	Liver.disorder	Kindey.disorder
75	1,00	,00	,00	,00	,00	,00
76	1,00	,00	,00	,00	,00	,00
77	,00	1,00	,00	1,00	,00	,00
78	,00	,00	,00	,00	,00	,00
79	1,00	1,00	,00	,00	,00	,00
80	,00	,00	,00	,00	,00	,00
81	1,00	,00	1,00	,00	,00	1,00
82	1,00	,00	,00	,00	,00	,00
83	1,00	,00	1,00	,00	,00	,00
84	,00	,00	,00	,00	,00	,00
85	1,00	1,00	,00	,00	,00	,00
86	1,00	,00	,00	,00	,00	,00
87	,00	,00	,00	,00	,00	,00
88	1,00	,00	,00	,00	,00	,00
89	,00	,00	,00	,00	,00	,00
90	,00	,00	,00	1,00	,00	,00
91	1,00	,00	,00	,00	,00	,00
92	1,00	1,00	,00	,00	,00	,00
93	1,00	1,00	,00	,00	,00	,00
94	,00	1,00	,00	,00	,00	,00
95	,00	,00	,00	,00	,00	,00
96	1,00	,00	1,00	1,00	,00	,00
97	1,00	1,00	,00	,00	,00	,00
98	,00	,00	,00	,00	,00	,00
99	,00	,00	,00	,00	,00	,00
100	,00	,00	,00	,00	,00	,00
101	1,00	1,00	,00	,00	,00	,00
102	,00	,00	,00	,00	,00	,00
103	1,00	,00	,00	,00	,00	,00
104	,00	,00	,00	,00	,00	,00
105	,00	,00	,00	,00	,00	,00
106	1,00	,00	,00	,00	,00	,00
107	,00	,00	,00	,00	,00	,00
108	,00	1,00	,00	1,00	,00	,00
109	1,00	1,00	1,00	,00	,00	,00
110	1,00	,00	,00	,00	,00	,00
111	,00	,00	,00	,00	,00	,00

	Diabetes	Varicosis	ASA	Simpson.grade	OP.duration	intraOP.brain swelling
75	,00	,00	2,00	1,00	163,00	,00
76	,00	,00	2,00	1,00	162,00	,00
77	,00	,00	3,00	1,00	274,00	,00
78	,00	,00	2,00	4,00	174,00	,00
79	,00	,00	2,00	1,00	181,00	,00
80	,00	,00	2,00	1,00	181,00	,00
81	,00	,00	2,00	2,00	375,00	,00
82	,00	,00	2,00	1,00	358,00	,00
83	,00	,00	3,00	1,00	245,00	,00
84	,00	,00	2,00	1,00	211,00	1,00
85	,00	,00	2,00	4,00	250,00	,00
86	1,00	,00	3,00	2,00	255,00	,00
87	,00	,00	1,00	2,00	338,00	,00
88	,00	,00	2,00	1,00	256,00	,00
89	,00	,00	2,00	2,00	260,00	,00
90	,00	,00	2,00	1,00	196,00	,00
91	,00	,00	3,00	1,00	302,00	,00
92	1,00	,00	2,00	2,00	410,00	,00
93	1,00	,00	3,00	4,00	295,00	,00
94	,00	,00	3,00	1,00	253,00	,00
95	,00	,00	2,00	1,00	125,00	,00
96	,00	,00	3,00	1,00	169,00	,00
97	,00	,00	3,00	4,00	437,00	,00
98	,00	,00	2,00	1,00	167,00	,00
99	,00	,00	2,00	1,00	304,00	,00
100	,00	,00	3,00	2,00	367,00	,00
101	,00	,00	3,00	1,00	396,00	,00
102	,00	,00	1,00	4,00	360,00	,00
103	,00	,00	2,00	1,00	184,00	,00
104	,00	,00	2,00	1,00	225,00	,00
105	,00	,00	2,00	2,00	269,00	,00
106	,00	,00	2,00	4,00	500,00	,00
107	,00	,00	2,00	1,00	128,00	,00
108	,00	,00	2,00	1,00	171,00	,00
109	1,00	,00	3,00	1,00	432,00	,00
110	,00	,00	3,00	4,00	392,00	,00
111	,00	,00	2,00	1,00	210,00	,00

	Transfusion	Use.CUSA	Craniotomy	Sinus.lesion	Bleeding	Dura.closure
75	,00	1,00	1,00	,00	,00	3,00
76	,00	,00	1,00	,00	,00	3,00
77	,00	,00	1,00	,00	,00	2,00
78	,00	,00	1,00	,00	,00	2,00
79	,00	,00	1,00	,00	,00	1,00
80	,00	,00	1,00	,00	,00	1,00
81	,00	1,00	1,00	,00	,00	3,00
82	,00	,00	1,00	,00	,00	2,00
83	,00	1,00	1,00	,00	,00	3,00
84	6,00	1,00	1,00	,00	1,00	3,00
85	,00	1,00	1,00	,00	,00	3,00
86	,00	,00	1,00	1,00	,00	2,00
87	,00	1,00	1,00	,00	,00	3,00
88	,00	,00	1,00	,00	,00	1,00
89	,00	1,00	1,00	,00	,00	2,00
90	,00	1,00	1,00	,00	,00	1,00
91	,00	1,00	1,00	,00	,00	3,00
92	,00	1,00	1,00	,00	,00	3,00
93	,00	,00	1,00	,00	,00	3,00
94	,00	,00	1,00	,00	,00	3,00
95	,00	,00	2,00	,00	,00	2,00
96	,00	,00	1,00	,00	,00	2,00
97	,00	1,00	1,00	,00	,00	1,00
98	,00	,00	1,00	,00	,00	2,00
99	,00	,00	1,00	,00	,00	2,00
100	,00	,00	2,00	1,00	,00	3,00
101	,00	,00	2,00	1,00	,00	3,00
102	,00	1,00	1,00	,00	,00	1,00
103	,00	,00	1,00	,00	,00	3,00
104	,00	,00	1,00	1,00	,00	2,00
105	,00	1,00	1,00	,00	,00	1,00
106	,00	1,00	1,00	,00	,00	2,00
107	,00	,00	1,00	,00	,00	2,00
108	,00	,00	1,00	,00	,00	2,00
109	,00	1,00	1,00	,00	,00	1,00
110	,00	1,00	1,00	,00	,00	1,00
111	,00	,00	1,00	,00	,00	3,00

	Tachosil.Fibrin	Transfusion.postOP	Seizure.therapy	Antibiotics.postOP	CSF.circulatory.disorder	Edema.postOP
75	1,00	,00	,00	,00	,00	,00
76	1,00	,00	,00	,00	,00	,00
77	1,00	,00	,00	,00	,00	,00
78	1,00	,00	2,00	,00	,00	,00
79	1,00	,00	,00	,00	,00	,00
80	1,00	,00	,00	,00	,00	,00
81	?	?	?	?	?	?
82	?	?	?	?	?	?
83	?	?	?	?	?	?
84	?	?	?	?	?	?
85	?	?	?	?	?	?
86	?	?	?	?	?	?
87	?	?	?	?	?	?
88	?	?	?	?	?	?
89	?	?	?	?	?	?
90	?	?	?	?	?	?
91	?	?	?	?	?	?
92	?	?	?	?	?	?
93	?	?	?	?	?	?
94	?	?	?	?	?	?
95	?	?	?	?	?	?
96	?	?	?	?	?	?
97	?	?	?	?	?	?
98	?	?	?	?	?	?
99	?	?	?	?	?	?
100	?	?	?	?	?	?
101	1,00	,00	,00	,00	,00	,00
102	1,00	,00	,00	,00	,00	,00
103	1,00	,00	,00	,00	,00	,00
104	1,00	,00	,00	,00	,00	,00
105	1,00	,00	,00	,00	,00	,00
106	1,00	,00	,00	,00	,00	,00
107	1,00	,00	2,00	,00	,00	1,00
108	1,00	,00	,00	,00	,00	,00
109	1,00	,00	,00	,00	,00	1,00
110	1,00	,00	,00	,00	,00	,00
111	1,00	,00	,00	,00	,00	,00

	Kind.of.bleeding	Infection	Revision1	Revision2	Revision3	Seizures.postOP
75	,00	,00	,00	,00	,00	,00
76	,00	,00	,00	,00	,00	,00
77	,00	,00	,00	,00	,00	,00
78	,00	,00	1,00	,00	,00	,00
79	,00	,00	,00	,00	,00	,00
80	,00	,00	,00	,00	,00	,00
81	2,00	,00	3,00	,00	?	?
82	,00	,00	,00	,00	?	?
83	4,00	1,00	2,00	,00	?	?
84	,00	,00	,00	,00	?	?
85	,00	,00	,00	,00	?	?
86	,00	,00	,00	,00	?	?
87	,00	,00	,00	,00	?	?
88	,00	,00	,00	,00	?	?
89	,00	,00	,00	,00	?	?
90	,00	,00	,00	,00	?	?
91	2,00	,00	,00	,00	?	?
92	,00	,00	,00	,00	?	?
93	,00	,00	,00	,00	?	?
94	,00	,00	,00	,00	?	?
95	,00	,00	,00	,00	?	?
96	1,00	,00	3,00	3,00	?	?
97	,00	,00	2,00	,00	?	?
98	,00	,00	,00	,00	?	?
99	,00	,00	,00	,00	?	?
100	,00	,00	,00	,00	?	?
101	,00	,00	,00	,00	?	?
102	,00	,00	,00	,00	?	?
103	,00	,00	,00	,00	?	?
104	,00	,00	,00	,00	?	?
105	,00	,00	,00	,00	?	?
106	,00	,00	,00	,00	?	?
107	1,00	,00	,00	,00	?	?
108	,00	,00	,00	,00	?	?
109	,00	,00	,00	,00	?	?
110	,00	,00	,00	,00	?	?
111	1,00	,00	,00	,00	?	?

	Thro.Emb	D.insidipus	Dys.Aphasia	Sens.Hemi	Motor.Hemi	Other.symptoms
75	,00	,00	,00	,00	,00	,00
76	,00	,00	,00	,00	,00	,00
77	,00	,00	,00	,00	,00	,00
78	,00	,00	,00	,00	,00	1,00
79	,00	,00	,00	,00	,00	,00
80	,00	,00	,00	,00	,00	,00
81	,00	,00	,00	,00	1,00	1,00
82	,00	,00	1,00	,00	,00	,00
83	,00	,00	,00	,00	,00	1,00
84	,00	,00	1,00	,00	1,00	,00
85	,00	,00	,00	,00	,00	1,00
86	,00	,00	,00	,00	,00	,00
87	,00	,00	,00	,00	,00	,00
88	,00	,00	,00	,00	,00	1,00
89	,00	,00	,00	,00	,00	,00
90	,00	,00	,00	,00	,00	,00
91	,00	,00	,00	,00	1,00	1,00
92	,00	,00	,00	,00	,00	,00
93	,00	,00	,00	,00	1,00	1,00
94	,00	,00	,00	,00	,00	,00
95	,00	,00	,00	,00	,00	,00
96	,00	,00	,00	,00	,00	,00
97	,00	,00	1,00	,00	1,00	1,00
98	,00	,00	,00	,00	,00	,00
99	,00	,00	,00	,00	,00	1,00
100	,00	,00	,00	,00	,00	1,00
101	1,00	,00	,00	,00	,00	1,00
102	,00	,00	,00	,00	,00	,00
103	,00	,00	,00	,00	,00	,00
104	,00	,00	,00	,00	,00	,00
105	,00	,00	,00	,00	,00	,00
106	,00	,00	,00	,00	,00	,00
107	,00	,00	1,00	,00	,00	,00
108	,00	,00	,00	,00	,00	,00
109	,00	,00	,00	,00	,00	1,00
110	,00	,00	,00	,00	,00	1,00
111	,00	,00	,00	,00	,00	,00

	ICU.stay	NCH.stay	Total.duration	Rehabilitation	Radiation	Recurrence
75	1,00	7,00	8,00	1,00	,00	,00
76	1,00	5,00	6,00	,00	,00	,00
77	1,00	5,00	6,00	,00	,00	,00
78	2,00	9,00	11,00	1,00	,00	,00
79	1,00	5,00	6,00	1,00	,00	,00
80	1,00	6,00	7,00	1,00	,00	,00
81	24,00	3,00	27,00	2,00	,00	,00
82	1,00	7,00	8,00	1,00	,00	,00
83	17,00	5,00	22,00	3,00	,00	,00
84	7,00	12,00	19,00	1,00	,00	1,00
85	1,00	12,00	13,00	1,00	,00	,00
86	2,00	12,00	14,00	1,00	,00	,00
87	1,00	8,00	9,00	1,00	,00	,00
88	1,00	12,00	13,00	,00	,00	,00
89	1,00	10,00	11,00	1,00	,00	,00
90	1,00	5,00	6,00	1,00	,00	,00
91	19,00	7,00	26,00	2,00	,00	,00
92	1,00	9,00	10,00	1,00	,00	,00
93	2,00	19,00	21,00	3,00	,00	,00
94	1,00	6,00	7,00	1,00	,00	,00
95	1,00	7,00	8,00	1,00	,00	,00
96	4,00	8,00	12,00	1,00	1,00	,00
97	3,00	11,00	14,00	1,00	,00	,00
98	1,00	4,00	5,00	1,00	,00	,00
99	1,00	6,00	7,00	1,00	,00	,00
100	32,00	14,00	46,00	2,00	,00	,00
101	5,00	11,00	16,00	3,00	,00	,00
102	1,00	9,00	10,00	1,00	,00	,00
103	1,00	5,00	6,00	,00	,00	,00
104	1,00	7,00	8,00	,00	,00	,00
105	1,00	8,00	9,00	1,00	,00	,00
106	3,00	22,00	25,00	3,00	,00	,00
107	1,00	5,00	6,00	,00	,00	,00
108	1,00	6,00	7,00	1,00	,00	,00
109	2,00	14,00	16,00	1,00	,00	,00
110	1,00	8,00	9,00	1,00	,00	,00
111	1,00	10,00	11,00	1,00	,00	,00

	Recurrence1	Daeth	Karnofsky.score.pre	Karnofsky.score.post1.3.month	Karnofsky.score.post6.12.month	Difference.K3.K1
75	,00	,00	70,00	70,00	70,00	,00
76	1,00	,00	80,00	70,00	80,00	,00
77	,00	,00	60,00	60,00	60,00	,00
78	,00	,00	80,00	70,00	80,00	,00
79	,00	,00	80,00	70,00	70,00	-10,00
80	,00	,00	80,00	80,00	70,00	-10,00
81	,00	,00	60,00	40,00	50,00	-10,00
82	,00	,00	80,00	70,00	.	.
83	,00	,00	60,00	50,00	.	.
84	,00	,00	70,00	50,00	70,00	,00
85	,00	,00	70,00	60,00	70,00	,00
86	,00	,00	70,00	60,00	70,00	,00
87	,00	,00	80,00	70,00	90,00	10,00
88	1,00	,00	80,00	70,00	80,00	,00
89	,00	,00	80,00	70,00	80,00	,00
90	,00	,00	70,00	70,00	70,00	,00
91	,00	,00	70,00	40,00	50,00	-20,00
92	,00	,00	50,00	60,00	80,00	30,00
93	1,00	,00	50,00	50,00	.	.
94	1,00	,00	90,00	80,00	80,00	-10,00
95	,00	,00	80,00	70,00	70,00	-10,00
96	,00	,00	50,00	50,00	70,00	20,00
97	,00	,00	80,00	60,00	70,00	-10,00
98	,00	,00	90,00	90,00	100,00	10,00
99	,00	,00	80,00	90,00	90,00	10,00
100	1,00	,00	70,00	40,00	.	.
101	1,00	,00	80,00	60,00	70,00	-10,00
102	,00	,00	80,00	70,00	70,00	-10,00
103	,00	,00	70,00	80,00	80,00	10,00
104	,00	,00	90,00	80,00	90,00	,00
105	,00	,00	70,00	70,00	70,00	,00
106	,00	,00	70,00	70,00	.	.
107	,00	,00	90,00	70,00	70,00	-20,00
108	,00	,00	70,00	80,00	80,00	10,00
109	,00	,00	70,00	70,00	80,00	10,00
110	,00	,00	80,00	70,00	.	.
111	,00	,00	80,00	70,00	80,00	,00

	Diffenrence.K3.K2	K2K1	Agegroup1	Agegroup2
75	,00	,00	6,00	4,00
76	10,00	-10,00	6,00	4,00
77	.	.	5,00	3,00
78	10,00	-10,00	4,00	3,00
79	,00	-10,00	6,00	4,00
80	-10,00	,00	5,00	3,00
81	?	?	?	?
82	?	?	?	?
83	?	?	?	?
84	?	?	?	?
85	?	?	?	?
86	?	?	?	?
87	?	?	?	?
88	?	?	?	?
89	?	?	?	?
90	?	?	?	?
91	?	?	?	?
92	?	?	?	?
93	?	?	?	?
94	?	?	?	?
95	?	?	?	?
96	?	?	?	?
97	?	?	?	?
98	?	?	?	?
99	?	?	?	?
100	?	?	?	?
101	?	?	?	?
102	?	?	?	?
103	?	?	?	?
104	?	?	?	?
105	?	?	?	?
106	?	?	?	?
107	?	?	?	?
108	?	?	?	?
109	10,00	,00	6,00	4,00
110	.	-10,00	5,00	3,00
111	10,00	-10,00	6,00	4,00

	Symptoms.duration	Number.tumors	Number.symptoms
75	4,00	1,00	5,00
76	1,00	1,00	,00
77	1,00	2,00	1,00
78	2,00	,00	3,00
79	6,00	2,00	,00
80	3,00	,00	2,00
81	1,00	3,00	,00
82	1,00	1,00	,00
83	1,00	2,00	1,00
84	3,00	,00	1,00
85	6,00	2,00	2,00
86	6,00	2,00	1,00
87	3,00	,00	2,00
88	1,00	1,00	2,00
89	1,00	,00	2,00
90	1,00	1,00	4,00
91	1,00	1,00	,00
92	1,00	3,00	4,00
93	2,00	3,00	5,00
94	1,00	1,00	,00
95	1,00	,00	,00
96	1,00	3,00	3,00
97	2,00	2,00	1,00
98	2,00	,00	1,00
99	1,00	,00	10,00
100	1,00	,00	2,00
101	1,00	2,00	,00
102	3,00	,00	1,00
103	6,00	1,00	6,00
104	1,00	,00	,00
105	1,00	,00	1,00
106	4,00	1,00	2,00
107	1,00	,00	,00
108	6,00	2,00	3,00
109	5,00	4,00	2,00
110	6,00	1,00	2,00
111	2,00	,00	3,00

	Operation.time	Volume.transfosion
75	2,00	,00
76	2,00	,00
77	3,00	,00
78	2,00	,00
79	2,00	,00
80	2,00	,00
81	4,00	,00
82	3,00	,00
83	3,00	,00
84	2,00	1698,00
85	3,00	,00
86	3,00	,00
87	3,00	,00
88	3,00	,00
89	3,00	,00
90	2,00	,00
91	3,00	,00
92	4,00	,00
93	3,00	,00
94	3,00	,00
95	2,00	,00
96	2,00	,00
97	4,00	,00
98	2,00	,00
99	3,00	,00
100	4,00	,00
101	4,00	,00
102	3,00	,00
103	2,00	,00
104	2,00	,00
105	3,00	,00
106	5,00	,00
107	2,00	,00
108	2,00	,00
109	4,00	,00
110	4,00	,00
111	2,00	,00

	Volume.transfusion.postOP	Rebleeding	ICU.stay.groups
75	,00	,00	1,00
76	,00	,00	1,00
77	,00	,00	1,00
78	,00	,00	1,00
79	,00	,00	1,00
80	,00	,00	1,00
81	,00	1,00	5,00
82	,00	,00	1,00
83	283,00	1,00	4,00
84	,00	,00	3,00
85	,00	,00	1,00
86	,00	,00	1,00
87	,00	,00	1,00
88	,00	,00	1,00
89	,00	,00	1,00
90	,00	,00	1,00
91	,00	1,00	4,00
92	,00	,00	1,00
93	,00	,00	1,00
94	,00	,00	1,00
95	,00	,00	1,00
96	,00	1,00	2,00
97	,00	,00	2,00
98	,00	,00	1,00
99	,00	,00	1,00
100	566,00	,00	5,00
101	,00	,00	2,00
102	,00	,00	1,00
103	,00	,00	1,00
104	,00	,00	1,00
105	,00	,00	1,00
106	,00	,00	2,00
107	,00	1,00	1,00
108	,00	,00	1,00
109	,00	,00	1,00
110	,00	,00	1,00
111	,00	1,00	1,00

	NCH.stay.groups	Number.symptoms.postOP	Symptoms.postOP	First.symptome.groups
75	1,00	,00	,00	7,00
76	1,00	,00	,00	,00
77	1,00	,00	,00	,00
78	2,00	1,00	1,00	1,00
79	1,00	,00	,00	9,00
80	1,00	,00	,00	7,00
81	1,00	2,00	1,00	,00
82	1,00	2,00	1,00	,00
83	1,00	1,00	1,00	2,00
84	2,00	2,00	1,00	1,00
85	2,00	1,00	1,00	6,00
86	2,00	,00	,00	2,00
87	2,00	,00	,00	4,00
88	2,00	1,00	1,00	4,00
89	2,00	,00	,00	2,00
90	1,00	,00	,00	7,00
91	1,00	2,00	1,00	,00
92	2,00	,00	,00	1,00
93	3,00	3,00	1,00	7,00
94	1,00	,00	,00	,00
95	1,00	,00	,00	,00
96	2,00	,00	,00	3,00
97	2,00	3,00	1,00	4,00
98	1,00	,00	,00	7,00
99	1,00	1,00	1,00	2,00
100	2,00	2,00	1,00	5,00
101	2,00	2,00	1,00	,00
102	2,00	,00	,00	4,00
103	1,00	,00	,00	5,00
104	1,00	,00	,00	,00
105	2,00	,00	,00	4,00
106	4,00	,00	,00	7,00
107	1,00	2,00	1,00	,00
108	1,00	,00	,00	1,00
109	2,00	1,00	1,00	3,00
110	2,00	1,00	1,00	4,00
111	2,00	,00	,00	4,00

	Neurological.disorder	Histology.groups	Revision.groups	Localisation.revision
75	1,00	1,00	,00	1,00
76	,00	4,00	,00	1,00
77	1,00	1,00	,00	1,00
78	1,00	1,00	1,00	1,00
79	,00	5,00	,00	1,00
80	1,00	5,00	,00	1,00
81	,00	1,00	1,00	2,00
82	,00	5,00	,00	1,00
83	1,00	5,00	1,00	1,00
84	1,00	5,00	,00	1,00
85	1,00	5,00	,00	1,00
86	1,00	1,00	,00	1,00
87	1,00	1,00	,00	1,00
88	1,00	1,00	,00	2,00
89	1,00	1,00	,00	1,00
90	1,00	2,00	,00	1,00
91	,00	1,00	,00	2,00
92	1,00	2,00	,00	1,00
93	1,00	4,00	,00	2,00
94	,00	1,00	,00	1,00
95	,00	1,00	,00	1,00
96	1,00	5,00	1,00	1,00
97	1,00	1,00	1,00	1,00
98	1,00	1,00	,00	1,00
99	1,00	5,00	,00	2,00
100	1,00	4,00	,00	2,00
101	,00	1,00	1,00	2,00
102	1,00	1,00	,00	2,00
103	1,00	2,00	,00	1,00
104	,00	1,00	,00	1,00
105	1,00	1,00	,00	1,00
106	1,00	1,00	,00	1,00
107	,00	1,00	,00	1,00
108	1,00	2,00	,00	1,00
109	1,00	1,00	,00	2,00
110	1,00	1,00	,00	2,00
111	1,00	3,00	,00	1,00

	Masseffect.revision	Simpson.revision	antiepileptc.therapy.revision
75	,00	1,00	,00
76	,00	1,00	,00
77	,00	1,00	,00
78	1,00	4,00	1,00
79	,00	1,00	,00
80	1,00	1,00	,00
81	?	?	?
82	?	?	?
83	?	?	?
84	?	?	?
85	?	?	?
86	?	?	?
87	?	?	?
88	?	?	?
89	?	?	?
90	?	?	?
91	?	?	?
92	?	?	?
93	?	?	?
94	?	?	?
95	?	?	?
96	?	?	?
97	?	?	?
98	?	?	?
99	?	?	?
100	?	?	?
101	?	?	?
102	?	?	?
103	?	?	?
104	?	?	?
105	?	?	?
106	?	?	?
107	?	?	?
108	?	?	?
109	?	?	?
110	?	?	?
111	?	?	?

	Post.revision.symptoms	Recurrence.revision	ASA.class.4	Kd_disorder
75	,00	,00	2,00	,00
76	,00	1,00	2,00	,00
77	,00	,00	3,00	,00
78	,00	,00	2,00	,00
79	,00	,00	2,00	1,00
80	,00	,00	2,00	1,00
81	?	?	?	?
82	?	?	?	?
83	?	?	?	?
84	?	?	?	?
85	?	?	?	?
86	?	?	?	?
87	?	?	?	?
88	?	?	?	?
89	?	?	?	?
90	,00	,00	2,00	,00
91	1,00	,00	3,00	1,00
92	,00	1,00	2,00	,00
93	1,00	,00	3,00	.
94	,00	,00	3,00	1,00
95	,00	1,00	2,00	1,00
96	,00	,00	3,00	,00
97	1,00	,00	3,00	1,00
98	,00	,00	2,00	,00
99	,00	,00	2,00	,00
100	,00	,00	3,00	.
101	,00	,00	3,00	1,00
102	,00	,00	1,00	1,00
103	,00	,00	2,00	,00
104	,00	,00	2,00	,00
105	,00	,00	2,00	,00
106	,00	,00	2,00	.
107	1,00	,00	2,00	1,00
108	,00	,00	2,00	,00
109	,00	,00	3,00	,00
110	,00	,00	3,00	.
111	,00	,00	2,00	,00

	age_disorder	K1_cut	K1_3gr	Kd_3gr	ASA_di
75	1,00	,00	2,00	2,00	,00
76	1,00	,00	3,00	2,00	,00
77	,00	1,00	1,00	2,00	1,00
78	,00	,00	3,00	2,00	,00
79	1,00	,00	3,00	1,00	,00
80	,00	,00	3,00	1,00	,00
81	1,00	1,00	1,00	1,00	,00
82	,00	,00	3,00	.	,00
83	1,00	1,00	1,00	.	1,00
84	,00	,00	2,00	2,00	,00
85	1,00	,00	2,00	2,00	,00
86	1,00	,00	2,00	2,00	1,00
87	,00	,00	3,00	3,00	,00
88	1,00	,00	3,00	2,00	,00
89	1,00	,00	3,00	2,00	,00
90	,00	,00	2,00	2,00	,00
91	1,00	,00	2,00	1,00	1,00
92	,00	1,00	1,00	3,00	,00
93	1,00	1,00	1,00	.	1,00
94	1,00	,00	3,00	1,00	1,00
95	,00	,00	3,00	1,00	,00
96	1,00	1,00	1,00	3,00	1,00
97	1,00	,00	3,00	1,00	1,00
98	,00	,00	3,00	3,00	,00
99	,00	,00	3,00	3,00	,00
100	,00	,00	2,00	.	1,00
101	1,00	,00	3,00	1,00	1,00
102	1,00	,00	3,00	1,00	,00
103	,00	,00	2,00	3,00	,00
104	1,00	,00	3,00	2,00	,00
105	,00	,00	2,00	2,00	,00
106	1,00	,00	2,00	.	,00
107	1,00	,00	3,00	1,00	,00
108	,00	,00	2,00	3,00	,00
109	1,00	,00	2,00	3,00	1,00
110	,00	,00	3,00	.	1,00
111	1,00	,00	3,00	2,00	,00

	Simpson_2gr	WHO_di	Age_cut	Localisation_di
75	,00	,00	,00	1,00
76	,00	1,00	1,00	1,00
77	,00	,00	,00	1,00
78	1,00	,00	,00	1,00
79	,00	,00	,00	1,00
80	,00	,00	,00	,00
81	,00	,00	1,00	1,00
82	,00	,00	,00	1,00
83	,00	,00	1,00	1,00
84	,00	,00	,00	1,00
85	1,00	,00	1,00	1,00
86	,00	,00	1,00	,00
87	,00	,00	,00	1,00
88	,00	,00	1,00	1,00
89	,00	,00	1,00	,00
90	,00	,00	,00	.
91	,00	,00	1,00	1,00
92	,00	,00	,00	1,00
93	1,00	1,00	1,00	1,00
94	,00	,00	,00	1,00
95	,00	,00	,00	.
96	,00	1,00	1,00	,00
97	1,00	,00	,00	1,00
98	,00	,00	,00	,00
99	,00	,00	,00	1,00
100	,00	1,00	,00	1,00
101	,00	,00	1,00	1,00
102	1,00	,00	1,00	1,00
103	,00	,00	,00	1,00
104	,00	,00	1,00	,00
105	,00	,00	,00	1,00
106	1,00	,00	1,00	1,00
107	,00	,00	1,00	,00
108	,00	,00	,00	,00
109	,00	,00	,00	1,00
110	1,00	,00	,00	1,00
111	,00	,00	,00	1,00

	K3_3gr	Age3gr	RF_r	Reha_r	K3_cut
75	2,00	2,00	1,00	1,00	1,00
76	3,00	2,00	1,00	,00	,00
77	1,00	2,00	1,00	,00	1,00
78	3,00	1,00	,00	1,00	,00
79	2,00	2,00	1,00	1,00	1,00
80	2,00	2,00	,00	1,00	1,00
81	1,00	3,00	1,00	2,00	1,00
82	.	2,00	1,00	1,00	.
83	.	3,00	1,00	2,00	.
84	2,00	1,00	,00	1,00	1,00
85	2,00	2,00	1,00	1,00	1,00
86	2,00	3,00	1,00	1,00	1,00
87	3,00	1,00	,00	1,00	,00
88	3,00	2,00	1,00	,00	,00
89	3,00	2,00	,00	1,00	,00
90	2,00	1,00	1,00	1,00	1,00
91	1,00	3,00	1,00	2,00	1,00
92	3,00	2,00	1,00	1,00	,00
93	.	3,00	1,00	2,00	.
94	3,00	2,00	1,00	1,00	,00
95	2,00	1,00	,00	1,00	1,00
96	2,00	3,00	1,00	1,00	1,00
97	2,00	2,00	1,00	1,00	1,00
98	3,00	1,00	,00	1,00	,00
99	3,00	1,00	,00	1,00	,00
100	.	2,00	,00	2,00	.
101	2,00	3,00	1,00	2,00	1,00
102	2,00	3,00	,00	1,00	1,00
103	3,00	1,00	1,00	,00	,00
104	3,00	2,00	,00	,00	,00
105	2,00	1,00	,00	1,00	1,00
106	.	3,00	1,00	2,00	.
107	2,00	2,00	,00	,00	1,00
108	3,00	1,00	1,00	1,00	,00
109	3,00	2,00	1,00	1,00	,00
110	.	2,00	1,00	1,00	.
111	3,00	2,00	,00	1,00	,00

	Localisation3gr	localisation2gr	Uni_di	age7groups
75	1,00	1,00	,00	5,00
76	1,00	1,00	,00	5,00
77	1,00	1,00	,00	4,00
78	1,00	1,00	,00	3,00
79	1,00	1,00	,00	5,00
80	3,00	,00	,00	4,00
81	2,00	.	1,00	6,00
82	1,00	1,00	,00	4,00
83	1,00	1,00	1,00	7,00
84	2,00	.	1,00	3,00
85	1,00	1,00	1,00	5,00
86	3,00	,00	1,00	6,00
87	1,00	1,00	,00	3,00
88	1,00	1,00	1,00	5,00
89	3,00	,00	,00	5,00
90	.	.	,00	2,00
91	1,00	1,00	1,00	6,00
92	2,00	.	,00	4,00
93	2,00	.	1,00	6,00
94	1,00	1,00	,00	5,00
95	.	.	,00	3,00
96	3,00	,00	1,00	6,00
97	1,00	1,00	1,00	5,00
98	3,00	,00	,00	1,00
99	1,00	1,00	,00	2,00
100	2,00	.	1,00	4,00
101	2,00	.	1,00	6,00
102	1,00	1,00	,00	6,00
103	2,00	.	,00	3,00
104	3,00	,00	,00	5,00
105	1,00	1,00	,00	3,00
106	2,00	.	1,00	6,00
107	3,00	,00	,00	5,00
108	3,00	,00	,00	3,00
109	1,00	1,00	1,00	5,00
110	1,00	1,00	,00	4,00
111	1,00	1,00	,00	5,00

	Number	Sex	OP.year	Age	Histology.WHO	Type.histology
112	112,00	1,00	2009,00	50,00	3,00	15,00
113	113,00	1,00	2012,00	77,00	1,00	1,00
114	114,00	1,00	2011,00	72,00	1,00	3,00
115	115,00	1,00	2011,00	56,00	1,00	1,00
116	116,00	,00	2010,00	74,00	2,00	12,00
117	117,00	1,00	2009,00	49,00	1,00	1,00
118	118,00	1,00	2010,00	75,00	1,00	1,00
119	119,00	1,00	2009,00	58,00	1,00	2,00
120	120,00	1,00	2010,00	69,00	1,00	1,00
121	121,00	1,00	2011,00	39,00	1,00	2,00
122	122,00	1,00	2011,00	70,00	1,00	3,00
123	123,00	1,00	2011,00	71,00	2,00	12,00
124	124,00	1,00	2011,00	56,00	2,00	12,00
125	125,00	1,00	2010,00	52,00	1,00	2,00
126	126,00	,00	2011,00	77,00	2,00	12,00
127	127,00	1,00	2011,00	64,00	1,00	1,00
128	128,00	1,00	2011,00	54,00	1,00	2,00
129	129,00	1,00	2011,00	57,00	1,00	1,00
130	130,00	1,00	2011,00	65,00	1,00	1,00
131	131,00	1,00	2011,00	49,00	2,00	12,00
132	132,00	,00	2011,00	67,00	1,00	5,00
133	133,00	1,00	2011,00	55,00	1,00	2,00
134	134,00	,00	2011,00	78,00	1,00	2,00
135	135,00	1,00	2011,00	72,00	1,00	1,00
136	136,00	1,00	2011,00	75,00	1,00	1,00
137	137,00	,00	2011,00	81,00	1,00	1,00
138	138,00	,00	2011,00	17,00	1,00	4,00
139	139,00	,00	2011,00	41,00	1,00	3,00
140	140,00	,00	2011,00	75,00	2,00	10,00
141	141,00	1,00	2011,00	89,00	1,00	7,00
142	142,00	,00	2011,00	60,00	1,00	2,00
143	143,00	1,00	2011,00	36,00	1,00	5,00
144	144,00	1,00	2011,00	25,00	1,00	1,00
145	145,00	,00	2011,00	51,00	1,00	1,00
146	146,00	1,00	2011,00	67,00	1,00	1,00
147	147,00	1,00	2011,00	49,00	1,00	1,00
148	148,00	1,00	2011,00	70,00	1,00	7,00

	Side	Localisation	Duration.of.symptoms	No.symptoms	First.symptom	Haedache
112	2,00	1,00	6,00	,00	17,00	1,00
113	3,00	5,00	24,00	,00	15,00	,00
114	2,00	12,00	2,00	,00	15,00	,00
115	1,00	1,00	13,00	,00	13,00	,00
116	2,00	1,00	60,00	,00	1,00	1,00
117	1,00	3,00	6,00	1,00	1,00	1,00
118	2,00	3,00	1,00	,00	6,00	,00
119	3,00	5,00	3,00	,00	11,00	1,00
120	1,00	2,00	60,00	,00	15,00	,00
121	1,00	11,00	1,00	,00	?	?
122	1,00	1,00	,00	1,00	?	?
123	2,00	2,00	,00	1,00	?	?
124	1,00	6,00	18,00	1,00	?	?
125	2,00	6,00	11,00	1,00	?	?
126	1,00	12,00	12,00	1,00	?	?
127	3,00	5,00	79,00	,00	?	?
128	2,00	11,00	2,00	,00	?	?
129	2,00	3,00	2,00	,00	?	?
130	2,00	3,00	5,00	,00	?	?
131	1,00	3,00	21,00	,00	?	?
132	1,00	2,00	1,00	,00	?	?
133	2,00	3,00	38,00	,00	?	?
134	3,00	3,00	,00	,00	?	?
135	2,00	11,00	24,00	,00	?	?
136	2,00	2,00	,00	1,00	?	?
137	2,00	3,00	6,00	,00	?	?
138	2,00	8,00	8,00	,00	?	?
139	3,00	3,00	1,00	1,00	?	?
140	3,00	12,00	3,00	1,00	?	?
141	1,00	2,00	,00	1,00	?	?
142	3,00	13,00	3,00	,00	?	?
143	1,00	11,00	36,00	1,00	?	?
144	3,00	3,00	3,00	,00	?	?
145	3,00	6,00	,00	1,00	?	?
146	2,00	5,00	3,00	,00	?	?
147	1,00	6,00	6,00	,00	?	?
148	2,00	3,00	7,00	1,00	?	?

	Nausea	Emesis	Nausea_Emesis	Seizures	Oculo.paresis	Viszual.paresis
112	,00	,00	,00	,00	,00	,00
113	,00	,00	,00	,00	,00	,00
114	,00	,00	,00	,00	,00	,00
115	,00	,00	,00	1,00	,00	,00
116	,00	,00	,00	,00	,00	,00
117	,00	,00	,00	,00	,00	,00
118	,00	,00	,00	,00	,00	1,00
119	,00	,00	,00	,00	,00	,00
120	,00	,00	,00	,00	,00	,00
121	1,00	,00	1,00	,00	,00	,00
122	,00	,00	,00	,00	,00	,00
123	,00	,00	,00	,00	,00	,00
124	,00	,00	,00	,00	,00	,00
125	,00	,00	,00	1,00	,00	,00
126	,00	,00	,00	,00	,00	,00
127	,00	,00	,00	,00	,00	,00
128	,00	,00	,00	,00	,00	,00
129	,00	,00	,00	,00	,00	,00
130	,00	,00	,00	,00	,00	,00
131	,00	,00	,00	,00	,00	,00
132	,00	,00	,00	1,00	,00	,00
133	,00	,00	,00	,00	,00	1,00
134	,00	,00	,00	,00	,00	1,00
135	1,00	,00	1,00	,00	,00	,00
136	,00	,00	,00	,00	,00	,00
137	,00	,00	,00	,00	,00	,00
138	,00	,00	,00	,00	,00	1,00
139	,00	,00	,00	,00	,00	1,00
140	,00	,00	,00	,00	,00	,00
141	,00	,00	,00	,00	,00	,00
142	,00	,00	,00	,00	,00	,00
143	,00	,00	,00	,00	,00	,00
144	,00	,00	,00	,00	,00	1,00
145	,00	,00	,00	,00	,00	,00
146	,00	,00	,00	,00	,00	,00
147	,00	,00	,00	,00	,00	,00
148	,00	,00	,00	,00	,00	1,00

	Viszual.deficite	Papilloedema	Optic.atrophies	Exophthalmus	Kakosmia	Other.nerve.paresis
112	,00	,00	,00	,00	,00	,00
113	,00	,00	,00	,00	,00	,00
114	,00	,00	,00	,00	,00	,00
115	,00	,00	,00	,00	,00	,00
116	,00	,00	,00	,00	,00	,00
117	,00	,00	,00	,00	,00	,00
118	,00	,00	,00	,00	,00	,00
119	,00	,00	,00	,00	1,00	1,00
120	,00	,00	,00	,00	,00	,00
121	,00	,00	,00	,00	,00	,00
122	,00	,00	,00	,00	,00	,00
123	,00	,00	,00	,00	,00	,00
124	1,00	,00	,00	,00	,00	1,00
125	,00	,00	,00	,00	,00	,00
126	,00	,00	,00	,00	,00	1,00
127	,00	,00	,00	,00	1,00	1,00
128	,00	,00	,00	,00	,00	,00
129	,00	,00	,00	,00	,00	,00
130	,00	,00	,00	,00	,00	,00
131	,00	,00	,00	,00	,00	1,00
132	,00	,00	,00	,00	,00	,00
133	1,00	,00	,00	1,00	,00	,00
134	,00	,00	,00	,00	1,00	,00
135	,00	,00	,00	,00	,00	1,00
136	,00	,00	,00	,00	,00	,00
137	,00	,00	,00	1,00	,00	,00
138	,00	1,00	,00	,00	,00	,00
139	1,00	,00	,00	,00	,00	,00
140	,00	,00	,00	,00	,00	,00
141	,00	,00	,00	,00	,00	,00
142	,00	,00	,00	,00	,00	,00
143	,00	,00	,00	,00	,00	,00
144	1,00	,00	,00	,00	,00	,00
145	,00	,00	,00	,00	,00	,00
146	,00	,00	,00	,00	,00	,00
147	,00	,00	,00	,00	,00	,00
148	,00	,00	,00	,00	,00	,00

	Sensibility.disorder	Motoric.disorder	Cerebellar.symptoms	Aphasia	Concentration.disoreders	Personallity.change
112	,00	,0	,00	,00	1,00	,00
113	,00	,0	1,00	,00	1,00	1,00
114	,00	,0	1,00	,00	,00	,00
115	1,00	,0	,00	,00	,00	,00
116	,00	,0	,00	,00	,00	,00
117	,00	,0	,00	,00	,00	,00
118	,00	,0	,00	,00	,00	,00
119	,00	,0	,00	,00	,00	,00
120	,00	,0	1,00	,00	,00	,00
121	,00	,0	1,00	,00	,00	,00
122	,00	,0	,00	,00	,00	,00
123	,00	,0	,00	,00	,00	,00
124	,00	,0	1,00	,00	,00	,00
125	,00	,0	,00	,00	,00	,00
126	,00	,0	,00	,00	,00	,00
127	,00	,0	,00	,00	,00	,00
128	,00	,0	1,00	,00	,00	,00
129	,00	,0	,00	1,00	,00	,00
130	,00	,0	,00	,00	,00	,00
131	,00	,0	1,00	,00	,00	,00
132	,00	,0	,00	,00	,00	,00
133	,00	,0	,00	,00	,00	,00
134	,00	,0	,00	,00	,00	,00
135	,00	,0	1,00	,00	,00	,00
136	,00	,0	,00	,00	,00	,00
137	,00	,0	,00	,00	,00	,00
138	,00	,0	,00	,00	1,00	,00
139	,00	,0	,00	,00	,00	,00
140	,00	,0	,00	,00	,00	,00
141	,00	,0	1,00	,00	1,00	,00
142	1,00	,0	,00	,00	,00	,00
143	,00	,0	1,00	,00	,00	,00
144	,00	,0	,00	,00	,00	,00
145	,00	,0	,00	,00	,00	,00
146	,00	,0	,00	,00	,00	,00
147	,00	,0	1,00	,00	,00	,00
148	,00	,0	,00	,00	,00	,00

	Other.motoric.disorder	loss.counsciuoness	Double.vision	Histological.clear	Histological.unclear	Size
112	,00	,00	,00	1,00	,00	1,00
113	,00	,00	,00	1,00	,00	2,00
114	,00	,00	,00	1,00	,00	2,00
115	,00	,00	,00	1,00	,00	1,00
116	,00	,00	,00	2,00	,00	1,00
117	,00	,00	,00	1,00	,00	1,00
118	,00	,00	,00	1,00	,00	1,00
119	,00	,00	,00	1,00	,00	1,00
120	,00	,00	,00	1,00	,00	1,00
121	,00	,00	,00	1,00	,00	1,00
122	,00	,00	,00	1,00	,00	2,00
123	,00	,00	,00	1,00	,00	1,00
124	,00	,00	,00	1,00	,00	2,00
125	,00	,00	,00	1,00	,00	1,00
126	,00	,00	,00	1,00	,00	1,00
127	,00	,00	,00	1,00	,00	1,00
128	,00	,00	,00	1,00	,00	1,00
129	,00	,00	,00	1,00	2,00	2,00
130	,00	,00	,00	1,00	,00	1,00
131	,00	,00	,00	1,00	,00	1,00
132	,00	,00	,00	1,00	,00	2,00
133	,00	,00	,00	1,00	,00	1,00
134	,00	,00	,00	1,00	2,00	2,00
135	,00	,00	,00	1,00	,00	2,00
136	,00	,00	1,00	1,00	,00	2,00
137	,00	,00	,00	1,00	,00	2,00
138	,00	,00	,00	1,00	,00	1,00
139	,00	1,00	,00	1,00	,00	1,00
140	,00	,00	1,00	1,00	,00	2,00
141	?	?	?	?	?	?
142	?	?	?	?	?	?
143	?	?	?	?	?	?
144	?	?	?	?	?	?
145	?	?	?	?	?	?
146	?	?	?	?	?	?
147	?	?	?	?	?	?
148	?	?	?	?	?	?

	MRi.CCT	Form	CSF	Edema	Masseffect	Embolisation
112	2,00	2,00	,00	,00	,00	,00
113	2,00	1,00	,00	1,00	,00	,00
114	2,00	1,00	,00	,00	,00	,00
115	2,00	1,00	,00	1,00	,00	,00
116	2,00	1,00	,00	,00	,00	,00
117	2,00	1,00	,00	,00	,00	,00
118	2,00	1,00	,00	,00	1,00	,00
119	2,00	1,00	,00	,00	,00	,00
120	2,00	1,00	,00	,00	,00	,00
121	2,00	2,00	,00	,00	1,00	,00
122	2,00	2,00	,00	1,00	,00	,00
123	2,00	1,00	,00	,00	,00	,00
124	2,00	2,00	,00	,00	1,00	,00
125	1,00	1,00	,00	,00	,00	,00
126	2,00	2,00	,00	,00	1,00	,00
127	2,00	2,00	,00	,00	,00	,00
128	2,00	1,00	,00	,00	1,00	,00
129	2,00	1,00	,00	1,00	2,00	1,00
130	2,00	1,00	,00	,00	,00	,00
131	2,00	1,00	,00	,00	,00	,00
132	2,00	2,00	,00	1,00	1,00	,00
133	2,00	2,00	,00	,00	1,00	,00
134	2,00	2,00	,00	,00	1,00	,00
135	2,00	2,00	1,00	1,00	1,00	,00
136	1,00	1,00	,00	,00	,00	1,00
137	2,00	2,00	,00	1,00	1,00	,00
138	2,00	1,00	,00	,00	1,00	,00
139	1,00	1,00	,00	,00	1,00	,00
140	2,00	2,00	1,00	1,00	1,00	,00
141	2,00	1,00	1,00	1,00	2,00	,00
142	2,00	1,00	,00	,00	1,00	,00
143	2,00	1,00	,00	,00	1,00	,00
144	2,00	2,00	,00	,00	1,00	,00
145	2,00	2,00	,00	,00	1,00	,00
146	2,00	1,00	,00	,00	,00	,00
147	2,00	2,00	,00	,00	1,00	,00
148	2,00	2,00	,00	,00	2,00	,00

	Hypertonia	Adipositas	Heart.disorder	Lung.disorder	Liver.disorder	Kindey.disorder
112	1,00	,00	,00	,00	,00	,00
113	1,00	,00	1,00	1,00	,00	,00
114	1,00	1,00	,00	,00	,00	,00
115	,00	,00	,00	,00	,00	,00
116	1,00	,00	,00	,00	,00	,00
117	,00	,00	,00	,00	,00	,00
118	1,00	,00	1,00	,00	,00	,00
119	1,00	,00	,00	1,00	,00	,00
120	1,00	1,00	,00	,00	,00	,00
121	,00	,00	,00	1,00	,00	,00
122	,00	,00	,00	,00	,00	,00
123	1,00	1,00	,00	1,00	,00	,00
124	1,00	,00	1,00	,00	,00	,00
125	1,00	1,00	,00	,00	,00	,00
126	,00	1,00	,00	,00	1,00	1,00
127	,00	1,00	,00	,00	,00	,00
128	,00	1,00	1,00	,00	,00	,00
129	,00	1,00	,00	,00	,00	,00
130	1,00	,00	,00	,00	,00	,00
131	,00	1,00	,00	,00	,00	,00
132	1,00	,00	1,00	,00	,00	,00
133	,00	1,00	,00	,00	,00	,00
134	1,00	1,00	1,00	,00	,00	,00
135	1,00	,00	,00	,00	,00	,00
136	1,00	1,00	1,00	,00	,00	,00
137	1,00	1,00	,00	,00	,00	,00
138	,00	,00	,00	,00	,00	,00
139	1,00	1,00	1,00	,00	,00	1,00
140	1,00	,00	,00	,00	,00	,00
141	1,00	,00	1,00	,00	,00	,00
142	1,00	,00	,00	,00	,00	,00
143	,00	,00	,00	,00	,00	,00
144	,00	,00	,00	,00	,00	,00
145	,00	1,00	,00	,00	,00	,00
146	1,00	1,00	,00	,00	,00	,00
147	,00	,00	,00	,00	,00	,00
148	1,00	1,00	,00	,00	,00	,00

	Diabetes	Varicosis	ASA	Simpson.grade	OP.duration	intraOP.brain swelling
112	,00	,00	3,00	1,00	217,00	,00
113	,00	,00	3,00	1,00	233,00	,00
114	1,00	1,00	2,00	2,00	323,00	,00
115	,00	,00	2,00	1,00	181,00	,00
116	,00	,00	2,00	4,00	265,00	,00
117	,00	,00	2,00	2,00	186,00	,00
118	1,00	,00	2,00	1,00	233,00	,00
119	,00	,00	2,00	1,00	206,00	,00
120	,00	,00	2,00	2,00	90,00	,00
121	,00	,00	?	?	?	?
122	,00	,00	?	?	?	?
123	,00	,00	?	?	?	?
124	1,00	,00	?	?	?	?
125	,00	,00	?	?	?	?
126	,00	,00	?	?	?	?
127	,00	1,00	?	?	?	?
128	,00	,00	?	?	?	?
129	,00	,00	?	?	?	?
130	,00	,00	?	?	?	?
131	,00	,00	?	?	?	?
132	1,00	,00	?	?	?	?
133	,00	,00	?	?	?	?
134	1,00	,00	?	?	?	?
135	,00	,00	?	?	?	?
136	1,00	,00	?	?	?	?
137	,00	,00	?	?	?	?
138	,00	,00	?	?	?	?
139	1,00	,00	?	?	?	?
140	,00	,00	?	?	?	?
141	,00	,00	3,00	1,00	209,00	,00
142	,00	,00	2,00	1,00	451,00	,00
143	,00	,00	1,00	4,00	372,00	,00
144	,00	,00	1,00	1,00	340,00	,00
145	,00	,00	2,00	3,00	357,00	,00
146	,00	,00	2,00	1,00	203,00	,00
147	,00	,00	2,00	2,00	163,00	,00
148	,00	,00	3,00	2,00	319,00	,00

	Transfusion	Use.CUSA	Craniotomy	Sinus.lesion	Bleeding	Dura.closure
112	,00	1,00	1,00	,00	,00	1,00
113	,00	1,00	1,00	,00	,00	2,00
114	,00	1,00	1,00	,00	,00	3,00
115	,00	,00	1,00	,00	,00	3,00
116	,00	,00	1,00	,00	,00	2,00
117	,00	,00	1,00	,00	,00	1,00
118	,00	,00	1,00	,00	,00	1,00
119	,00	,00	1,00	1,00	,00	2,00
120	,00	,00	1,00	,00	,00	2,00
121	,00	1,00	2,00	,00	,00	3,00
122	,00	1,00	1,00	,00	,00	1,00
123	,00	,00	1,00	,00	,00	2,00
124	,00	1,00	1,00	,00	,00	1,00
125	,00	,00	1,00	,00	,00	3,00
126	,00	1,00	1,00	1,00	,00	3,00
127	,00	,00	1,00	,00	,00	1,00
128	,00	1,00	1,00	,00	,00	3,00
129	,00	1,00	1,00	,00	,00	2,00
130	,00	1,00	1,00	,00	,00	1,00
131	,00	,00	1,00	,00	1,00	2,00
132	,00	,00	1,00	,00	,00	3,00
133	5,00	,00	1,00	,00	,00	2,00
134	,00	1,00	1,00	,00	,00	2,00
135	,00	1,00	1,00	,00	,00	3,00
136	6,00	,00	1,00	1,00	,00	2,00
137	,00	1,00	1,00	,00	,00	2,00
138	,00	,00	1,00	,00	,00	3,00
139	,00	,00	1,00	,00	,00	1,00
140	,00	1,00	1,00	,00	,00	3,00
141	,00	1,00	1,00	,00	,00	2,00
142	,00	1,00	1,00	,00	,00	3,00
143	,00	1,00	1,00	,00	,00	1,00
144	,00	1,00	2,00	,00	,00	1,00
145	,00	1,00	1,00	,00	,00	1,00
146	,00	1,00	1,00	,00	,00	1,00
147	,00	1,00	1,00	,00	,00	1,00
148	,00	1,00	1,00	,00	,00	2,00

	Tachosil.Fibrin	Transfusion.postOP	Seizure.therapy	Antibiotics.postOP	CSF.circulatory.disorder	Edema.postOP
112	1,00	,00	,00	,00	,00	,00
113	1,00	,00	,00	,00	,00	,00
114	1,00	,00	,00	,00	,00	,00
115	1,00	,00	2,00	,00	,00	,00
116	1,00	,00	,00	,00	,00	,00
117	1,00	,00	,00	,00	,00	,00
118	1,00	,00	,00	,00	,00	,00
119	1,00	,00	,00	,00	,00	,00
120	1,00	,00	,00	,00	,00	,00
121	1,00	,00	,00	,00	,00	,00
122	1,00	3,00	,00	,00	,00	1,00
123	1,00	,00	,00	,00	,00	,00
124	1,00	,00	,00	,00	,00	,00
125	1,00	,00	,00	,00	1,00	,00
126	1,00	,00	,00	,00	,00	,00
127	1,00	,00	2,00	,00	,00	,00
128	1,00	,00	,00	,00	,00	,00
129	1,00	,00	,00	,00	,00	1,00
130	1,00	,00	,00	,00	,00	,00
131	1,00	,00	,00	,00	,00	,00
132	1,00	,00	1,00	,00	,00	,00
133	1,00	,00	,00	,00	,00	,00
134	1,00	,00	,00	,00	,00	,00
135	1,00	,00	,00	,00	,00	1,00
136	1,00	,00	,00	,00	,00	1,00
137	1,00	,00	,00	1,00	,00	,00
138	1,00	,00	,00	,00	,00	,00
139	1,00	,00	2,00	,00	,00	,00
140	1,00	,00	,00	,00	,00	,00
141	1,00	,00	,00	,00	,00	,00
142	1,00	,00	,00	1,00	,00	,00
143	1,00	,00	,00	,00	,00	,00
144	1,00	,00	,00	,00	,00	,00
145	1,00	,00	,00	1,00	,00	,00
146	1,00	,00	,00	,00	,00	,00
147	1,00	,00	,00	,00	,00	,00
148	1,00	,00	2,00	,00	,00	,00

	Kind.of.bleeding	Infection	Revision1	Revision2	Revision3	Seizures.postOP
112	,00	,00	,00	,00	,00	,00
113	,00	,00	,00	,00	,00	,00
114	,00	,00	,00	,00	,00	,00
115	,00	,00	,00	,00	,00	1,00
116	,00	,00	,00	,00	,00	,00
117	,00	,00	,00	,00	,00	,00
118	,00	,00	,00	,00	,00	,00
119	,00	,00	,00	,00	,00	,00
120	,00	,00	,00	,00	,00	,00
121	,00	,00	2,00	,00	,00	,00
122	3,00	,00	3,00	2,00	,00	,00
123	,00	,00	,00	,00	,00	,00
124	,00	,00	,00	,00	,00	,00
125	2,00	,00	3,00	2,00	,00	,00
126	,00	,00	,00	,00	,00	,00
127	,00	,00	,00	,00	,00	,00
128	,00	,00	,00	,00	,00	,00
129	,00	,00	,00	,00	,00	,00
130	,00	,00	,00	,00	,00	,00
131	,00	,00	,00	,00	,00	,00
132	,00	,00	,00	,00	,00	,00
133	,00	,00	,00	,00	,00	,00
134	,00	,00	,00	,00	,00	,00
135	,00	,00	,00	,00	,00	,00
136	2,00	,00	,00	,00	,00	,00
137	,00	1,00	2,00	,00	,00	1,00
138	,00	,00	,00	,00	,00	,00
139	,00	,00	,00	,00	,00	,00
140	,00	,00	1,00	,00	,00	,00
141	,00	,00	,00	,00	?	?
142	,00	,00	,00	,00	?	?
143	,00	,00	,00	,00	?	?
144	,00	,00	,00	,00	?	?
145	,00	1,00	2,00	,00	?	?
146	,00	,00	,00	,00	?	?
147	,00	,00	2,00	,00	?	?
148	,00	,00	,00	,00	?	?

	Thro.Emb	D.insidipus	Dys.Aphasia	Sens.Hemi	Motor.Hemi	Other.symptoms
112	,00	,00	,00	,00	,00	,00
113	,00	,00	,00	,00	,00	,00
114	,00	,00	,00	,00	,00	,00
115	,00	,00	,00	,00	,00	,00
116	,00	,00	,00	,00	,00	,00
117	,00	,00	,00	,00	,00	,00
118	,00	,00	,00	,00	,00	1,00
119	,00	,00	,00	,00	,00	,00
120	,00	,00	,00	,00	,00	,00
121	,00	1,00	,00	,00	,00	,00
122	1,00	,00	,00	,00	,00	1,00
123	,00	,00	,00	,00	,00	,00
124	1,00	,00	,00	,00	,00	1,00
125	,00	,00	,00	,00	,00	1,00
126	,00	,00	,00	,00	,00	,00
127	,00	,00	,00	,00	,00	,00
128	,00	,00	,00	,00	,00	,00
129	,00	,00	1,00	,00	,00	,00
130	,00	,00	,00	,00	,00	,00
131	,00	,00	,00	,00	,00	1,00
132	,00	,00	,00	,00	,00	1,00
133	,00	,00	,00	,00	,00	,00
134	,00	,00	,00	,00	,00	1,00
135	,00	,00	,00	,00	1,00	,00
136	,00	,00	1,00	,00	1,00	,00
137	,00	,00	1,00	,00	,00	,00
138	,00	,00	,00	,00	,00	1,00
139	,00	,00	1,00	,00	,00	,00
140	,00	,00	,00	,00	,00	,00
141	,00	,00	,00	,00	,00	,00
142	,00	,00	,00	,00	,00	,00
143	,00	,00	,00	,00	,00	,00
144	,00	,00	,00	,00	,00	,00
145	,00	,00	,00	,00	,00	,00
146	,00	,00	,00	,00	,00	,00
147	,00	,00	,00	,00	,00	,00
148	,00	,00	1,00	,00	,00	,00

	ICU.stay	NCH.stay	Total.duration	Rehabilitation	Radiation	Recurrence
112	1,00	7,00	8,00	1,00	,00	,00
113	1,00	21,00	22,00	3,00	,00	,00
114	1,00	9,00	10,00	1,00	,00	,00
115	1,00	7,00	8,00	1,00	,00	,00
116	1,00	5,00	6,00	1,00	,00	,00
117	1,00	5,00	6,00	1,00	,00	,00
118	7,00	5,00	12,00	1,00	,00	,00
119	1,00	5,00	6,00	1,00	,00	,00
120	1,00	6,00	7,00	,00	,00	,00
121	1,00	11,00	12,00	1,00	,00	,00
122	27,00	1,00	28,00	2,00	,00	,00
123	1,00	7,00	8,00	1,00	,00	,00
124	2,00	2,00	4,00	,00	,00	,00
125	3,00	16,00	19,00	1,00	,00	,00
126	2,00	8,00	10,00	1,00	,00	,00
127	1,00	6,00	7,00	1,00	,00	,00
128	1,00	6,00	7,00	1,00	,00	,00
129	1,00	6,00	7,00	1,00	,00	,00
130	1,00	5,00	6,00	,00	,00	,00
131	1,00	6,00	7,00	,00	,00	1,00
132	1,00	5,00	6,00	1,00	,00	,00
133	1,00	6,00	7,00	1,00	,00	,00
134	25,00	3,00	28,00	2,00	,00	,00
135	5,00	4,00	9,00	1,00	,00	,00
136	3,00	11,00	14,00	3,00	,00	,00
137	7,00	10,00	17,00	1,00	1,00	,00
138	1,00	5,00	6,00	,00	,00	,00
139	2,00	8,00	10,00	1,00	,00	,00
140	3,00	13,00	16,00	1,00	1,00	,00
141	1,00	15,00	16,00	,00	,00	,00
142	1,00	9,00	10,00	1,00	,00	,00
143	1,00	6,00	7,00	1,00	,00	,00
144	1,00	7,00	8,00	,00	,00	,00
145	1,00	7,00	8,00	1,00	,00	,00
146	1,00	5,00	6,00	2,00	,00	,00
147	1,00	6,00	7,00	1,00	,00	,00
148	2,00	20,00	22,00	1,00	,00	,00

	Recurrence1	Daeth	Karnofsky.score.pre	Karnofsky.score.post1.3.month	Karnofsky.score.post6.12.month	Difference.K3.K1
112	1,00	,00	70,00	70,00	70,00	,00
113	,00	,00	70,00	60,00	.	.
114	,00	,00	60,00	70,00	80,00	20,00
115	,00	,00	70,00	70,00	80,00	10,00
116	,00	,00	70,00	40,00	50,00	-20,00
117	,00	,00	90,00	90,00	90,00	,00
118	,00	,00	70,00	70,00	70,00	,00
119	,00	,00	70,00	70,00	90,00	20,00
120	,00	,00	70,00	70,00	80,00	10,00
121	,00	,00	80,00	70,00	70,00	-10,00
122	,00	,00	80,00	60,00	70,00	-10,00
123	,00	,00	80,00	70,00	80,00	,00
124	,00	1,00	80,00	.	.	.
125	,00	,00	80,00	70,00	70,00	-10,00
126	,00	,00	70,00	70,00	70,00	,00
127	1,00	,00	70,00	70,00	70,00	,00
128	,00	,00	80,00	70,00	80,00	,00
129	,00	,00	80,00	70,00	80,00	,00
130	1,00	,00	70,00	70,00	70,00	,00
131	,00	,00	90,00	70,00	70,00	-20,00
132	,00	,00	60,00	70,00	70,00	10,00
133	,00	,00	70,00	70,00	80,00	10,00
134	,00	,00	70,00	70,00	70,00	,00
135	,00	,00	50,00	70,00	70,00	20,00
136	,00	,00	70,00	60,00	70,00	,00
137	,00	,00	70,00	60,00	70,00	,00
138	,00	,00	80,00	80,00	80,00	,00
139	,00	,00	90,00	70,00	80,00	-10,00
140	,00	,00	70,00	50,00	70,00	,00
141	,00	,00	50,00	50,00	50,00	,00
142	,00	,00	60,00	70,00	80,00	20,00
143	,00	,00	100,00	80,00	90,00	-10,00
144	,00	,00	70,00	70,00	90,00	20,00
145	,00	,00	70,00	70,00	80,00	10,00
146	,00	,00	80,00	70,00	90,00	10,00
147	,00	,00	80,00	70,00	70,00	-10,00
148	,00	,00	70,00	70,00	70,00	,00

	Diffenrence.K3.K2	K2K1	Agegroup1	Agegroup2
112	,00	,00	4,00	3,00
113	.	-10,00	7,00	4,00
114	10,00	10,00	7,00	4,00
115	10,00	,00	5,00	3,00
116	10,00	-30,00	7,00	4,00
117	,00	,00	4,00	3,00
118	,00	,00	7,00	4,00
119	20,00	,00	5,00	3,00
120	10,00	,00	6,00	4,00
121	,00	-10,00	3,00	2,00
122	10,00	-20,00	6,00	4,00
123	10,00	-10,00	7,00	4,00
124	.	.	5,00	3,00
125	,00	-10,00	5,00	3,00
126	,00	,00	7,00	4,00
127	,00	,00	6,00	4,00
128	10,00	-10,00	5,00	3,00
129	10,00	-10,00	5,00	3,00
130	,00	,00	6,00	4,00
131	,00	-20,00	4,00	3,00
132	,00	10,00	6,00	4,00
133	10,00	,00	5,00	3,00
134	,00	,00	7,00	4,00
135	,00	20,00	7,00	4,00
136	10,00	-10,00	7,00	4,00
137	10,00	-10,00	8,00	5,00
138	,00	,00	1,00	1,00
139	10,00	-20,00	4,00	3,00
140	20,00	-20,00	7,00	4,00
141	?	?	?	?
142	?	?	?	?
143	?	?	?	?
144	?	?	?	?
145	?	?	?	?
146	?	?	?	?
147	?	?	?	?
148	?	?	?	?

	Symptoms.duration	Number.tumors	Number.symptoms
112	3,00	1,00	2,00
113	5,00	3,00	3,00
114	2,00	4,00	1,00
115	5,00	,00	2,00
116	6,00	1,00	1,00
117	3,00	,00	1,00
118	1,00	3,00	1,00
119	2,00	2,00	3,00
120	6,00	2,00	1,00
121	1,00	1,00	3,00
122	1,00	,00	,00
123	1,00	3,00	,00
124	5,00	3,00	3,00
125	4,00	2,00	1,00
126	4,00	3,00	1,00
127	7,00	2,00	2,00
128	2,00	2,00	1,00
129	2,00	1,00	2,00
130	3,00	1,00	,00
131	5,00	1,00	3,00
132	1,00	3,00	1,00
133	6,00	1,00	3,00
134	1,00	4,00	2,00
135	5,00	1,00	4,00
136	1,00	4,00	1,00
137	3,00	2,00	2,00
138	4,00	,00	3,00
139	1,00	5,00	3,00
140	2,00	1,00	2,00
141	1,00	2,00	2,00
142	2,00	1,00	2,00
143	6,00	,00	2,00
144	2,00	,00	3,00
145	1,00	1,00	,00
146	2,00	2,00	,00
147	3,00	,00	2,00
148	4,00	2,00	1,00

	Operation.time	Volume.transfosion
112	2,00	,00
113	2,00	,00
114	3,00	,00
115	2,00	,00
116	3,00	,00
117	2,00	,00
118	2,00	,00
119	2,00	,00
120	1,00	,00
121	3,00	,00
122	2,00	,00
123	2,00	,00
124	5,00	,00
125	1,00	,00
126	2,00	,00
127	2,00	,00
128	2,00	,00
129	3,00	,00
130	3,00	,00
131	3,00	,00
132	2,00	,00
133	4,00	1415,00
134	4,00	,00
135	2,00	,00
136	2,00	1698,00
137	4,00	,00
138	4,00	,00
139	3,00	,00
140	2,00	,00
141	2,00	,00
142	4,00	,00
143	4,00	,00
144	3,00	,00
145	3,00	,00
146	2,00	,00
147	2,00	,00
148	3,00	,00

	Volume.transfusion.postOP	Rebleeding	ICU.stay.groups
112	,00	,00	1,00
113	,00	,00	1,00
114	,00	,00	1,00
115	,00	,00	1,00
116	,00	,00	1,00
117	,00	,00	1,00
118	,00	,00	3,00
119	,00	,00	1,00
120	,00	,00	1,00
121	,00	,00	1,00
122	849,00	1,00	5,00
123	,00	,00	1,00
124	,00	,00	1,00
125	,00	1,00	2,00
126	,00	,00	1,00
127	,00	,00	1,00
128	,00	,00	1,00
129	,00	,00	1,00
130	,00	,00	1,00
131	,00	,00	1,00
132	,00	,00	1,00
133	,00	,00	1,00
134	,00	,00	5,00
135	,00	,00	2,00
136	,00	1,00	2,00
137	,00	,00	3,00
138	,00	,00	1,00
139	,00	,00	1,00
140	,00	,00	2,00
141	,00	?	?
142	,00	?	?
143	,00	?	?
144	,00	?	?
145	,00	?	?
146	,00	?	?
147	,00	?	?
148	,00	?	?

	NCH.stay.groups	Number.symptoms.postOP	Symptoms.postOP	First.symptome.groups
112	1,00	,00	,00	3,00
113	3,00	,00	,00	7,00
114	2,00	,00	,00	7,00
115	1,00	1,00	1,00	5,00
116	1,00	,00	,00	1,00
117	1,00	,00	,00	1,00
118	1,00	1,00	1,00	4,00
119	1,00	,00	,00	6,00
120	1,00	,00	,00	7,00
121	2,00	1,00	1,00	1,00
122	1,00	2,00	1,00	,00
123	1,00	,00	,00	,00
124	1,00	2,00	1,00	6,00
125	3,00	1,00	1,00	2,00
126	2,00	,00	,00	6,00
127	1,00	,00	,00	6,00
128	1,00	,00	,00	7,00
129	1,00	1,00	1,00	8,00
130	1,00	,00	,00	9,00
131	1,00	1,00	1,00	6,00
132	1,00	1,00	1,00	2,00
133	1,00	,00	,00	4,00
134	1,00	1,00	1,00	4,00
135	1,00	1,00	1,00	7,00
136	2,00	2,00	1,00	4,00
137	2,00	2,00	1,00	1,00
138	1,00	1,00	1,00	4,00
139	2,00	1,00	1,00	4,00
140	2,00	,00	,00	1,00
141	3,00	,00	,00	3,00
142	2,00	,00	,00	5,00
143	1,00	,00	,00	7,00
144	1,00	,00	,00	4,00
145	1,00	,00	,00	,00
146	1,00	,00	,00	9,00
147	1,00	,00	,00	7,00
148	3,00	1,00	1,00	4,00

	Neurological.disorder	Histology.groups	Revision.groups	Localisation.revision
112	1,00	5,00	,00	1,00
113	1,00	1,00	,00	2,00
114	1,00	3,00	,00	1,00
115	1,00	1,00	,00	1,00
116	1,00	4,00	,00	1,00
117	1,00	1,00	,00	1,00
118	1,00	1,00	,00	1,00
119	1,00	2,00	,00	2,00
120	1,00	1,00	,00	1,00
121	1,00	2,00	1,00	1,00
122	,00	3,00	1,00	1,00
123	,00	4,00	,00	1,00
124	1,00	4,00	,00	1,00
125	1,00	2,00	1,00	1,00
126	1,00	4,00	,00	1,00
127	1,00	1,00	,00	2,00
128	1,00	2,00	,00	1,00
129	1,00	1,00	,00	1,00
130	,00	1,00	,00	1,00
131	1,00	4,00	,00	1,00
132	1,00	5,00	,00	1,00
133	1,00	2,00	,00	1,00
134	1,00	2,00	,00	2,00
135	1,00	1,00	,00	1,00
136	1,00	1,00	,00	1,00
137	1,00	1,00	1,00	1,00
138	1,00	5,00	,00	1,00
139	1,00	3,00	,00	2,00
140	1,00	5,00	1,00	2,00
141	1,00	5,00	,00	1,00
142	1,00	2,00	,00	2,00
143	1,00	5,00	,00	1,00
144	1,00	1,00	,00	2,00
145	,00	1,00	1,00	2,00
146	,00	1,00	,00	1,00
147	1,00	1,00	1,00	1,00
148	1,00	5,00	,00	1,00

	Masseffect.revision	Simpson.revision	antiepileptc.therapy.revision
112	,00	1,00	,00
113	,00	1,00	,00
114	,00	1,00	,00
115	,00	1,00	1,00
116	,00	4,00	,00
117	,00	1,00	,00
118	1,00	1,00	,00
119	,00	1,00	,00
120	,00	1,00	,00
121	?	?	?
122	?	?	?
123	?	?	?
124	?	?	?
125	?	?	?
126	?	?	?
127	?	?	?
128	?	?	?
129	?	?	?
130	?	?	?
131	?	?	?
132	?	?	?
133	?	?	?
134	?	?	?
135	?	?	?
136	?	?	?
137	?	?	?
138	?	?	?
139	?	?	?
140	?	?	?
141	?	?	?
142	?	?	?
143	?	?	?
144	?	?	?
145	?	?	?
146	?	?	?
147	?	?	?
148	?	?	?

	Post.revision.symptoms	Recurrence.revision	ASA.class.4	Kd_disorder
112	,00	1,00	3,00	,00
113	,00	,00	3,00	.
114	,00	,00	2,00	,00
115	,00	,00	2,00	,00
116	,00	,00	2,00	1,00
117	,00	,00	2,00	,00
118	,00	,00	2,00	,00
119	,00	,00	2,00	,00
120	,00	,00	2,00	,00
121	,00	,00	2,00	1,00
122	,00	,00	2,00	1,00
123	,00	,00	3,00	,00
124	,00	,00	2,00	.
125	,00	,00	2,00	1,00
126	,00	,00	2,00	,00
127	,00	,00	3,00	,00
128	,00	,00	2,00	,00
129	1,00	,00	2,00	,00
130	,00	,00	2,00	,00
131	,00	1,00	2,00	1,00
132	,00	,00	3,00	,00
133	,00	,00	2,00	,00
134	,00	,00	3,00	,00
135	1,00	,00	2,00	,00
136	1,00	,00	2,00	,00
137	1,00	,00	2,00	,00
138	,00	,00	2,00	,00
139	1,00	,00	3,00	1,00
140	,00	,00	2,00	,00
141	?	?	?	?
142	?	?	?	?
143	?	?	?	?
144	?	?	?	?
145	?	?	?	?
146	?	?	?	?
147	?	?	?	?
148	?	?	?	?

	age_disorder	K1_cut	K1_3gr	Kd_3gr	ASA_di
112	,00	,00	2,00	2,00	1,00
113	1,00	,00	2,00	.	1,00
114	1,00	1,00	1,00	3,00	,00
115	,00	,00	2,00	3,00	,00
116	1,00	,00	2,00	1,00	,00
117	,00	,00	3,00	2,00	,00
118	1,00	,00	2,00	2,00	,00
119	,00	,00	2,00	3,00	,00
120	1,00	,00	2,00	3,00	,00
121	,00	,00	3,00	1,00	,00
122	1,00	,00	3,00	1,00	,00
123	1,00	,00	3,00	2,00	1,00
124	,00	,00	3,00	.	,00
125	,00	,00	3,00	1,00	,00
126	1,00	,00	2,00	2,00	,00
127	1,00	,00	2,00	2,00	1,00
128	,00	,00	3,00	2,00	,00
129	,00	,00	3,00	2,00	,00
130	1,00	,00	2,00	2,00	,00
131	,00	,00	3,00	1,00	,00
132	1,00	1,00	1,00	3,00	1,00
133	,00	,00	2,00	3,00	,00
134	1,00	,00	2,00	2,00	1,00
135	1,00	1,00	1,00	3,00	,00
136	1,00	,00	2,00	2,00	,00
137	1,00	,00	2,00	2,00	,00
138	,00	,00	3,00	2,00	,00
139	,00	,00	3,00	1,00	1,00
140	1,00	,00	2,00	2,00	,00
141	1,00	1,00	1,00	2,00	1,00
142	,00	1,00	1,00	3,00	,00
143	,00	,00	3,00	1,00	,00
144	,00	,00	2,00	3,00	,00
145	,00	,00	2,00	3,00	,00
146	1,00	,00	3,00	3,00	,00
147	,00	,00	3,00	1,00	,00
148	1,00	,00	2,00	2,00	1,00

	Simpson_2gr	WHO_di	Age_cut	Localisation_di
112	,00	1,00	,00	1,00
113	,00	,00	1,00	1,00
114	,00	,00	1,00	.
115	,00	,00	,00	1,00
116	1,00	1,00	1,00	1,00
117	,00	,00	,00	1,00
118	,00	,00	1,00	1,00
119	,00	,00	,00	1,00
120	,00	,00	1,00	,00
121	,00	,00	,00	1,00
122	,00	,00	1,00	1,00
123	,00	1,00	1,00	,00
124	,00	1,00	,00	.
125	,00	,00	,00	.
126	1,00	1,00	1,00	.
127	,00	,00	,00	1,00
128	,00	,00	,00	1,00
129	,00	,00	,00	1,00
130	1,00	,00	1,00	1,00
131	,00	1,00	,00	1,00
132	,00	,00	1,00	,00
133	,00	,00	,00	1,00
134	,00	,00	1,00	1,00
135	,00	,00	1,00	1,00
136	,00	,00	1,00	,00
137	,00	,00	1,00	1,00
138	1,00	,00	,00	.
139	,00	,00	,00	1,00
140	1,00	1,00	1,00	.
141	,00	,00	1,00	,00
142	,00	,00	,00	.
143	1,00	,00	,00	1,00
144	,00	,00	,00	1,00
145	1,00	,00	,00	.
146	,00	,00	1,00	1,00
147	,00	,00	,00	.
148	,00	,00	1,00	1,00

	K3_3gr	Age3gr	RF_r	Reha_r	K3_cut
112	2,00	2,00	1,00	1,00	1,00
113	.	3,00	1,00	2,00	.
114	3,00	3,00	1,00	1,00	,00
115	3,00	2,00	,00	1,00	,00
116	1,00	3,00	1,00	1,00	1,00
117	3,00	1,00	,00	1,00	,00
118	2,00	3,00	1,00	1,00	1,00
119	3,00	2,00	1,00	1,00	,00
120	3,00	2,00	1,00	,00	,00
121	2,00	1,00	1,00	1,00	1,00
122	2,00	2,00	,00	2,00	1,00
123	3,00	3,00	1,00	1,00	,00
124	.	2,00	1,00	,00	.
125	2,00	2,00	1,00	1,00	1,00
126	2,00	3,00	1,00	1,00	1,00
127	2,00	2,00	1,00	1,00	1,00
128	3,00	2,00	1,00	1,00	,00
129	3,00	2,00	1,00	1,00	,00
130	2,00	2,00	1,00	,00	1,00
131	2,00	1,00	1,00	,00	1,00
132	2,00	2,00	1,00	1,00	1,00
133	3,00	2,00	1,00	1,00	,00
134	2,00	3,00	1,00	2,00	1,00
135	2,00	3,00	1,00	1,00	1,00
136	2,00	3,00	1,00	2,00	1,00
137	2,00	3,00	1,00	1,00	1,00
138	3,00	1,00	,00	,00	,00
139	3,00	1,00	1,00	1,00	,00
140	2,00	3,00	1,00	1,00	1,00
141	1,00	3,00	1,00	,00	?
142	3,00	2,00	1,00	1,00	?
143	3,00	1,00	,00	1,00	?
144	3,00	1,00	,00	,00	?
145	3,00	2,00	1,00	1,00	?
146	3,00	2,00	1,00	2,00	?
147	2,00	1,00	,00	1,00	?
148	2,00	2,00	1,00	1,00	?

	Localisation3gr	localisation2gr	Uni_di	age7groups
112	2,00	.	,00	3,00
113	1,00	1,00	1,00	6,00
114	.	.	,00	6,00
115	2,00	.	,00	4,00
116	2,00	.	,00	6,00
117	1,00	1,00	,00	3,00
118	1,00	1,00	1,00	6,00
119	1,00	1,00	,00	4,00
120	3,00	,00	,00	5,00
121	1,00	1,00	1,00	2,00
122	2,00	.	1,00	5,00
123	3,00	,00	,00	6,00
124	.	.	,00	4,00
125	.	.	1,00	4,00
126	.	.	,00	6,00
127	1,00	1,00	,00	5,00
128	1,00	1,00	,00	4,00
129	1,00	1,00	,00	4,00
130	1,00	1,00	,00	5,00
131	1,00	1,00	,00	3,00
132	3,00	,00	,00	5,00
133	1,00	1,00	,00	4,00
134	1,00	1,00	1,00	6,00
135	1,00	1,00	,00	6,00
136	3,00	,00	1,00	6,00
137	1,00	1,00	1,00	7,00
138	.	.	,00	1,00
139	1,00	1,00	,00	3,00
140	.	.	1,00	6,00
141	3,00	,00	1,00	7,00
142	.	.	,00	4,00
143	1,00	1,00	,00	2,00
144	1,00	1,00	,00	1,00
145	.	.	,00	4,00
146	1,00	1,00	,00	5,00
147	.	.	,00	3,00
148	1,00	1,00	1,00	5,00

	Number	Sex	OP.year	Age	Histology.WHO	Type.histology
149	149,00	,00	2011,00	62,00	2,00	12,00
150	150,00	1,00	2011,00	74,00	1,00	1,00
151	151,00	1,00	2011,00	44,00	1,00	1,00
152	152,00	1,00	2011,00	67,00	1,00	4,00
153	153,00	1,00	2011,00	75,00	1,00	1,00
154	154,00	,00	2011,00	55,00	1,00	5,00
155	155,00	1,00	2011,00	72,00	1,00	1,00
156	156,00	1,00	2011,00	69,00	1,00	4,00
157	157,00	,00	2011,00	56,00	1,00	1,00
158	158,00	1,00	2011,00	74,00	1,00	1,00
159	159,00	1,00	2011,00	76,00	1,00	1,00
160	160,00	1,00	2011,00	55,00	1,00	1,00
161	161,00	,00	2011,00	63,00	2,00	12,00
162	162,00	,00	2011,00	55,00	1,00	1,00
163	163,00	,00	2011,00	68,00	1,00	1,00
164	164,00	1,00	2011,00	67,00	1,00	1,00
165	165,00	1,00	2011,00	49,00	1,00	1,00
166	166,00	1,00	2011,00	81,00	2,00	12,00
167	167,00	1,00	2011,00	61,00	1,00	3,00
168	168,00	1,00	2011,00	53,00	1,00	1,00
169	169,00	1,00	2011,00	74,00	1,00	4,00
170	170,00	1,00	2010,00	72,00	1,00	3,00
171	171,00	1,00	2010,00	54,00	1,00	1,00
172	172,00	,00	2010,00	73,00	1,00	1,00
173	173,00	1,00	2010,00	39,00	1,00	1,00
174	174,00	1,00	2010,00	49,00	3,00	15,00
175	175,00	,00	2010,00	39,00	1,00	1,00
176	176,00	,00	2010,00	43,00	1,00	1,00
177	177,00	1,00	2010,00	46,00	1,00	1,00
178	178,00	1,00	2010,00	79,00	1,00	2,00
179	179,00	1,00	2010,00	71,00	1,00	2,00
180	180,00	1,00	2010,00	66,00	1,00	3,00
181	?	?	?	57,00	1,00	1,00
182	182,00	1,00	2010,00	72,00	2,00	12,00
183	183,00	1,00	2010,00	59,00	1,00	2,00
184	184,00	,00	2010,00	58,00	1,00	2,00
185	185,00	1,00	2010,00	55,00	1,00	1,00

	Side	Localisation	Duration.of.symptoms	No.symptoms	First.symptom	Haedache
149	2,00	10,00	6,00	1,00	10,00	,00
150	1,00	2,00	50,00	,00	7,00	,00
151	2,00	12,00	3,00	,00	12,00	1,00
152	2,00	1,00	6,00	,00	4,00	,00
153	1,00	1,00	8,00	,00	15,00	,00
154	2,00	1,00	,00	1,00	16,00	,00
155	1,00	3,00	1,00	1,00	10,00	1,00
156	1,00	10,00	1,00	1,00	15,00	,00
157	2,00	1,00	6,00	1,00	1,00	1,00
158	1,00	6,00	6,00	1,00	15,00	,00
159	1,00	6,00	12,00	,00	12,00	,00
160	2,00	1,00	12,00	,00	4,00	,00
161	1,00	2,00	1,00	,00	14,00	1,00
162	2,00	1,00	36,00	1,00	,00	,00
163	1,00	4,00	,00	1,00	,00	,00
164	2,00	11,00	6,00	,00	15,00	1,00
165	1,00	3,00	9,00	1,00	6,00	,00
166	2,00	11,00	,00	,00	1,00	1,00
167	1,00	1,00	,00	1,00	,00	,00
168	1,00	1,00	12,00	1,00	4,00	,00
169	1,00	4,00	3,00	,00	7,00	,00
170	1,00	2,00	6,00	,00	22,00	,00
171	1,00	3,00	6,00	,00	5,00	,00
172	3,00	5,00	,00	1,00	,00	,00
173	1,00	2,00	,00	1,00	,00	,00
174	1,00	5,00	12,00	1,00	11,00	,00
175	2,00	2,00	1,00	,00	4,00	,00
176	2,00	2,00	1,00	,00	4,00	,00
177	1,00	3,00	1,00	1,00	5,00	,00
178	3,00	1,00	6,00	1,00	14,00	,00
179	2,00	12,00	,00	1,00	,00	,00
180	3,00	5,00	,00	1,00	,00	,00
181	3,00	3,00	6,00	,00	6,00	,00
182	2,00	2,00	12,00	,00	16,00	,00
183	2,00	1,00	2,00	,00	18,00	,00
184	1,00	2,00	3,00	,00	13,00	,00
185	2,00	12,00	2,00	,00	1,00	1,00

	Nausea	Emesis	Nausea_Emesis	Seizures	Oculo.paresis	Viszual.paresis
149	,00	,00	,00	,00	,00	,00
150	,00	,00	,00	,00	,00	,00
151	1,00	,00	1,00	,00	,00	,00
152	,00	,00	,00	1,00	,00	,00
153	,00	,00	,00	,00	,00	,00
154	,00	,00	,00	,00	,00	,00
155	,00	,00	,00	,00	,00	,00
156	,00	,00	,00	,00	,00	,00
157	,00	,00	,00	,00	,00	,00
158	,00	,00	,00	,00	,00	,00
159	,00	,00	,00	,00	,00	,00
160	,00	,00	,00	1,00	,00	,00
161	,00	,00	,00	,00	,00	,00
162	,00	,00	,00	,00	,00	,00
163	,00	,00	,00	,00	,00	,00
164	,00	,00	,00	,00	,00	,00
165	,00	,00	,00	,00	,00	1,00
166	,00	,00	,00	,00	,00	,00
167	,00	,00	,00	,00	,00	,00
168	,00	,00	,00	1,00	,00	,00
169	,00	,00	,00	,00	,00	1,00
170	,00	,00	,00	,00	,00	,00
171	,00	,00	,00	,00	1,00	,00
172	,00	,00	,00	,00	,00	,00
173	,00	,00	,00	,00	,00	,00
174	,00	,00	,00	,00	1,00	1,00
175	,00	,00	,00	1,00	,00	,00
176	,00	,00	,00	1,00	,00	,00
177	,00	,00	,00	,00	1,00	,00
178	,00	,00	,00	,00	,00	,00
179	,00	,00	,00	,00	,00	,00
180	,00	,00	,00	,00	,00	,00
181	,00	,00	,00	,00	,00	1,00
182	,00	,00	,00	,00	,00	,00
183	,00	,00	,00	,00	,00	,00
184	,00	,00	,00	,00	,00	,00
185	,00	,00	,00	,00	,00	,00

	Viszual.deficite	Papilloedema	Optic.atrophie	Exophthalmus	Kakosmia	Other.nerve.paresis
149	,00	,00	,00	1,00	,00	,00
150	1,00	,00	,00	,00	,00	,00
151	,00	,00	,00	,00	,00	1,00
152	,00	,00	,00	,00	,00	,00
153	,00	,00	,00	,00	,00	,00
154	,00	,00	,00	,00	,00	,00
155	,00	,00	,00	1,00	,00	,00
156	,00	,00	,00	,00	,00	,00
157	,00	,00	,00	,00	,00	,00
158	,00	,00	,00	,00	,00	,00
159	,00	,00	,00	,00	,00	1,00
160	,00	,00	,00	,00	,00	,00
161	,00	,00	,00	,00	,00	,00
162	,00	,00	,00	,00	,00	,00
163	,00	,00	,00	,00	,00	,00
164	,00	,00	,00	,00	,00	,00
165	,00	,00	,00	,00	,00	,00
166	,00	,00	,00	,00	,00	,00
167	,00	,00	,00	,00	,00	,00
168	,00	,00	,00	,00	,00	,00
169	1,00	,00	,00	,00	,00	,00
170	,00	,00	,00	,00	,00	,00
171	,00	,00	,00	,00	,00	,00
172	,00	,00	,00	,00	,00	,00
173	,00	,00	,00	,00	,00	,00
174	,00	,00	,00	,00	1,00	1,00
175	,00	,00	,00	,00	,00	,00
176	,00	,00	,00	,00	,00	,00
177	,00	,00	,00	,00	,00	,00
178	,00	,00	,00	,00	,00	,00
179	,00	,00	,00	,00	,00	,00
180	,00	,00	,00	,00	,00	,00
181	1,00	,00	,00	,00	,00	,00
182	,00	,00	,00	,00	,00	,00
183	,00	,00	,00	,00	,00	,00
184	,00	,00	,00	,00	,00	,00
185	,00	,00	,00	,00	,00	,00

	Sensibility.disorder	Motoric.disorder	Cerebellar.symptoms	Aphasia	Concentration.disoreders	Personallity.change
149	,00	,0	,00	,00	,00	,00
150	,00	,0	,00	,00	,00	,00
151	,00	,0	1,00	,00	,00	,00
152	,00	,0	,00	,00	,00	,00
153	,00	,0	1,00	,00	1,00	1,00
154	,00	,0	,00	1,00	,00	1,00
155	,00	,0	1,00	,00	,00	,00
156	,00	,0	1,00	,00	,00	,00
157	,00	1,0	1,00	,00	,00	,00
158	,00	,0	1,00	,00	,00	,00
159	,00	,0	1,00	,00	,00	,00
160	,00	,0	,00	,00	,00	,00
161	,00	1,0	,00	,00	,00	,00
162	,00	,0	,00	,00	,00	,00
163	,00	,0	,00	,00	,00	,00
164	,00	,0	1,00	,00	,00	,00
165	,00	,0	,00	,00	,00	,00
166	,00	,0	1,00	,00	,00	,00
167	,00	,0	,00	,00	,00	,00
168	,00	,0	,00	,00	,00	,00
169	,00	,0	,00	,00	,00	,00
170	,00	,0	,00	,00	,00	,00
171	,00	,0	,00	,00	,00	,00
172	,00	,0	,00	,00	,00	,00
173	,00	,0	,00	,00	,00	,00
174	1,00	,0	1,00	,00	,00	,00
175	,00	,0	,00	,00	,00	,00
176	,00	,0	,00	,00	,00	,00
177	,00	,0	,00	,00	,00	,00
178	,00	1,0	1,00	,00	,00	,00
179	,00	,0	,00	,00	,00	,00
180	,00	,0	,00	,00	,00	,00
181	,00	,0	,00	,00	,00	,00
182	,00	,0	,00	1,00	,00	,00
183	,00	1,0	,00	,00	1,00	1,00
184	1,00	,0	,00	,00	,00	,00
185	,00	,0	,00	,00	,00	,00

	Other.motoric .disorder	loss.counsciu oness	Double.vision	Histological.c lear	Histological.u nclear	Size
149	,00	,00	,00	1,00	,00	1,00
150	,00	,00	,00	1,00	,00	2,00
151	,00	,00	,00	1,00	,00	2,00
152	,00	,00	,00	1,00	,00	1,00
153	,00	,00	,00	1,00	1,00	1,00
154	,00	,00	,00	1,00	,00	1,00
155	,00	,00	,00	1,00	,00	2,00
156	,00	,00	,00	1,00	,00	1,00
157	,00	,00	,00	1,00	,00	1,00
158	,00	,00	,00	1,00	,00	1,00
159	,00	,00	,00	1,00	,00	1,00
160	,00	,00	,00	1,00	,00	1,00
161	,00	,00	,00	1,00	,00	2,00
162	,00	,00	,00	1,00	1,00	1,00
163	,00	,00	,00	1,00	,00	1,00
164	,00	,00	,00	1,00	,00	2,00
165	,00	,00	1,00	1,00	,00	1,00
166	,00	,00	,00	1,00	,00	2,00
167	,00	,00	,00	1,00	,00	1,00
168	,00	,00	,00	1,00	,00	1,00
169	,00	,00	,00	1,00	,00	1,00
170	,00	,00	,00	1,00	,00	1,00
171	,00	,00	,00	1,00	,00	2,00
172	,00	,00	,00	1,00	,00	2,00
173	,00	,00	,00	1,00	,00	2,00
174	,00	,00	,00	1,00	,00	1,00
175	,00	,00	,00	1,00	1,00	2,00
176	,00	,00	,00	1,00	,00	2,00
177	,00	,00	,00	1,00	,00	1,00
178	,00	,00	,00	1,00	,00	2,00
179	,00	,00	,00	1,00	,00	1,00
180	,00	,00	,00	1,00	,00	1,00
181	?	?	?	?	?	?
182	?	?	?	?	?	?
183	?	?	?	?	?	?
184	?	?	?	?	?	?
185	?	?	?	?	?	?

	MRi.CCT	Form	CSF	Edema	Masseffect	Embolisation
149	2,00	2,00	,00	1,00	2,00	,00
150	2,00	1,00	,00	,00	,00	,00
151	2,00	2,00	,00	,00	2,00	,00
152	2,00	1,00	,00	,00	,00	,00
153	2,00	1,00	,00	1,00	2,00	,00
154	2,00	2,00	,00	1,00	2,00	,00
155	2,00	2,00	,00	1,00	1,00	,00
156	2,00	1,00	,00	1,00	2,00	,00
157	2,00	1,00	,00	1,00	2,00	,00
158	2,00	2,00	,00	,00	1,00	,00
159	2,00	2,00	,00	,00	1,00	,00
160	2,00	1,00	,00	1,00	2,00	,00
161	2,00	2,00	,00	1,00	2,00	1,00
162	2,00	1,00	,00	1,00	,00	,00
163	2,00	1,00	,00	,00	1,00	,00
164	2,00	1,00	1,00	1,00	2,00	,00
165	2,00	2,00	,00	,00	1,00	,00
166	2,00	2,00	,00	,00	,00	,00
167	2,00	1,00	,00	,00	,00	,00
168	2,00	1,00	,00	,00	,00	,00
169	2,00	2,00	,00	,00	1,00	,00
170	2,00	1,00	,00	,00	,00	,00
171	2,00	2,00	,00	,00	1,00	,00
172	2,00	2,00	,00	,00	1,00	,00
173	2,00	1,00	,00	,00	1,00	,00
174	2,00	1,00	,00	,00	2,00	,00
175	2,00	1,00	,00	1,00	2,00	1,00
176	2,00	2,00	,00	1,00	1,00	,00
177	2,00	2,00	,00	,00	,00	,00
178	2,00	1,00	,00	1,00	1,00	,00
179	2,00	2,00	,00	,00	1,00	,00
180	2,00	1,00	,00	,00	,00	,00
181	2,00	2,00	,00	1,00	?	?
182	2,00	2,00	,00	1,00	?	?
183	2,00	2,00	,00	1,00	?	?
184	2,00	1,00	,00	1,00	?	?
185	2,00	1,00	,00	,00	?	?

	Hypertonia	Adipositas	Heart.disorder	Lung.disorder	Liver.disorder	Kindey.disorder
149	,00	,00	,00	,00	,00	,00
150	,00	,00	,00	,00	,00	,00
151	,00	,00	,00	1,00	,00	,00
152	,00	,00	,00	,00	,00	,00
153	1,00	1,00	1,00	,00	,00	,00
154	1,00	1,00	,00	,00	,00	,00
155	1,00	,00	,00	,00	,00	,00
156	1,00	,00	,00	,00	,00	,00
157	1,00	,00	,00	,00	,00	,00
158	,00	1,00	1,00	,00	,00	,00
159	1,00	1,00	,00	,00	,00	,00
160	,00	1,00	,00	,00	,00	,00
161	,00	,00	1,00	,00	,00	,00
162	1,00	1,00	,00	,00	,00	,00
163	1,00	,00	,00	,00	,00	,00
164	1,00	,00	,00	,00	,00	,00
165	,00	1,00	,00	,00	,00	,00
166	1,00	1,00	,00	,00	,00	,00
167	1,00	1,00	,00	,00	,00	,00
168	,00	,00	,00	,00	,00	,00
169	1,00	,00	,00	,00	,00	,00
170	1,00	1,00	,00	1,00	,00	,00
171	1,00	,00	,00	,00	,00	,00
172	1,00	,00	,00	1,00	,00	,00
173	,00	,00	,00	,00	,00	,00
174	,00	,00	,00	,00	,00	,00
175	,00	,00	,00	,00	,00	,00
176	1,00	1,00	,00	,00	,00	,00
177	,00	1,00	,00	,00	,00	,00
178	1,00	1,00	1,00	1,00	,00	,00
179	1,00	1,00	1,00	,00	,00	,00
180	1,00	,00	1,00	1,00	,00	,00
181	1,00	,00	,00	,00	,00	,00
182	1,00	,00	1,00	,00	,00	,00
183	,00	,00	,00	,00	,00	,00
184	1,00	,00	,00	,00	,00	,00
185	1,00	1,00	1,00	,00	,00	,00

	Diabetes	Varicosis	ASA	Simpson.grad e	OP.duration	intraOP.brain swelling
149	,00	,00	3,00	4,00	409,00	,00
150	,00	,00	2,00	1,00	120,00	,00
151	,00	,00	2,00	1,00	317,00	,00
152	,00	,00	2,00	2,00	126,00	,00
153	1,00	,00	2,00	1,00	237,00	,00
154	,00	,00	2,00	2,00	240,00	,00
155	,00	,00	3,00	1,00	281,00	,00
156	,00	,00	2,00	1,00	185,00	,00
157	,00	,00	2,00	1,00	237,00	,00
158	,00	,00	2,00	1,00	170,00	,00
159	,00	,00	2,00	2,00	183,00	,00
160	,00	,00	2,00	2,00	194,00	,00
161	,00	,00	2,00	1,00	359,00	,00
162	1,00	,00	3,00	2,00	263,00	,00
163	,00	,00	3,00	2,00	219,00	,00
164	,00	,00	3,00	2,00	199,00	,00
165	,00	,00	2,00	2,00	328,00	,00
166	,00	,00	2,00	4,00	357,00	,00
167	,00	,00	2,00	2,00	215,00	,00
168	,00	,00	3,00	1,00	90,00	,00
169	,00	,00	2,00	2,00	254,00	,00
170	,00	,00	2,00	1,00	140,00	,00
171	1,00	,00	2,00	4,00	244,00	,00
172	,00	,00	3,00	1,00	407,00	,00
173	,00	,00	2,00	1,00	260,00	,00
174	,00	,00	3,00	1,00	308,00	,00
175	,00	,00	2,00	2,00	295,00	,00
176	,00	,00	2,00	1,00	265,00	,00
177	,00	,00	2,00	2,00	323,00	,00
178	1,00	,00	3,00	2,00	362,00	,00
179	1,00	,00	2,00	2,00	195,00	,00
180	1,00	,00	2,00	1,00	290,00	,00
181	,00	,00	2,00	1,00	383,00	,00
182	,00	,00	2,00	2,00	265,00	,00
183	,00	,00	3,00	2,00	221,00	,00
184	,00	,00	2,00	1,00	101,00	,00
185	,00	,00	3,00	1,00	150,00	,00

	Transfusion	Use.CUSA	Craniotomy	Sinus.lesion	Bleeding	Dura.closure
149	6,00	1,00	1,00	,00	,00	1,00
150	,00	,00	1,00	,00	,00	2,00
151	,00	1,00	1,00	,00	,00	1,00
152	,00	1,00	1,00	,00	,00	2,00
153	,00	1,00	1,00	,00	,00	2,00
154	,00	,00	1,00	1,00	,00	2,00
155	,00	1,00	1,00	,00	,00	3,00
156	,00	,00	1,00	,00	,00	1,00
157	,00	1,00	1,00	,00	,00	2,00
158	,00	,00	1,00	1,00	,00	1,00
159	,00	1,00	1,00	,00	,00	1,00
160	,00	1,00	1,00	,00	,00	2,00
161	,00	1,00	1,00	,00	,00	3,00
162	,00	,00	1,00	1,00	,00	3,00
163	,00	1,00	1,00	,00	,00	2,00
164	,00	1,00	1,00	,00	,00	1,00
165	,00	1,00	1,00	,00	,00	2,00
166	,00	1,00	1,00	,00	,00	3,00
167	,00	,00	1,00	1,00	,00	2,00
168	,00	,00	1,00	,00	,00	2,00
169	,00	1,00	2,00	,00	1,00	1,00
170	,00	,00	1,00	,00	,00	2,00
171	,00	1,00	2,00	,00	,00	1,00
172	,00	1,00	1,00	,00	,00	1,00
173	,00	,00	1,00	,00	,00	2,00
174	4,00	,00	1,00	,00	,00	1,00
175	,00	1,00	1,00	,00	,00	2,00
176	,00	1,00	1,00	,00	,00	2,00
177	,00	1,00	1,00	,00	,00	2,00
178	,00	1,00	1,00	,00	,00	2,00
179	,00	1,00	1,00	,00	,00	2,00
180	,00	,00	1,00	,00	,00	2,00
181	,00	1,00	1,00	,00	1,00	1,00
182	2,00	,00	1,00	1,00	1,00	2,00
183	,00	,00	1,00	1,00	1,00	2,00
184	,00	,00	1,00	,00	,00	2,00
185	,00	1,00	2,00	,00	,00	1,00

	Tachosil.Fibrin	Transfusion.postOP	Seizure.therapy	Antibiotics.postOP	CSF.circulatory.disorder	Edema.postOP
149	1,00	,00	2,00	,00	,00	,00
150	1,00	,00	2,00	,00	,00	,00
151	1,00	,00	,00	,00	,00	,00
152	1,00	,00	1,00	,00	,00	,00
153	1,00	,00	,00	,00	,00	,00
154	1,00	,00	,00	,00	,00	,00
155	1,00	,00	,00	,00	,00	,00
156	1,00	,00	,00	,00	,00	,00
157	1,00	,00	,00	,00	,00	,00
158	1,00	,00	,00	,00	,00	,00
159	1,00	,00	,00	1,00	,00	,00
160	1,00	,00	1,00	,00	,00	,00
161	1,00	,00	,00	,00	,00	,00
162	1,00	,00	,00	,00	,00	,00
163	1,00	,00	2,00	,00	,00	,00
164	1,00	,00	,00	,00	,00	1,00
165	1,00	,00	,00	,00	,00	1,00
166	1,00	,00	,00	,00	,00	,00
167	1,00	,00	,00	,00	,00	,00
168	1,00	,00	,00	,00	,00	,00
169	1,00	,00	,00	,00	,00	1,00
170	1,00	,00	2,00	,00	,00	,00
171	1,00	,00	,00	,00	,00	,00
172	1,00	,00	,00	,00	,00	,00
173	1,00	,00	,00	,00	,00	,00
174	1,00	,00	,00	,00	,00	,00
175	1,00	,00	2,00	,00	,00	,00
176	1,00	,00	2,00	,00	,00	,00
177	1,00	,00	,00	,00	,00	,00
178	1,00	,00	,00	,00	,00	1,00
179	1,00	,00	,00	,00	,00	,00
180	1,00	,00	,00	,00	,00	,00
181	1,00	,00	,00	1,00	,00	,00
182	1,00	,00	,00	,00	,00	,00
183	1,00	,00	,00	,00	,00	,00
184	1,00	,00	2,00	,00	,00	,00
185	1,00	,00	,00	1,00	,00	,00

	Kind.of.bleeding	Infection	Revision1	Revision2	Revision3	Seizures.postOP
149	,00	,00	,00	,00	,00	1,00
150	3,00	,00	,00	,00	,00	1,00
151	,00	,00	,00	,00	,00	,00
152	,00	,00	,00	,00	,00	,00
153	,00	,00	,00	,00	,00	,00
154	2,00	,00	2,00	,00	,00	,00
155	,00	,00	,00	,00	,00	,00
156	,00	,00	,00	,00	,00	,00
157	,00	,00	,00	,00	,00	,00
158	,00	,00	,00	,00	,00	,00
159	,00	,00	1,00	1,00	,00	,00
160	,00	,00	,00	,00	,00	,00
161	,00	,00	,00	,00	,00	,00
162	,00	,00	,00	,00	,00	,00
163	,00	,00	,00	,00	,00	1,00
164	,00	,00	1,00	,00	,00	,00
165	,00	,00	,00	,00	,00	,00
166	,00	,00	,00	,00	,00	,00
167	,00	,00	,00	,00	,00	,00
168	,00	,00	,00	,00	,00	,00
169	,00	,00	,00	,00	,00	,00
170	,00	,00	,00	,00	,00	1,00
171	,00	,00	,00	,00	,00	,00
172	,00	,00	,00	,00	,00	,00
173	,00	,00	,00	,00	,00	,00
174	,00	,00	,00	,00	,00	,00
175	,00	,00	,00	,00	,00	,00
176	,00	,00	,00	,00	,00	,00
177	,00	,00	,00	,00	,00	,00
178	,00	,00	2,00	,00	,00	,00
179	,00	,00	,00	,00	,00	,00
180	,00	,00	,00	,00	,00	,00
181	,00	1,00	2,00	,00	,00	,00
182	,00	,00	,00	,00	,00	,00
183	,00	,00	,00	,00	,00	,00
184	,00	,00	,00	,00	,00	1,00
185	,00	1,00	2,00	,00	,00	,00

	Thro.Emb	D.insidipus	Dys.Aphasia	Sens.Hemi	Motor.Hemi	Other.symptoms
149	,00	,00	,00	,00	,00	1,00
150	,00	,00	,00	,00	,00	,00
151	,00	,00	,00	,00	,00	,00
152	,00	,00	,00	,00	,00	,00
153	,00	,00	,00	,00	,00	,00
154	,00	,00	,00	,00	1,00	,00
155	,00	,00	,00	,00	,00	,00
156	,00	,00	,00	,00	,00	,00
157	,00	,00	,00	,00	1,00	,00
158	,00	,00	,00	,00	,00	,00
159	,00	,00	,00	,00	1,00	1,00
160	,00	,00	,00	,00	,00	,00
161	,00	,00	,00	,00	,00	,00
162	,00	,00	,00	,00	,00	1,00
163	,00	,00	,00	,00	,00	,00
164	,00	,00	,00	,00	,00	1,00
165	,00	,00	,00	,00	1,00	1,00
166	,00	,00	,00	,00	,00	1,00
167	,00	,00	,00	,00	,00	,00
168	,00	,00	,00	,00	,00	,00
169	,00	,00	,00	,00	,00	1,00
170	,00	,00	,00	,00	,00	,00
171	,00	,00	,00	,00	,00	,00
172	,00	,00	,00	,00	,00	,00
173	,00	,00	,00	,00	,00	,00
174	,00	,00	,00	,00	,00	,00
175	,00	,00	,00	,00	,00	,00
176	,00	,00	,00	,00	,00	,00
177	,00	,00	,00	,00	,00	,00
178	1,00	,00	,00	,00	,00	,00
179	,00	,00	,00	,00	,00	,00
180	,00	,00	,00	,00	,00	,00
181	,00	,00	,00	,00	,00	,00
182	,00	,00	,00	,00	,00	,00
183	,00	,00	,00	,00	,00	,00
184	,00	,00	,00	,00	,00	,00
185	,00	,00	,00	,00	,00	,00

	ICU.stay	NCH.stay	Total.duration	Rehabilitation	Radiation	Recurrence
149	3,00	13,00	16,00	3,00	,00	,00
150	4,00	9,00	13,00	1,00	,00	,00
151	4,00	5,00	9,00	1,00	,00	,00
152	1,00	4,00	5,00	1,00	,00	,00
153	1,00	7,00	8,00	1,00	,00	,00
154	3,00	9,00	14,00	1,00	,00	,00
155	1,00	10,00	11,00	1,00	,00	,00
156	1,00	6,00	7,00	1,00	,00	,00
157	2,00	8,00	10,00	1,00	,00	,00
158	1,00	5,00	6,00	,00	,00	,00
159	23,00	2,00	25,00	2,00	,00	,00
160	1,00	6,00	7,00	1,00	,00	,00
161	6,00	7,00	?	?	?	?
162	1,00	7,00	?	?	?	?
163	8,00	5,00	?	?	?	?
164	2,00	17,00	?	?	?	?
165	1,00	7,00	?	?	?	?
166	3,00	6,00	?	?	?	?
167	1,00	5,00	?	?	?	?
168	1,00	6,00	?	?	?	?
169	1,00	7,00	?	?	?	?
170	3,00	11,00	?	?	?	?
171	1,00	12,00	?	?	?	?
172	4,00	6,00	?	?	?	?
173	1,00	5,00	?	?	?	?
174	1,00	7,00	?	?	?	?
175	1,00	7,00	?	?	?	?
176	1,00	5,00	?	?	?	?
177	1,00	6,00	?	?	?	?
178	3,00	35,00	?	?	?	?
179	1,00	7,00	?	?	?	?
180	1,00	5,00	?	?	?	?
181	1,00	10,00	?	?	?	?
182	1,00	12,00	?	?	?	?
183	2,00	7,00	?	?	?	?
184	1,00	8,00	?	?	?	?
185	1,00	5,00	?	?	?	?

	Recurrence1	Daeth	Karnofsky.score.pre	Karnofsky.score.post1.3.month	Karnofsky.score.post6.12.month	Difference.K3.K1
149	,00	,00	70,00	60,00	50,00	-20,00
150	,00	,00	80,00	70,00	80,00	,00
151	,00	,00	80,00	70,00	80,00	,00
152	,00	,00	70,00	70,00	80,00	10,00
153	,00	,00	70,00	70,00	70,00	,00
154	,00	,00	60,00	70,00	70,00	10,00
155	,00	,00	70,00	70,00	70,00	,00
156	,00	,00	70,00	70,00	80,00	10,00
157	,00	,00	70,00	70,00	80,00	10,00
158	,00	,00	70,00	80,00	80,00	10,00
159	,00	,00	80,00	50,00	.	.
160	,00	,00	60,00	70,00	70,00	10,00
161	,00	,00	70,00	70,00	70,00	,00
162	,00	,00	90,00	60,00	70,00	-20,00
163	,00	,00	90,00	70,00	80,00	-10,00
164	,00	,00	70,00	70,00	70,00	,00
165	,00	,00	80,00	80,00	80,00	,00
166	1,00	1,00	70,00	70,00	.	.
167	,00	,00	90,00	90,00	90,00	,00
168	,00	,00	90,00	80,00	80,00	-10,00
169	,00	,00	70,00	70,00	70,00	,00
170	,00	,00	80,00	70,00	70,00	-10,00
171	1,00	,00	80,00	70,00	60,00	-20,00
172	,00	,00	50,00	50,00	50,00	,00
173	,00	,00	80,00	70,00	90,00	10,00
174	,00	,00	50,00	50,00	60,00	10,00
175	,00	,00	80,00	80,00	90,00	10,00
176	,00	,00	80,00	80,00	90,00	10,00
177	,00	,00	80,00	90,00	80,00	,00
178	,00	,00	40,00	50,00	50,00	10,00
179	,00	,00	90,00	90,00	90,00	,00
180	,00	,00	80,00	80,00	80,00	,00
181	,00	,00	70,00	80,00	80,00	10,00
182	,00	,00	70,00	70,00	80,00	10,00
183	,00	,00	50,00	70,00	80,00	30,00
184	,00	,00	80,00	80,00	80,00	,00
185	,00	,00	80,00	70,00	90,00	10,00

	Diffenrence.K3.K2	K2K1	Agegroup1	Agegroup2
149	-10,00	-10,00	6,00	4,00
150	10,00	-10,00	7,00	4,00
151	10,00	-10,00	4,00	3,00
152	10,00	,00	6,00	4,00
153	,00	,00	7,00	4,00
154	,00	10,00	5,00	3,00
155	,00	,00	7,00	4,00
156	10,00	,00	6,00	4,00
157	10,00	,00	5,00	3,00
158	,00	10,00	7,00	4,00
159	.	-30,00	7,00	4,00
160	,00	10,00	5,00	3,00
161	?	?	?	?
162	?	?	?	?
163	?	?	?	?
164	?	?	?	?
165	?	?	?	?
166	?	?	?	?
167	?	?	?	?
168	?	?	?	?
169	?	?	?	?
170	?	?	?	?
171	?	?	?	?
172	?	?	?	?
173	?	?	?	?
174	?	?	?	?
175	?	?	?	?
176	?	?	?	?
177	?	?	?	?
178	?	?	?	?
179	?	?	?	?
180	?	?	?	?
181	?	?	?	?
182	?	?	?	?
183	?	?	?	?
184	?	?	?	?
185	?	?	?	?

	Symptoms.duration	Number.tumors	Number.symptoms
149	3,00	,00	1,00
150	6,00	,00	1,00
151	2,00	1,00	4,00
152	3,00	,00	1,00
153	4,00	4,00	3,00
154	1,00	2,00	2,00
155	1,00	1,00	3,00
156	1,00	1,00	1,00
157	3,00	1,00	3,00
158	3,00	2,00	1,00
159	4,00	2,00	2,00
160	4,00	2,00	1,00
161	1,00	1,00	2,00
162	6,00	3,00	,00
163	1,00	1,00	,00
164	3,00	1,00	2,00
165	4,00	1,00	2,00
166	1,00	2,00	2,00
167	1,00	2,00	,00
168	4,00	,00	1,00
169	2,00	1,00	2,00
170	3,00	3,00	,00
171	3,00	2,00	1,00
172	1,00	2,00	,00
173	1,00	,00	,00
174	4,00	,00	6,00
175	1,00	,00	1,00
176	1,00	2,00	1,00
177	1,00	1,00	1,00
178	3,00	5,00	2,00
179	1,00	4,00	,00
180	1,00	4,00	,00
181	3,00	1,00	2,00
182	4,00	2,00	1,00
183	2,00	,00	4,00
184	2,00	1,00	1,00
185	2,00	3,00	1,00

	Operation.time	Volume.transfosion
149	4,00	1698,00
150	1,00	,00
151	3,00	,00
152	2,00	,00
153	2,00	,00
154	2,00	,00
155	3,00	,00
156	2,00	,00
157	2,00	,00
158	2,00	,00
159	2,00	,00
160	2,00	,00
161	3,00	,00
162	3,00	,00
163	2,00	,00
164	2,00	,00
165	3,00	,00
166	3,00	,00
167	2,00	,00
168	1,00	,00
169	3,00	,00
170	2,00	,00
171	3,00	,00
172	4,00	,00
173	3,00	,00
174	3,00	1132,00
175	3,00	,00
176	3,00	,00
177	3,00	,00
178	4,00	,00
179	2,00	,00
180	3,00	,00
181	4,00	,00
182	3,00	566,00
183	2,00	,00
184	1,00	,00
185	2,00	,00

	Volume.transfusion.postOP	Rebleeding	ICU.stay.groups
149	,00	,00	2,00
150	,00	1,00	2,00
151	,00	,00	2,00
152	,00	,00	1,00
153	,00	,00	1,00
154	,00	1,00	2,00
155	,00	,00	1,00
156	,00	,00	1,00
157	,00	,00	1,00
158	,00	,00	1,00
159	,00	,00	5,00
160	,00	,00	1,00
161	,00	,00	3,00
162	,00	,00	1,00
163	,00	,00	3,00
164	,00	,00	1,00
165	,00	,00	1,00
166	,00	,00	2,00
167	,00	,00	1,00
168	,00	,00	1,00
169	,00	,00	1,00
170	,00	,00	2,00
171	,00	,00	1,00
172	,00	,00	2,00
173	,00	,00	1,00
174	,00	,00	1,00
175	,00	,00	1,00
176	,00	,00	1,00
177	,00	,00	1,00
178	,00	,00	2,00
179	,00	,00	1,00
180	,00	,00	1,00
181	,00	?	?
182	,00	?	?
183	,00	?	?
184	,00	?	?
185	,00	?	?

	NCH.stay.groups	Number.symptoms.postOP	Symptoms.postOP	First.symptome.groups
149	2,00	2,00	1,00	4,00
150	2,00	1,00	1,00	4,00
151	1,00	,00	,00	6,00
152	1,00	,00	,00	2,00
153	1,00	,00	,00	7,00
154	2,00	1,00	1,00	8,00
155	2,00	,00	,00	4,00
156	1,00	,00	,00	7,00
157	2,00	1,00	1,00	1,00
158	1,00	,00	,00	7,00
159	1,00	2,00	1,00	6,00
160	1,00	,00	,00	2,00
161	1,00	,00	,00	5,00
162	1,00	1,00	1,00	,00
163	1,00	1,00	1,00	,00
164	3,00	1,00	1,00	7,00
165	1,00	2,00	1,00	4,00
166	1,00	1,00	1,00	1,00
167	1,00	,00	,00	,00
168	1,00	,00	,00	2,00
169	1,00	1,00	1,00	4,00
170	2,00	1,00	1,00	9,00
171	2,00	,00	,00	4,00
172	1,00	,00	,00	,00
173	1,00	,00	,00	,00
174	1,00	,00	,00	6,00
175	1,00	,00	,00	2,00
176	1,00	,00	,00	2,00
177	1,00	,00	,00	4,00
178	5,00	1,00	1,00	5,00
179	1,00	,00	,00	,00
180	1,00	,00	,00	,00
181	2,00	,00	,00	4,00
182	2,00	,00	,00	8,00
183	1,00	,00	,00	3,00
184	2,00	1,00	1,00	5,00
185	1,00	,00	,00	1,00

	Neurological.disorder	Histology.groups	Revision.groups	Localisation.revision
149	1,00	4,00	,00	1,00
150	1,00	1,00	,00	1,00
151	1,00	1,00	,00	1,00
152	1,00	5,00	,00	1,00
153	1,00	1,00	,00	1,00
154	1,00	5,00	1,00	1,00
155	1,00	1,00	,00	1,00
156	1,00	5,00	,00	1,00
157	1,00	1,00	,00	1,00
158	1,00	1,00	,00	1,00
159	1,00	1,00	1,00	1,00
160	1,00	1,00	,00	1,00
161	1,00	4,00	,00	1,00
162	,00	1,00	,00	1,00
163	,00	1,00	,00	1,00
164	1,00	1,00	1,00	1,00
165	1,00	1,00	,00	1,00
166	1,00	4,00	,00	1,00
167	,00	3,00	,00	1,00
168	1,00	1,00	,00	1,00
169	1,00	5,00	,00	1,00
170	,00	3,00	,00	1,00
171	1,00	1,00	,00	1,00
172	,00	1,00	,00	2,00
173	,00	1,00	,00	1,00
174	1,00	5,00	,00	1,00
175	1,00	1,00	,00	1,00
176	1,00	1,00	,00	1,00
177	1,00	1,00	,00	1,00
178	1,00	2,00	1,00	2,00
179	,00	2,00	,00	1,00
180	,00	3,00	,00	2,00
181	1,00	1,00	1,00	2,00
182	1,00	4,00	,00	1,00
183	1,00	2,00	,00	1,00
184	1,00	2,00	,00	1,00
185	1,00	1,00	1,00	1,00

	Masseffect.revision	Simpson.revision	antiepileptc.therapy.revision
149	1,00	4,00	1,00
150	,00	1,00	1,00
151	1,00	1,00	,00
152	,00	1,00	1,00
153	1,00	1,00	,00
154	1,00	1,00	,00
155	1,00	1,00	,00
156	1,00	1,00	,00
157	1,00	1,00	,00
158	1,00	1,00	,00
159	1,00	1,00	,00
160	1,00	1,00	1,00
161	?	?	?
162	?	?	?
163	?	?	?
164	?	?	?
165	?	?	?
166	?	?	?
167	?	?	?
168	?	?	?
169	?	?	?
170	?	?	?
171	?	?	?
172	?	?	?
173	?	?	?
174	?	?	?
175	?	?	?
176	?	?	?
177	?	?	?
178	?	?	?
179	?	?	?
180	?	?	?
181	?	?	?
182	?	?	?
183	?	?	?
184	?	?	?
185	?	?	?

	Post.revision.symptoms	Recurrence.revision	ASA.class.4	Kd_disorder
149	,00	,00	3,00	1,00
150	,00	,00	2,00	,00
151	,00	,00	2,00	,00
152	,00	,00	2,00	,00
153	,00	,00	2,00	,00
154	1,00	,00	2,00	,00
155	,00	,00	3,00	,00
156	,00	,00	2,00	,00
157	1,00	,00	2,00	,00
158	,00	,00	2,00	,00
159	1,00	,00	2,00	.
160	,00	,00	2,00	,00
161	,00	,00	2,00	,00
162	,00	,00	3,00	1,00
163	,00	,00	3,00	1,00
164	,00	,00	3,00	,00
165	1,00	,00	2,00	,00
166	,00	,00	2,00	.
167	,00	,00	2,00	,00
168	,00	,00	3,00	1,00
169	,00	,00	2,00	,00
170	,00	,00	2,00	1,00
171	,00	,00	2,00	1,00
172	,00	,00	3,00	,00
173	,00	,00	2,00	,00
174	,00	,00	3,00	,00
175	,00	,00	2,00	,00
176	,00	,00	2,00	,00
177	,00	,00	2,00	,00
178	,00	,00	3,00	,00
179	,00	,00	2,00	,00
180	,00	,00	2,00	,00
181	,00	,00	2,00	,00
182	,00	,00	2,00	,00
183	,00	,00	3,00	,00
184	,00	,00	2,00	,00
185	,00	,00	3,00	,00

	age_disorder	K1_cut	K1_3gr	Kd_3gr	ASA_di
149	1,00	,00	2,00	1,00	1,00
150	1,00	,00	3,00	2,00	,00
151	,00	,00	3,00	2,00	,00
152	1,00	,00	2,00	3,00	,00
153	1,00	,00	2,00	2,00	,00
154	,00	1,00	1,00	3,00	,00
155	1,00	,00	2,00	2,00	1,00
156	1,00	,00	2,00	3,00	,00
157	,00	,00	2,00	3,00	,00
158	1,00	,00	2,00	3,00	,00
159	1,00	,00	3,00	.	,00
160	,00	1,00	1,00	3,00	,00
161	1,00	,00	2,00	2,00	,00
162	,00	,00	3,00	1,00	1,00
163	1,00	,00	3,00	1,00	1,00
164	1,00	,00	2,00	2,00	1,00
165	,00	,00	3,00	2,00	,00
166	1,00	,00	2,00	.	,00
167	1,00	,00	3,00	2,00	,00
168	,00	,00	3,00	1,00	1,00
169	1,00	,00	2,00	2,00	,00
170	1,00	,00	3,00	1,00	,00
171	,00	,00	3,00	1,00	,00
172	1,00	1,00	1,00	2,00	1,00
173	,00	,00	3,00	3,00	,00
174	,00	1,00	1,00	3,00	1,00
175	,00	,00	3,00	3,00	,00
176	,00	,00	3,00	3,00	,00
177	,00	,00	3,00	2,00	,00
178	1,00	1,00	1,00	3,00	1,00
179	1,00	,00	3,00	2,00	,00
180	1,00	,00	3,00	2,00	,00
181	,00	,00	2,00	3,00	,00
182	1,00	,00	2,00	3,00	,00
183	,00	1,00	1,00	3,00	1,00
184	,00	,00	3,00	2,00	,00
185	,00	,00	3,00	3,00	1,00

	Simpson_2gr	WHO_di	Age_cut	Localisation_di
149	1,00	1,00	,00	1,00
150	,00	,00	1,00	,00
151	,00	,00	,00	.
152	,00	,00	1,00	1,00
153	,00	,00	1,00	1,00
154	,00	,00	,00	1,00
155	,00	,00	1,00	1,00
156	,00	,00	1,00	1,00
157	,00	,00	,00	1,00
158	,00	,00	1,00	.
159	,00	,00	1,00	.
160	,00	,00	,00	1,00
161	,00	1,00	,00	,00
162	,00	,00	,00	1,00
163	,00	,00	1,00	1,00
164	,00	,00	1,00	1,00
165	,00	,00	,00	1,00
166	1,00	1,00	1,00	1,00
167	,00	,00	,00	1,00
168	,00	,00	,00	1,00
169	,00	,00	1,00	1,00
170	,00	,00	1,00	,00
171	1,00	,00	,00	1,00
172	,00	,00	1,00	1,00
173	,00	,00	,00	,00
174	,00	1,00	,00	1,00
175	,00	,00	,00	,00
176	,00	,00	,00	,00
177	,00	,00	,00	1,00
178	,00	,00	1,00	1,00
179	,00	,00	1,00	.
180	,00	,00	1,00	1,00
181	,00	,00	,00	1,00
182	,00	1,00	1,00	,00
183	,00	,00	,00	1,00
184	,00	,00	,00	,00
185	,00	,00	,00	.

	K3_3gr	Age3gr	RF_r	Reha_r	K3_cut
149	1,00	2,00	,00	2,00	1,00
150	3,00	3,00	,00	1,00	,00
151	3,00	1,00	1,00	1,00	,00
152	3,00	2,00	,00	1,00	,00
153	2,00	3,00	1,00	1,00	1,00
154	2,00	2,00	1,00	1,00	1,00
155	2,00	3,00	1,00	1,00	1,00
156	3,00	2,00	1,00	1,00	,00
157	3,00	2,00	1,00	1,00	,00
158	3,00	3,00	1,00	,00	,00
159	.	3,00	1,00	2,00	.
160	2,00	2,00	1,00	1,00	1,00
161	2,00	2,00	1,00	1,00	1,00
162	2,00	2,00	1,00	1,00	1,00
163	3,00	2,00	1,00	1,00	,00
164	2,00	2,00	1,00	1,00	1,00
165	3,00	1,00	1,00	1,00	,00
166	.	3,00	1,00	,00	.
167	3,00	2,00	1,00	1,00	,00
168	3,00	2,00	,00	1,00	,00
169	2,00	3,00	1,00	1,00	1,00
170	2,00	3,00	1,00	1,00	1,00
171	1,00	2,00	1,00	1,00	1,00
172	1,00	3,00	1,00	,00	1,00
173	3,00	1,00	,00	1,00	,00
174	1,00	1,00	,00	,00	1,00
175	3,00	1,00	,00	1,00	,00
176	3,00	1,00	1,00	,00	,00
177	3,00	1,00	1,00	1,00	,00
178	1,00	3,00	1,00	2,00	1,00
179	3,00	3,00	1,00	,00	,00
180	3,00	2,00	1,00	1,00	,00
181	3,00	2,00	1,00	1,00	,00
182	3,00	3,00	1,00	,00	,00
183	3,00	2,00	,00	,00	,00
184	3,00	2,00	1,00	1,00	,00
185	3,00	2,00	1,00	1,00	,00

	Localisation3gr	localisation2gr	Uni_di	age7groups
149	1,00	1,00	1,00	5,00
150	3,00	,00	1,00	6,00
151	.	.	,00	3,00
152	2,00	.	,00	5,00
153	2,00	.	,00	6,00
154	2,00	.	1,00	4,00
155	1,00	1,00	,00	6,00
156	1,00	1,00	,00	5,00
157	2,00	.	,00	4,00
158	.	.	,00	6,00
159	.	.	1,00	6,00
160	2,00	.	,00	4,00
161	3,00	,00	1,00	5,00
162	2,00	.	,00	4,00
163	1,00	1,00	1,00	5,00
164	1,00	1,00	1,00	5,00
165	1,00	1,00	,00	3,00
166	1,00	1,00	,00	7,00
167	2,00	.	,00	5,00
168	2,00	.	,00	4,00
169	1,00	1,00	,00	6,00
170	3,00	,00	1,00	6,00
171	1,00	1,00	1,00	4,00
172	1,00	1,00	,00	6,00
173	3,00	,00	,00	2,00
174	1,00	1,00	,00	3,00
175	3,00	,00	,00	2,00
176	3,00	,00	,00	3,00
177	1,00	1,00	,00	3,00
178	2,00	.	1,00	6,00
179	.	.	,00	6,00
180	1,00	1,00	,00	5,00
181	1,00	1,00	,00	4,00
182	3,00	,00	1,00	6,00
183	2,00	.	,00	4,00
184	3,00	,00	,00	4,00
185	.	.	,00	4,00

	Number	Sex	OP.year	Age	Histology.WHO	Type.histology
186	186,00	1,00	2010,00	74,00	1,00	1,00
187	187,00	,00	2010,00	55,00	1,00	2,00
188	188,00	,00	2010,00	48,00	2,00	12,00
189	189,00	1,00	2010,00	31,00	1,00	1,00
190	190,00	1,00	2010,00	57,00	1,00	1,00
191	191,00	1,00	2010,00	57,00	1,00	1,00
192	192,00	1,00	2010,00	25,00	2,00	12,00
193	193,00	1,00	2010,00	63,00	1,00	1,00
194	194,00	,00	2010,00	39,00	1,00	5,00
195	195,00	1,00	2010,00	78,00	1,00	2,00
196	196,00	1,00	2010,00	78,00	1,00	2,00
197	197,00	1,00	2010,00	58,00	1,00	1,00
198	198,00	,00	2010,00	66,00	2,00	12,00
199	199,00	1,00	2010,00	70,00	2,00	12,00
200	200,00	,00	2010,00	29,00	1,00	1,00
201	?	?	?	?	?	?
202	?	?	?	?	?	?
203	?	?	?	?	?	?
204	?	?	?	?	?	?
205	?	?	?	?	?	?
206	?	?	?	?	?	?
207	?	?	?	?	?	?
208	?	?	?	?	?	?
209	?	?	?	?	?	?
210	?	?	?	?	?	?
211	?	?	?	?	?	?
212	?	?	?	?	?	?
213	?	?	?	?	?	?
214	?	?	?	?	?	?
215	?	?	?	?	?	?
216	?	?	?	?	?	?
217	?	?	?	?	?	?
218	?	?	?	?	?	?
219	?	?	?	?	?	?
220	?	?	?	?	?	?
221	?	?	?	?	?	?
222	?	?	?	?	?	?

	Side	Localisation	Duration.of.symptoms	No.symptoms	First.symptom	Haedache
186	1,00	2,00	,00	1,00	,00	,00
187	1,00	13,00	,00	1,00	15,00	,00
188	1,00	2,00	1,00	,00	4,00	,00
189	2,00	7,00	10,00	,00	12,00	,00
190	1,00	1,00	6,00	,00	1,00	1,00
191	2,00	1,00	,00	1,00	,00	,00
192	2,00	3,00	4,00	,00	1,00	1,00
193	1,00	3,00	36,00	1,00	12,00	,00
194	2,00	13,00	,00	1,00	1,00	1,00
195	2,00	7,00	1,00	1,00	15,00	,00
196	2,00	2,00	4,00	,00	15,00	,00
197	1,00	8,00	,00	,00	7,00	,00
198	2,00	1,00	,00	1,00	4,00	,00
199	2,00	3,00	,00	1,00	,00	,00
200	1,00	6,00	1,00	1,00	15,00	,00
201	1,00	2,00	4,00	,00	4,00	,00
202	1,00	1,00	9,00	,00	14,00	,00
203	3,00	5,00	4,00	,00	1,00	1,00
204	2,00	3,00	3,00	,00	1,00	1,00
205	1,00	11,00	72,00	,00	1,00	1,00
206	2,00	3,00	,00	1,00	,00	,00
207	2,00	2,00	,00	1,00	16,00	1,00
208	1,00	1,00	36,00	1,00	21,00	,00
209	3,00	5,00	72,00	,00	1,00	1,00
210	2,00	6,00	18,00	,00	1,00	1,00
211	2,00	6,00	6,00	,00	1,00	1,00
212	2,00	11,00	18,00	,00	12,00	1,00
213	1,00	8,00	72,00	,00	10,00	,00
214	1,00	13,00	,00	1,00	4,00	,00
215	2,00	2,00	6,00	,00	17,00	,00
216	3,00	10,00	,00	1,00	,00	,00
217	1,00	2,00	1,00	1,00	22,00	,00
218	2,00	2,00	6,00	,00	1,00	1,00
219	1,00	1,00	1,00	,00	4,00	,00
220	2,00	3,00	2,00	,00	16,00	,00
221	2,00	3,00	,00	1,00	4,00	,00
222	1,00	13,00	3,00	,00	4,00	,00

	Nausea	Emesis	Nausea_Emesis	Seizures	Oculo.paresis	Viszual.paresis
186	,00	,00	,00	,00	,00	,00
187	,00	,00	,00	,00	,00	,00
188	,00	,00	,00	1,00	,00	,00
189	,00	,00	,00	,00	,00	,00
190	,00	,00	,00	,00	,00	,00
191	,00	,00	,00	,00	,00	,00
192	,00	,00	,00	,00	,00	,00
193	,00	,00	,00	,00	,00	,00
194	1,00	,00	1,00	,00	,00	,00
195	,00	,00	,00	,00	,00	,00
196	,00	,00	,00	,00	,00	,00
197	,00	,00	,00	,00	,00	,00
198	,00	,00	,00	1,00	,00	,00
199	,00	,00	,00	,00	,00	,00
200	1,00	1,00	1,00	,00	,00	,00
201	,00	,00	,00	1,00	,00	,00
202	,00	,00	,00	,00	,00	,00
203	,00	,00	,00	,00	,00	1,00
204	,00	,00	,00	,00	,00	,00
205	,00	,00	,00	,00	,00	,00
206	,00	,00	,00	,00	,00	,00
207	,00	,00	,00	,00	,00	,00
208	,00	,00	,00	,00	,00	,00
209	,00	,00	,00	,00	,00	,00
210	,00	,00	,00	,00	,00	,00
211	1,00	,00	1,00	,00	,00	,00
212	,00	,00	,00	,00	,00	,00
213	,00	,00	,00	,00	,00	,00
214	,00	,00	,00	1,00	,00	,00
215	,00	,00	,00	,00	,00	,00
216	,00	,00	,00	,00	,00	,00
217	,00	,00	,00	,00	,00	,00
218	,00	,00	,00	,00	,00	,00
219	,00	,00	,00	1,00	,00	,00
220	,00	,00	,00	,00	,00	,00
221	,00	,00	,00	1,00	,00	,00
222	,00	,00	,00	1,00	,00	,00

	Viszual.deficite	Papilloedema	Optic.atrophie	Exophthalmus	Kakosmia	Other.nerve.paresis
186	,00	,00	,00	,00	,00	,00
187	,00	,00	,00	,00	,00	,00
188	,00	,00	,00	,00	,00	,00
189	,00	,00	,00	,00	,00	1,00
190	,00	,00	,00	,00	,00	,00
191	,00	,00	,00	,00	,00	,00
192	,00	,00	,00	,00	,00	,00
193	,00	,00	,00	,00	,00	1,00
194	,00	,00	,00	,00	,00	,00
195	,00	,00	,00	,00	,00	,00
196	,00	,00	,00	,00	,00	,00
197	1,00	,00	,00	1,00	,00	,00
198	,00	,00	,00	,00	,00	,00
199	,00	,00	,00	,00	,00	,00
200	,00	,00	,00	,00	,00	,00
201	,00	,00	,00	,00	,00	,00
202	,00	,00	,00	,00	,00	,00
203	1,00	,00	,00	,00	1,00	1,00
204	,00	,00	,00	,00	,00	,00
205	,00	,00	,00	,00	,00	,00
206	,00	,00	,00	,00	,00	,00
207	,00	,00	,00	,00	,00	,00
208	1,00	,00	,00	,00	,00	,00
209	,00	,00	,00	,00	,00	,00
210	,00	,00	,00	,00	,00	,00
211	,00	,00	,00	,00	,00	,00
212	,00	,00	,00	,00	,00	1,00
213	,00	,00	,00	1,00	,00	,00
214	,00	,00	,00	,00	,00	,00
215	,00	,00	,00	,00	,00	,00
216	,00	,00	,00	,00	,00	,00
217	,00	,00	,00	,00	,00	,00
218	,00	,00	,00	,00	,00	,00
219	,00	,00	,00	,00	,00	,00
220	,00	,00	,00	,00	,00	,00
221	,00	,00	,00	,00	,00	,00
222	,00	,00	,00	,00	,00	,00

	Sensibility.disorder	Motoric.disorder	Cerebellar.symptoms	Aphasia	Concentration.disoreders	Personallity.change
186	,00	,0	,00	,00	,00	,00
187	,00	,0	1,00	,00	,00	,00
188	,00	,0	,00	,00	,00	,00
189	,00	,0	,00	,00	,00	,00
190	,00	1,0	,00	,00	,00	,00
191	,00	,0	,00	,00	,00	,00
192	,00	,0	,00	,00	,00	,00
193	,00	,0	,00	,00	,00	,00
194	,00	,0	,00	,00	1,00	,00
195	,00	,0	1,00	,00	,00	,00
196	,00	,0	1,00	,00	,00	,00
197	,00	,0	,00	,00	,00	,00
198	,00	,0	,00	,00	,00	,00
199	,00	,0	,00	,00	,00	,00
200	,00	,0	1,00	,00	,00	,00
201	,00	,0	,00	,00	,00	,00
202	,00	1,0	,00	,00	,00	,00
203	,00	,0	,00	,00	1,00	,00
204	,00	,0	,00	,00	,00	,00
205	,00	,0	,00	,00	,00	,00
206	,00	,0	,00	,00	,00	,00
207	,00	,0	,00	1,00	,00	1,00
208	,00	,0	,00	,00	,00	,00
209	,00	,0	,00	,00	,00	,00
210	,00	,0	1,00	,00	,00	,00
211	,00	,0	1,00	,00	1,00	,00
212	,00	,0	1,00	,00	,00	,00
213	,00	,0	,00	,00	,00	,00
214	,00	,0	,00	,00	,00	,00
215	,00	,0	,00	,00	1,00	,00
216	,00	,0	,00	,00	,00	,00
217	,00	,0	,00	,00	,00	1,00
218	,00	,0	,00	,00	,00	,00
219	,00	,0	,00	,00	,00	,00
220	,00	,0	,00	1,00	1,00	,00
221	,00	,0	,00	,00	,00	,00
222	,00	,0	,00	,00	,00	,00

	Other.motoric.disorder	loss.counsciuoness	Double.vision	Histological.clear	Histological.unclear	Size
186	,00	,00	,00	1,00	,00	1,00
187	,00	,00	,00	1,00	,00	1,00
188	,00	,00	,00	1,00	,00	2,00
189	,00	,00	,00	1,00	,00	1,00
190	,00	,00	,00	1,00	,00	1,00
191	,00	,00	,00	1,00	,00	1,00
192	,00	,00	,00	1,00	,00	2,00
193	,00	,00	,00	1,00	,00	1,00
194	,00	,00	,00	1,00	,00	2,00
195	,00	,00	,00	1,00	1,00	1,00
196	,00	,00	,00	1,00	,00	1,00
197	,00	,00	,00	1,00	,00	1,00
198	,00	,00	,00	1,00	,00	2,00
199	,00	,00	,00	1,00	,00	1,00
200	,00	,00	,00	1,00	,00	1,00
201	,00	,00	,00	1,00	,00	1,00
202	,00	,00	,00	1,00	,00	2,00
203	,00	,00	,00	1,00	,00	1,00
204	,00	,00	,00	1,00	,00	2,00
205	,00	,00	,00	1,00	,00	2,00
206	,00	,00	,00	1,00	,00	1,00
207	,00	,00	,00	1,00	,00	2,00
208	,00	,00	1,00	1,00	,00	2,00
209	,00	,00	,00	1,00	,00	2,00
210	,00	,00	,00	1,00	1,00	2,00
211	,00	,00	,00	1,00	,00	2,00
212	,00	,00	,00	1,00	,00	1,00
213	,00	,00	1,00	1,00	,00	1,00
214	,00	,00	,00	1,00	,00	1,00
215	,00	,00	,00	1,00	,00	1,00
216	,00	,00	,00	1,00	,00	2,00
217	,00	,00	,00	1,00	,00	1,00
218	,00	,00	,00	1,00	,00	1,00
219	,00	,00	,00	1,00	,00	1,00
220	,00	,00	,00	1,00	,00	1,00
221	?	?	?	?	?	?
222	?	?	?	?	?	?

	MRi.CCT	Form	CSF	Edema	Masseffect	Embolisation
186	2,00	1,00	,00	,00	,00	,00
187	2,00	1,00	,00	,00	2,00	,00
188	2,00	2,00	,00	1,00	2,00	,00
189	2,00	2,00	,00	,00	1,00	,00
190	2,00	2,00	,00	,00	,00	,00
191	2,00	1,00	,00	,00	,00	,00
192	2,00	2,00	,00	1,00	2,00	1,00
193	2,00	2,00	,00	,00	,00	,00
194	2,00	2,00	,00	1,00	2,00	,00
195	2,00	2,00	1,00	1,00	2,00	,00
196	2,00	1,00	,00	1,00	1,00	,00
197	2,00	2,00	,00	,00	,00	,00
198	2,00	2,00	,00	1,00	,00	,00
199	2,00	2,00	,00	,00	,00	,00
200	2,00	2,00	,00	,00	,00	,00
201	2,00	1,00	,00	1,00	1,00	,00
202	2,00	1,00	,00	,00	2,00	1,00
203	2,00	2,00	,00	,00	,00	,00
204	2,00	2,00	,00	1,00	2,00	1,00
205	2,00	1,00	,00	,00	1,00	,00
206	2,00	2,00	,00	,00	,00	,00
207	2,00	2,00	,00	1,00	2,00	,00
208	2,00	1,00	,00	1,00	2,00	,00
209	2,00	2,00	,00	1,00	1,00	,00
210	2,00	2,00	,00	1,00	2,00	,00
211	2,00	1,00	,00	1,00	1,00	,00
212	2,00	1,00	,00	,00	1,00	,00
213	2,00	2,00	,00	,00	1,00	,00
214	2,00	1,00	,00	1,00	,00	,00
215	2,00	1,00	,00	,00	,00	,00
216	2,00	2,00	,00	1,00	,00	,00
217	2,00	1,00	,00	,00	,00	,00
218	2,00	2,00	,00	,00	2,00	,00
219	2,00	1,00	,00	1,00	,00	,00
220	2,00	1,00	,00	1,00	2,00	,00
221	2,00	2,00	,00	,00	1,00	,00
222	2,00	2,00	,00	,00	,00	,00

	Hypertonia	Adipositas	Heart.disorder	Lung.disorder	Liver.disorder	Kindey.disorder
186	1,00	,00	,00	,00	,00	,00
187	,00	,00	1,00	,00	,00	,00
188	1,00	1,00	,00	,00	,00	,00
189	,00	1,00	,00	,00	,00	,00
190	1,00	,00	,00	,00	,00	,00
191	,00	,00	,00	,00	,00	,00
192	,00	,00	,00	,00	,00	,00
193	1,00	1,00	,00	,00	,00	,00
194	,00	1,00	,00	,00	,00	,00
195	1,00	,00	,00	,00	,00	,00
196	1,00	1,00	1,00	,00	,00	,00
197	,00	,00	,00	1,00	,00	,00
198	,00	,00	,00	,00	,00	,00
199	1,00	,00	1,00	,00	,00	,00
200	,00	1,00	,00	,00	,00	,00
201	1,00	1,00	,00	,00	,00	,00
202	1,00	,00	,00	,00	,00	,00
203	,00	,00	,00	,00	,00	,00
204	,00	,00	,00	,00	,00	,00
205	1,00	,00	,00	,00	,00	,00
206	,00	,00	,00	,00	,00	,00
207	,00	,00	,00	,00	,00	,00
208	,00	1,00	1,00	,00	,00	,00
209	,00	,00	,00	,00	,00	,00
210	1,00	,00	,00	,00	,00	,00
211	1,00	,00	,00	,00	,00	,00
212	,00	,00	,00	,00	,00	,00
213	1,00	1,00	1,00	,00	,00	,00
214	,00	,00	,00	,00	,00	,00
215	,00	1,00	,00	,00	,00	,00
216	,00	,00	,00	,00	,00	,00
217	1,00	,00	,00	,00	,00	,00
218	,00	1,00	,00	,00	,00	,00
219	,00	,00	,00	1,00	,00	,00
220	,00	,00	,00	,00	,00	,00
221	1,00	1,00	,00	,00	,00	,00
222	1,00	,00	,00	1,00	,00	,00

	Diabetes	Varicosis	ASA	Simpson.grade	OP.duration	intraOP.brain swelling
186	1,00	,00	2,00	1,00	167,00	,00
187	1,00	,00	3,00	1,00	174,00	,00
188	,00	,00	2,00	1,00	263,00	,00
189	,00	,00	1,00	4,00	463,00	,00
190	,00	,00	2,00	2,00	112,00	,00
191	,00	,00	2,00	2,00	139,00	,00
192	,00	,00	1,00	2,00	395,00	,00
193	,00	,00	2,00	1,00	122,00	,00
194	,00	,00	1,00	1,00	215,00	,00
195	1,00	,00	2,00	2,00	375,00	,00
196	1,00	1,00	3,00	1,00	94,00	,00
197	,00	,00	2,00	4,00	300,00	,00
198	,00	,00	2,00	2,00	268,00	,00
199	,00	,00	2,00	2,00	211,00	,00
200	,00	,00	2,00	2,00	189,00	,00
201	,00	,00	2,00	4,00	518,00	,00
202	1,00	,00	2,00	2,00	299,00	,00
203	,00	,00	2,00	1,00	200,00	,00
204	,00	,00	4,00	2,00	318,00	,00
205	,00	,00	2,00	1,00	331,00	,00
206	,00	,00	2,00	2,00	358,00	,00
207	,00	,00	2,00	2,00	262,00	,00
208	,00	,00	2,00	1,00	338,00	1,00
209	,00	,00	1,00	1,00	398,00	1,00
210	,00	,00	2,00	2,00	494,00	1,00
211	1,00	,00	2,00	1,00	227,00	,00
212	,00	,00	2,00	1,00	244,00	,00
213	,00	,00	2,00	4,00	566,00	,00
214	,00	,00	3,00	2,00	394,00	,00
215	,00	,00	2,00	1,00	115,00	,00
216	,00	,00	2,00	1,00	317,00	,00
217	,00	,00	2,00	1,00	158,00	,00
218	,00	,00	2,00	1,00	241,00	,00
219	,00	,00	3,00	2,00	287,00	,00
220	,00	,00	2,00	1,00	196,00	,00
221	,00	,00	2,00	4,00	256,00	,00
222	,00	,00	2,00	2,00	77,00	,00

	Transfusion	Use.CUSA	Craniotomy	Sinus.lesion	Bleeding	Dura.closure
186	,00	,00	2,00	,00	,00	2,00
187	,00	,00	1,00	,00	,00	3,00
188	,00	,00	1,00	,00	,00	2,00
189	,00	1,00	1,00	,00	,00	1,00
190	,00	,00	1,00	,00	,00	1,00
191	,00	,00	1,00	,00	,00	2,00
192	,00	1,00	1,00	,00	,00	2,00
193	,00	,00	1,00	,00	,00	1,00
194	,00	1,00	1,00	,00	,00	2,00
195	2,00	1,00	1,00	,00	,00	3,00
196	,00	,00	1,00	,00	,00	1,00
197	,00	,00	1,00	,00	,00	1,00
198	,00	1,00	1,00	,00	,00	2,00
199	,00	,00	1,00	1,00	,00	3,00
200	,00	,00	1,00	,00	,00	1,00
201	2,00	1,00	1,00	,00	,00	1,00
202	,00	1,00	1,00	,00	,00	1,00
203	,00	1,00	1,00	,00	,00	1,00
204	,00	1,00	1,00	,00	,00	1,00
205	,00	1,00	1,00	,00	,00	1,00
206	,00	1,00	1,00	,00	,00	2,00
207	,00	1,00	1,00	,00	,00	1,00
208	,00	1,00	1,00	,00	,00	3,00
209	,00	1,00	1,00	,00	,00	1,00
210	,00	1,00	1,00	1,00	,00	3,00
211	,00	1,00	1,00	,00	,00	1,00
212	,00	1,00	1,00	,00	,00	3,00
213	,00	,00	1,00	,00	,00	3,00
214	,00	,00	1,00	1,00	,00	1,00
215	,00	,00	1,00	,00	,00	1,00
216	,00	1,00	1,00	,00	,00	1,00
217	,00	,00	1,00	,00	,00	2,00
218	,00	,00	1,00	,00	,00	3,00
219	,00	1,00	1,00	1,00	,00	3,00
220	,00	,00	1,00	,00	,00	1,00
221	,00	,00	1,00	,00	,00	1,00
222	,00	,00	1,00	,00	,00	3,00

	Tachosil.Fibrin	Transfusion.postOP	Seizure.therapy	Antibiotics.postOP	CSF.circulatory.disorder	Edema.postOP
186	1,00	,00	,00	,00	,00	,00
187	1,00	,00	,00	,00	,00	,00
188	1,00	,00	2,00	,00	,00	,00
189	1,00	,00	,00	,00	,00	,00
190	1,00	,00	,00	,00	,00	,00
191	1,00	,00	,00	,00	,00	,00
192	1,00	,00	,00	,00	,00	,00
193	1,00	,00	,00	,00	,00	,00
194	1,00	,00	,00	,00	,00	,00
195	1,00	,00	,00	,00	1,00	,00
196	1,00	,00	,00	,00	,00	,00
197	1,00	,00	,00	,00	,00	1,00
198	1,00	,00	2,00	,00	,00	1,00
199	1,00	,00	,00	,00	,00	,00
200	1,00	,00	,00	,00	,00	,00
201	1,00	3,00	2,00	,00	,00	1,00
202	1,00	,00	1,00	,00	,00	,00
203	1,00	,00	,00	,00	,00	,00
204	1,00	,00	1,00	,00	,00	,00
205	1,00	,00	,00	,00	,00	,00
206	1,00	,00	,00	,00	,00	,00
207	1,00	,00	1,00	,00	,00	,00
208	1,00	,00	,00	1,00	,00	,00
209	1,00	,00	,00	,00	,00	,00
210	1,00	,00	,00	,00	,00	,00
211	1,00	,00	,00	,00	,00	,00
212	1,00	,00	,00	,00	,00	,00
213	1,00	,00	,00	,00	,00	,00
214	1,00	,00	2,00	,00	,00	,00
215	1,00	,00	1,00	,00	,00	,00
216	1,00	,00	,00	1,00	,00	,00
217	1,00	,00	,00	,00	,00	,00
218	1,00	,00	,00	,00	,00	,00
219	1,00	,00	2,00	,00	,00	,00
220	1,00	,00	2,00	,00	,00	,00
221	1,00	,00	2,00	,00	,00	,00
222	1,00	,00	,00	1,00	,00	,00

	Kind.of.bleeding	Infection	Revision1	Revision2	Revision3	Seizures.postOP
186	,00	,00	,00	,00	,00	,00
187	,00	,00	,00	,00	,00	,00
188	,00	,00	,00	,00	,00	,00
189	,00	,00	,00	,00	,00	,00
190	,00	,00	,00	,00	,00	,00
191	,00	,00	,00	,00	,00	,00
192	,00	,00	,00	,00	,00	,00
193	,00	,00	,00	,00	,00	,00
194	,00	,00	,00	,00	,00	,00
195	2,00	,00	1,00	,00	,00	,00
196	,00	,00	,00	,00	,00	,00
197	,00	,00	,00	,00	,00	,00
198	,00	,00	,00	,00	,00	,00
199	3,00	,00	,00	,00	,00	,00
200	,00	,00	2,00	,00	,00	,00
201	,00	,00	,00	,00	,00	,00
202	,00	,00	,00	,00	,00	,00
203	,00	,00	,00	,00	,00	,00
204	1,00	,00	3,00	,00	,00	,00
205	,00	,00	,00	,00	,00	,00
206	,00	,00	,00	,00	,00	,00
207	,00	,00	,00	,00	,00	,00
208	,00	1,00	2,00	,00	,00	,00
209	,00	,00	,00	,00	,00	,00
210	,00	,00	,00	,00	,00	,00
211	,00	,00	,00	,00	,00	,00
212	,00	,00	,00	,00	,00	,00
213	,00	,00	,00	,00	,00	,00
214	,00	,00	,00	,00	,00	,00
215	,00	,00	,00	,00	,00	,00
216	,00	1,00	2,00	,00	,00	,00
217	,00	,00	,00	,00	,00	,00
218	,00	,00	,00	,00	,00	,00
219	,00	,00	,00	,00	,00	,00
220	,00	,00	,00	,00	,00	1,00
221	,00	,00	,00	,00	,00	,00
222	,00	1,00	2,00	,00	,00	,00

	Thro.Emb	D.insidipus	Dys.Aphasia	Sens.Hemi	Motor.Hemi	Other.symptoms
186	,00	,00	,00	,00	,00	,00
187	,00	,00	,00	,00	,00	,00
188	,00	,00	,00	,00	,00	,00
189	,00	,00	,00	,00	,00	,00
190	,00	,00	,00	,00	,00	1,00
191	,00	,00	,00	,00	,00	,00
192	,00	,00	,00	,00	,00	,00
193	,00	,00	,00	,00	,00	,00
194	,00	,00	,00	,00	,00	,00
195	,00	,00	,00	,00	,00	1,00
196	,00	,00	,00	,00	,00	1,00
197	,00	,00	,00	,00	,00	1,00
198	,00	,00	,00	,00	1,00	,00
199	,00	,00	,00	,00	,00	,00
200	,00	,00	,00	,00	,00	,00
201	,00	,00	,00	,00	1,00	1,00
202	,00	,00	,00	,00	,00	,00
203	,00	,00	,00	,00	,00	,00
204	,00	,00	,00	,00	1,00	1,00
205	,00	,00	,00	,00	,00	,00
206	1,00	,00	,00	,00	,00	,00
207	,00	,00	,00	,00	,00	,00
208	,00	,00	,00	,00	,00	,00
209	,00	,00	,00	,00	,00	1,00
210	,00	,00	,00	,00	,00	,00
211	,00	,00	,00	,00	,00	,00
212	,00	,00	,00	,00	,00	,00
213	,00	,00	,00	,00	,00	,00
214	,00	,00	,00	,00	,00	,00
215	,00	,00	,00	,00	,00	,00
216	,00	,00	,00	,00	,00	,00
217	,00	,00	,00	,00	,00	,00
218	,00	,00	,00	,00	,00	,00
219	,00	,00	,00	,00	,00	,00
220	,00	,00	1,00	,00	,00	,00
221	,00	,00	,00	,00	,00	1,00
222	,00	,00	,00	,00	,00	,00

	ICU.stay	NCH.stay	Total.duration	Rehabilitation	Radiation	Recurrence
186	1,00	5,00	6,00	1,00	,00	,00
187	1,00	9,00	10,00	1,00	,00	,00
188	1,00	6,00	7,00	1,00	,00	,00
189	1,00	6,00	7,00	1,00	1,00	,00
190	1,00	5,00	6,00	,00	,00	,00
191	1,00	9,00	10,00	1,00	,00	,00
192	1,00	6,00	7,00	1,00	,00	1,00
193	1,00	5,00	6,00	,00	,00	,00
194	1,00	6,00	7,00	1,00	,00	,00
195	34,00	2,00	36,00	2,00	,00	,00
196	5,00	6,00	1,00	,00	,00	,00
197	20,00	7,00	27,00	3,00	,00	,00
198	1,00	17,00	18,00	2,00	,00	,00
199	1,00	5,00	6,00	1,00	,00	,00
200	1,00	7,00	8,00	1,00	,00	,00
201	5,00	10,00	15,00	1,00	,00	,00
202	2,00	8,00	10,00	1,00	1,00	,00
203	2,00	4,00	6,00	1,00	,00	,00
204	2,00	15,00	17,00	3,00	,00	,00
205	1,00	8,00	9,00	1,00	,00	,00
206	1,00	5,00	6,00	1,00	,00	,00
207	2,00	5,00	7,00	1,00	,00	,00
208	1,00	8,00	9,00	1,00	,00	,00
209	1,00	6,00	7,00	1,00	,00	,00
210	1,00	8,00	9,00	1,00	,00	,00
211	1,00	8,00	9,00	1,00	,00	,00
212	2,00	9,00	11,00	1,00	,00	,00
213	1,00	6,00	7,00	1,00	,00	,00
214	1,00	7,00	8,00	1,00	,00	,00
215	1,00	5,00	6,00	1,00	,00	,00
216	1,00	8,00	9,00	,00	,00	,00
217	1,00	8,00	9,00	1,00	,00	,00
218	1,00	5,00	6,00	1,00	,00	,00
219	1,00	5,00	6,00	,00	1,00	,00
220	2,00	9,00	11,00	1,00	,00	,00
221	12,00	7,00	19,00	3,00	,00	,00
222	1,00	5,00	6,00	1,00	,00	,00

	Recurrence1	Daeth	Karnofsky.score.pre	Karnofsky.score.post1.3.month	Karnofsky.score.post6.12.month	Difference.K3.K1
186	,00	,00	90,00	90,00	90,00	,00
187	,00	,00	70,00	70,00	70,00	,00
188	,00	,00	70,00	80,00	90,00	20,00
189	,00	,00	80,00	90,00	100,00	20,00
190	,00	,00	70,00	70,00	80,00	10,00
191	,00	,00	90,00	70,00	80,00	-10,00
192	,00	,00	70,00	90,00	70,00	,00
193	,00	,00	80,00	80,00	80,00	,00
194	,00	,00	60,00	70,00	80,00	20,00
195	,00	,00	70,00	40,00	.	.
196	,00	,00	70,00	70,00	70,00	,00
197	1,00	,00	70,00	70,00	70,00	,00
198	,00	,00	70,00	50,00	70,00	,00
199	,00	,00	80,00	80,00	80,00	,00
200	,00	,00	60,00	70,00	90,00	30,00
201	,00	,00	70,00	60,00	.	.
202	,00	,00	70,00	80,00	80,00	10,00
203	,00	,00	70,00	80,00	80,00	10,00
204	,00	,00	60,00	60,00	70,00	10,00
205	,00	,00	80,00	70,00	80,00	,00
206	,00	,00	100,00	80,00	100,00	,00
207	,00	,00	70,00	80,00	80,00	10,00
208	,00	,00	70,00	60,00	70,00	,00
209	,00	,00	90,00	80,00	80,00	-10,00
210	,00	,00	70,00	80,00	90,00	20,00
211	,00	,00	70,00	.	.	.
212	,00	,00	80,00	70,00	70,00	-10,00
213	,00	,00	80,00	90,00	90,00	10,00
214	,00	,00	60,00	70,00	.	.
215	,00	,00	70,00	70,00	.	.
216	1,00	,00	80,00	70,00	60,00	-20,00
217	,00	,00	80,00	70,00	80,00	,00
218	1,00	,00	70,00	70,00	.	.
219	,00	,00	70,00	70,00	70,00	,00
220	,00	,00	70,00	70,00	80,00	10,00
221	,00	,00	60,00	70,00	.	.
222	,00	,00	80,00	70,00	80,00	,00

	Diffenrence.K3.K2	K2K1	Agegroup1	Agegroup2
186	,00	,00	7,00	4,00
187	,00	,00	5,00	3,00
188	10,00	10,00	4,00	3,00
189	10,00	10,00	3,00	2,00
190	10,00	,00	5,00	3,00
191	10,00	-20,00	5,00	3,00
192	-20,00	20,00	2,00	2,00
193	,00	,00	6,00	4,00
194	10,00	10,00	3,00	2,00
195	.	-30,00	7,00	4,00
196	,00	,00	7,00	4,00
197	,00	,00	5,00	3,00
198	20,00	-20,00	6,00	4,00
199	,00	,00	6,00	4,00
200	20,00	10,00	2,00	2,00
201	.	-10,00	7,00	4,00
202	,00	10,00	6,00	4,00
203	,00	10,00	3,00	2,00
204	10,00	,00	6,00	4,00
205	10,00	-10,00	4,00	3,00
206	20,00	-20,00	4,00	3,00
207	,00	10,00	7,00	4,00
208	10,00	-10,00	5,00	3,00
209	,00	-10,00	3,00	2,00
210	10,00	10,00	5,00	3,00
211	.	.	6,00	4,00
212	,00	-10,00	4,00	3,00
213	,00	10,00	6,00	4,00
214	.	10,00	4,00	3,00
215	.	,00	5,00	3,00
216	-10,00	-10,00	5,00	3,00
217	10,00	-10,00	7,00	4,00
218	.	,00	7,00	4,00
219	,00	,00	6,00	4,00
220	10,00	,00	5,00	3,00
221	.	10,00	7,00	4,00
222	10,00	-10,00	6,00	4,00

	Symptoms.duration	Number.tumors	Number.symptoms
186	1,00	2,00	,00
187	1,00	2,00	1,00
188	1,00	2,00	1,00
189	4,00	1,00	1,00
190	3,00	1,00	2,00
191	1,00	,00	,00
192	3,00	,00	1,00
193	6,00	2,00	1,00
194	1,00	1,00	3,00
195	1,00	2,00	1,00
196	3,00	5,00	1,00
197	1,00	1,00	2,00
198	1,00	,00	1,00
199	1,00	2,00	,00
200	1,00	1,00	3,00
201	3,00	2,00	1,00
202	4,00	2,00	1,00
203	3,00	,00	6,00
204	2,00	,00	1,00
205	7,00	1,00	1,00
206	1,00	,00	,00
207	1,00	,00	3,00
208	6,00	2,00	2,00
209	.	,00	1,00
210	5,00	1,00	2,00
211	3,00	2,00	4,00
212	5,00	,00	3,00
213	7,00	3,00	2,00
214	1,00	,00	1,00
215	3,00	1,00	1,00
216	1,00	,00	,00
217	1,00	1,00	1,00
218	3,00	1,00	1,00
219	1,00	1,00	1,00
220	2,00	,00	2,00
221	1,00	2,00	1,00
222	2,00	2,00	1,00

	Operation.time	Volume.transfosion
186	2,00	,00
187	2,00	,00
188	3,00	,00
189	4,00	,00
190	1,00	,00
191	2,00	,00
192	4,00	,00
193	2,00	,00
194	2,00	,00
195	4,00	566,00
196	1,00	,00
197	3,00	,00
198	3,00	,00
199	2,00	,00
200	2,00	,00
201	5,00	566,00
202	3,00	,00
203	2,00	,00
204	3,00	,00
205	3,00	,00
206	3,00	,00
207	3,00	,00
208	3,00	,00
209	4,00	,00
210	5,00	,00
211	2,00	,00
212	3,00	,00
213	5,00	,00
214	4,00	,00
215	1,00	,00
216	3,00	,00
217	2,00	,00
218	3,00	,00
219	3,00	,00
220	2,00	,00
221	3,00	,00
222	1,00	,00

	Volume.transfusion.postOP	Rebleeding	ICU.stay.groups
186	,00	,00	1,00
187	,00	,00	1,00
188	,00	,00	1,00
189	,00	,00	1,00
190	,00	,00	1,00
191	,00	,00	1,00
192	,00	,00	1,00
193	,00	,00	1,00
194	,00	,00	1,00
195	,00	1,00	5,00
196	,00	,00	2,00
197	,00	,00	4,00
198	,00	,00	1,00
199	,00	1,00	1,00
200	,00	,00	1,00
201	849,00	?	?
202	,00	?	?
203	,00	?	?
204	,00	?	?
205	,00	?	?
206	,00	?	?
207	,00	?	?
208	,00	?	?
209	,00	?	?
210	,00	?	?
211	,00	?	?
212	,00	?	?
213	,00	?	?
214	,00	?	?
215	,00	?	?
216	,00	?	?
217	,00	,00	1,00
218	,00	,00	1,00
219	,00	,00	1,00
220	,00	,00	1,00
221	,00	,00	4,00
222	,00	,00	1,00

	NCH.stay.groups	Number.symptoms.postOP	Symptoms.postOP	First.symptome.groups
186	1,00	,00	,00	,00
187	2,00	,00	,00	7,00
188	1,00	,00	,00	2,00
189	1,00	,00	,00	6,00
190	1,00	1,00	1,00	1,00
191	2,00	,00	,00	,00
192	1,00	,00	,00	1,00
193	1,00	,00	,00	6,00
194	1,00	,00	,00	1,00
195	1,00	1,00	1,00	7,00
196	1,00	1,00	1,00	7,00
197	1,00	1,00	1,00	4,00
198	3,00	1,00	1,00	2,00
199	1,00	,00	,00	,00
200	1,00	,00	,00	7,00
201	2,00	2,00	1,00	2,00
202	2,00	,00	,00	5,00
203	1,00	,00	,00	1,00
204	3,00	2,00	1,00	1,00
205	2,00	,00	,00	1,00
206	1,00	1,00	1,00	,00
207	1,00	,00	,00	8,00
208	2,00	,00	,00	4,00
209	1,00	1,00	1,00	1,00
210	2,00	,00	,00	1,00
211	2,00	,00	,00	1,00
212	2,00	,00	,00	6,00
213	1,00	,00	,00	4,00
214	1,00	,00	,00	2,00
215	1,00	,00	,00	3,00
216	2,00	,00	,00	,00
217	2,00	,00	,00	9,00
218	1,00	,00	,00	1,00
219	1,00	,00	,00	2,00
220	2,00	2,00	1,00	8,00
221	1,00	1,00	1,00	2,00
222	1,00	,00	,00	2,00

	Neurological.disorder	Histology.groups	Revision.groups	Localisation.revision
186	,00	1,00	,00	1,00
187	1,00	2,00	,00	1,00
188	1,00	4,00	,00	1,00
189	1,00	1,00	,00	1,00
190	1,00	1,00	,00	1,00
191	,00	1,00	,00	1,00
192	1,00	4,00	,00	1,00
193	1,00	1,00	,00	1,00
194	1,00	5,00	,00	1,00
195	1,00	2,00	1,00	1,00
196	1,00	2,00	,00	1,00
197	1,00	1,00	,00	1,00
198	1,00	4,00	,00	1,00
199	,00	4,00	,00	1,00
200	1,00	1,00	1,00	1,00
201	1,00	5,00	,00	1,00
202	1,00	2,00	,00	1,00
203	1,00	4,00	,00	2,00
204	1,00	1,00	1,00	1,00
205	1,00	3,00	,00	1,00
206	,00	1,00	,00	1,00
207	1,00	1,00	,00	1,00
208	1,00	3,00	1,00	1,00
209	1,00	1,00	,00	2,00
210	1,00	3,00	,00	1,00
211	1,00	3,00	,00	1,00
212	1,00	3,00	,00	1,00
213	1,00	3,00	,00	1,00
214	1,00	3,00	,00	1,00
215	1,00	3,00	,00	1,00
216	,00	3,00	1,00	2,00
217	1,00	3,00	,00	1,00
218	1,00	2,00	,00	1,00
219	1,00	3,00	,00	1,00
220	1,00	3,00	,00	1,00
221	1,00	1,00	,00	1,00
222	1,00	5,00	1,00	1,00

	Masseffect.revision	Simpson.revision	antiepileptc.therapy.revision
186	,00	1,00	,00
187	1,00	1,00	,00
188	1,00	1,00	1,00
189	1,00	4,00	,00
190	,00	1,00	,00
191	,00	1,00	,00
192	1,00	1,00	,00
193	,00	1,00	,00
194	1,00	1,00	,00
195	1,00	1,00	,00
196	1,00	1,00	,00
197	,00	4,00	,00
198	,00	1,00	1,00
199	,00	1,00	,00
200	,00	1,00	,00
201	1,00	4,00	1,00
202	1,00	1,00	1,00
203	,00	1,00	,00
204	1,00	1,00	1,00
205	1,00	1,00	,00
206	,00	1,00	,00
207	1,00	1,00	1,00
208	1,00	1,00	,00
209	1,00	1,00	,00
210	1,00	1,00	,00
211	1,00	1,00	,00
212	1,00	1,00	,00
213	1,00	4,00	,00
214	,00	1,00	1,00
215	,00	1,00	1,00
216	,00	1,00	,00
217	,00	1,00	,00
218	1,00	1,00	,00
219	,00	1,00	1,00
220	1,00	1,00	1,00
221	?	?	?
222	?	?	?

	Post.revision.symptoms	Recurrence.revision	ASA.class.4	Kd_disorder
186	,00	,00	2,00	,00
187	,00	,00	3,00	,00
188	,00	,00	2,00	,00
189	,00	1,00	1,00	,00
190	,00	,00	2,00	,00
191	,00	,00	2,00	1,00
192	,00	1,00	1,00	,00
193	,00	,00	2,00	,00
194	,00	,00	1,00	,00
195	,00	,00	2,00	.
196	,00	,00	3,00	,00
197	,00	1,00	2,00	,00
198	1,00	1,00	2,00	,00
199	,00	,00	2,00	,00
200	,00	,00	2,00	,00
201	1,00	,00	2,00	.
202	,00	,00	2,00	,00
203	,00	,00	2,00	,00
204	1,00	,00	4,00	,00
205	,00	,00	2,00	,00
206	,00	,00	2,00	,00
207	,00	,00	2,00	,00
208	,00	,00	2,00	,00
209	,00	,00	1,00	1,00
210	,00	,00	2,00	,00
211	,00	,00	2,00	.
212	,00	,00	2,00	1,00
213	,00	,00	2,00	,00
214	,00	,00	3,00	.
215	,00	,00	2,00	.
216	,00	,00	2,00	1,00
217	,00	,00	2,00	,00
218	,00	,00	2,00	.
219	,00	,00	3,00	,00
220	1,00	,00	2,00	,00
221	,00	,00	2,00	.
222	,00	,00	2,00	,00

	age_disorder	K1_cut	K1_3gr	Kd_3gr	ASA_di
186	1,00	,00	3,00	2,00	,00
187	,00	,00	2,00	2,00	1,00
188	,00	,00	2,00	3,00	,00
189	,00	,00	3,00	3,00	,00
190	,00	,00	2,00	3,00	,00
191	,00	,00	3,00	1,00	,00
192	,00	,00	2,00	2,00	,00
193	1,00	,00	3,00	2,00	,00
194	,00	1,00	1,00	3,00	,00
195	1,00	,00	2,00	.	,00
196	1,00	,00	2,00	2,00	1,00
197	,00	,00	2,00	2,00	,00
198	1,00	,00	2,00	2,00	,00
199	1,00	,00	3,00	2,00	,00
200	,00	1,00	1,00	3,00	,00
201	1,00	,00	2,00	.	,00
202	1,00	,00	2,00	3,00	,00
203	,00	,00	2,00	3,00	,00
204	1,00	1,00	1,00	3,00	1,00
205	,00	,00	3,00	2,00	,00
206	,00	,00	3,00	2,00	,00
207	1,00	,00	2,00	3,00	,00
208	,00	,00	2,00	2,00	,00
209	,00	,00	3,00	1,00	,00
210	,00	,00	2,00	3,00	,00
211	1,00	,00	2,00	.	,00
212	,00	,00	3,00	1,00	,00
213	1,00	,00	3,00	3,00	,00
214	,00	1,00	1,00	.	1,00
215	,00	,00	2,00	.	,00
216	,00	,00	3,00	1,00	,00
217	1,00	,00	3,00	2,00	,00
218	1,00	,00	2,00	.	,00
219	1,00	,00	2,00	2,00	1,00
220	,00	,00	2,00	3,00	,00
221	1,00	1,00	1,00	?	?
222	1,00	,00	3,00	?	?

	Simpson_2gr	WHO_di	Age_cut	Localisation_di
186	,00	,00	1,00	,00
187	,00	,00	,00	.
188	,00	1,00	,00	,00
189	1,00	,00	,00	1,00
190	,00	,00	,00	1,00
191	,00	,00	,00	1,00
192	,00	1,00	,00	1,00
193	,00	,00	,00	1,00
194	,00	,00	,00	.
195	,00	,00	1,00	1,00
196	,00	,00	1,00	,00
197	1,00	,00	,00	.
198	,00	1,00	1,00	1,00
199	,00	1,00	1,00	1,00
200	,00	,00	,00	.
201	1,00	,00	1,00	,00
202	,00	,00	1,00	1,00
203	,00	1,00	,00	1,00
204	,00	1,00	1,00	1,00
205	,00	,00	,00	1,00
206	,00	,00	,00	1,00
207	,00	,00	1,00	,00
208	,00	,00	,00	1,00
209	,00	,00	,00	1,00
210	,00	,00	,00	.
211	,00	,00	,00	.
212	,00	,00	,00	1,00
213	1,00	,00	,00	.
214	,00	,00	,00	.
215	,00	,00	,00	,00
216	,00	,00	,00	1,00
217	,00	,00	1,00	,00
218	,00	,00	1,00	,00
219	,00	,00	1,00	1,00
220	,00	,00	,00	1,00
221	1,00	,00	1,00	1,00
222	,00	,00	,00	.

	K3_3gr	Age3gr	RF_r	Reha_r	K3_cut
186	3,00	3,00	1,00	1,00	,00
187	2,00	2,00	1,00	1,00	1,00
188	3,00	1,00	1,00	1,00	,00
189	3,00	1,00	1,00	1,00	,00
190	3,00	2,00	1,00	,00	,00
191	3,00	2,00	,00	1,00	,00
192	2,00	1,00	,00	1,00	1,00
193	3,00	2,00	1,00	,00	,00
194	3,00	1,00	1,00	1,00	,00
195	.	3,00	1,00	2,00	.
196	2,00	3,00	1,00	,00	1,00
197	2,00	2,00	1,00	2,00	1,00
198	2,00	2,00	,00	2,00	1,00
199	3,00	2,00	1,00	1,00	,00
200	3,00	1,00	1,00	1,00	,00
201	.	3,00	1,00	1,00	.
202	3,00	2,00	1,00	1,00	,00
203	3,00	1,00	,00	1,00	,00
204	2,00	2,00	,00	2,00	1,00
205	3,00	1,00	1,00	1,00	,00
206	3,00	1,00	,00	1,00	,00
207	3,00	3,00	,00	1,00	,00
208	2,00	2,00	1,00	1,00	1,00
209	3,00	1,00	,00	1,00	,00
210	3,00	2,00	1,00	1,00	,00
211	.	2,00	1,00	1,00	.
212	2,00	1,00	,00	1,00	1,00
213	3,00	2,00	1,00	1,00	,00
214	.	1,00	,00	1,00	.
215	.	2,00	1,00	1,00	.
216	1,00	2,00	,00	,00	1,00
217	3,00	3,00	1,00	1,00	,00
218	.	3,00	1,00	1,00	.
219	2,00	2,00	1,00	,00	1,00
220	3,00	2,00	,00	1,00	,00
221	.	3,00	1,00	2,00	.
222	3,00	2,00	1,00	1,00	,00

	Localisation3gr	localisation2gr	Uni_di	age7groups
186	3,00	,00	,00	6,00
187	.	.	,00	4,00
188	3,00	,00	,00	3,00
189	1,00	1,00	,00	2,00
190	2,00	.	,00	4,00
191	2,00	.	,00	4,00
192	1,00	1,00	,00	1,00
193	1,00	1,00	,00	5,00
194	.	.	,00	2,00
195	1,00	1,00	1,00	6,00
196	3,00	,00	,00	6,00
197	.	.	1,00	4,00
198	2,00	.	1,00	5,00
199	1,00	1,00	,00	5,00
200	.	.	,00	1,00
201	3,00	,00	1,00	6,00
202	2,00	.	,00	5,00
203	1,00	1,00	,00	2,00
204	1,00	1,00	1,00	5,00
205	1,00	1,00	,00	3,00
206	1,00	1,00	,00	3,00
207	3,00	,00	,00	6,00
208	2,00	.	,00	4,00
209	1,00	1,00	,00	2,00
210	.	.	,00	4,00
211	.	.	,00	5,00
212	1,00	1,00	,00	3,00
213	.	.	,00	5,00
214	.	.	,00	3,00
215	3,00	,00	,00	4,00
216	1,00	1,00	,00	4,00
217	3,00	,00	,00	6,00
218	3,00	,00	,00	6,00
219	2,00	.	,00	5,00
220	1,00	1,00	,00	4,00
221	1,00	1,00	1,00	6,00
222	.	.	,00	5,00

	Number	Sex	OP.year	Age	Histology.WHO	Type.histology
223	223,00	1,00	2015,00	74,00	1,00	1,00
224	224,00	1,00	2015,00	60,00	1,00	3,00
225	225,00	1,00	2015,00	74,00	1,00	3,00
226	226,00	1,00	2015,00	53,00	1,00	1,00
227	227,00	1,00	2015,00	50,00	1,00	3,00
228	228,00	,00	2015,00	63,00	2,00	12,00
229	229,00	1,00	2015,00	65,00	1,00	1,00
230	230,00	1,00	2015,00	61,00	1,00	3,00
231	231,00	,00	2015,00	72,00	1,00	1,00
232	232,00	,00	2015,00	71,00	1,00	1,00
233	233,00	1,00	2015,00	38,00	1,00	1,00
234	234,00	,00	2015,00	80,00	1,00	1,00
235	235,00	,00	2015,00	84,00	1,00	1,00
236	236,00	1,00	2015,00	56,00	2,00	12,00
237	237,00	1,00	2015,00	41,00	1,00	1,00
238	238,00	1,00	2015,00	58,00	1,00	4,00
239	239,00	1,00	2015,00	61,00	1,00	1,00
240	240,00	1,00	2015,00	77,00	1,00	3,00
241	241,00	1,00	2015,00	47,00	1,00	3,00
242	242,00	,00	2015,00	73,00	1,00	1,00
243	243,00	1,00	2015,00	70,00	1,00	3,00
244	244,00	,00	2015,00	33,00	1,00	1,00
245	245,00	,00	2009,00	54,00	1,00	1,00
246	246,00	,00	2009,00	72,00	2,00	12,00
247	247,00	1,00	2009,00	75,00	1,00	1,00
248	248,00	1,00	2009,00	49,00	1,00	1,00
249	249,00	1,00	2009,00	78,00	2,00	12,00
250	250,00	,00	2009,00	68,00	2,00	12,00
251	251,00	1,00	2009,00	70,00	1,00	3,00
252	252,00	,00	2009,00	56,00	2,00	12,00
253	253,00	,00	2009,00	68,00	1,00	1,00
254	254,00	1,00	2009,00	69,00	1,00	1,00
255	255,00	1,00	2009,00	33,00	1,00	3,00
256	256,00	1,00	2009,00	65,00	2,00	12,00
257	257,00	1,00	2009,00	74,00	2,00	12,00
258	258,00	1,00	2009,00	63,00	1,00	5,00
259	259,00	,00	2009,00	67,00	1,00	1,00

	Side	Localisation	Duration.of.symptoms	No.symptoms	First.symptom	Haedache
223	2,00	6,00	,00	,00	,00	,00
224	3,00	5,00	,00	,00	1,00	1,00
225	2,00	3,00	6,00	,00	15,00	1,00
226	1,00	3,00	,00	,00	21,00	,00
227	1,00	11,00	6,00	,00	15,00	,00
228	3,00	5,00	12,00	,00	11,00	1,00
229	1,00	5,00	12,00	,00	6,00	,00
230	2,00	2,00	24,00	,00	17,00	,00
231	1,00	3,00	96,00	,00	6,00	,00
232	1,00	3,00	,00	1,00	4,00	,00
233	1,00	3,00	24,00	,00	6,00	,00
234	2,00	3,00	,00	,00	16,00	,00
235	1,00	13,00	7,00	,00	17,00	,00
236	2,00	10,00	2,00	1,00	16,00	,00
237	2,00	3,00	,00	,00	7,00	,00
238	3,00	12,00	7,00	1,00	4,00	,00
239	2,00	1,00	,00	1,00	,00	,00
240	2,00	1,00	24,00	,00	13,00	,00
241	3,00	5,00	6,00	1,00	?	?
242	2,00	3,00	4,00	,00	?	?
243	3,00	1,00	18,00	1,00	?	?
244	2,00	1,00	2,00	1,00	?	?
245	1,00	1,00	,00	1,00	?	?
246	1,00	1,00	1,00	1,00	?	?
247	3,00	3,00	3,00	,00	?	?
248	2,00	2,00	8,00	,00	?	?
249	2,00	3,00	24,00	,00	?	?
250	2,00	2,00	,00	,00	?	,00
251	2,00	1,00	1,00	,00	1,00	1,00
252	2,00	2,00	72,00	,00	4,00	,00
253	3,00	10,00	,00	1,00	,00	,00
254	1,00	3,00	2,00	,00	5,00	,00
255	2,00	10,00	6,00	,00	4,00	,00
256	3,00	1,00	6,00	,00	17,00	,00
257	2,00	2,00	5,00	,00	1,00	1,00
258	2,00	1,00	,00	1,00	,00	,00
259	3,00	5,00	2,00	,00	4,00	,00

	Nausea	Emesis	Nausea_Emesis	Seizures	Oculo.paresis	Viszual.paresis
223	,00	,00	,00	,00	,00	,00
224	,00	,00	,00	,00	,00	,00
225	,00	,00	,00	,00	,00	,00
226	,00	,00	,00	,00	,00	,00
227	,00	,00	,00	,00	,00	,00
228	,00	,00	,00	,00	,00	,00
229	,00	,00	,00	,00	,00	1,00
230	,00	,00	,00	,00	,00	,00
231	,00	,00	,00	,00	,00	1,00
232	,00	,00	,00	1,00	,00	,00
233	,00	,00	,00	,00	,00	1,00
234	,00	,00	,00	,00	,00	,00
235	,00	,00	,00	,00	,00	,00
236	,00	,00	,00	,00	,00	,00
237	,00	,00	,00	,00	,00	1,00
238	,00	,00	,00	1,00	,00	,00
239	,00	,00	,00	,00	,00	,00
240	,00	,00	,00	,00	,00	,00
241	,00	,00	,00	,00	,00	,00
242	,00	,00	,00	1,00	,00	,00
243	,00	,00	,00	,00	,00	,00
244	,00	,00	,00	,00	,00	,00
245	,00	,00	,00	,00	,00	,00
246	,00	,00	,00	1,00	,00	,00
247	,00	,00	,00	1,00	,00	,00
248	,00	,00	,00	,00	,00	,00
249	,00	,00	,00	,00	,00	,00
250	,00	,00	,00	,00	,00	,00
251	,00	,00	,00	,00	,00	,00
252	,00	,00	,00	1,00	,00	,00
253	,00	,00	,00	,00	,00	,00
254	,00	,00	,00	,00	1,00	,00
255	,00	,00	,00	1,00	,00	,00
256	,00	,00	,00	,00	,00	,00
257	,00	,00	,00	,00	,00	,00
258	,00	,00	,00	,00	,00	,00
259	,00	,00	,00	1,00	,00	,00

	Viszual.deficite	Papilloedema	Optic.atrophie	Exophthalmus	Kakosmia	Other.nerve.paresis
223	,00	,00	,00	,00	,00	,00
224	,00	,00	,00	,00	1,00	1,00
225	,00	,00	,00	,00	,00	1,00
226	,00	,00	,00	,00	,00	,00
227	,00	,00	,00	,00	,00	,00
228	,00	,00	,00	,00	1,00	,00
229	,00	,00	,00	,00	1,00	,00
230	,00	,00	,00	,00	,00	,00
231	1,00	,00	,00	,00	,00	,00
232	,00	,00	,00	,00	,00	,00
233	1,00	,00	,00	,00	,00	,00
234	,00	,00	,00	,00	,00	1,00
235	,00	,00	,00	,00	,00	,00
236	,00	,00	,00	,00	,00	,00
237	1,00	,00	,00	,00	,00	,00
238	,00	,00	,00	,00	,00	,00
239	,00	,00	,00	,00	,00	,00
240	,00	,00	,00	,00	,00	,00
241	,00	,00	,00	,00	,00	,00
242	,00	,00	,00	,00	,00	,00
243	,00	,00	,00	,00	,00	,00
244	,00	,00	,00	,00	,00	,00
245	,00	,00	,00	,00	,00	,00
246	,00	,00	,00	,00	,00	,00
247	,00	,00	,00	,00	,00	,00
248	,00	,00	,00	,00	,00	,00
249	,00	,00	,00	,00	,00	,00
250	,00	,00	,00	,00	,00	,00
251	,00	,00	,00	,00	,00	,00
252	,00	,00	,00	,00	,00	,00
253	,00	,00	,00	,00	,00	,00
254	,00	,00	,00	,00	,00	1,00
255	,00	,00	,00	,00	,00	,00
256	,00	,00	,00	,00	,00	,00
257	,00	,00	,00	,00	,00	,00
258	,00	,00	,00	,00	,00	,00
259	,00	,00	,00	,00	,00	,00

	Sensibility.disorder	Motoric.disorder	Cerebellar.symptoms	Aphasia	Concentration.disoreders	Personallity.change
223	,00	,0	,00	,00	,00	,00
224	,00	,0	,00	,00	,00	,00
225	,00	,0	1,00	,00	,00	,00
226	,00	,0	,00	,00	,00	,00
227	,00	,0	1,00	,00	,00	,00
228	,00	,0	,00	,00	,00	,00
229	,00	,0	,00	,00	,00	,00
230	,00	,0	,00	,00	1,00	,00
231	,00	,0	,00	,00	,00	,00
232	,00	,0	,00	,00	,00	,00
233	,00	,0	,00	,00	,00	,00
234	,00	,0	,00	1,00	,00	,00
235	,00	,0	,00	,00	1,00	,00
236	,00	,0	,00	1,00	,00	,00
237	,00	,0	,00	,00	,00	,00
238	,00	,0	,00	,00	,00	,00
239	,00	,0	,00	,00	,00	,00
240	1,00	,0	,00	1,00	1,00	,00
241	,00	,0	,00	,00	,00	,00
242	,00	,0	,00	1,00	1,00	,00
243	,00	,0	,00	,00	1,00	1,00
244	,00	,0	,00	,00	1,00	,00
245	,00	1,0	,00	1,00	,00	,00
246	1,00	1,0	,00	,00	,00	,00
247	,00	,0	1,00	,00	,00	,00
248	,00	,0	,00	1,00	,00	,00
249	,00	,0	,00	,00	,00	,00
250	,00	,0	,00	,00	,00	,00
251	,00	1,0	,00	1,00	,00	,00
252	,00	1,0	1,00	,00	,00	,00
253	,00	,0	,00	,00	,00	,00
254	,00	,0	1,00	,00	,00	,00
255	,00	,0	,00	,00	,00	,00
256	,00	,0	,00	1,00	1,00	1,00
257	,00	,0	,00	1,00	1,00	,00
258	,00	,0	,00	,00	,00	,00
259	,00	,0	,00	,00	,00	,00

	Other.motoric.disorder	loss.counsciuoness	Double.vision	Histological.clear	Histological.unclear	Size
223	,00	,00	,00	1,00	1,00	1,00
224	,00	,00	,00	1,00	,00	1,00
225	,00	,00	,00	1,00	,00	1,00
226	,00	,00	1,00	1,00	,00	1,00
227	,00	,00	,00	1,00	,00	1,00
228	,00	,00	,00	1,00	,00	2,00
229	,00	,00	,00	1,00	,00	2,00
230	,00	,00	,00	1,00	,00	2,00
231	,00	,00	,00	1,00	,00	2,00
232	,00	1,00	,00	1,00	1,00	2,00
233	,00	,00	,00	1,00	,00	2,00
234	,00	,00	,00	1,00	,00	1,00
235	,00	,00	,00	1,00	,00	2,00
236	,00	,00	,00	1,00	,00	2,00
237	,00	,00	,00	1,00	,00	1,00
238	,00	,00	,00	1,00	,00	1,00
239	,00	,00	,00	1,00	,00	2,00
240	,00	,00	,00	1,00	,00	2,00
241	,00	,00	,00	1,00	,00	1,00
242	,00	,00	,00	1,00	,00	2,00
243	,00	,00	,00	1,00	,00	2,00
244	,00	,00	,00	1,00	,00	2,00
245	,00	,00	,00	1,00	1,00	2,00
246	,00	,00	,00	1,00	,00	2,00
247	,00	,00	,00	1,00	,00	2,00
248	,00	,00	1,00	1,00	,00	1,00
249	,00	,00	,00	1,00	2,00	1,00
250	,00	,00	,00	1,00	,00	1,00
251	1,00	,00	,00	1,00	,00	1,00
252	,00	,00	,00	1,00	,00	2,00
253	,00	,00	,00	1,00	,00	1,00
254	,00	,00	1,00	1,00	,00	1,00
255	,00	,00	,00	1,00	,00	1,00
256	,00	,00	,00	1,00	,00	2,00
257	,00	,00	,00	1,00	,00	1,00
258	,00	,00	,00	1,00	,00	1,00
259	,00	,00	,00	1,00	,00	1,00

	MRi.CCT	Form	CSF	Edema	Masseffect	Embolisation
223	2,00	1,00	,00	,00	,00	,00
224	2,00	2,00	,00	,00	,00	,00
225	2,00	1,00	,00	,00	,00	,00
226	2,00	2,00	,00	,00	,00	,00
227	2,00	1,00	,00	1,00	1,00	,00
228	2,00	2,00	,00	1,00	,00	,00
229	2,00	1,00	,00	1,00	2,00	,00
230	2,00	2,00	,00	1,00	2,00	,00
231	2,00	2,00	,00	,00	2,00	,00
232	2,00	2,00	,00	1,00	2,00	,00
233	2,00	2,00	,00	,00	2,00	,00
234	2,00	2,00	,00	1,00	,00	,00
235	2,00	2,00	,00	1,00	2,00	,00
236	2,00	1,00	,00	1,00	2,00	,00
237	2,00	2,00	,00	,00	1,00	,00
238	2,00	2,00	,00	,00	1,00	,00
239	2,00	2,00	,00	1,00	,00	,00
240	2,00	2,00	,00	1,00	,00	,00
241	2,00	1,00	,00	1,00	2,00	,00
242	2,00	2,00	,00	,00	,00	,00
243	2,00	2,00	,00	1,00	1,00	,00
244	2,00	1,00	,00	,00	,00	,00
245	2,00	1,00	1,00	1,00	2,00	,00
246	2,00	1,00	,00	1,00	,00	,00
247	2,00	1,00	,00	1,00	2,00	,00
248	2,00	1,00	,00	,00	,00	,00
249	2,00	1,00	,00	1,00	,00	,00
250	2,00	1,00	,00	,00	,00	,00
251	2,00	1,00	,00	1,00	2,00	,00
252	2,00	1,00	,00	1,00	2,00	,00
253	2,00	1,00	,00	,00	,00	,00
254	2,00	2,00	,00	,00	1,00	,00
255	2,00	1,00	,00	,00	,00	,00
256	2,00	2,00	,00	1,00	2,00	1,00
257	2,00	1,00	,00	1,00	,00	,00
258	2,00	1,00	,00	,00	,00	,00
259	2,00	1,00	,00	1,00	2,00	,00

	Hypertonia	Adipositas	Heart.disorder	Lung.disorder	Liver.disorder	Kindey.disorder
223	1,00	,00	1,00	,00	,00	,00
224	,00	,00	,00	,00	,00	,00
225	1,00	1,00	1,00	,00	,00	,00
226	1,00	,00	,00	,00	,00	,00
227	,00	,00	,00	,00	,00	,00
228	,00	,00	,00	,00	,00	,00
229	1,00	1,00	1,00	,00	1,00	,00
230	,00	,00	,00	1,00	,00	,00
231	1,00	1,00	,00	,00	,00	,00
232	1,00	1,00	,00	,00	,00	,00
233	,00	,00	,00	,00	,00	,00
234	1,00	,00	1,00	,00	,00	,00
235	,00	1,00	1,00	,00	,00	1,00
236	,00	,00	,00	,00	,00	,00
237	,00	,00	,00	,00	,00	,00
238	1,00	,00	,00	,00	,00	,00
239	,00	,00	,00	,00	,00	,00
240	,00	,00	,00	,00	,00	,00
241	,00	,00	,00	,00	,00	,00
242	1,00	,00	,00	,00	,00	1,00
243	,00	1,00	,00	,00	,00	,00
244	,00	,00	1,00	,00	,00	,00
245	,00	1,00	,00	,00	,00	,00
246	1,00	1,00	,00	,00	,00	1,00
247	,00	,00	1,00	,00	,00	,00
248	,00	,00	,00	,00	,00	,00
249	1,00	,00	,00	,00	,00	,00
250	1,00	1,00	,00	,00	,00	,00
251	1,00	1,00	,00	,00	1,00	,00
252	1,00	1,00	,00	,00	,00	,00
253	1,00	,00	,00	,00	,00	,00
254	1,00	,00	,00	,00	,00	,00
255	,00	,00	,00	,00	,00	,00
256	1,00	1,00	1,00	,00	,00	,00
257	,00	,00	,00	,00	,00	,00
258	1,00	,00	,00	,00	,00	,00
259	,00	,00	,00	,00	,00	,00

	Diabetes	Varicosis	ASA	Simpson.grade	OP.duration	intraOP.brain swelling
223	,00	,00	3,00	1,00	137,00	,00
224	,00	,00	3,00	2,00	277,00	,00
225	,00	,00	3,00	2,00	224,00	,00
226	1,00	,00	2,00	3,00	220,00	,00
227	,00	,00	1,00	1,00	207,00	,00
228	,00	,00	2,00	1,00	341,00	,00
229	1,00	,00	3,00	1,00	470,00	,00
230	,00	,00	2,00	1,00	244,00	,00
231	,00	,00	2,00	4,00	296,00	,00
232	,00	,00	2,00	2,00	504,00	1,00
233	,00	,00	1,00	4,00	461,00	1,00
234	1,00	,00	3,00	1,00	270,00	,00
235	,00	,00	2,00	1,00	167,00	,00
236	,00	,00	1,00	3,00	386,00	,00
237	,00	,00	1,00	2,00	495,00	,00
238	,00	,00	2,00	4,00	458,00	,00
239	,00	,00	2,00	2,00	272,00	,00
240	,00	,00	2,00	1,00	235,00	,00
241	,00	,00	1,00	1,00	293,00	,00
242	,00	,00	2,00	4,00	665,00	,00
243	,00	,00	2,00	2,00	601,00	,00
244	,00	,00	2,00	2,00	432,00	,00
245	,00	,00	2,00	1,00	346,00	,00
246	1,00	,00	3,00	2,00	280,00	,00
247	,00	,00	3,00	3,00	412,00	,00
248	,00	,00	2,00	1,00	115,00	,00
249	,00	,00	2,00	1,00	181,00	,00
250	,00	,00	3,00	1,00	191,00	,00
251	,00	,00	2,00	2,00	144,00	,00
252	,00	,00	2,00	2,00	239,00	,00
253	,00	,00	2,00	2,00	340,00	,00
254	,00	,00	2,00	2,00	190,00	1,00
255	,00	,00	2,00	1,00	268,00	,00
256	1,00	,00	3,00	1,00	368,00	,00
257	,00	,00	2,00	1,00	125,00	1,00
258	,00	,00	2,00	1,00	282,00	1,00
259	,00	,00	3,00	1,00	197,00	,00

	Transfusion	Use.CUSA	Craniotomy	Sinus.lesion	Bleeding	Dura.closure
223	,00	,00	1,00	,00	,00	3,00
224	,00	,00	1,00	,00	,00	2,00
225	,00	,00	1,00	,00	,00	3,00
226	,00	1,00	1,00	,00	,00	2,00
227	,00	1,00	1,00	,00	,00	1,00
228	,00	1,00	1,00	,00	,00	3,00
229	,00	1,00	1,00	,00	,00	2,00
230	,00	1,00	1,00	1,00	,00	2,00
231	,00	,00	1,00	,00	,00	3,00
232	,00	1,00	1,00	1,00	,00	3,00
233	,00	1,00	1,00	,00	,00	1,00
234	,00	,00	1,00	,00	,00	1,00
235	,00	,00	1,00	,00	,00	1,00
236	,00	1,00	1,00	,00	,00	2,00
237	,00	1,00	1,00	,00	,00	3,00
238	,00	1,00	1,00	,00	,00	3,00
239	,00	1,00	1,00	,00	,00	2,00
240	,00	1,00	2,00	,00	,00	2,00
241	,00	,00	1,00	,00	,00	1,00
242	1,00	,00	2,00	1,00	,00	3,00
243	,00	1,00	1,00	,00	,00	1,00
244	,00	1,00	1,00	,00	1,00	3,00
245	2,00	1,00	1,00	,00	,00	2,00
246	,00	,00	1,00	1,00	,00	2,00
247	,00	1,00	1,00	,00	,00	1,00
248	,00	,00	1,00	,00	,00	2,00
249	2,00	,00	1,00	,00	,00	2,00
250	,00	,00	2,00	,00	,00	2,00
251	,00	,00	1,00	1,00	,00	2,00
252	,00	1,00	2,00	,00	,00	3,00
253	,00	1,00	1,00	,00	,00	3,00
254	,00	,00	1,00	,00	,00	1,00
255	,00	,00	1,00	,00	,00	3,00
256	,00	1,00	1,00	,00	,00	3,00
257	,00	1,00	2,00	,00	,00	1,00
258	,00	,00	1,00	,00	,00	1,00
259	,00	1,00	1,00	,00	,00	3,00

	Tachosil.Fibrin	Transfusion.postOP	Seizure.therapy	Antibiotics.postOP	CSF.circulatory.disorder	Edema.postOP
223	1,00	,00	1,00	,00	,00	,00
224	1,00	,00	1,00	,00	,00	,00
225	1,00	,00	,00	,00	,00	,00
226	1,00	,00	,00	,00	,00	,00
227	1,00	,00	,00	,00	,00	,00
228	1,00	,00	,00	,00	,00	,00
229	1,00	,00	,00	,00	,00	,00
230	1,00	,00	1,00	,00	,00	,00
231	1,00	,00	,00	,00	,00	,00
232	1,00	,00	,00	,00	,00	,00
233	1,00	,00	,00	,00	,00	1,00
234	1,00	,00	2,00	,00	,00	1,00
235	1,00	,00	,00	,00	,00	,00
236	1,00	,00	,00	,00	,00	,00
237	1,00	,00	,00	,00	,00	,00
238	1,00	,00	,00	,00	,00	,00
239	1,00	,00	,00	,00	,00	,00
240	1,00	,00	,00	,00	,00	,00
241	1,00	,00	,00	,00	,00	1,00
242	1,00	,00	1,00	,00	,00	1,00
243	1,00	,00	,00	,00	,00	,00
244	1,00	,00	,00	,00	,00	,00
245	1,00	,00	,00	,00	,00	,00
246	1,00	,00	1,00	1,00	,00	1,00
247	1,00	,00	1,00	,00	,00	1,00
248	1,00	,00	,00	,00	,00	,00
249	1,00	,00	,00	,00	,00	,00
250	1,00	,00	,00	,00	,00	,00
251	1,00	,00	,00	,00	,00	,00
252	1,00	,00	,00	,00	,00	,00
253	1,00	,00	,00	,00	,00	,00
254	1,00	,00	,00	,00	,00	,00
255	1,00	,00	,00	,00	,00	,00
256	1,00	,00	,00	,00	,00	,00
257	1,00	,00	,00	,00	,00	,00
258	1,00	,00	,00	,00	,00	,00
259	1,00	,00	1,00	,00	,00	,00

	Kind.of.bleeding	Infection	Revision1	Revision2	Revision3	Seizures.postOP
223	,00	,00	,00	,00	,00	,00
224	,00	,00	,00	,00	,00	,00
225	2,00	,00	3,00	,00	,00	,00
226	,00	,00	,00	,00	,00	,00
227	,00	,00	,00	,00	,00	,00
228	,00	,00	,00	,00	,00	,00
229	,00	,00	,00	,00	,00	,00
230	2,00	,00	1,00	,00	,00	,00
231	,00	,00	1,00	,00	,00	,00
232	3,00	,00	,00	,00	,00	,00
233	3,00	,00	3,00	1,00	,00	,00
234	,00	,00	1,00	,00	,00	1,00
235	,00	,00	,00	,00	,00	,00
236	,00	,00	,00	,00	,00	,00
237	,00	,00	,00	,00	,00	,00
238	,00	,00	,00	,00	,00	,00
239	,00	,00	,00	,00	,00	,00
240	,00	,00	,00	,00	,00	,00
241	,00	,00	,00	,00	,00	,00
242	,00	,00	,00	,00	,00	,00
243	,00	,00	,00	,00	,00	,00
244	,00	,00	,00	,00	,00	,00
245	,00	,00	,00	,00	,00	,00
246	,00	1,00	2,00	,00	,00	,00
247	,00	,00	,00	,00	,00	,00
248	,00	,00	,00	,00	,00	,00
249	,00	,00	,00	,00	,00	,00
250	,00	,00	,00	,00	,00	,00
251	,00	,00	,00	,00	,00	,00
252	,00	,00	,00	,00	,00	,00
253	,00	,00	,00	,00	,00	,00
254	,00	,00	,00	,00	,00	,00
255	,00	,00	,00	,00	,00	,00
256	,00	,00	2,00	,00	,00	,00
257	,00	,00	,00	,00	,00	,00
258	,00	,00	,00	,00	,00	1,00
259	2,00	,00	3,00	,00	,00	,00

	Thro.Emb	D.insidipus	Dys.Aphasia	Sens.Hemi	Motor.Hemi	Other.symptoms
223	,00	,00	,00	,00	,00	,00
224	,00	,00	,00	,00	,00	,00
225	,00	,00	,00	,00	,00	,00
226	,00	,00	,00	,00	,00	,00
227	,00	,00	,00	,00	,00	,00
228	,00	,00	,00	,00	,00	,00
229	,00	,00	,00	,00	,00	,00
230	,00	,00	,00	,00	,00	,00
231	,00	,00	,00	,00	,00	,00
232	,00	,00	,00	,00	,00	,00
233	,00	1,00	,00	,00	,00	,00
234	,00	,00	1,00	,00	1,00	,00
235	,00	,00	,00	,00	,00	,00
236	,00	,00	,00	,00	,00	,00
237	,00	,00	,00	,00	,00	,00
238	,00	,00	,00	,00	,00	1,00
239	,00	,00	,00	,00	,00	,00
240	,00	,00	,00	,00	,00	1,00
241	,00	,00	,00	,00	,00	1,00
242	,00	,00	,00	,00	1,00	1,00
243	,00	,00	,00	,00	,00	,00
244	,00	,00	,00	,00	,00	,00
245	,00	,00	,00	,00	,00	1,00
246	,00	,00	,00	,00	1,00	,00
247	,00	,00	1,00	,00	1,00	,00
248	,00	,00	,00	,00	,00	,00
249	,00	,00	,00	,00	,00	1,00
250	,00	,00	,00	,00	,00	,00
251	,00	,00	,00	,00	,00	,00
252	,00	,00	,00	,00	,00	,00
253	,00	,00	,00	,00	,00	,00
254	,00	,00	,00	,00	,00	,00
255	,00	,00	,00	,00	,00	,00
256	1,00	,00	,00	,00	,00	1,00
257	,00	,00	,00	,00	,00	,00
258	,00	,00	,00	,00	,00	,00
259	,00	,00	,00	,00	,00	,00

	ICU.stay	NCH.stay	Total.duration	Rehabilitation	Radiation	Recurrence
223	1,00	6,00	7,00	,00	1,00	,00
224	1,00	7,00	8,00	1,00	,00	,00
225	1,00	9,00	10,00	1,00	,00	,00
226	1,00	6,00	7,00	1,00	,00	,00
227	1,00	7,00	8,00	1,00	,00	,00
228	1,00	8,00	9,00	1,00	,00	,00
229	4,00	14,00	18,00	1,00	,00	,00
230	3,00	4,00	7,00	1,00	,00	,00
231	1,00	9,00	10,00	1,00	1,00	,00
232	5,00	18,00	23,00	,00	1,00	,00
233	1,00	3,00	4,00	2,00	,00	,00
234	4,00	27,00	31,00	3,00	,00	,00
235	2,00	16,00	18,00	3,00	,00	,00
236	1,00	22,00	23,00	1,00	1,00	,00
237	1,00	6,00	7,00	,00	,00	,00
238	3,00	13,00	16,00	3,00	,00	,00
239	1,00	19,00	25,00	1,00	,00	,00
240	1,00	9,00	10,00	1,00	,00	,00
241	4,00	4,00	8,00	1,00	,00	,00
242	29,00	4,00	33,00	2,00	1,00	,00
243	10,00	25,00	35,00	3,00	,00	,00
244	1,00	7,00	8,00	1,00	,00	,00
245	13,00	,00	13,00	,00	,00	,00
246	2,00	30,00	32,00	3,00	,00	,00
247	6,00	10,00	16,00	3,00	,00	,00
248	1,00	5,00	6,00	,00	,00	,00
249	1,00	5,00	6,00	1,00	,00	,00
250	1,00	4,00	5,00	,00	,00	,00
251	1,00	6,00	7,00	1,00	,00	,00
252	1,00	7,00	8,00	,00	,00	,00
253	1,00	9,00	10,00	1,00	,00	,00
254	1,00	8,00	9,00	1,00	,00	,00
255	1,00	5,00	6,00	1,00	,00	,00
256	39,00	2,00	53,00	,00	,00	,00
257	1,00	9,00	10,00	1,00	,00	,00
258	1,00	6,00	7,00	,00	,00	,00
259	2,00	6,00	8,00	1,00	,00	,00

	Recurrence1	Daeth	Karnofsky.score.pre	Karnofsky.score.post1.3.month	Karnofsky.score.post6.12.month	Difference.K3.K1
223	1,00	,00	60,00	60,00	60,00	,00
224	1,00	,00	70,00	70,00	80,00	10,00
225	,00	,00	70,00	70,00	.	.
226	1,00	,00	70,00	70,00	70,00	,00
227	,00	,00	70,00	70,00	70,00	,00
228	,00	,00	80,00	70,00	70,00	-10,00
229	,00	,00	70,00	80,00	80,00	10,00
230	,00	,00	70,00	70,00	80,00	10,00
231	1,00	,00	70,00	70,00	70,00	,00
232	,00	,00	50,00	70,00	.	.
233	,00	,00	70,00	70,00	70,00	,00
234	,00	,00	50,00	60,00	50,00	,00
235	,00	,00	50,00	50,00	60,00	10,00
236	,00	,00	70,00	70,00	.	.
237	1,00	,00	80,00	90,00	90,00	10,00
238	,00	,00	80,00	70,00	70,00	-10,00
239	,00	,00	70,00	60,00	70,00	,00
240	,00	,00	70,00	70,00	70,00	,00
241	,00	,00	80,00	80,00	90,00	10,00
242	1,00	,00	70,00	50,00	.	.
243	,00	,00	50,00	50,00	.	.
244	,00	,00	70,00	70,00	.	.
245	,00	,00	40,00	60,00	.	.
246	,00	,00	60,00	50,00	50,00	-10,00
247	,00	,00	70,00	60,00	.	.
248	,00	,00	70,00	80,00	80,00	10,00
249	,00	,00	70,00	.	.	.
250	,00	,00	70,00	70,00	70,00	,00
251	,00	,00	60,00	70,00	70,00	10,00
252	,00	,00	60,00	70,00	.	.
253	,00	,00	80,00	80,00	90,00	10,00
254	,00	,00	70,00	70,00	70,00	,00
255	,00	,00	80,00	70,00	.	.
256	,00	1,00	60,00	.	.	.
257	,00	,00	70,00	70,00	70,00	,00
258	,00	,00	80,00	70,00	80,00	,00
259	,00	,00	70,00	70,00	80,00	10,00

	Diffenrence.K 3.K2	K2K1	Agegroup1	Agegroup2
223	,00	,00	7,00	4,00
224	10,00	,00	5,00	3,00
225	.	,00	7,00	4,00
226	,00	,00	5,00	3,00
227	,00	,00	4,00	3,00
228	,00	-10,00	6,00	4,00
229	,00	10,00	6,00	4,00
230	10,00	,00	6,00	4,00
231	,00	,00	7,00	4,00
232	.	20,00	7,00	4,00
233	,00	,00	3,00	2,00
234	-10,00	10,00	7,00	4,00
235	.	.	8,00	5,00
236	.	,00	5,00	3,00
237	,00	10,00	4,00	3,00
238	,00	-10,00	5,00	3,00
239	10,00	-10,00	6,00	4,00
240	,00	,00	7,00	4,00
241	10,00	,00	4,00	3,00
242	.	-20,00	7,00	4,00
243	.	,00	6,00	4,00
244	.	,00	3,00	2,00
245	.	20,00	5,00	3,00
246	,00	-10,00	7,00	4,00
247	.	-10,00	7,00	4,00
248	,00	10,00	4,00	3,00
249	.	.	7,00	4,00
250	,00	,00	6,00	4,00
251	,00	10,00	6,00	4,00
252	.	10,00	5,00	3,00
253	10,00	,00	6,00	4,00
254	,00	,00	6,00	4,00
255	.	-10,00	3,00	2,00
256	.	.	6,00	4,00
257	,00	,00	7,00	4,00
258	10,00	-10,00	6,00	4,00
259	10,00	,00	6,00	4,00

	Symptoms.duration	Number.tumors	Number.symptoms
223	1,00	2,00	,00
224	1,00	,00	3,00
225	3,00	3,00	3,00
226	1,00	2,00	1,00
227	3,00	,00	1,00
228	4,00	,00	2,00
229	4,00	5,00	2,00
230	5,00	1,00	1,00
231	7,00	2,00	2,00
232	1,00	2,00	2,00
233	5,00	,00	2,00
234	1,00	3,00	2,00
235	4,00	3,00	1,00
236	2,00	,00	1,00
237	1,00	,00	2,00
238	4,00	1,00	1,00
239	1,00	,00	,00
240	5,00	,00	3,00
241	3,00	,00	1,00
242	3,00	2,00	3,00
243	5,00	1,00	2,00
244	2,00	1,00	1,00
245	1,00	1,00	2,00
246	1,00	4,00	3,00
247	2,00	1,00	2,00
248	4,00	,00	3,00
249	5,00	1,00	,00
250	1,00	2,00	,00
251	1,00	3,00	4,00
252	7,00	2,00	3,00
253	1,00	1,00	,00
254	2,00	1,00	4,00
255	3,00	,00	1,00
256	3,00	4,00	3,00
257	3,00	,00	3,00
258	1,00	1,00	,00
259	2,00	,00	1,00

	Operation.time	Volume.transfosion
223	2,00	,00
224	3,00	,00
225	2,00	,00
226	2,00	,00
227	2,00	,00
228	3,00	,00
229	4,00	,00
230	3,00	,00
231	3,00	,00
232	5,00	,00
233	4,00	,00
234	3,00	,00
235	2,00	,00
236	4,00	,00
237	5,00	,00
238	4,00	,00
239	3,00	,00
240	2,00	,00
241	3,00	,00
242	5,00	283,00
243	5,00	,00
244	4,00	,00
245	3,00	566,00
246	3,00	,00
247	4,00	,00
248	1,00	,00
249	2,00	566,00
250	2,00	,00
251	2,00	,00
252	2,00	,00
253	3,00	,00
254	2,00	,00
255	3,00	,00
256	4,00	,00
257	2,00	,00
258	3,00	,00
259	2,00	,00

	Volume.transfusion.postOP	Rebleeding	ICU.stay.groups
223	,00	,00	1,00
224	,00	,00	1,00
225	,00	1,00	1,00
226	,00	,00	1,00
227	,00	,00	1,00
228	,00	,00	1,00
229	,00	,00	2,00
230	,00	1,00	2,00
231	,00	,00	1,00
232	,00	1,00	2,00
233	,00	1,00	1,00
234	,00	,00	2,00
235	,00	,00	1,00
236	,00	,00	1,00
237	,00	,00	1,00
238	,00	,00	2,00
239	,00	,00	1,00
240	,00	,00	1,00
241	,00	?	?
242	,00	?	?
243	,00	?	?
244	,00	?	?
245	,00	?	?
246	,00	?	?
247	,00	?	?
248	,00	?	?
249	,00	?	?
250	,00	?	?
251	,00	?	?
252	,00	?	?
253	,00	?	?
254	,00	?	?
255	,00	?	?
256	,00	?	?
257	,00	?	?
258	,00	?	?
259	,00	?	?

	NCH.stay.groups	Number.symptoms.postOP	Symptoms.postOP	First.symptome.groups
223	1,00	,00	,00	,00
224	1,00	,00	,00	1,00
225	2,00	,00	,00	7,00
226	1,00	,00	,00	4,00
227	1,00	,00	,00	7,00
228	2,00	,00	,00	6,00
229	2,00	,00	,00	4,00
230	1,00	,00	,00	3,00
231	2,00	,00	,00	4,00
232	3,00	,00	,00	2,00
233	1,00	1,00	1,00	4,00
234	4,00	3,00	1,00	8,00
235	3,00	,00	,00	3,00
236	4,00	,00	,00	8,00
237	1,00	,00	,00	4,00
238	2,00	1,00	1,00	2,00
239	3,00	,00	,00	,00
240	2,00	1,00	1,00	5,00
241	?	?	?	?
242	?	?	?	?
243	?	?	?	?
244	?	?	?	?
245	?	?	?	?
246	?	?	?	?
247	?	?	?	?
248	?	?	?	?
249	?	?	?	?
250	?	?	?	?
251	?	?	?	?
252	?	?	?	?
253	?	?	?	?
254	?	?	?	?
255	?	?	?	?
256	?	?	?	?
257	?	?	?	?
258	?	?	?	?
259	?	?	?	?

	Neurological.disorder	Histology.groups	Revision.groups	Localisation.revision
223	,00	1,00	,00	1,00
224	1,00	3,00	,00	2,00
225	1,00	3,00	1,00	1,00
226	1,00	1,00	,00	1,00
227	1,00	3,00	,00	1,00
228	1,00	4,00	,00	2,00
229	1,00	1,00	,00	1,00
230	1,00	3,00	1,00	1,00
231	1,00	1,00	1,00	1,00
232	1,00	1,00	,00	1,00
233	1,00	1,00	1,00	1,00
234	1,00	1,00	1,00	1,00
235	1,00	1,00	,00	1,00
236	1,00	4,00	,00	1,00
237	1,00	1,00	,00	1,00
238	1,00	5,00	,00	2,00
239	,00	1,00	,00	1,00
240	1,00	3,00	,00	1,00
241	?	?	?	?
242	?	?	?	?
243	?	?	?	?
244	?	?	?	?
245	?	?	?	?
246	?	?	?	?
247	?	?	?	?
248	?	?	?	?
249	?	?	?	?
250	?	?	?	?
251	?	?	?	?
252	?	?	?	?
253	?	?	?	?
254	?	?	?	?
255	?	?	?	?
256	?	?	?	?
257	?	?	?	?
258	?	?	?	?
259	?	?	?	?

	Masseffect.revision	Simpson.revision	antiepileptc.therapy.revision
223	,00	1,00	1,00
224	,00	1,00	1,00
225	,00	1,00	,00
226	,00	1,00	,00
227	1,00	1,00	,00
228	,00	1,00	,00
229	1,00	1,00	,00
230	1,00	1,00	1,00
231	1,00	4,00	,00
232	1,00	1,00	,00
233	1,00	4,00	,00
234	,00	1,00	1,00
235	1,00	1,00	,00
236	1,00	1,00	,00
237	1,00	1,00	,00
238	1,00	4,00	,00
239	,00	1,00	,00
240	,00	1,00	,00
241	?	?	?
242	?	?	?
243	?	?	?
244	?	?	?
245	?	?	?
246	?	?	?
247	?	?	?
248	?	?	?
249	?	?	?
250	?	?	?
251	?	?	?
252	?	?	?
253	?	?	?
254	?	?	?
255	?	?	?
256	?	?	?
257	?	?	?
258	?	?	?
259	?	?	?

	Post.revision.symptoms	Recurrence.revision	ASA.class.4	Kd_disorder
223	,00	,00	3,00	,00
224	,00	,00	3,00	,00
225	,00	,00	3,00	.
226	,00	,00	2,00	,00
227	,00	,00	1,00	,00
228	,00	,00	2,00	1,00
229	,00	,00	3,00	,00
230	,00	,00	2,00	,00
231	,00	,00	2,00	,00
232	,00	,00	2,00	.
233	,00	,00	1,00	,00
234	1,00	,00	3,00	,00
235	,00	,00	2,00	,00
236	,00	,00	1,00	.
237	,00	,00	1,00	,00
238	,00	,00	2,00	1,00
239	,00	,00	2,00	,00
240	,00	,00	2,00	,00
241	?	?	?	?
242	?	?	?	?
243	?	?	?	?
244	?	?	?	?
245	?	?	?	?
246	?	?	?	?
247	?	?	?	?
248	?	?	?	?
249	?	?	?	?
250	?	?	?	?
251	?	?	?	?
252	?	?	?	?
253	?	?	?	?
254	?	?	?	?
255	?	?	?	?
256	?	?	?	?
257	?	?	?	?
258	?	?	?	?
259	?	?	?	?

	age_disorder	K1_cut	K1_3gr	Kd_3gr	ASA_di
223	1,00	1,00	1,00	2,00	1,00
224	,00	,00	2,00	3,00	1,00
225	1,00	,00	2,00	.	1,00
226	,00	,00	2,00	2,00	,00
227	,00	,00	2,00	2,00	,00
228	1,00	,00	3,00	1,00	,00
229	1,00	,00	2,00	3,00	1,00
230	1,00	,00	2,00	3,00	,00
231	1,00	,00	2,00	2,00	,00
232	1,00	1,00	1,00	.	,00
233	,00	,00	2,00	2,00	,00
234	1,00	1,00	1,00	2,00	1,00
235	1,00	1,00	1,00	3,00	,00
236	,00	,00	2,00	.	,00
237	,00	,00	3,00	3,00	,00
238	,00	,00	3,00	1,00	,00
239	1,00	,00	2,00	2,00	,00
240	1,00	,00	2,00	2,00	,00
241	?	?	?	?	?
242	?	?	?	?	?
243	?	?	?	?	?
244	?	?	?	?	?
245	?	?	?	?	?
246	?	?	?	?	?
247	?	?	?	?	?
248	?	?	?	?	?
249	?	?	?	?	?
250	?	?	?	?	?
251	?	?	?	?	?
252	?	?	?	?	?
253	?	?	?	?	?
254	?	?	?	?	?
255	?	?	?	?	?
256	?	?	?	?	?
257	?	?	?	?	?
258	?	?	?	?	?
259	?	?	?	?	?

	Simpson_2gr	WHO_di	Age_cut	Localisation_di
223	,00	,00	1,00	.
224	,00	,00	,00	1,00
225	,00	,00	1,00	1,00
226	1,00	,00	,00	1,00
227	,00	,00	,00	1,00
228	,00	1,00	,00	1,00
229	,00	,00	1,00	1,00
230	,00	,00	,00	,00
231	1,00	,00	1,00	1,00
232	,00	,00	1,00	1,00
233	1,00	,00	,00	1,00
234	,00	,00	1,00	1,00
235	,00	,00	1,00	.
236	1,00	1,00	,00	1,00
237	,00	,00	,00	1,00
238	1,00	,00	,00	.
239	,00	,00	,00	1,00
240	,00	,00	1,00	1,00
241	?	?	?	?
242	?	?	?	?
243	?	?	?	?
244	?	?	?	?
245	?	?	?	?
246	?	?	?	?
247	?	?	?	?
248	?	?	?	?
249	?	?	?	?
250	?	?	?	?
251	?	?	?	?
252	?	?	?	?
253	?	?	?	?
254	?	?	?	?
255	?	?	?	?
256	?	?	?	?
257	?	?	?	?
258	?	?	?	?
259	?	?	?	?

	K3_3gr	Age3gr	RF_r	Reha_r	K3_cut
223	1,00	3,00	1,00	,00	1,00
224	3,00	2,00	,00	1,00	,00
225	.	3,00	1,00	1,00	.
226	2,00	2,00	1,00	1,00	1,00
227	2,00	2,00	,00	1,00	1,00
228	2,00	2,00	,00	1,00	1,00
229	3,00	2,00	1,00	1,00	,00
230	3,00	2,00	1,00	1,00	,00
231	2,00	3,00	1,00	1,00	1,00
232	.	3,00	1,00	,00	.
233	2,00	1,00	,00	2,00	1,00
234	1,00	3,00	1,00	2,00	1,00
235	1,00	3,00	1,00	2,00	1,00
236	.	2,00	,00	1,00	.
237	3,00	1,00	,00	,00	,00
238	2,00	2,00	1,00	2,00	1,00
239	2,00	2,00	,00	1,00	1,00
240	2,00	3,00	,00	1,00	1,00
241	?	?	?	?	?
242	?	?	?	?	?
243	?	?	?	?	?
244	?	?	?	?	?
245	?	?	?	?	?
246	?	?	?	?	?
247	?	?	?	?	?
248	?	?	?	?	?
249	?	?	?	?	?
250	?	?	?	?	?
251	?	?	?	?	?
252	?	?	?	?	?
253	?	?	?	?	?
254	?	?	?	?	?
255	?	?	?	?	?
256	?	?	?	?	?
257	?	?	?	?	?
258	?	?	?	?	?
259	?	?	?	?	?

	Localisation3gr	localisation2gr	Uni_di	age7groups
223	.	.	,00	6,00
224	1,00	1,00	,00	4,00
225	1,00	1,00	,00	6,00
226	1,00	1,00	,00	4,00
227	1,00	1,00	,00	3,00
228	1,00	1,00	,00	5,00
229	1,00	1,00	1,00	5,00
230	3,00	,00	,00	5,00
231	1,00	1,00	,00	6,00
232	1,00	1,00	1,00	6,00
233	1,00	1,00	,00	2,00
234	1,00	1,00	1,00	6,00
235	.	.	1,00	7,00
236	1,00	1,00	1,00	4,00
237	1,00	1,00	,00	3,00
238	.	.	1,00	4,00
239	2,00	.	1,00	5,00
240	2,00	.	,00	6,00
241	?	?	?	?
242	?	?	?	?
243	?	?	?	?
244	?	?	?	?
245	?	?	?	?
246	?	?	?	?
247	?	?	?	?
248	?	?	?	?
249	?	?	?	?
250	?	?	?	?
251	?	?	?	?
252	?	?	?	?
253	?	?	?	?
254	?	?	?	?
255	?	?	?	?
256	?	?	?	?
257	?	?	?	?
258	?	?	?	?
259	?	?	?	?

	Number	Sex	OP.year	Age	Histology.WHO	Type.histology
260	260,00	,00	2009,00	41,00	1,00	1,00
261	?	?	?	?	?	?
262	?	?	?	?	?	?
263	?	?	?	?	?	?
264	?	?	?	?	?	?
265	?	?	?	?	?	?
266	?	?	?	?	?	?
267	?	?	?	?	?	?
268	?	?	?	?	?	?
269	?	?	?	?	?	?
270	?	?	?	?	?	?
271	?	?	?	?	?	?
272	?	?	?	?	?	?
273	?	?	?	?	?	?
274	?	?	?	?	?	?
275	?	?	?	?	?	?
276	?	?	?	?	?	?
277	?	?	?	?	?	?
278	?	?	?	?	?	?
279	?	?	?	?	?	?
280	?	?	?	?	?	?
281	?	?	?	?	?	?
282	?	?	?	?	?	?
283	?	?	?	?	?	?
284	?	?	?	?	?	?
285	?	?	?	?	?	?
286	?	?	?	?	?	?
287	?	?	?	?	?	?
288	?	?	?	?	?	?
289	?	?	?	?	?	?
290	?	?	?	?	?	?
291	?	?	?	?	?	?
292	?	?	?	?	?	?
293	?	?	?	?	?	?
294	?	?	?	?	?	?

	Side	Localisation	Duration.of.symptoms	No.symptoms	First.symptom	Haedache
260	3,00	4,00	2,00	,00	7,00	,00
261	?	?	?	?	?	?
262	?	?	?	?	?	?
263	?	?	?	?	?	?
264	?	?	?	?	?	?
265	?	?	?	?	?	?
266	?	?	?	?	?	?
267	?	?	?	?	?	?
268	?	?	?	?	?	?
269	?	?	?	?	?	?
270	?	?	?	?	?	?
271	?	?	?	?	?	?
272	?	?	?	?	?	?
273	?	?	?	?	?	?
274	?	?	?	?	?	?
275	?	?	?	?	?	?
276	?	?	?	?	?	?
277	?	?	?	?	?	?
278	?	?	?	?	?	?
279	?	?	?	?	?	?
280	?	?	?	?	?	?
281	?	?	?	?	?	?
282	?	?	?	?	?	?
283	?	?	?	?	?	?
284	?	?	?	?	?	?
285	?	?	?	?	?	?
286	?	?	?	?	?	?
287	?	?	?	?	?	?
288	?	?	?	?	?	?
289	?	?	?	?	?	?
290	?	?	?	?	?	?
291	?	?	?	?	?	?
292	?	?	?	?	?	?
293	?	?	?	?	?	?
294	?	?	?	?	?	?

	Nausea	Emesis	Nausea_Emesis	Seizures	Oculo.paresis	Viszual.paresis
260	,00	,00	,00	1,00	,00	,00
261	?	?	?	?	?	?
262	?	?	?	?	?	?
263	?	?	?	?	?	?
264	?	?	?	?	?	?
265	?	?	?	?	?	?
266	?	?	?	?	?	?
267	?	?	?	?	?	?
268	?	?	?	?	?	?
269	?	?	?	?	?	?
270	?	?	?	?	?	?
271	?	?	?	?	?	?
272	?	?	?	?	?	?
273	?	?	?	?	?	?
274	?	?	?	?	?	?
275	?	?	?	?	?	?
276	?	?	?	?	?	?
277	?	?	?	?	?	?
278	?	?	?	?	?	?
279	?	?	?	?	?	?
280	?	?	?	?	?	?
281	?	?	?	?	?	?
282	?	?	?	?	?	?
283	?	?	?	?	?	?
284	?	?	?	?	?	?
285	?	?	?	?	?	?
286	?	?	?	?	?	?
287	?	?	?	?	?	?
288	?	?	?	?	?	?
289	?	?	?	?	?	?
290	?	?	?	?	?	?
291	?	?	?	?	?	?
292	?	?	?	?	?	?
293	?	?	?	?	?	?
294	?	?	?	?	?	?

	Viszual.deficite	Papilloedema	Optic.atrophie	Exophthalmus	Kakosmia	Other.nerve.paresis
260	1,00	,00	,00	,00	,00	,00
261	?	?	?	?	?	?
262	?	?	?	?	?	?
263	?	?	?	?	?	?
264	?	?	?	?	?	?
265	?	?	?	?	?	?
266	?	?	?	?	?	?
267	?	?	?	?	?	?
268	?	?	?	?	?	?
269	?	?	?	?	?	?
270	?	?	?	?	?	?
271	?	?	?	?	?	?
272	?	?	?	?	?	?
273	?	?	?	?	?	?
274	?	?	?	?	?	?
275	?	?	?	?	?	?
276	?	?	?	?	?	?
277	?	?	?	?	?	?
278	?	?	?	?	?	?
279	?	?	?	?	?	?
280	?	?	?	?	?	?
281	?	?	?	?	?	?
282	?	?	?	?	?	?
283	?	?	?	?	?	?
284	?	?	?	?	?	?
285	?	?	?	?	?	?
286	?	?	?	?	?	?
287	?	?	?	?	?	?
288	?	?	?	?	?	?
289	?	?	?	?	?	?
290	?	?	?	?	?	?
291	?	?	?	?	?	?
292	?	?	?	?	?	?
293	?	?	?	?	?	?
294	?	?	?	?	?	?

	Sensibility.disorder	Motoric.disorder	Cerebellar.symptoms	Aphasia	Concentration.disoreders	Personallity.change
260	,00	,0	,00	,00	,00	,00
261	?	?	?	?	?	?
262	?	?	?	?	?	?
263	?	?	?	?	?	?
264	?	?	?	?	?	?
265	?	?	?	?	?	?
266	?	?	?	?	?	?
267	?	?	?	?	?	?
268	?	?	?	?	?	?
269	?	?	?	?	?	?
270	?	?	?	?	?	?
271	?	?	?	?	?	?
272	?	?	?	?	?	?
273	?	?	?	?	?	?
274	?	?	?	?	?	?
275	?	?	?	?	?	?
276	?	?	?	?	?	?
277	?	?	?	?	?	?
278	?	?	?	?	?	?
279	?	?	?	?	?	?
280	?	?	?	?	?	?
281	?	?	?	?	?	?
282	?	?	?	?	?	?
283	?	?	?	?	?	?
284	?	?	?	?	?	?
285	?	?	?	?	?	?
286	?	?	?	?	?	?
287	?	?	?	?	?	?
288	?	?	?	?	?	?
289	?	?	?	?	?	?
290	?	?	?	?	?	?
291	?	?	?	?	?	?
292	?	?	?	?	?	?
293	?	?	?	?	?	?
294	?	?	?	?	?	?

	Other.motoric.disorder	loss.counsciuoness	Double.vision	Histological.clear	Histological.unclear	Size
260	,00	,00	,00	1,00	,00	1,00
261	?	?	?	?	?	?
262	?	?	?	?	?	?
263	?	?	?	?	?	?
264	?	?	?	?	?	?
265	?	?	?	?	?	?
266	?	?	?	?	?	?
267	?	?	?	?	?	?
268	?	?	?	?	?	?
269	?	?	?	?	?	?
270	?	?	?	?	?	?
271	?	?	?	?	?	?
272	?	?	?	?	?	?
273	?	?	?	?	?	?
274	?	?	?	?	?	?
275	?	?	?	?	?	?
276	?	?	?	?	?	?
277	?	?	?	?	?	?
278	?	?	?	?	?	?
279	?	?	?	?	?	?
280	?	?	?	?	?	?
281	?	?	?	?	?	?
282	?	?	?	?	?	?
283	?	?	?	?	?	?
284	?	?	?	?	?	?
285	?	?	?	?	?	?
286	?	?	?	?	?	?
287	?	?	?	?	?	?
288	?	?	?	?	?	?
289	?	?	?	?	?	?
290	?	?	?	?	?	?
291	?	?	?	?	?	?
292	?	?	?	?	?	?
293	?	?	?	?	?	?
294	?	?	?	?	?	?

	MRi.CCT	Form	CSF	Edema	Masseffect	Embolisation
260	2,00	1,00	,00	,00	1,00	,00
261	?	?	?	?	?	?
262	?	?	?	?	?	?
263	?	?	?	?	?	?
264	?	?	?	?	?	?
265	?	?	?	?	?	?
266	?	?	?	?	?	?
267	?	?	?	?	?	?
268	?	?	?	?	?	?
269	?	?	?	?	?	?
270	?	?	?	?	?	?
271	?	?	?	?	?	?
272	?	?	?	?	?	?
273	?	?	?	?	?	?
274	?	?	?	?	?	?
275	?	?	?	?	?	?
276	?	?	?	?	?	?
277	?	?	?	?	?	?
278	?	?	?	?	?	?
279	?	?	?	?	?	?
280	?	?	?	?	?	?
281	?	?	?	?	?	?
282	?	?	?	?	?	?
283	?	?	?	?	?	?
284	?	?	?	?	?	?
285	?	?	?	?	?	?
286	?	?	?	?	?	?
287	?	?	?	?	?	?
288	?	?	?	?	?	?
289	?	?	?	?	?	?
290	?	?	?	?	?	?
291	?	?	?	?	?	?
292	?	?	?	?	?	?
293	?	?	?	?	?	?
294	?	?	?	?	?	?

	Hypertonia	Adipositas	Heart.disorder	Lung.disorder	Liver.disorder	Kindey.disorder
260	,00	1,00	,00	,00	,00	,00
261	?	?	?	?	?	?
262	?	?	?	?	?	?
263	?	?	?	?	?	?
264	?	?	?	?	?	?
265	?	?	?	?	?	?
266	?	?	?	?	?	?
267	?	?	?	?	?	?
268	?	?	?	?	?	?
269	?	?	?	?	?	?
270	?	?	?	?	?	?
271	?	?	?	?	?	?
272	?	?	?	?	?	?
273	?	?	?	?	?	?
274	?	?	?	?	?	?
275	?	?	?	?	?	?
276	?	?	?	?	?	?
277	?	?	?	?	?	?
278	?	?	?	?	?	?
279	?	?	?	?	?	?
280	?	?	?	?	?	?
281	?	?	?	?	?	?
282	?	?	?	?	?	?
283	?	?	?	?	?	?
284	?	?	?	?	?	?
285	?	?	?	?	?	?
286	?	?	?	?	?	?
287	?	?	?	?	?	?
288	?	?	?	?	?	?
289	?	?	?	?	?	?
290	?	?	?	?	?	?
291	?	?	?	?	?	?
292	?	?	?	?	?	?
293	?	?	?	?	?	?
294	?	?	?	?	?	?

	Diabetes	Varicosis	ASA	Simpson.grade	OP.duration	intraOP.brain swelling
260	1,00	,00	2,00	2,00	342,00	,00
261	?	?	?	?	?	?
262	?	?	?	?	?	?
263	?	?	?	?	?	?
264	?	?	?	?	?	?
265	?	?	?	?	?	?
266	?	?	?	?	?	?
267	?	?	?	?	?	?
268	?	?	?	?	?	?
269	?	?	?	?	?	?
270	?	?	?	?	?	?
271	?	?	?	?	?	?
272	?	?	?	?	?	?
273	?	?	?	?	?	?
274	?	?	?	?	?	?
275	?	?	?	?	?	?
276	?	?	?	?	?	?
277	?	?	?	?	?	?
278	?	?	?	?	?	?
279	?	?	?	?	?	?
280	?	?	?	?	?	?
281	?	?	?	?	?	?
282	?	?	?	?	?	?
283	?	?	?	?	?	?
284	?	?	?	?	?	?
285	?	?	?	?	?	?
286	?	?	?	?	?	?
287	?	?	?	?	?	?
288	?	?	?	?	?	?
289	?	?	?	?	?	?
290	?	?	?	?	?	?
291	?	?	?	?	?	?
292	?	?	?	?	?	?
293	?	?	?	?	?	?
294	?	?	?	?	?	?

	Transfusion	Use.CUSA	Craniotomy	Sinus.lesion	Bleeding	Dura.closure
260	,00	1,00	1,00	,00	,00	1,00
261	?	?	?	?	?	?
262	?	?	?	?	?	?
263	?	?	?	?	?	?
264	?	?	?	?	?	?
265	?	?	?	?	?	?
266	?	?	?	?	?	?
267	?	?	?	?	?	?
268	?	?	?	?	?	?
269	?	?	?	?	?	?
270	?	?	?	?	?	?
271	?	?	?	?	?	?
272	?	?	?	?	?	?
273	?	?	?	?	?	?
274	?	?	?	?	?	?
275	?	?	?	?	?	?
276	?	?	?	?	?	?
277	?	?	?	?	?	?
278	?	?	?	?	?	?
279	?	?	?	?	?	?
280	?	?	?	?	?	?
281	?	?	?	?	?	?
282	?	?	?	?	?	?
283	?	?	?	?	?	?
284	?	?	?	?	?	?
285	?	?	?	?	?	?
286	?	?	?	?	?	?
287	?	?	?	?	?	?
288	?	?	?	?	?	?
289	?	?	?	?	?	?
290	?	?	?	?	?	?
291	?	?	?	?	?	?
292	?	?	?	?	?	?
293	?	?	?	?	?	?
294	?	?	?	?	?	?

	Tachosil.Fibrin	Transfusion.postOP	Seizure.therapy	Antibiotics.postOP	CSF.circulatory.disorder	Edema.postOP
260	1,00	,00	,00	,00	,00	,00
261	?	?	?	?	?	?
262	?	?	?	?	?	?
263	?	?	?	?	?	?
264	?	?	?	?	?	?
265	?	?	?	?	?	?
266	?	?	?	?	?	?
267	?	?	?	?	?	?
268	?	?	?	?	?	?
269	?	?	?	?	?	?
270	?	?	?	?	?	?
271	?	?	?	?	?	?
272	?	?	?	?	?	?
273	?	?	?	?	?	?
274	?	?	?	?	?	?
275	?	?	?	?	?	?
276	?	?	?	?	?	?
277	?	?	?	?	?	?
278	?	?	?	?	?	?
279	?	?	?	?	?	?
280	?	?	?	?	?	?
281	?	?	?	?	?	?
282	?	?	?	?	?	?
283	?	?	?	?	?	?
284	?	?	?	?	?	?
285	?	?	?	?	?	?
286	?	?	?	?	?	?
287	?	?	?	?	?	?
288	?	?	?	?	?	?
289	?	?	?	?	?	?
290	?	?	?	?	?	?
291	?	?	?	?	?	?
292	?	?	?	?	?	?
293	?	?	?	?	?	?
294	?	?	?	?	?	?

	Kind.of.bleeding	Infection	Revision1	Revision2	Revision3	Seizures.postOP
260	,00	,00	,00	,00	,00	,00
261	?	?	?	?	?	?
262	?	?	?	?	?	?
263	?	?	?	?	?	?
264	?	?	?	?	?	?
265	?	?	?	?	?	?
266	?	?	?	?	?	?
267	?	?	?	?	?	?
268	?	?	?	?	?	?
269	?	?	?	?	?	?
270	?	?	?	?	?	?
271	?	?	?	?	?	?
272	?	?	?	?	?	?
273	?	?	?	?	?	?
274	?	?	?	?	?	?
275	?	?	?	?	?	?
276	?	?	?	?	?	?
277	?	?	?	?	?	?
278	?	?	?	?	?	?
279	?	?	?	?	?	?
280	?	?	?	?	?	?
281	?	?	?	?	?	?
282	?	?	?	?	?	?
283	?	?	?	?	?	?
284	?	?	?	?	?	?
285	?	?	?	?	?	?
286	?	?	?	?	?	?
287	?	?	?	?	?	?
288	?	?	?	?	?	?
289	?	?	?	?	?	?
290	?	?	?	?	?	?
291	?	?	?	?	?	?
292	?	?	?	?	?	?
293	?	?	?	?	?	?
294	?	?	?	?	?	?

	Thro.Emb	D.insidipus	Dys.Aphasia	Sens.Hemi	Motor.Hemi	Other.symptoms
260	,00	,00	,00	,00	,00	,00
261	?	?	?	?	?	?
262	?	?	?	?	?	?
263	?	?	?	?	?	?
264	?	?	?	?	?	?
265	?	?	?	?	?	?
266	?	?	?	?	?	?
267	?	?	?	?	?	?
268	?	?	?	?	?	?
269	?	?	?	?	?	?
270	?	?	?	?	?	?
271	?	?	?	?	?	?
272	?	?	?	?	?	?
273	?	?	?	?	?	?
274	?	?	?	?	?	?
275	?	?	?	?	?	?
276	?	?	?	?	?	?
277	?	?	?	?	?	?
278	?	?	?	?	?	?
279	?	?	?	?	?	?
280	?	?	?	?	?	?
281	?	?	?	?	?	?
282	?	?	?	?	?	?
283	?	?	?	?	?	?
284	?	?	?	?	?	?
285	?	?	?	?	?	?
286	?	?	?	?	?	?
287	?	?	?	?	?	?
288	?	?	?	?	?	?
289	?	?	?	?	?	?
290	?	?	?	?	?	?
291	?	?	?	?	?	?
292	?	?	?	?	?	?
293	?	?	?	?	?	?
294	?	?	?	?	?	?

	ICU.stay	NCH.stay	Total.duration	Rehabilitation	Radiation	Recurrence
260	1,00	10,00	11,00	1,00	,00	,00
261	?	?	?	?	?	?
262	?	?	?	?	?	?
263	?	?	?	?	?	?
264	?	?	?	?	?	?
265	?	?	?	?	?	?
266	?	?	?	?	?	?
267	?	?	?	?	?	?
268	?	?	?	?	?	?
269	?	?	?	?	?	?
270	?	?	?	?	?	?
271	?	?	?	?	?	?
272	?	?	?	?	?	?
273	?	?	?	?	?	?
274	?	?	?	?	?	?
275	?	?	?	?	?	?
276	?	?	?	?	?	?
277	?	?	?	?	?	?
278	?	?	?	?	?	?
279	?	?	?	?	?	?
280	?	?	?	?	?	?
281	?	?	?	?	?	?
282	?	?	?	?	?	?
283	?	?	?	?	?	?
284	?	?	?	?	?	?
285	?	?	?	?	?	?
286	?	?	?	?	?	?
287	?	?	?	?	?	?
288	?	?	?	?	?	?
289	?	?	?	?	?	?
290	?	?	?	?	?	?
291	?	?	?	?	?	?
292	?	?	?	?	?	?
293	?	?	?	?	?	?
294	?	?	?	?	?	?

	Recurrence1	Daeth	Karnofsky.score.pre	Karnofsky.score.post1.3.month	Karnofsky.score.post6.12.month	Difference.K3.K1
260	,00	,00	80,00	70,00	80,00	,00
261	?	?	?	?	?	?
262	?	?	?	?	?	?
263	?	?	?	?	?	?
264	?	?	?	?	?	?
265	?	?	?	?	?	?
266	?	?	?	?	?	?
267	?	?	?	?	?	?
268	?	?	?	?	?	?
269	?	?	?	?	?	?
270	?	?	?	?	?	?
271	?	?	?	?	?	?
272	?	?	?	?	?	?
273	?	?	?	?	?	?
274	?	?	?	?	?	?
275	?	?	?	?	?	?
276	?	?	?	?	?	?
277	?	?	?	?	?	?
278	?	?	?	?	?	?
279	?	?	?	?	?	?
280	?	?	?	?	?	?
281	?	?	?	?	?	?
282	?	?	?	?	?	?
283	?	?	?	?	?	?
284	?	?	?	?	?	?
285	?	?	?	?	?	?
286	?	?	?	?	?	?
287	?	?	?	?	?	?
288	?	?	?	?	?	?
289	?	?	?	?	?	?
290	?	?	?	?	?	?
291	?	?	?	?	?	?
292	?	?	?	?	?	?
293	?	?	?	?	?	?
294	?	?	?	?	?	?

	Diffenrence.K3.K2	K2K1	Agegroup1	Agegroup2
260	10,00	-10,00	4,00	3,00
261	?	?	?	?
262	?	?	?	?
263	?	?	?	?
264	?	?	?	?
265	?	?	?	?
266	?	?	?	?
267	?	?	?	?
268	?	?	?	?
269	?	?	?	?
270	?	?	?	?
271	?	?	?	?
272	?	?	?	?
273	?	?	?	?
274	?	?	?	?
275	?	?	?	?
276	?	?	?	?
277	?	?	?	?
278	?	?	?	?
279	?	?	?	?
280	?	?	?	?
281	?	?	?	?
282	?	?	?	?
283	?	?	?	?
284	?	?	?	?
285	?	?	?	?
286	?	?	?	?
287	?	?	?	?
288	?	?	?	?
289	?	?	?	?
290	?	?	?	?
291	?	?	?	?
292	?	?	?	?
293	?	?	?	?
294	?	?	?	?

	Symptoms.duration	Number.tumors	Number.symptoms
260	2,00	2,00	2,00
261	3,00	2,00	2,00
262	4,00	2,00	2,00
263	2,00	,00	1,00
264	2,00	3,00	4,00
265	2,00	,00	2,00
266	1,00	1,00	,00
267	1,00	,00	,00
268	2,00	1,00	1,00
269	6,00	2,00	3,00
270	1,00	,00	1,00
271	1,00	2,00	,00
272	1,00	4,00	1,00
273	1,00	3,00	2,00
274	6,00	3,00	2,00
275	2,00	,00	4,00
276	1,00	1,00	,00
277	4,00	,00	2,00
278	1,00	,00	2,00
279	4,00	3,00	3,00
280	1,00	,00	,00
281	4,00	,00	2,00
282	5,00	,00	1,00
283	5,00	3,00	1,00
284	3,00	,00	1,00
285	2,00	1,00	1,00
286	3,00	1,00	1,00
287	4,00	1,00	2,00
288	2,00	,00	2,00
289	2,00	3,00	3,00
290	2,00	,00	1,00
291	5,00	3,00	1,00
292	3,00	4,00	3,00
293	3,00	3,00	2,00
294	2,00	1,00	2,00

	Operation.time	Volume.transfosion
260	3,00	,00
261	2,00	,00
262	2,00	,00
263	4,00	566,00
264	3,00	,00
265	4,00	,00
266	3,00	,00
267	4,00	,00
268	3,00	,00
269	3,00	,00
270	4,00	1698,00
271	4,00	,00
272	2,00	,00
273	2,00	,00
274	4,00	,00
275	2,00	,00
276	1,00	283,00
277	3,00	,00
278	2,00	,00
279	4,00	,00
280	2,00	,00
281	4,00	,00
282	2,00	,00
283	4,00	566,00
284	3,00	,00
285	2,00	,00
286	1,00	,00
287	3,00	,00
288	3,00	,00
289	4,00	,00
290	3,00	,00
291	2,00	,00
292	2,00	,00
293	2,00	,00
294	2,00	566,00

	Volume.transfusion.postOP	Rebleeding	ICU.stay.groups
260	,00	,00	1,00
261	,00	,00	1,00
262	,00	,00	1,00
263	,00	,00	1,00
264	,00	,00	1,00
265	,00	,00	1,00
266	,00	,00	1,00
267	,00	,00	1,00
268	,00	,00	1,00
269	,00	,00	2,00
270	,00	,00	1,00
271	,00	,00	1,00
272	,00	,00	1,00
273	,00	,00	1,00
274	,00	,00	3,00
275	,00	,00	3,00
276	,00	,00	1,00
277	,00	,00	1,00
278	,00	,00	1,00
279	,00	,00	1,00
280	,00	,00	1,00
281	,00	?	?
282	,00	?	?
283	,00	?	?
284	,00	?	?
285	,00	?	?
286	,00	?	?
287	,00	?	?
288	,00	?	?
289	,00	?	?
290	,00	?	?
291	,00	?	?
292	,00	?	?
293	,00	?	?
294	,00	?	?

	NCH.stay.groups	Number.symptoms.postOP	Symptoms.postOP	First.symptome.groups
260	2,00	,00	,00	4,00
261	1,00	,00	,00	1,00
262	2,00	1,00	1,00	6,00
263	2,00	,00	,00	4,00
264	2,00	,00	,00	3,00
265	1,00	,00	,00	4,00
266	1,00	,00	,00	9,00
267	2,00	1,00	1,00	,00
268	1,00	,00	,00	8,00
269	2,00	1,00	1,00	1,00
270	2,00	1,00	1,00	2,00
271	2,00	1,00	1,00	,00
272	3,00	3,00	1,00	2,00
273	2,00	,00	,00	5,00
274	4,00	4,00	1,00	8,00
275	1,00	2,00	1,00	4,00
276	1,00	,00	,00	,00
277	1,00	,00	,00	4,00
278	1,00	,00	,00	1,00
279	2,00	,00	,00	6,00
280	1,00	,00	,00	,00
281	1,00	3,00	1,00	7,00
282	1,00	,00	,00	2,00
283	3,00	1,00	1,00	7,00
284	2,00	,00	,00	7,00
285	2,00	,00	,00	2,00
286	1,00	,00	,00	4,00
287	2,00	2,00	1,00	7,00
288	1,00	,00	,00	4,00
289	2,00	2,00	1,00	2,00
290	1,00	,00	,00	6,00
291	1,00	,00	,00	7,00
292	1,00	,00	,00	3,00
293	2,00	2,00	1,00	9,00
294	1,00	,00	,00	5,00

	Neurological.disorder	Histology.groups	Revision.groups	Localisation.revision
260	1,00	1,00	,00	2,00
261	1,00	4,00	,00	1,00
262	1,00	5,00	,00	1,00
263	1,00	5,00	,00	1,00
264	1,00	1,00	1,00	1,00
265	1,00	1,00	,00	1,00
266	,00	1,00	,00	1,00
267	,00	1,00	,00	2,00
268	1,00	5,00	,00	1,00
269	1,00	4,00	,00	1,00
270	1,00	5,00	,00	2,00
271	,00	4,00	,00	2,00
272	1,00	4,00	,00	1,00
273	1,00	5,00	1,00	1,00
274	1,00	2,00	1,00	1,00
275	1,00	1,00	,00	1,00
276	,00	3,00	,00	1,00
277	1,00	1,00	,00	1,00
278	1,00	2,00	,00	1,00
279	1,00	1,00	,00	1,00
280	,00	1,00	,00	1,00
281	1,00	1,00	1,00	1,00
282	1,00	1,00	,00	2,00
283	1,00	2,00	1,00	1,00
284	1,00	5,00	,00	1,00
285	1,00	3,00	,00	1,00
286	1,00	2,00	,00	2,00
287	1,00	1,00	,00	2,00
288	1,00	3,00	,00	1,00
289	1,00	1,00	,00	1,00
290	1,00	1,00	,00	1,00
291	1,00	5,00	,00	1,00
292	1,00	5,00	,00	1,00
293	1,00	4,00	,00	1,00
294	1,00	4,00	1,00	1,00

	Masseffect.revision	Simpson.revision	antiepileptc.therapy.revision
260	1,00	1,00	,00
261	?	?	?
262	?	?	?
263	?	?	?
264	?	?	?
265	?	?	?
266	?	?	?
267	?	?	?
268	?	?	?
269	?	?	?
270	?	?	?
271	?	?	?
272	?	?	?
273	?	?	?
274	?	?	?
275	?	?	?
276	?	?	?
277	?	?	?
278	?	?	?
279	?	?	?
280	?	?	?
281	?	?	?
282	?	?	?
283	?	?	?
284	?	?	?
285	?	?	?
286	?	?	?
287	?	?	?
288	?	?	?
289	?	?	?
290	?	?	?
291	?	?	?
292	?	?	?
293	?	?	?
294	?	?	?

	Post.revision.symptoms	Recurrence.revision	ASA.class.4	Kd_disorder
260	,00	,00	2,00	,00
261	,00	,00	2,00	,00
262	,00	,00	2,00	,00
263	,00	,00	2,00	1,00
264	,00	,00	2,00	,00
265	,00	,00	2,00	,00
266	,00	,00	2,00	,00
267	,00	,00	2,00	,00
268	,00	,00	3,00	,00
269	,00	,00	3,00	,00
270	,00	1,00	3,00	1,00
271	,00	,00	3,00	,00
272	1,00	,00	3,00	.
273	,00	1,00	3,00	,00
274	1,00	1,00	3,00	1,00
275	,00	,00	2,00	,00
276	,00	,00	3,00	,00
277	,00	,00	2,00	,00
278	,00	,00	2,00	,00
279	,00	,00	3,00	,00
280	,00	,00	2,00	1,00
281	1,00	,00	1,00	1,00
282	,00	,00	2,00	,00
283	1,00	,00	3,00	1,00
284	,00	,00	2,00	,00
285	,00	,00	2,00	,00
286	,00	1,00	2,00	,00
287	1,00	,00	3,00	1,00
288	,00	,00	2,00	,00
289	1,00	,00	3,00	.
290	,00	,00	2,00	,00
291	,00	,00	3,00	,00
292	,00	,00	3,00	.
293	1,00	,00	2,00	,00
294	,00	,00	2,00	,00

	age_disorder	K1_cut	K1_3gr	Kd_3gr	ASA_di
260	,00	,00	3,00	2,00	,00
261	,00	,00	2,00	?	?
262	,00	,00	2,00	?	?
263	,00	,00	3,00	?	?
264	,00	,00	2,00	?	?
265	,00	,00	2,00	?	?
266	,00	,00	2,00	3,00	,00
267	,00	,00	3,00	2,00	,00
268	1,00	,00	2,00	2,00	1,00
269	,00	,00	3,00	3,00	1,00
270	,00	,00	2,00	1,00	1,00
271	,00	,00	2,00	3,00	1,00
272	1,00	1,00	1,00	.	1,00
273	,00	,00	2,00	2,00	1,00
274	1,00	1,00	1,00	1,00	1,00
275	1,00	,00	2,00	2,00	,00
276	,00	,00	3,00	2,00	1,00
277	,00	,00	2,00	3,00	,00
278	,00	,00	3,00	2,00	,00
279	1,00	,00	2,00	2,00	1,00
280	1,00	,00	2,00	1,00	,00
281	,00	,00	3,00	?	?
282	,00	,00	2,00	?	?
283	,00	,00	3,00	?	?
284	,00	,00	3,00	?	?
285	1,00	,00	2,00	?	?
286	,00	,00	2,00	?	?
287	1,00	,00	2,00	?	?
288	1,00	,00	2,00	?	?
289	1,00	,00	2,00	?	?
290	,00	,00	3,00	?	?
291	1,00	,00	2,00	?	?
292	1,00	1,00	1,00	?	?
293	1,00	,00	2,00	?	?
294	,00	1,00	1,00	?	?

	Simpson_2gr	WHO_di	Age_cut	Localisation_di
260	,00	,00	,00	1,00
261	,00	1,00	,00	.
262	,00	,00	,00	1,00
263	1,00	,00	,00	1,00
264	,00	,00	,00	1,00
265	,00	,00	,00	1,00
266	,00	,00	,00	1,00
267	,00	,00	,00	1,00
268	,00	,00	1,00	1,00
269	,00	1,00	,00	,00
270	,00	1,00	,00	1,00
271	,00	1,00	,00	1,00
272	,00	1,00	1,00	,00
273	,00	1,00	,00	1,00
274	,00	,00	1,00	1,00
275	,00	,00	1,00	1,00
276	,00	,00	,00	.
277	,00	,00	,00	1,00
278	,00	,00	,00	.
279	,00	,00	1,00	1,00
280	,00	1,00	,00	,00
281	,00	,00	,00	,00
282	,00	,00	,00	1,00
283	,00	,00	,00	.
284	,00	,00	,00	,00
285	,00	,00	1,00	,00
286	1,00	,00	,00	1,00
287	1,00	,00	1,00	1,00
288	,00	,00	,00	1,00
289	,00	,00	1,00	,00
290	,00	,00	,00	1,00
291	1,00	,00	1,00	1,00
292	,00	,00	1,00	.
293	1,00	1,00	1,00	1,00
294	,00	1,00	,00	,00

	K3_3gr	Age3gr	RF_r	Reha_r	K3_cut
260	3,00	1,00	1,00	1,00	,00
261	3,00	1,00	1,00	1,00	?
262	2,00	2,00	1,00	,00	?
263	2,00	1,00	,00	1,00	?
264	3,00	2,00	1,00	1,00	?
265	2,00	2,00	,00	1,00	?
266	3,00	2,00	1,00	1,00	?
267	3,00	2,00	,00	1,00	?
268	2,00	2,00	1,00	1,00	?
269	3,00	1,00	1,00	,00	?
270	1,00	2,00	,00	1,00	?
271	3,00	1,00	1,00	,00	?
272	.	3,00	1,00	2,00	?
273	2,00	2,00	1,00	1,00	?
274	1,00	2,00	1,00	2,00	?
275	2,00	3,00	,00	1,00	?
276	3,00	1,00	1,00	1,00	?
277	3,00	2,00	,00	,00	?
278	3,00	1,00	,00	1,00	?
279	2,00	2,00	1,00	2,00	?
280	1,00	2,00	,00	1,00	?
281	2,00	2,00	,00	1,00	?
282	3,00	2,00	,00	1,00	?
283	2,00	2,00	1,00	2,00	?
284	3,00	2,00	,00	1,00	?
285	3,00	2,00	1,00	,00	?
286	2,00	2,00	1,00	,00	?
287	1,00	2,00	1,00	2,00	?
288	2,00	2,00	,00	,00	?
289	.	3,00	1,00	1,00	?
290	3,00	2,00	,00	,00	?
291	3,00	3,00	1,00	1,00	?
292	.	3,00	1,00	1,00	?
293	2,00	3,00	1,00	2,00	?
294	3,00	1,00	1,00	1,00	?

	Localisation3gr	localisation2gr	Uni_di	age7groups
260	1,00	1,00	,00	3,00
261	.	.	,00	3,00
262	1,00	1,00	,00	4,00
263	1,00	1,00	,00	2,00
264	1,00	1,00	,00	4,00
265	1,00	1,00	,00	4,00
266	1,00	1,00	,00	4,00
267	1,00	1,00	1,00	4,00
268	2,00	.	,00	5,00
269	3,00	,00	1,00	2,00
270	2,00	.	,00	4,00
271	1,00	1,00	,00	3,00
272	3,00	,00	1,00	6,00
273	2,00	.	1,00	4,00
274	1,00	1,00	1,00	5,00
275	1,00	1,00	1,00	6,00
276	.	.	,00	3,00
277	1,00	1,00	,00	3,00
278	.	.	,00	3,00
279	1,00	1,00	,00	5,00
280	3,00	,00	,00	5,00
281	3,00	,00	,00	4,00
282	1,00	1,00	,00	4,00
283	.	.	1,00	4,00
284	3,00	,00	1,00	4,00
285	3,00	,00	,00	5,00
286	1,00	1,00	,00	4,00
287	1,00	1,00	1,00	5,00
288	1,00	1,00	,00	5,00
289	3,00	,00	1,00	6,00
290	1,00	1,00	,00	4,00
291	1,00	1,00	,00	6,00
292	.	.	,00	6,00
293	1,00	1,00	1,00	6,00
294	3,00	,00	,00	3,00
